# Supplementary material for: Sickness absence after carpal tunnel release: a multicentre prospective cohort study
Source: BMJ Open. 2021 Feb 10;11(2):e041656. doi: 10.1136/bmjopen-2020-041656 (PMC7878133; doi:10.1136/bmjopen-2020-041656)
Supplement: Supplementary data [file bmjopen-2020-041656supp001.pdf]

Newington et al. (2020). Sickness absence after carpal tunnel release: a multicentre prospective cohort study

Development of the REACTS study questionnaire: Reasoning for item inclusion

Questions were chosen to capture information on variables that might be expected to influence return to work after CTR.

Demographic factors

General demographic information was requested including date of birth, sex and hand dominance. Other studies have found that older age was associated with poorer work outcomes after CTR [1, 2], while no clear sex effect has been shown [3, 4]. Hand dominance in relation to side of surgery is rarely reported in CTR studies, however, surgery to the non-dominant hand has been linked to quicker and more complete resolution of CTS symptoms [5].

Carpal tunnel release planning

Information was collected on the expected date of CTR, side of surgery, availability of occupational health services and the patient's expectations about time off work post-surgery. Expected duration of work absence has been identified as a determinant of return to work time in previous CTR studies [2, 6].

Occupational factors

Participants were asked to list their main occupation and the industry in which they work (examples were provided to facilitate the response). This information was processed using the UK Office for National Statistics Standard Occupational Classification [7] and Computer Assisted Structured Coding tool (Cascot) [8] to generate manual and non-manual categories. Cases where the coding match was confirmed as less than 64% were reviewed by the lead researcher and coded by hand [8]. This was checked by the department data manager and any queries resolved through discussion.

Participants were asked to categorise their employment type as: employed (permanent contract), employed (temporary/renewable contract), zero hours contract and self-employed. Our systematic review found earlier return to work times for self-employed individuals compared to those who were employed [9]; but this was only investigated in two

studies [10, 11]. The additional sub-categories for employment type listed above and a separate question about sick-pay entitlement were included to allow the impact of work contract type to be explored [12]. Participants were also asked how many hours they usually worked each week and over how many days; this information was collected for the participant's main job and any other routine paid work. The total number of work hours per week was calculated by combining the hours for main and additional jobs.

Occupational activities that load the upper limb and potential work stressors were asked as a series of yes/no questions following the format of a recent multi-centre RCT exploring management of non-specific distal arm pain [13]. These questions originated in the Job Content Questionnaire, designed to assess psychological and physical aspects of work [14]. Activities included: computer use, tasks involving repeated wrist/finger movement, holding vibrating tools, lifting more than 5 or 10kgs, pushing/pulling a heavy weight, working with the neck flexed or rotated, and driving. Our systematic review found that manual workers took longer to return to work than non-manual workers [9] and these questions were used to determine the self-reported level of upper limb manual activity involved in each participant's job.

Potential psychosocial work stressors were also assessed. These included piecemeal work, activity targets and bonuses, and tight deadlines. As the first three items all concerned payment for results, these were combined for the analyses. Participants were also asked whether they found their main job demanding on their hands/wrist and whether their boss/colleagues were supportive. Both questions were scored on a 0-10 scale as reported previously in a study of sick leave duration after endoscopic CTR [15]. These were dichotomised as supportive (7-10) and neutral/unsupportive (0-6). A question about general job satisfaction was also included later in this section of the questionnaire, with the Likert response options: very satisfied, satisfied/fairly satisfied, dissatisfied and very dissatisfied [15, 16]. The last two options were condensed to give three categories for the analyses.

To assess self-reported work function, participants were asked to complete the work performance section of the Michigan Hand Questionnaire (MHQ) [17]. This patient reported outcome measure is frequently used in upper limb clinical practice and research and has

been validated for use with CTS and CTR populations [18]. Permission was granted for the MHQ to be used in the study. The questionnaire asked participants to recall how much difficulty they had with general work tasks over the past four weeks in relation to problems with their hands/wrists, for example: needing to shorten their working day, taking longer to complete tasks or needing to take breaks. Using the standard scoring, each question was completed on a Likert scale of: always, often, sometimes, rarely and never, and combined to give a score from 0-100, with 100 representing no problems with work functioning [19]. Participants were also asked whether they had taken any periods of sickness absence from work over the previous four weeks, both related to the hand/wrist problem, or for any other problem.

General health

Seven general health questions were included to capture information on comorbidities, physical and mental health and somatisation. Self-reported health was assessed using the first SF-36 question: In general, would you say your health is – excellent, very good, good, fair, poor [20]. This was taken from the original SF-36 version, which is free from licence charges and was dichotomised as excellent/very good/good and fair/poor for the analyses.

Participants were asked their height and weight to enable the calculation of BMI (body mass index; weight in kilograms/height in metres squared). This was categorised using standard WHO classification: underweight (BMI <18.5), normal weight (BMI 18.5-24.9), overweight (BMI 25.0-29.9) and obese (BMI ≥30.0) [21]. Smoking status was categorised as those who have never smoked regularly, those who have smoked in the past and those who regularly smoke, with the latter two categories combined for the analyses. Previous studies have found that obesity (BMI ≥30) was linked to poorer work outcomes [1] and smoking was linked to poorer clinical outcomes after CTR [22].

A list of common health problems and their impact on general activities was assessed using the Self-Administered Comorbidity Questionnaire [23]. Participants were asked first to select whether they have any of the 14 medical conditions and if so whether this limited their activities. All medical conditions were worded in an accessible format, as evaluated by the patient advisory group. Responses were analysed as the number of comorbidities and

the number of disabling comorbidities using the scale: 0, 1, ≥2. Mental health was assessed using the mental health and vitality questions from the licence-free version of the SF-36 [20]. One modification was made to change the wording of the question 'Did you feel full of pep?' to 'Did you feel full of get-up-and-go?' for the UK rather than US setting. The questions were used to calculate the summary score from 0-100 (where 100 represents no disability).

Somatisation was assessed using a subset of five questions from the Four-Dimensional Symptom Questionnaire [24] as previously reported in UK cohort studies of health and employment [25] and upper limb pain in primary care [26]. The number of symptoms that were rated by the participant as at least moderately distressing were used to create the analysis categories of: 0, 1, ≥2 symptoms.

Hand and wrist symptoms and function

Katz & Stirrat hand diagrams were included for the participants to indicate where on their hand(s) they experience pain and/or tingling and numbness [27]. This self-administered tool can be used clinically as part of the CTS diagnosis process using the scoring system modified by Calfee et al. [28]. A question on symptom duration was also included and categorised as less than 3 months, 3-6 months, 6-12 months and more than a year. This was dichotomised to ≤1 year and >1 year for analysis. All participants were expected to have clinically diagnosed CTS as they were undergoing CTR, but participants were asked to answer for both hands, so that symptoms in the non-operated hand were also assessed. This was used to define bilateral or unilateral symptoms. Hand diagram scores were dichotomised according to a stringent definition of CTS (classic and probable) and unlikely CTS (possible and unlikely).

The CTS-6 questionnaire was included to assess the severity of CTS symptoms [29]. This tool is a shorter version of the Boston Carpal Tunnel Questionnaire [30] and has been assessed for use pre- and post CTR surgery. The six questions explore the severity of pain and numbness, whether this occurs during the daytime or at night, and whether this wakes the individual. Participants were asked to complete these questions separately for each hand using a 5-point Likert scale. Using the standard scoring criteria, responses for each item

were combined to give a mean score ranging from 1-5, with 5 representing the highest severity of symptoms. If one response was missing (per hand) this was imputed using the mean score of the remaining responses [29].

Hand function was assessed using the MHQ sub-sections on unilateral hand function (asked for each hand), satisfaction with hand function (asked for each hand), and ability to perform unilateral and bilateral activities of daily living (ADLs) [17]. The MHQ summary question relating to the level of satisfaction with the appearance of each hand was also included. All questions were scored on the 5-point Likert scales provided and used the standard wording and scoring to enable comparison with other study populations. Possible scores for each sub-section range from 1-100, with 100 representing no problems or the highest level of satisfaction. Missing data were imputed according to the MHQ guidelines, which allow the scale to be calculated if more than 50% of the questions for each sub-section have been completed [19].

Health beliefs

The remaining questions related to health beliefs. Beliefs about the cause of symptoms and likely prognosis have been identified as key themes in health-seeking behaviour for CTS [31] and upper limb pain [26], and blaming oneself for the hand problem has been associated with long durations of sick leave after endoscopic CTR [15]. The participant's expectations for being able to use the affected hand normally within 3 months of surgery, fear of long-term hand problems, blaming oneself for the hand problem and the perceived level of support available from friends and family were assessed. Responses were rated on a 0-10 scale as reported by Hansen et al. [15]. All responses were converted to a unidirectional scale with 10 being the best outcome, and were dichotomised as neutral/negative response 0-6 and positive response 7-10.

Participants were also asked to agree/disagree (via a 5-point Likert scale) with a series of seven questions about the believed cause of their symptoms. Using previously reported methods, the responses were dichotomised to those who agreed (agree/strongly agree) and those who did not agree (neither agree nor disagree/disagree/disagree strongly) with each statement [26]. The first two questions were combined to generate six items: 1) I think I was

born with a weakness in this part of my body/problems like this run in my family; 2) my problem was caused by work; 3) my problem probably wasn't caused by work, but work made it worse; 4) I have a lot of stress in my life and that has made my problem a lot worse; 5) a lack of exercise probably contributed to my problem; 6) as you get older, parts of the body wear out and problems like mine are likely [26].

Finally, the abbreviated Pain Catastrophizing Scale was included, which provides insight about the participant's pain beliefs [32]. Responses were dichotomised to those who reported catastrophising pain thoughts and feelings to at least a moderate degree in response to all questions, and those who did not.

References

1. Parot-Schinkel E, Roquelaure Y, Ha C et al. Factors affecting return to work after carpal tunnel syndrome surgery in a large french cohort. *Arch of Phys Med Rehabil* 2011;92(11):1863-69.
2. Peters S, Johnston V, Hines S et al. Prognostic factors for return-to-work following surgery for carpal tunnel syndrome: a systematic review. *JBI Database of Systematic Reviews and Implementation Reports* 2016;14(9):135-216.
3. Bekkelund SI, Pierre-Jerome C, Torbergsen T et al. Impact of occupational variables in carpal tunnel syndrome. *Acta Neurol Scand* 2001;103(3):193-97.
4. Atroshi I, Zhou C, Joud A et al. Sickness absence from work among persons with new physician-diagnosed carpal tunnel syndrome: a population-based matched-cohort study. *PLoS One* 2015;10(3):e0119795.
5. Tang Q, Lai W, Tay S. The effect of hand dominance on patient-reported outcomes of carpal tunnel release in patients with bilateral carpal tunnel syndrome. *Journal of Hand Surgey Asian Pacific Volume* 2017;22(3):303-08.
6. Cowan J, Makanji H, Mudgal C et al. Determinants of return to work after carpal tunnel release. *J Hand Surg Am* 2012;37(1):18-27.
7. Office for National Statistics. Standard occupational classification for the UK: https://www.ons.gov.uk/methodology/classificationsandstandards/standardoccupationalclassificationsoc/soc2010. Accessed 08/06/2018.
8. Warwick Institute for Employment Research. Cascot: Computer assisted structured coding tool https://warwick.ac.uk/fac/soc/ier/software/cascot/. Accessed 15/05/2019.
9. Newington L, Stevens M, Warwick D et al. Sickness absence after carpal tunnel release: a systematic review. *Scandinavian Journal of Work, Environment and Health* 2018;44(6):557-67.
10. De Kesel R, Donceel P, De Smet L. Factors influencing return to work after surgical treatment for carpal tunnel syndrome. *Occup Med* 2008;58(3):187-90.

11. Chaise F, Bellemere P, Fril JP et al. Return-to-work interval and surgery for carpal tunnel syndrome. Results of a prospective series of 233 patients. *J Hand Surg Br* 2004;29(6):568-70.
12. Tran M, Sokas RK. The gig economy and contingent work: an occupational health assessment. *Journal of Occupational and Environmental Medicine* 2017;59(4):E63-E66.
13. Jones GT, Mertens K, Macfarlane GJ et al. Maintained physical activity and physiotherapy in the management of distal upper limb pain - a protocol for a randomised controlled trial (the arm pain trial). *BMC Musculoskelet Disord* 2014;15:71.
14. Karasek R, Brisson C, Kawakami N et al. The Job Content Questionnaire (JCQ): an instrument for internationally comparative assessments of psychosocial job characteristics. *Journal of occupational health psychology* 1998;3(4):322-55.
15. Hansen TB, Dalsgaard J, Meldgaard A et al. A prospective study of prognostic factors for duration of sick leave after endoscopic carpal tunnel release. *BMC Musculoskelet Disord* 2009;10:144.
16. D'Angelo S, Coggon D, Harris EC et al. Job dissatisfaction and the older worker: baseline findings from the Health and Employment After Fifty study. *Occupational and environmental medicine* 2016;73(8):512-19.
17. Chung KC, Pillsbury MS, Walters MR et al. Reliability and validity testing of the Michigan Hand Outcomes Questionnaire. *J Hand Surg Am* 1998;23(4):575-87.
18. Hadi M, Gibbons E, Fitzpatrick R. A structured review of patient-reported outcome measures for procedures for carpal tunnel syndrome. UK: Patient-Reported Outcome Measures Group, University of Oxford; 2011.
19. University of Michigan Medical School. The Michigan Hand Questionnaire: http://mhq.lab.medicine.umich.edu/. Accessed 04/04/2019.
20. Ware J, Sherbourne C. The MOS 36-item short-form health survey (SF-36): I. conceptual framework and item selection. *Medical Care* 1992;30:473-83
21. World Health Organization. Obesity: preventing and managing the global epidemic. Report of a WHO consultation. *World Health Organization technical report series* 2000;894:1-253.
22. Dahlin E, Zimmerman M, Bjorkman A et al. Impact of smoking and preoperative electrophysiology on outcome after open carpal tunnel release. *Journal of Plastic Surgery & Hand Surgery* 2017;51(5):329-35.
23. Sangha O, Stucki G, Liang MH et al. The self-administered comorbidity questionnaire: a new method to assess comorbidity for clinical and health services research *Arthritis & Rheumatism* 2003;49(2):156-63.
24. Terluin B, van Marwijk HW, Adèr HJ et al. The Four-Dimensional Symptom Questionnaire (4DSQ): a validation study of a multidimensional self-report questionnaire to assess distress, depression, anxiety and somatization. *BMC Psychiatry* 2006;6(1):34.
25. Palmer KT, Walker-Bone K, Harris EC et al. Health and Employment after Fifty (HEAF): a new prospective cohort study. *BMC public health* 2015;15:1071.
26. Palmer K, Calnan M, Wainwright D et al. Upper limb pain in primary care: health beliefs, somatic distress, consulting and patient satisfaction. *Family Practice* 2006;23(6):609-17.

27. Katz JN, Stirrat CR. A self-administered hand diagram for the diagnosis of carpal tunnel. *J Hand Surg Am* 1990;15:360-63.
28. Calfee RP, Dale AM, Ryan D et al. Performance of simplified scoring systems for hand diagrams in carpal tunnel syndrome screening. *J Hand Surg Am* 2012;37(1):10-17.
29. Atroshi I, Lyren PE, Ornstein E et al. The six-item CTS symptoms scale and palmar pain scale in carpal tunnel syndrome. *J Hand Surg Am* 2011;36(5):788-94.
30. Levine DW, Simmons BP, Koris MJ et al. A self-administered questionnaire for the assessment of severity of symptoms and functional status in carpal tunnel syndrome. *J Bone Joint Surg Am* 1993;75:1585-92.
31. Martin H. Patients' health beliefs and adaptation to carpal tunnel syndrome based on duration of symptomatic presentation. *J Hand Ther* 2007;20(1):29-35.
32. Bot AG, Becker SJ, Bruijnzeel H et al. Creation of the abbreviated measures of the Pain Catastrophizing Scale and the Short Health Anxiety Inventory: the PCS-4 and SHAI-5. *Journal of Musculoskeletal Pain* 2014;22(2):145-51.
