# Supplementary material for: Sickness absence after carpal tunnel release: a multicentre prospective cohort study
Source: BMJ Open. 2021 Feb 10;11(2):e041656. doi: 10.1136/bmjopen-2020-041656 (PMC7878133; doi:10.1136/bmjopen-2020-041656)
Supplement: Supplementary data [file bmjopen-2020-041656supp002.pdf]

Site

Please fill in today's date

d d m m y y

Before Your Carpal Tunnel Release Surgery

Return to Employment After Carpal Tunnel Release Surgery (REACTS)

ln@mrc.soton.ac.uk | 023 8077 7624
Arthritis Research UK – MRC Centre for Musculoskeletal Health and Work
MRC Lifecourse Epidemiology Unit, University of Southampton
Southampton General Hosptial (MP 95), SO16 6YD

IRAS reference: 209840

CONSENT FORM (IRAS reference: 209840)

You should complete this form after you have read the Participant Information Sheet.

REACTS: Return to employment after carpal tunnel release surgery

Thank you for considering taking part in this research. If you have any questions arising from the Participant Information Sheet, please ask the research team before you decide whether to take part.

Please initial the boxes if you agree with each statement

1. I have read the Participant Information Sheet (version 2.0; 06.12.16) and have had the opportunity to ask questions about the study. ☐

2. I meet the criteria for being involved in this study: ☐
   - Aged over 18 and referred for carpal tunnel release surgery
   - Routinely work in paid employment for at least 20 hours per week
   - Plan to return to work after carpal tunnel release surgery
   - Have not previously had carpal tunnel release surgery on either hand
   - Have not previously had a serious injury to the same wrist/hand that will have the carpal tunnel release operation

3. I agree to take part in this research and agree for my data to be used for the purposes explained in the Participant Information Sheet (version 2.0; 06.12.16). I understand that this information will be handled in accordance with the terms of the UK Data Protection Act 1998. ☐
   a. I agree for the REACTS research team to access pre-operative test results concerning my hand and wrist symptoms. No other information will be accessed. ☐
   b. I agree for the REACTS research team to access my carpal tunnel release surgical record. No other information will be accessed. ☐

4. I understand that if I decide at any time during the research that I no longer wish to take part, I can notify the researchers and withdraw from the study immediately, without giving a reason. If I do, I understand that I can ask for any contribution I have already made to be removed from the study, up to the time when I have completed the final questionnaire. ☐

Signature ______________________________ Date ______ / ______ / ______

Name ______________________________ Phone ____________________
(please print) *(only to be used if we lose touch)*

Postal address ____

____

Email address ____
(please print)

University Hospital Southampton NHS Foundation Trust | REACTS | Arthritis Research UK MRC centre for musculoskeletal health & work | UNIVERSITY OF Southampton

ADDITIONAL QUESTIONS

Please circle one response for each question

I prefer to receive the next two questionnaires by	**Post**	**Email**	**Don't mind**
I prefer to receive correspondence about the study by	**Post**	**Email**	**Don't mind**
I would like to be notified of the findings from this research	**Yes**	**No**	
I am happy to be contacted about the next stage of the research, which will involve a one-off discussion with the lead researcher	**Yes**	**No**	
I am happy to be contacted about other studies related to this research	**Yes**	**No**	

When the research team receives your completed questionnaire and consent form, we will sign it below and return a copy to you for your records.

Researcher signature ____ Date ____ / ____ / ____

Researcher name ____

University of Southampton research supervisors:
Professor Karen Walker-Bone | Professor Jo Adams | Professor David Warwick

Newington L, *et al. BMJ Open* 2021; 11:e041656. doi: 10.1136/bmjopen-2020-041656

SECTION A: BACKGROUND

1 What is your date of birth?

☐☐☐☐☐☐
d d m m y y

2 Are you:

Male ☐ Female ☐ Other ☐

3 Are you:

Right handed ☐ Left handed ☐ Both ☐

4 Do you routinely carry out paid work for 20 hours or longer in a given week?

Yes ☐ No ☐

If no, *thank you for your interest in our study, however, we are only looking for individuals who carry out paid work for at least 20 hours per week. You* ***do not*** *need to complete the rest of the questionnaire, but please return it using the pre-paid envelope provided.*

5 When do you expect to have your carpal tunnel surgery?
Please enter the exact date if known, or provide the approximate month and year if unsure.

☐☐☐☐☐☐
d d m m y y

6 Which hand will be operated on?
If both hands please answer Question 6.1; if one hand, please move on to Question 7.

Right ☐ Left ☐ Both ☐

6.1 If both hands, which side will be operated on first?

Right ☐ Left ☐ Both sides operated on the same day ☐ Unsure ☐

7 Do you have access to an occupational health service through your place of work?

Yes ☐ No ☐ Unsure ☐

8 Do you expect to take any time off work following your surgery?
If yes, please answer Question 8.1; if no, please move on to Question 9.

Yes ☐ No ☐ Unsure ☐

8.1 If you do expect to take time off work, how long do you expect to take?
Please complete using days, weeks or months; whichever applies.

Days ☐ Weeks ☐ Months ☐

Newington L, *et al. BMJ Open* 2021; 11:e041656. doi: 10.1136/bmjopen-2020-041656

SECTION A: BACKGROUND

9 Have you been given any information about your operation?
If yes, please answer Question 9.1; if no, please move on to Question 10.

Yes ☐ No ☐

9.1 If yes, who provided this information? Please tick all that apply.

a) Your surgeon or a member of the surgical team ☐
b) Hospital nurse ☐
c) GP or practice nurse ☐
d) Hand therapist ☐
e) Physiotherapist or occupational therapist ☐
f) Occupational health nurse or doctor ☐
g) Employer ☐
h) Friend or family member ☐
i) Internet ☐
j) Other *(please specify)* ☐

---

10 Have you been given any information about returning to work after your surgery?
If yes, please answer the rest of Question 10; if no, please move on to Question 11.

Yes ☐ No ☐

10.1 If yes, who provided this information? Please tick all that apply.

a) Your surgeon or a member of the surgical team ☐
b) Hospital nurse ☐
c) GP or practice nurse ☐
d) Hand therapist ☐
e) Physiotherapist or occupational therapist ☐
f) Occupational health nurse or doctor ☐
g) Employer ☐
h) Friend or family member ☐
i) Internet ☐
j) Other *(please specify)* ☐

10.2 What advice were you given?
If this advice came from more than one source, please indicate who advised what.

SECTION B: WORK

12 What is your MAIN occupation at the moment (e.g. secretary, teacher, builder etc.)?

----

13 And in what industry do you work (e.g. farming, shipyard, car factory, shoe shop, hospital, insurance office etc)?

----

14 Which of the following best describes your present work situation for your MAIN occupation? Please tick one box.

a) Employed (permanent contract) ☐

b) Employed (temporary/renewable contract) ☐

c) Zero hours contract ☐

d) Self-employed ☐

e) Other *(please specify)* ☐

----

15 On average, how many *hours* per week do you normally work in your main occupation?

☐☐☐ hours

16 On average, how many *days* per week do you normally work in your main occupation?

☐ days

17 Do you have any other paid work?
If yes, please answer Question 17.1; if no, please move on to Question 18.

Yes ☐ No ☐

17.1 If yes, on average, how many hours a week do you work in other paid jobs? ☐☐ hours

18 Does an average day at work in your MAIN job normally involve any of the following? Please tick one box for each question.

		Yes	No
a)	Piecework in which you are paid according to the number of articles or tasks you or your team make or finish in the day?	☐	☐
b)	A target number of articles or tasks that you or your team are expected to make or finish in the day?	☐	☐
c)	Payment of a bonus if you make or finish more than an agreed number of articles/tasks in the day?	☐	☐
d)	Working to tight deadlines	☐	☐
e)	Use of a computer keyboard or mouse for longer than 1 hour in total?	☐	☐

SECTION B: WORK

		Yes	No
f)	Use of a computer keyboard or mouse for longer than 4 hours in total?	☐	☐
g)	Other tasks involving repeated movements of the wrist or fingers for longer than 4 hours in total? *(Please indicate which tasks)* ------------------------------ ------------------------------	☐	☐
h)	Working with a powered tool that makes your hand(s) or arm(s) vibrate (e.g. chain saw, pneumatic drill)?	☐	☐
i)	Working with your hand(s) above shoulder height for longer than 1 hour in total?	☐	☐
j)	Lifting or carrying weights of 5 kg (11 lbs) or more in one hand (e.g. a tool bag or heavy briefcase)?	☐	☐
k)	Lifting or carrying a weight of 10 kg (22 lbs) or more?	☐	☐
l)	Tasks involving pushing or pulling a heavy weight?	☐	☐
m)	Working for longer than two hours in total with your neck bent forward?	☐	☐
n)	Working for longer than half an hour in total with your neck twisted e.g. when looking to one side?	☐	☐
o)	Driving for more than an hour?	☐	☐

19 Do you find your MAIN job demanding on your hands/wrists?
Please circle one number, where 0 represents not at all, and 10 represents very much.

0 1 2 3 4 5 6 7 8 9 10

Not at all Very much

20 Does your MAIN employer (or boss/colleagues if self-employed) know about your hand/wrist problem?
If yes, please answer Question 20.1; if no, or not applicable, please move on to Question 21.

Yes ☐ No ☐ N/A self-employed and work alone ☐

20.1 Is your MAIN employer (or boss/colleagues if self-employed) supportive of your hand/wrist problem?
Please circle one number, where 0 represents not at all, and 10 represents very much

0 1 2 3 4 5 6 7 8 9 10

Not at all Very much

Newington L, *et al. BMJ Open* 2021; 11:e041656. doi: 10.1136/bmjopen-2020-041656

SECTION B: WORK

21 The following questions refer to how you did in your MAIN job during the *past 4 weeks*.
Please tick one box for each question.

	How much of the time during the past 4 weeks …	**Always**	**Often**	**Sometimes**	**Rarely**	**Never**
a)	Were you unable to do your work because of problems with your hand(s) / wrist(s)?	☐	☐	☐	☐	☐
b)	Did you have to shorten your work day because of problems with your hand(s) / wrists(s)	☐	☐	☐	☐	☐
c)	Did you have to take breaks at work because of problems with your hand(s) / wrists(s)?	☐	☐	☐	☐	☐
d)	Did you get less done because of problems with your hand(s) / wrist(s)?	☐	☐	☐	☐	☐
e)	Did you take longer to do the tasks in your work because of problems with your hand(s) / wrists(s)?	☐	☐	☐	☐	☐

22 During the *past 4 weeks*, how much time have you missed from your MAIN job for the following reasons?
Please write 0 if you have not missed any time from work during this period. You can answer in days or hours, whichever applies.

a) Time missed because of the problem with your hand(s)/wrist(s) ☐☐ Days *or* ☐☐ Hours

b) Time missed because of any other problem ☐☐ Days *or* ☐☐ Hours

23 If you fell ill and were off work, how long could you get your normal full pay (excluding bonuses)?
Please tick the option that best represents your MAIN job.

a) Less than one week ☐
b) 1 – 4 weeks ☐
c) 1 – 6 months ☐
d) More than 6 months ☐
e) Not sure ☐

24 How satisfied are you with your MAIN job as a whole, taking everything into consideration? This includes your salary, career possibilities, management, colleagues etc. Please tick one box.

a) Very satisfied ☐
b) Satisfied/fairly satisfied ☐
c) Dissatisfied ☐
d) Very dissatisfied ☐

Newington L, *et al. BMJ Open* 2021; 11:e041656. doi: 10.1136/bmjopen-2020-041656

SECTION C: GENERAL HEALTH

25 In general, would you say your health is:

a) Excellent ☐
b) Very good ☐
c) Good ☐
d) Fair ☐
e) Poor ☐

26 What is your height? Please answer in either feet and inches or centimetres.

☐ feet ☐☐ inches ***or*** ☐☐☐ cms

27 What is your weight? Please answer in either stones and pounds or kilograms.

☐☐ stones ☐☐ lbs ***or*** ☐☐ kgs

28 Do you, or have you ever, smoked regularly? Please tick one box.

a) I have never smoked regularly ☐
b) I have smoked in the past, but do not currently smoke regularly ☐
c) I regularly smoke ☐

29 The following is a list of common health problems. Please indicate if you currently have, or don't have, the problem listed in part 1. If you have the problem, please answer the corresponding question in part 2.
Please answer all questions in part 1.

	HEALTH PROBLEM	PART 1 Do you have the problem? NO	YES *(if yes move to part 2)* →	PART 2 Does it limit your activities? NO	YES
a)	Heart disease	☐	☐	☐	☐
b)	High blood pressure	☐	☐	☐	☐
c)	Lung disease	☐	☐	☐	☐
d)	Diabetes	☐	☐	☐	☐
e)	Ulcer or stomach disease	☐	☐	☐	☐
f)	Kidney disease	☐	☐	☐	☐
g)	Liver disease	☐	☐	☐	☐
h)	Thyroid disease	☐	☐	☐	☐
i)	Anaemia or other blood disease	☐	☐	☐	☐
j)	Cancer	☐	☐	☐	☐

Newington L, *et al. BMJ Open* 2021; 11:e041656. doi: 10.1136/bmjopen-2020-041656

SECTION C: GENERAL HEALTH

	**HEALTH PROBLEM continued…**	**Do you have the problem? NO**	**YES** *(if yes move to part 2)* →	**Does it limit your activities? NO**	**YES**
k)	Depression	☐	☐	☐	☐
l)	Osteoarthritis	☐	☐	☐	☐
m)	Back pain	☐	☐	☐	☐
n)	Rheumatoid arthritis	☐	☐	☐	☐

29.1 Please list any other medical problems that have not been mentioned.

		**Does it limit your activities? NO**	**YES**
o)	....	☐	☐
p)	....	☐	☐
q)	....	☐	☐

30 The following questions are about how you feel and how things have been with you during the *past 4 weeks*. For each question, please give the answer that comes closest to the way you have been feeling. Please tick one box for each row.

	**How much of the time during the past 4 weeks …**	**All of the time**	**Most of the time**	**A good bit of the time**	**Some of the time**	**A little bit of the time**	**None of the time**
a)	Did you feel full of 'get-up-and-go'?	☐	☐	☐	☐	☐	☐
b)	Have you been a very nervous person?	☐	☐	☐	☐	☐	☐
c)	Have you felt so down in the dumps that nothing could cheer you up?	☐	☐	☐	☐	☐	☐
d)	Have you felt calm and peaceful?	☐	☐	☐	☐	☐	☐
e)	Did you have a lot of energy?	☐	☐	☐	☐	☐	☐
f)	Have you felt downhearted and blue?	☐	☐	☐	☐	☐	☐
g)	Did you feel worn out?	☐	☐	☐	☐	☐	☐
h)	Have you been a happy person?	☐	☐	☐	☐	☐	☐
i)	Did you feel tired?	☐	☐	☐	☐	☐	☐

SECTION C: GENERAL HEALTH

31 **Below is a list of problems that people sometimes have. Please read each one carefully and tick the box that best describes how much that problem has distressed or bothered you during the *past 7 days*, including today?**
Please tick one box for each row.

		Not at all	A little bit	Moderately	Quite a bit	Extremely
a)	Faintness or dizziness	☐	☐	☐	☐	☐
b)	Pains in the heart or chest	☐	☐	☐	☐	☐
c)	Nausea or upset stomach	☐	☐	☐	☐	☐
d)	Trouble getting your breath	☐	☐	☐	☐	☐
e)	Hot or cold spells	☐	☐	☐	☐	☐

Newington L, *et al. BMJ Open* 2021; 11:e041656. doi: 10.1136/bmjopen-2020-041656

SECTION D: HAND AND WRIST FUNCTION

32 **In the *past 7 days*, have you experienced any pain, tingling (pins and needles) or numbness (loss of sensation) in your RIGHT hand or wrist?**
Please mark where on your hand/wrist you experienced these symptoms using the key below.
If you do not have any symptoms in your right hand, please move on to Question 34.

Pain | Tingling or numbness

RIGHT HAND

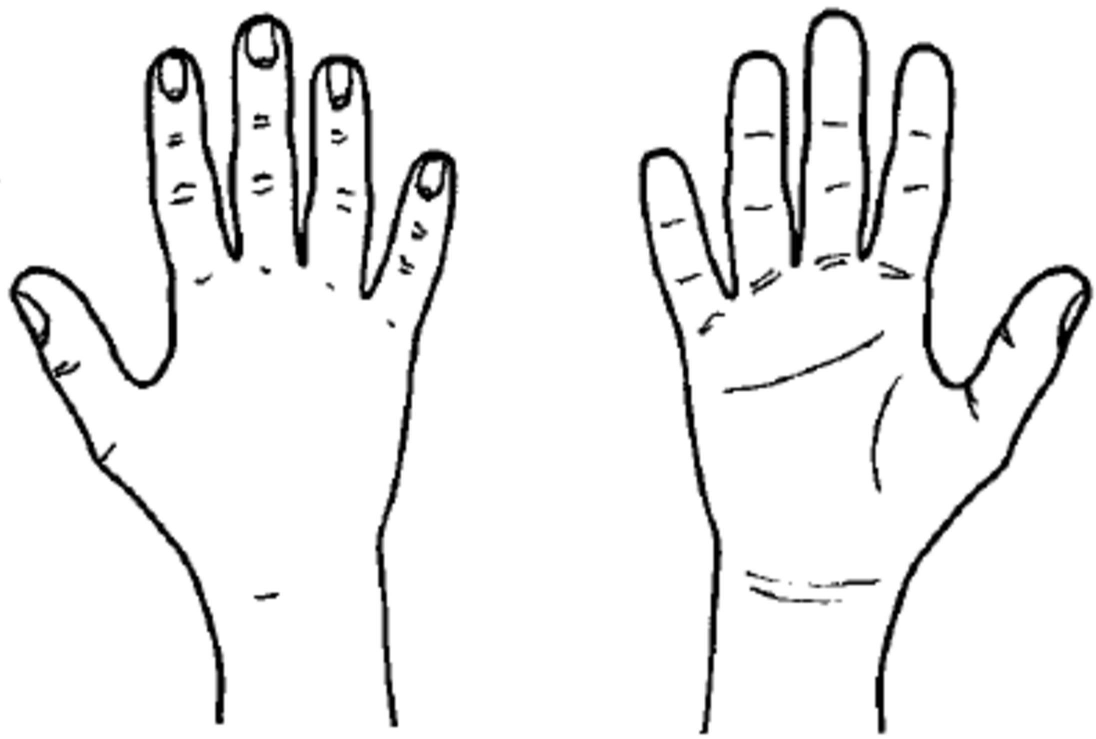

33 How long ago did the first of these symptoms begin? Please tick one box.

a) Less than 3 months ☐
b) 3 – 6 months ☐
c) 6 – 12 months ☐
d) More than a year ☐

SECTION D: HAND AND WRIST FUNCTION

34 **In the *past 7 days*, have you experienced any pain, tingling (pins and needles) or numbness (loss of sensation) in your LEFT hand or wrist?**
Please mark where on your hand/wrist you experienced these symptoms using the key below.
If you do not have any symptoms in your right hand, please move on to Question 36.

Pain | Tingling or numbness

LEFT HAND

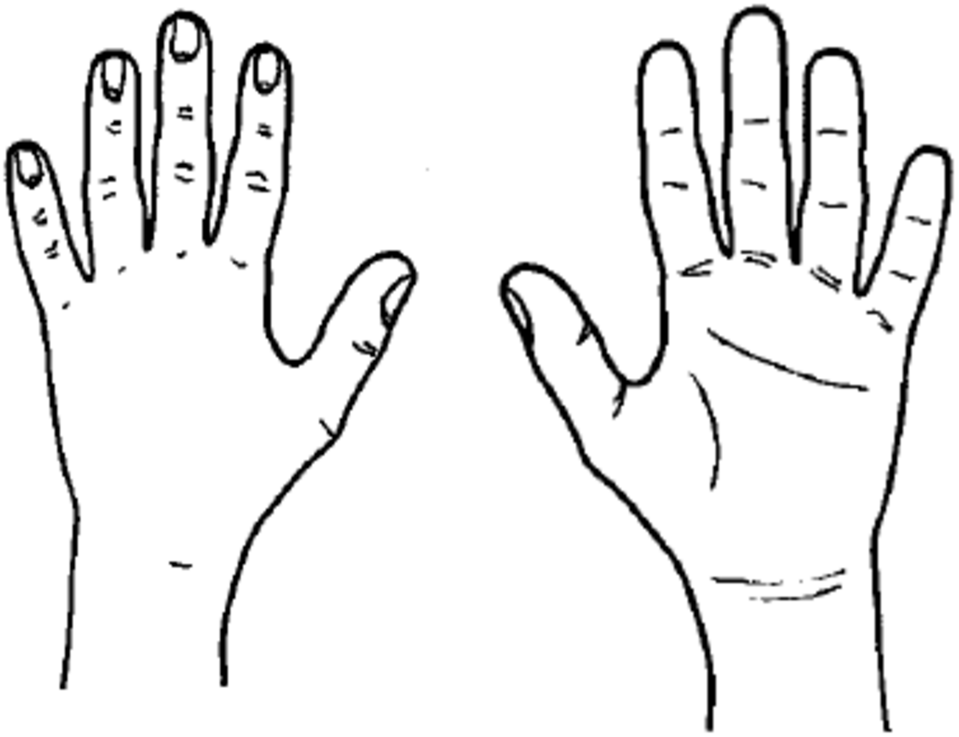

35 How long ago did the first of these symptoms begin? Please tick one box.

a) Less than 3 months ☐
b) 3 – 6 months ☐
c) 6 – 12 months ☐
d) More than a year ☐

Newington L, *et al. BMJ Open* 2021; 11:e041656. doi: 10.1136/bmjopen-2020-041656

SECTION D: HAND AND WRIST FUNCTION

36 **The following questions refer to your symptoms over the *last 7 days*.**
Please answer for each hand, even if you only have problems with one side.
Please tick one box for each row.

**36.1 How severe were the following symptoms in your RIGHT hand?**	**None**	**Mild**	**Moderate**	**Severe**	**Very severe**
a) Pain at night	☐	☐	☐	☐	☐
b) Pain during the daytime	☐	☐	☐	☐	☐
c) Numbness or tingling at night	☐	☐	☐	☐	☐
d) Numbness or tingling during the daytime	☐	☐	☐	☐	☐

**How often did the following symptoms in your RIGHT hand wake you up at night?**	**Never**	**Once**	**2 or 3 times**	**4 or 5 times**	**More than 5 times**
e) Pain	☐	☐	☐	☐	☐
f) Numbness or tingling	☐	☐	☐	☐	☐

**36.2 How severe were the following symptoms in your LEFT hand?**	**None**	**Mild**	**Moderate**	**Severe**	**Very severe**
a) Pain at night	☐	☐	☐	☐	☐
b) Pain during the daytime	☐	☐	☐	☐	☐
c) Numbness or tingling at night	☐	☐	☐	☐	☐
d) Numbness or tingling during the daytime	☐	☐	☐	☐	☐

**How often did the following symptoms in your LEFT hand wake you up at night?**	**Never**	**Once**	**2 or 3 times**	**4 or 5 times**	**More than 5 times**
e) Pain	☐	☐	☐	☐	☐
f) Numbness or tingling	☐	☐	☐	☐	☐

37 **This question refers to the appearance (look) of your hand during the *past 7 days*.** Please tick one box for each hand.

	**Strongly agree**	**Agree**	**Neither agree nor disagree**	**Disagree**	**Strongly disagree**
a) I am satisfied with the appearance (look) of my **RIGHT** hand	☐	☐	☐	☐	☐
b) I am satisfied with the appearance (look) of my **LEFT** hand	☐	☐	☐	☐	☐

Newington L, *et al. BMJ Open* 2021; 11:e041656. doi: 10.1136/bmjopen-2020-041656

SECTION D: HAND AND WRIST FUNCTION

38 Please answer the following questions on a scale of 0-10, where 0 represents not at all, and 10 represents very much. Please circle one number for each question.

38.1 Do you think that you will be able to use your hand normally 3 months after the operation?

0 1 2 3 4 5 6 7 8 9 10

38.2 Are you afraid of having long-term problems with your hand?

0 1 2 3 4 5 6 7 8 9 10

38.3 Do you blame yourself for your hand problem?

0 1 2 3 4 5 6 7 8 9 10

38.4 Are your family and friends supportive of your hand problem?

0 1 2 3 4 5 6 7 8 9 10

39 The following statements describe people's beliefs about their health problems. Please indicate whether you agree or disagree with them in relation to the problems you have with your hand(s) or wrist(s).
Please tick the box which most closely reflects how you feel for each statement.

		Strongly agree	Agree	Neither agree or disagree	Disagree	Strongly Disagree
a)	Problems like this run in my family	☐	☐	☐	☐	☐
b)	I think I was born with a weakness or underlying problem in this part of my body	☐	☐	☐	☐	☐
c)	My problem was caused by work	☐	☐	☐	☐	☐
d)	Work probably didn't cause my problem, but it made it worse	☐	☐	☐	☐	☐
e)	I have a lot of stress in my life and that has made my problem a lot worse	☐	☐	☐	☐	☐
f)	I think a lack of exercise probably contributed to my problem	☐	☐	☐	☐	☐
g)	As you get older, parts of the body start to wear out and problems like mine are likely to occur	☐	☐	☐	☐	☐

Newington L, *et al. BMJ Open* 2021; 11:e041656. doi: 10.1136/bmjopen-2020-041656

SECTION D: HAND AND WRIST FUNCTION

40 We are interested in the types of thoughts and feelings that you have when you are in pain. The following statements describe different thoughts and feelings that may be associated with pain. Please indicate the degree to which you have these thoughts and feelings when you are experiencing pain.
Please tick one box for each statement.

	Not at all	To a slight degree	To a moderate degree	To a great degree	All of the time
a) I keep thinking about how badly I want the pain to stop	☐	☐	☐	☐	☐
b) It's terrible and I think it's never going to get any better	☐	☐	☐	☐	☐
c) I become afraid that the pain may get worse	☐	☐	☐	☐	☐
d) I anxiously want the pain to go away	☐	☐	☐	☐	☐

The following questions refer to the function of your hands/wrists during the ***past 7 days***. Please answer all questions for the right and left sides, even if you do not experience any problems. Please tick one box for each question.

**41 RIGHT SIDE**	**Very well**	**Well**	**Adequately**	**Poorly**	**Very poorly**
a) Overall, how well did your ***right*** hand work?	☐	☐	☐	☐	☐
b) How well did your ***right*** fingers move?	☐	☐	☐	☐	☐
c) How well did your ***right*** wrist move?	☐	☐	☐	☐	☐
	**Very good**	**Good**	**Fair**	**Poor**	**Very poor**
d) How was the strength in your ***right*** hand?	☐	☐	☐	☐	☐
e) How was the sensation (feeling) in your ***right*** hand?	☐	☐	☐	☐	☐

**42 LEFT SIDE**	**Very well**	**Well**	**Adequately**	**Poorly**	**Very poorly**
a) Overall, how well did your ***left*** hand work?	☐	☐	☐	☐	☐
b) How well did your ***left*** fingers move?	☐	☐	☐	☐	☐
c) How well did your ***left*** wrist move?	☐	☐	☐	☐	☐
	**Very good**	**Good**	**Fair**	**Poor**	**Very poor**
d) How was the strength in your ***left*** hand?	☐	☐	☐	☐	☐
e) How was the sensation (feeling) in your ***left*** hand?	☐	☐	☐	☐	☐

Newington L, *et al. BMJ Open* 2021; 11:e041656. doi: 10.1136/bmjopen-2020-041656

SECTION D: HAND AND WRIST FUNCTION

The following questions refer to the ability of your hands to do certain tasks during the ***past 7 days***. If you do not do a certain task, please estimate the difficulty you would have in performing it. Please tick one box for every activity.

43 How difficult was it for you to perform the following activities using your RIGHT HAND?

		Not at all difficult	A little difficult	Somewhat difficult	Moderately difficult	Very difficult
a)	Turn a door knob	☐	☐	☐	☐	☐
b)	Pick up a coin	☐	☐	☐	☐	☐
c)	Hold a glass of water	☐	☐	☐	☐	☐
d)	Turn a key in a lock	☐	☐	☐	☐	☐
e)	Hold a frying pan	☐	☐	☐	☐	☐

44 How difficult was it for you to perform the following activities using your LEFT HAND?

		Not at all difficult	A little difficult	Somewhat difficult	Moderately difficult	Very difficult
a)	Turn a door knob	☐	☐	☐	☐	☐
b)	Pick up a coin	☐	☐	☐	☐	☐
c)	Hold a glass of water	☐	☐	☐	☐	☐
d)	Turn a key in a lock	☐	☐	☐	☐	☐
e)	Hold a frying pan	☐	☐	☐	☐	☐

45 How difficult was it for you to perform the following activities using BOTH HANDS?

		Not at all difficult	A little difficult	Somewhat difficult	Moderately difficult	Very difficult
a)	Open a jar	☐	☐	☐	☐	☐
b)	Button a shirt/blouse	☐	☐	☐	☐	☐
c)	Eat with a knife/fork	☐	☐	☐	☐	☐
d)	Carry a grocery bag	☐	☐	☐	☐	☐
e)	Wash dishes	☐	☐	☐	☐	☐
f)	Wash your hair	☐	☐	☐	☐	☐
g)	Tie shoelaces/knots	☐	☐	☐	☐	☐

Newington L, *et al. BMJ Open* 2021; 11:e041656. doi: 10.1136/bmjopen-2020-041656

SECTION D: HAND AND WRIST FUNCTION

The following questions refer to your satisfaction with your hands/wrists during the ***past 7 days***. Please tick one box for each question

46 How satisfied were you with your RIGHT hand/wrist during the *past 7 days*?

	RIGHT HAND	Very satisfied	Somewhat satisfied	Neither satisfied or dissatisfied	Somewhat dissatisfied	Very dissatisfied
a)	Overall function of your hand	☐	☐	☐	☐	☐
b)	Movement of the fingers	☐	☐	☐	☐	☐
c)	Movement of your wrist	☐	☐	☐	☐	☐
d)	Strength of your hand	☐	☐	☐	☐	☐
e)	Pain level of your hand	☐	☐	☐	☐	☐
f)	Sensation (feeling) of your hand	☐	☐	☐	☐	☐

47 How satisfied were you with your LEFT hand/wrist during the *past 7 days*?

	LEFT HAND	Very satisfied	Somewhat satisfied	Neither satisfied or dissatisfied	Somewhat dissatisfied	Very dissatisfied
a)	Overall function of your hand	☐	☐	☐	☐	☐
b)	Movement of the fingers	☐	☐	☐	☐	☐
c)	Movement of your wrist	☐	☐	☐	☐	☐
d)	Strength of your hand	☐	☐	☐	☐	☐
e)	Pain level of your hand	☐	☐	☐	☐	☐
f)	Sensation (feeling) of your hand	☐	☐	☐	☐	☐

Thank you for completing this questionnaire!
Please return it to the REACTS team
using the pre-paid envelope.

REACTS

If you have any questions, or would like any additional information, please contact Lisa Newington on:
ln@mrc.soton.ac.uk | 023 8077 7624 | 07866 997732

Newington L, *et al. BMJ Open* 2021; 11:e041656. doi: 10.1136/bmjopen-2020-041656
