# Supplementary material for: Sickness absence after carpal tunnel release: a multicentre prospective cohort study
Source: BMJ Open. 2021 Feb 10;11(2):e041656. doi: 10.1136/bmjopen-2020-041656 (PMC7878133; doi:10.1136/bmjopen-2020-041656)
Supplement: Supplementary data [file bmjopen-2020-041656supp003.pdf]

REACTS ID: | | | | | | |

One Month After Your Carpal Tunnel Release Surgery

Return to Employment After Carpal Tunnel Release Surgery (REACTS)

ln@mrc.soton.ac.uk | 023 8077 7624
Arthritis Research UK – MRC Centre for Musculoskeletal Health and Work
MRC Lifecourse Epidemiology Unit, University of Southampton
Southampton General Hosptial (MP 95), SO16 6YD

IRAS reference: 209840

SECTION A: ABOUT YOUR OPERATION

Please fill in today's date ☐☐☐☐☐☐☐☐
d d m m y y y y

1 What was the date of your carpal tunnel release surgery? ☐☐☐☐☐☐☐☐
d d m m y y y y

2 Which side was operated on? Please tick one box.

Right ☐ Left ☐ Both ☐

3 What type of anaesthetic did you have? Please tick one box.

a) General anaesthetic (you were sent to sleep) ☐

b) Local or regional anaesthetic (your arm was made numb, but you were still awake) ☐

c) Other *(please specify)* ☐

----

d) Unsure ☐

4 How long did you need to stay in the hospital/clinic after your operation?
Please tick one box (and specify the number of nights, if applicable).

a) I went home the same day ☐

b) I needed to stay overnight (one night only) ☐

c) I needed to stay for more than one night ☐

(Please specify for how long) ☐☐ nights

SECTION A: ABOUT YOUR OPERATION

5 Have you used any of the following services specifically for your operated hand(s) since your surgery?
Please give the number of visits for each service, and the date(s) attended, if known.

	**I used this service in the NHS**		**I used this service privately**	
	Number of visits	Dates attended, if known	Number of visits	Dates attended, if known
a) Your surgeon, or one of the surgical team				
b) GP or practice nurse				
c) Hospital nurse				
d) Pharmacist				
e) Hand therapist				
f) Other physiotherapist or occupational therapist				
g) Chiropractor or osteopath				
h) Occupational health nurse or doctor				
i) Accident and emergency (A&E) or minor injuries unit				
j) Other (*please specify)*				

SECTION A: ABOUT YOUR OPERATION

6 Have you taken any antibiotics for an infection in your surgical wound?
Please do not include any antibiotics you were prescribed at the time of your operation.

Yes ☐ No ☐

If yes, what date did you start taking the antibiotics? ☐☐☐☐☐☐☐☐
d d m m y y y y

7 Have you been admitted to hospital because of a problem with your operated hand(s)? If yes, please answer the rest of Question 7; if no, please move on to Question 8.

Yes ☐ No ☐

7.1 If yes, when were you first admitted? ☐☐☐☐☐☐☐☐
d d m m y y y y

7.2 How many nights did you stay in hospital?
Please answer 0 if you didn't stay overnight.
☐☐ nights

7.3 Did you require another operation? Yes ☐ No ☐

8 Have you been advised that you may need a carpal tunnel release for your other hand in the future?
If yes, please answer Question 8.1; if no, please move on to Question 9.

Yes ☐ No ☐

8.1 If yes, when are you expecting to have this surgery? Please tick one box.

a) In less than 2 months ☐
b) In 2-5 months ☐
c) In 6-11 months ☐
d) In more than a year ☐
e) Unsure ☐

9 If you would like to give us any other information about your operation, or the healthcare services you have used, please do so here:

SECTION B: WORK

10 Compared to before your surgery, which of the following best describes your current work situation? Please tick one box.

a) Returned to the same job, work duties and hours – **please go to Question 14** ☐

b) Returned to the same job, with altered duties or hours – **please go to Question 14** ☐

c) Started a new job – **please go to Question 11** ☐

d) Not yet returned to work, but plan to return in the future – **please go to Question 12** ☐

e) Do not plan to return to work – **please go to Question 13** ☐

11 Thinking about your new job:

11.1 What is your main occupation now (e.g. secretary, teacher, builder etc.)?

11.2 In what industry do you work (e.g. farming, shipyard, car factory, shoe shop, hospital, insurance office etc.)?

11.3 Did you change jobs because of your hand/wrist problem? Please tick one box.

a) Yes, my hand/wrist problem was the main reason for my job change ☐

b) Yes, my hand/wrist problem was one of several reasons for my job change ☐

c) No, my job change was nothing to do with my hand/wrist problem ☐

d) Other, please specify ☐

Please go to Question 14

Newington L, *et al. BMJ Open* 2021; 11:e041656. doi: 10.1136/bmjopen-2020-041656

SECTION B: WORK

12 If you have not yet returned to work, when do you think you might be able to return? Please give an estimated date if you are unsure.

☐☐☐☐☐☐☐☐
d d m m y y y y

12.1 Have you discussed when to return to work with anyone?
If yes, please answer the rest of Question 12; if no, please move on to Question 21.

Yes ☐ No ☐

12.2 If yes, who have you discussed this with? Please tick all that apply.

a) Your surgeon or a member of the surgical team ☐
b) Hospital nurse ☐
c) GP or practice nurse ☐
d) Hand therapist ☐
e) Physiotherapist or occupational therapist ☐
f) Occupational health nurse or doctor ☐
g) Employer or manager (or colleagues if self-employed ☐
h) Friend or family member ☐
i) Other *(please specify)* ☐

12.3 Since your operation, have you been given any specific advice about when and how to return to work? This could include any activities to avoid or timescales to follow. Please list any advice here, including who gave you this advice:

Please go to Question 21

SECTION B: WORK

13 If you do not plan to return to work, what is the main reason for this decision?
Please tick one box.

a) Retirement ☐
b) Redundancy ☐
c) Position/work no longer available ☐
d) Unable to do your work because of your problem with your hand(s)/wrist(s) ☐
e) Unable to do your work because of any other problem ☐
f) Other *(please specify)* ☐

13.1 Have you been advised not to return to work by anyone?
If yes, please answer Question 13.2; if no, please move on to Question 21.

Yes ☐ No ☐

13.2 If yes, who by? Please tick all that apply.

a) Your surgeon or a member of the surgical team ☐
b) Hospital nurse ☐
c) GP or practice nurse ☐
d) Hand therapist ☐
e) Physiotherapist or occupational therapist ☐
f) Occupational health nurse or doctor ☐
g) Employer or manager (or colleagues if self-employed) ☐
h) Friend or family member ☐
i) Other *(please specify)* ☐

Please go to Question 21

Newington L, *et al. BMJ Open* 2021; 11:e041656. doi: 10.1136/bmjopen-2020-041656

SECTION B: WORK

14 When did you first return to work after your carpal tunnel release surgery?

☐☐☐☐☐☐☐☐
d d m m y y y y

15 How much work-time did you miss between the date of your surgery and the date you first returned to work?
Please include all work-time missed, even if this had been pre-arranged with your employer, or was taken as annual leave. You can answer in hours, days or weeks, whichever applies.

☐☐☐ hours ☐☐ days ☐☐ weeks

15.1 Was any of this time paid?
Please tick one box (and provide the amount of time, if applicable).

a) Yes, all of my time away from work was paid ☐

b) Yes, some of my time away from work was paid ☐
(please specify how much time was paid, you can use hours, days or weeks, whichever applies)

☐☐☐ hours ☐☐ days ☐☐ weeks

c) No, none of my time off was paid ☐

d) Not sure ☐

16 Since your surgery, have you discussed when to return to work with anyone?
If yes, please answer the rest of Question 16; if no, please move on to Question 17.

Yes ☐ No ☐

16.1 If yes, who did you discuss this with? Please tick all that apply.

a) Your surgeon or a member of the surgical team ☐

b) Hospital nurse ☐

c) GP or practice nurse ☐

d) Hand therapist ☐

e) Physiotherapist or occupational therapist ☐

f) Occupational health nurse or doctor ☐

g) Employer or manager (or colleagues if self-employed ☐

h) Friend or family member ☐

i) Other *(please specify)* ☐

Newington L, *et al. BMJ Open* 2021; 11:e041656. doi: 10.1136/bmjopen-2020-041656

SECTION B: WORK

16.2 Please list any advice you have been given (since your surgery) about when and how to return to work?
This could include any activities to avoid or timescales to follow. If this advice came from more than one place, please indicate who advised what.

----

17 Since returning to work after your operation, have you needed to take any time off work because of a problem with your operated hand(s)/wrist(s)?
If yes, please answer Question 17.1; if no, please move on to Question 18.

Yes ☐ No ☐

17.1 If yes, how much time did you take off work?
Please answer in days or hours, whichever applies.

☐☐☐ hours ☐☐ days ☐☐ weeks

18 When you first returned to work after your surgery, did you work shorter hours than would be normal for your job as a direct result of your operation?
If yes, please answer the rest of Question 18; if no, please move on to Question 19.

Yes ☐ No ☐

18.1 Have you since gone back to working full hours?
If yes, please answer Question 18.2; if no, please move on to Question 19.

Yes ☐ No ☐

18.2 If yes, when did you return to full working hours?
If you do not know the exact date, approximately how many weeks did you work reduced hours?

☐☐☐☐☐☐☐☐
d d m m y y y y

a) Less than a week ☐
b) 1 – 2 weeks ☐
c) More than 2 weeks, but less than 3 weeks ☐
d) 3 weeks or longer ☐

Newington L, *et al. BMJ Open* 2021; 11:e041656. doi: 10.1136/bmjopen-2020-041656

SECTION B: WORK

19 When you first returned to work after your surgery, did you need to alter or avoid any of your usual work duties as a direct result of your operation?
If yes, please answer the rest of Question 19; if no, please move on to Question 20.

Yes ☐ No ☐

19.1 Have you since gone back to full duties?
If yes, please answer Question 19.2; if no, please move on to Question 20.

Yes ☐ No ☐

19.2 If yes, when did you return to full working duties?
If you do not know the exact date, approximately how many weeks did you have altered work duties?

d	d	m	m	y	y	y	y

a) Less than a week ☐

b) 1 – 2 weeks ☐

c) More than 2 weeks, but less than 3 weeks ☐

d) 3 weeks or longer ☐

20 If you would like to give us any additional information about returning to work, please do so here:

SECTION C: HAND AND WRIST SYMPTOMS

21 The following questions refer to your symptoms over the *last 7 days*.
Please answer for each hand. Please tick one box for each row.

**21.1 How severe were the following symptoms in your RIGHT hand?**	None	Mild	Moderate	Severe	Very severe
a) Pain at night	☐	☐	☐	☐	☐
b) Pain during the daytime	☐	☐	☐	☐	☐
c) Numbness or tingling at night	☐	☐	☐	☐	☐
d) Numbness or tingling during the daytime	☐	☐	☐	☐	☐

**How often did the following symptoms in your RIGHT hand wake you up at night?**	Never	Once	2 or 3 times	4 or 5 times	More than 5 times
e) Pain	☐	☐	☐	☐	☐
f) Numbness or tingling	☐	☐	☐	☐	☐

**21.2 How severe were the following symptoms in your LEFT hand?**	None	Mild	Moderate	Severe	Very severe
a) Pain at night	☐	☐	☐	☐	☐
b) Pain during the daytime	☐	☐	☐	☐	☐
c) Numbness or tingling at night	☐	☐	☐	☐	☐
d) Numbness or tingling during the daytime	☐	☐	☐	☐	☐

**How often did the following symptoms in your LEFT hand wake you up at night?**	Never	Once	2 or 3 times	4 or 5 times	More than 5 times
e) Pain	☐	☐	☐	☐	☐
f) Numbness or tingling	☐	☐	☐	☐	☐

22 This question refers to the appearance (look) of your hands during the *past 7 days*. Please tick one box for each hand.

	Strongly agree	Agree	Neither agree nor disagree	Disagree	Strongly disagree
a) I am satisfied with the appearance (look) of my **RIGHT** hand	☐	☐	☐	☐	☐
b) I am satisfied with the appearance (look) of my **LEFT** hand	☐	☐	☐	☐	☐

Newington L, *et al. BMJ Open* 2021; 11:e041656. doi: 10.1136/bmjopen-2020-041656

SECTION C: HAND AND WRIST SYMPTOMS

23 How do you rate your symptoms in your operated hand(s) now, compared to before your surgery? Please tick one box.

a) Completely cured ☐ c) Unchanged ☐ e) Worse ☐

b) Much better ☐ d) Slightly better ☐

24 The following questions ask specifically about your scar. Please think about your scar over the ***past 7 days.***

24.1 Has your scar been itchy?
If yes, please continue; if no, please move on to Question 24.2

Yes ☐ No ☐

Yes, it was itchy:	Sometimes ☐	Often ☐	Always ☐
And when it was itchy, it was:	Slightly itchy ☐	Fairly itchy ☐	Very itchy ☐

24.2 Has your scar caused you pain?
If yes, please continue; if no, please move on to Question 24.3

Yes ☐ No ☐

Yes, it was painful:	Sometimes ☐	Often ☐	Always ☐
And when it hurt, it was:	Slightly painful ☐	Fairly painful ☐	Very painful ☐

24.3 Has your scar been uncomfortable?
If yes, please continue; if no, please move on to Question 24.4

Yes ☐ No ☐

Yes, it was uncomfortable:	Sometimes ☐	Often ☐	Always ☐
And when it was uncomfortable, it was:	Slightly uncomfortable ☐	Fairly uncomfortable ☐	Very uncomfortable ☐

SECTION C: HAND AND WRIST SYMPTOMS

24.4 Has your scar felt numb?
If yes, please continue; if no, please move on to Question 24.5

Yes ☐ No ☐

Yes, it was numb:	Sometimes ☐	Often ☐	Always ☐
And when it felt numb, it was:	Slightly numb ☐	Fairly numb ☐	Very numb ☐

24.5 Have you had odd sensations in your scar e.g. tightening, pulling or pins and needles? If yes, please continue; if no, please move on to Question 24.6

Yes ☐ No ☐

Yes, I have had odd sensations:	Sometimes ☐	Often ☐	Always ☐

24.6 Has your scar caught on things e.g. clothing?
If yes, please continue; if no, please move on to Question 24.7

Yes ☐ No ☐

Yes, it has caught on things:	Sometimes ☐	Often ☐	Always ☐

24.7 Overall, how troublesome are the symptoms from your scar?
Please tick one box.

Not at all troublesome	A little troublesome	Fairly troublesome	Very troublesome	Unbearable
☐	☐	☐	☐	☐

SECTION D: HAND AND WRIST FUNCTION

The following questions refer to the function of your hands/wrists during the ***past 7 days***. Please answer all questions for the right and left sides, even if you do not experience any problems. Please tick one box for each question.

**25 RIGHT SIDE**	**Very well**	**Well**	**Adequately**	**Poorly**	**Very poorly**
a) Overall, how well did your ***right*** hand work?	☐	☐	☐	☐	☐
b) How well did your ***right*** fingers move?	☐	☐	☐	☐	☐
c) How well did your ***right*** wrist move?	☐	☐	☐	☐	☐
	**Very good**	**Good**	**Fair**	**Poor**	**Very poor**
d) How was the strength in your ***right*** hand?	☐	☐	☐	☐	☐
e) How was the sensation (feeling) in your ***right*** hand?	☐	☐	☐	☐	☐

**26 LEFT SIDE**	**Very well**	**Well**	**Adequately**	**Poorly**	**Very poorly**
a) Overall, how well did your ***left*** hand work?	☐	☐	☐	☐	☐
b) How well did your ***left*** fingers move?	☐	☐	☐	☐	☐
c) How well did your ***left*** wrist move?	☐	☐	☐	☐	☐
	**Very good**	**Good**	**Fair**	**Poor**	**Very poor**
d) How was the strength in your ***left*** hand?	☐	☐	☐	☐	☐
e) How was the sensation (feeling) in your ***left*** hand?	☐	☐	☐	☐	☐

The following questions refer to the ability of your hands to do certain tasks during the ***past 7 days***. If you do not do a certain task, please estimate the difficulty you would have in performing it. Please tick one box for every activity.

27 How difficult was it for you to perform the following activities using your RIGHT HAND?

	**Not at all difficult**	**A little difficult**	**Somewhat difficult**	**Moderately difficult**	**Very difficult**
a) Turn a door knob	☐	☐	☐	☐	☐
b) Pick up a coin	☐	☐	☐	☐	☐
c) Hold a glass of water	☐	☐	☐	☐	☐
d) Turn a key in a lock	☐	☐	☐	☐	☐
e) Hold a frying pan	☐	☐	☐	☐	☐

SECTION D: HAND AND WRIST FUNCTION

28 How difficult was it for you to perform the following activities using your LEFT HAND?

		Not at all difficult	A little difficult	Somewhat difficult	Moderately difficult	Very difficult
a)	Turn a door knob	☐	☐	☐	☐	☐
b)	Pick up a coin	☐	☐	☐	☐	☐
c)	Hold a glass of water	☐	☐	☐	☐	☐
d)	Turn a key in a lock	☐	☐	☐	☐	☐
e)	Hold a frying pan	☐	☐	☐	☐	☐

29 How difficult was it for you to perform the following activities using BOTH HANDS?

		Not at all difficult	A little difficult	Somewhat difficult	Moderately difficult	Very difficult
a)	Open a jar	☐	☐	☐	☐	☐
b)	Button a shirt/blouse	☐	☐	☐	☐	☐
c)	Eat with a knife/fork	☐	☐	☐	☐	☐
d)	Carry a grocery bag	☐	☐	☐	☐	☐
e)	Wash dishes	☐	☐	☐	☐	☐
f)	Wash your hair	☐	☐	☐	☐	☐
g)	Tie shoelaces/knots	☐	☐	☐	☐	☐

The following questions refer to your satisfaction with your hands/wrists during the ***past 7 days***. Please tick one box for each question.

30 How satisfied were you with your RIGHT hand/wrist during the *past 7 days*?

	RIGHT HAND	Very satisfied	Somewhat satisfied	Neither satisfied or dissatisfied	Somewhat dissatisfied	Very dissatisfied
a)	Overall function of your hand	☐	☐	☐	☐	☐
b)	Movement of the fingers	☐	☐	☐	☐	☐
c)	Movement of your wrist	☐	☐	☐	☐	☐
d)	Strength of your hand	☐	☐	☐	☐	☐
e)	Pain level of your hand	☐	☐	☐	☐	☐
f)	Sensation (feeling) of your hand	☐	☐	☐	☐	☐

Newington L, *et al. BMJ Open* 2021; 11:e041656. doi: 10.1136/bmjopen-2020-041656

SECTION D: HAND AND WRIST FUNCTION

31 How satisfied were you with your LEFT hand/wrist during the *past 7 days*?

	LEFT HAND	Very satisfied	Somewhat satisfied	Neither satisfied or dissatisfied	Somewhat dissatisfied	Very dissatisfied
a)	Overall function of your hand	☐	☐	☐	☐	☐
b)	Movement of the fingers	☐	☐	☐	☐	☐
c)	Movement of your wrist	☐	☐	☐	☐	☐
d)	Strength of your hand	☐	☐	☐	☐	☐
e)	Pain level of your hand	☐	☐	☐	☐	☐
f)	Sensation (feeling) of your hand	☐	☐	☐	☐	☐

32 If you would like to give us any additional information about your hand and wrist function, please do so here:

Thank you for completing this questionnaire!
Please return it to the REACTS team
using the pre-paid envelope.

REACTS

If you have any questions or would like any additional information, please contact Lisa Newington on:
ln@mrc.soton.ac.uk | 023 8077 7624 | 07866 997732
