# Supplementary material for: Sickness absence after carpal tunnel release: a multicentre prospective cohort study
Source: BMJ Open. 2021 Feb 10;11(2):e041656. doi: 10.1136/bmjopen-2020-041656 (PMC7878133; doi:10.1136/bmjopen-2020-041656)
Supplement: Supplementary data [file bmjopen-2020-041656supp004.pdf]

## SUPPLEMENTARY TABLES

### 1. Cox proportional hazards univariate analyses of the association between demographic and general health factors and the duration of work absence after carpal tunnel release (non-significant findings)

| | N | Median time to return to work (days) | Inter quartile range | Hazards ratio [1] | 95% Confidence intervals | P value |
|---|---|---|---|---|---|---|
| *Body mass index (kg/m²) (censored 1 normal, 1 overweight, 2 obese; 13 missing)* | | | | | | |
| Normal (18.5-24.9) | 47 | 20 | 12-33 | 1 | - | - |
| Over (25.0-29.9) | 64 | 19 | 12.5-29 | 0.98 | 0.65, 1.46 | 0.91 |
| Obese (≥30) | 67 | 21 | 10-40 | 0.88 | 0.60, 1.28 | 0.50 |
| *Surgery to dominant hand [2] (censored 3 yes, 2 no; no missing data)* | | | | | | |
| Yes | 128 | 19.5 | 12-33 | 1 | - | - |
| No | 62 | 22 | 12-35 | 0.83 | 0.61, 1.14 | 0.25 |
| *General health status (censored 5 excellent; 1 missing)* | | | | | | |
| Excellent/very good/ good | 165 | 19 | 12-32 | 1 | - | - |
| Fair/poor | 24 | 28.5 | 12.5-42 | 0.74 | 0.47, 1.16 | 0.19 |
| *Number of comorbidities [3] (censored 3 none, 1 one; 1 missing)* | | | | | | |
| None | 49 | 18 | 7-32 | 1 | - | - |
| One | 67 | 24 | 14-42 | 0.87 | 0.59, 1.27 | 0.47 |
| Two or more | 74 | 19.5 | 10-29 | 1.11 | 0.76, 1.62 | 0.60 |
| *Number of disability comorbidities [3] (censored 3 none, 2 one; no missing data)* | | | | | | |
| None | 130 | 19 | 9-33 | 1 | - | - |
| One | 39 | 26 | 13-31 | 0.75 | 0.52, 1.08 | 0.12 |
| Two or more | 21 | 20 | 14-31 | 0.81 | 0.50, 1.32 | 0.40 |
| *Number of somatising symptoms [4] (censored 1 none, 1 one, 3 two or more; 1 missing)* | | | | | | |
| None | 96 | 19 | 10.5-35 | 1 | - | - |
| One | 51 | 22 | 13-34 | 0.98 | 0.70, 1.38 | 0.91 |
| Two or more | 42 | 19.5 | 14-31 | 0.84 | 0.58, 1.22 | 0.36 |
| *SF36 Mental health score (tertiles) [5] (censored 2 poor, 1 intermediate, 2 good; 2 missing)* | | | | | | |
| Poor (22.2-57.8) | 60 | 24 | 16-39 | 0.71 | 0.50, 1.03 | 0.07 |
| Intermediate (60.0-75.6) | 68 | 18.5 | 11-36.5 | 0.88 | 0.62, 1.25 | 0.48 |
| Good (77.8-97.8) | 60 | 16.5 | 7-30 | 1 | - | - |

Total sample size n=195. Median duration of work absence relates to the 190 non-censored events.

1. Adjusted for age and sex.
2. Considered and the non-dominant hand for those who reported ambidexterity (n=4) and dominant hand for those undergoing simultaneous bilateral surgery (n=2).
3. Adapted from the self-administered co-morbidity questionnaire [1].
4. Adapted from the four-dimensional symptom questionnaire [2].
5. Taken from the SF-36 with data-driven tertiles [3].

## 2. Cox proportional hazards univariate analyses of the association between health beliefs and the duration of work absence after carpal tunnel release (non-significant findings)

| | N | Median time to return to work (days) | Inter quartile range | Hazards ratio [1] | 95% Confidence intervals | P value |
|---|---|---|---|---|---|---|
| *Believe will be unable to use hand normally in 3 months [2] (censored 5 no; 2 missing)* | | | | | | |
| No | 168 | 20 | 12-32.5 | 1 | - | - |
| Yes | 20 | 20.5 | 14.5-40 | 1.04 | 0.65, 1.67 | 0.87 |
| *Blames self for hand problem [2] (censored 5 no; 2 missing)* | | | | | | |
| No | 162 | 20 | 12-35 | 1 | - | - |
| Yes | 26 | 22 | 14-31 | 1.08 | 0.70, 1.66 | 0.73 |
| *Lack of support from family/friends [2] (censored 4 no, 1 yes; 2 missing)* | | | | | | |
| No | 156 | 20 | 12-32.5 | 1 | - | - |
| Yes | 32 | 19.5 | 13-40 | 0.88 | 0.60, 1.29 | 0.51 |
| *Problem runs in the family/born with a weakness [3] (censored 3 no, 2 agree/strongly agree; 2 missing)* | | | | | | |
| No | 134 | 20 | 12-35 | 1 | - | - |
| Agree/strongly agree | 54 | 23 | 12-31 | 0.88 | 0.63, 1.21 | 0.42 |
| *Stress made the problem worse [3] (censored 5 no; 2 missing)* | | | | | | |
| No | 169 | 20 | 12-33 | 1 | - | - |
| Agree/strongly agree | 19 | 28 | 14-40 | 0.79 | 0.48, 1.28 | 0.33 |
| *Lack of exercise contributed to the problem [3] (censored 5 no; 2 missing)* | | | | | | |
| No | 172 | 20 | 13-33 | 1 | - | - |
| Agree/strongly agree | 16 | 15 | 10.5-34.5 | 1.23 | 0.73, 2.06 | 0.43 |
| *Getting older contributes to the problem [3] (censored 3 no, 2 yes; 3 missing)* | | | | | | |
| No | 79 | 26 | 14-42 | 1 | - | - |
| Agree/strongly agree | 108 | 16 | 10-29 | 1.28 | 0.94, 1.74 | 0.11 |
| *Work probably didn't cause the problem, but made it worse [3] (censored 2 no, 3 yes; 4 missing)* | | | | | | |
| No | 100 | 20.5 | 12-35 | 1 | - | - |
| Agree/strongly agree | 86 | 19.5 | 13-31 | 1.01 | 0.75, 1.35 | 0.95 |
| *Pain catastrophisation to at least a moderate degree [4] (censored 3 no, 2 yes; missing 2)* | | | | | | |
| No | 133 | 19 | 10-33 | 1 | - | - |
| Yes | 55 | 22 | 15-31 | 0.81 | 0.59, 1.12 | 0.21 |

Total sample size n=195. Median duration of work absence relates to the 190 non-censored events.

1. Adjusted for age and sex.

2. Reported on a 0-10 scale and dichotomised to no (0-6) and yes (7-10) [4].

3. Reported on a 5-point scale and dichotomised to agree/strongly agree and neither agree nor disagree/disagree/disagree strongly [5].

4. Pain catastrophizing scale dichotomised to those who reported catastrophising pain thoughts and feelings to at least a moderate degree in response to all questions and those who did not [6].

**3. Cox proportional hazards univariate analyses of the association between clinical and surgical factors and the duration of work absence after carpal tunnel release (non-significant findings)**

| | N | Median time to return to work (days) | Inter quartile range | Hazards ratio [1] | 95% Confidence intervals | P value |
|---|---|---|---|---|---|---|
| *Katz hand diagram score for side of surgery [2] (censored 3 classics/probable, 2 possible/unlikely; 4 missing)* | | | | | | |
| Classic/probable | 126 | 22 | 14-38 | 1 | - | - |
| Possible/unlikely | 60 | 16.5 | 9.5-30.5 | 1.10 | 0.81, 1.51 | 0.54 |
| *Duration of symptoms (censored 1 ≤ 1 year, 4 > 1 year; 2 missing)* | | | | | | |
| ≤ 1 year | 47 | 19 | 10-31 | 1 | - | - |
| > 1 year | 141 | 20 | 13-34 | 0.86 | 0.62, 1.21 | 0.38 |
| *Nerve conduction studies performed (censored 3 no, 2 yes; 40 missing)* | | | | | | |
| No | 71 | 21 | 14-35 | 1 | - | - |
| Yes | 79 | 20 | 11-33 | 1.14 | 0.83, 1.58 | 0.41 |
| *Type of suture material (censored 4 non-absorbable; 49 missing)* | | | | | | |
| Non-absorbable | 119 | 21 | 12-38 | 1 | - | - |
| Absorbable | 23 | 20 | 13-29 | 1.15 | 0.73, 1.83 | 0.55 |
| *MHQ function score for side of surgery (tertiles) [3] (censored 3 poor, 1 intermediate, 1 good; 2 missing)* | | | | | | |
| Poor (0-40) | 79 | 20 | 13-41 | 0.80 | 0.57, 1.12 | 0.19 |
| Intermediate (44-55) | 48 | 19.5 | 13.5-33 | 0.81 | 0.55, 1.19 | 0.29 |
| Good (60-100) | 61 | 20 | 7-29 | 1 | - | - |
| *MHQ bilateral activities of daily living score (tertiles) [3] (censored 1 poor, 4 intermediate; 1 missing)* | | | | | | |
| Poor (0-61) | 72 | 19 | 12.5-33.5 | 0.96 | 0.66, 1.38 | 0.81 |
| Intermediate (64-82) | 55 | 21 | 13-38 | 0.70 | 0.49, 1.01 | 0.06 |
| Good (83-100) | 62 | 20 | 10-31 | 1 | - | - |
| *MHQ activities of daily living score, side of surgery (tertiles) [3] (censored 2 poor, 3 intermediate; 1 missing)* | | | | | | |
| Poor (0-55) | 63 | 20 | 12-42 | 0.81 | 0.56, 1.18 | 0.27 |
| Intermediate (58-80) | 63 | 20 | 14-35 | 0.86 | 0.61, 1.23 | 0.42 |
| Good (85-100) | 63 | 20 | 9-32 | 1 | - | - |
| *MHQ satisfaction score for side of surgery (tertiles) [3] (censored 3 poor, 2 intermediate; no missing data)* | | | | | | |
| Poor (0-25) | 88 | 21.5 | 13-42 | 0.78 | 0.55, 1.11 | 0.16 |
| Intermediate (29-50) | 49 | 18 | 14-29 | 0.87 | 0.59, 1.28 | 0.48 |
| Good (54-100) | 53 | 20 | 9-31 | 1 | - | - |
| *Satisfaction with appearance for side of surgery [4] (censored 2 satisfied, 3 dissatisfied; 1 missing)* | | | | | | |
| Satisfied | 136 | 20 | 10.5-31.5 | 1 | - | - |
| Dissatisfied | 53 | 20 | 13-40 | 0.84 | 0.61, 1.16 | 0.29 |

Total sample size n=195. Median duration of work absence relates to the 190 non-censored events.

1. Adjusted for age and sex.

2. Adapted from Calfee et al. [7].

3. Michigan Hand Questionnaire [8]. Data-driven tertiles.

4. Reported on a 5-point scale in response to the statement: I am satisfied with the appearance (look) of my hand. Dichotomised as satisfied (strongly agree/agree) and dissatisfied (neither agree nor disagree, disagree, strongly disagree).

## 4. Cox proportional hazards univariate analyses of the association between occupational factors and the duration of work absence after carpal tunnel release (non-significant findings)

| | N | Median time to return to work (days) | Inter quartile range | Hazards ratio [1] | 95% Confidence intervals | P value |
|---|---|---|---|---|---|---|
| *Have more than one paid job (censored 4 no, 1 yes; 1 missing)* | | | | | | |
| No | 177 | 20 | 12-32 | 1 | - | - |
| Yes | 12 | 26.5 | 19-42 | 0.61 | 0.34, 1.11 | 0.11 |
| *Total paid work hours per week [2] (censored 4 ≤37.5, 1 >37.5; no missing data)* | | | | | | |
| ≤37.5 | 97 | 22 | 15-38 | 1 | - | - |
| >37.5 | 93 | 16 | 6-29 | 1.25 | 0.88, 1.79 | 0.21 |
| *Number of work days per week (censored 1 <5, 4 5; no missing data)* | | | | | | |
| <5 | 55 | 23 | 15-35 | 1 | 0.72, 1.39 | 0.98 |
| 5 | 116 | 19 | 10.5-33 | 1 | - | - |
| >5 | 19 | 13 | 6-31 | 1.28 | 0.77, 2.14 | 0.34 |
| *Sick leave taken for this problem in the last month (censored 5 no; 16 missing)* | | | | | | |
| No | 154 | 20 | 12-33 | 1 | - | - |
| Yes | 20 | 15 | 9.5-30 | 0.99 | 0.61, 1.60 | 0.95 |
| *Sick leave taken for another problem in the last month (censored 4 no, 1 yes; 20 missing)* | | | | | | |
| No | 152 | 19 | 10.5-31 | 1 | - | - |
| Yes | 18 | 30 | 21-42 | 0.67 | 0.41, 1.09 | 0.10 |
| *Received advice about return to work after surgery (censored 5 yes; 3 missing)* | | | | | | |
| Yes | 134 | 19 | 12-31 | 1 | - | - |
| No | 53 | 22 | 14-41 | 0.92 | 0.67, 1.27 | 0.61 |
| *Required to work to tight deadlines [3] (censored 3 no, 2 yes; missing 3)* | | | | | | |
| No | 75 | 23 | 14-40 | 1 | - | - |
| Yes | 112 | 18 | 9.5-31 | 1.19 | 0.89, 1.61 | 0.24 |
| *Use power tools that make the hand/arm vibrate [3] (censored 5 no; missing 7)* | | | | | | |
| No | 145 | 19 | 12-32 | 1 | - | - |
| Yes | 38 | 22 | 14-42 | 0.68 | 0.44, 1.05 | 0.08 |
| *Work with hands above shoulder height for >1 hour [3] (censored 4 no, 1 yes; missing 9)* | | | | | | |
| No | 147 | 20 | 12-33 | 1 | - | - |
| Yes | 34 | 19.5 | 13-33 | 0.89 | 0.60, 1.32 | 0.56 |
| *Work with neck bent forward for >2 hours [3] (censored 3 no, 2 yes; missing 3)* | | | | | | |
| No | 114 | 21 | 13-39 | 1 | - | - |
| Yes | 73 | 18 | 8-31 | 1.19 | 0.88, 1.60 | 0.26 |
| *Work with neck twisted for >30 minutes [3] (censored 4 no, 1 yes; missing 7)* | | | | | | |
| No | 136 | 20 | 12-34 | 1 | - | - |
| Yes | 47 | 22 | 13-32 | 0.93 | 0.66, 1.32 | 0.70 |
| *Drive for >1 hour per day [3] (censored 5 no; missing 5)* | | | | | | |
| No | 115 | 21 | 13-38 | 1 | - | - |
| Yes | 70 | 16 | 7-31 | 1.22 | 0.88, 1.68 | 0.24 |

Total sample size n=195. Median duration of work absence relates to the 190 non-censored events.

1. Adjusted for age and sex.

2. Dichotomised by the median for the sample population

3. As part of the normal working day [9].

**References**


1. Sangha O, Stucki G, Liang MH et al. The self-administered comorbidity questionnaire: a new method to assess comorbidity for clinical and health services research *Arthritis & Rheumatism* 2003;49(2):156-63.
2. Terluin B, van Marwijk HW, Adèr HJ et al. The Four-Dimensional Symptom Questionnaire (4DSQ): a validation study of a multidimensional self-report questionnaire to assess distress, depression, anxiety and somatization. *BMC Psychiatry* 2006;6(1):34.
3. Ware J, Sherbourne C. The MOS 36-item short-form health survey (SF-36): I. conceptual framework and item selection. *Medical Care* 1992;30:473-83.
4. Hansen TB, Dalsgaard J, Meldgaard A et al. A prospective study of prognostic factors for duration of sick leave after endoscopic carpal tunnel release. *BMC Musculoskelet Disord* 2009;10:144.
5. Palmer K, Calnan M, Wainwright D et al. Upper limb pain in primary care: health beliefs, somatic distress, consulting and patient satisfaction. *Family Practice* 2006;23(6):609-17.
6. Bot AG, Becker SJ, Bruijnzeel H et al. Creation of the abbreviated measures of the Pain Catastrophizing Scale and the Short Health Anxiety Inventory: the PCS-4 and SHAI-5. *J Musculoskelet Pain* 2014;22(2):145-51.
7. Calfee RP, Dale AM, Ryan D et al. Performance of simplified scoring systems for hand diagrams in carpal tunnel syndrome screening. *J Hand Surg Am* 2012;37(1):10-17.
8. University of Michigan Medical School. The Michigan Hand Questionnaire: http://mhq.lab.medicine.umich.edu/. Accessed 04/04/2019.
9. Karasek R, Brisson C, Kawakami N et al. The Job Content Questionnaire (JCQ): an instrument for internationally comparative assessments of psychosocial job characteristics. *J Occup Health Psychol* 1998;3(4):322-55.
