# Supplementary material for: Sickness absence after carpal tunnel release: a multicentre prospective cohort study
Source: BMJ Open. 2021 Feb 10;11(2):e041656. doi: 10.1136/bmjopen-2020-041656 (PMC7878133; doi:10.1136/bmjopen-2020-041656)
Supplement: Author's manuscript [file bmjopen-2020-041656.draft_revisions.pdf]

BMJ Open

BMJ Open is committed to open peer review. As part of this commitment we make the peer review history of every article we publish publicly available.

When an article is published we post the peer reviewers' comments and the authors' responses online. We also post the versions of the paper that were used during peer review. These are the versions that the peer review comments apply to.

The versions of the paper that follow are the versions that were submitted during the peer review process. They are not the versions of record or the final published versions. They should not be cited or distributed as the published version of this manuscript.

BMJ Open is an open access journal and the full, final, typeset and author-corrected version of record of the manuscript is available on our site with no access controls, subscription charges or pay-per-view fees (http://bmjopen.bmj.com).

If you have any questions on BMJ Open's open peer review process please email info.bmjopen@bmj.com

BMJ Open

Sickness absence after carpal tunnel release: a multi-centre prospective cohort study

Journal:	*BMJ Open*
Manuscript ID	bmjopen-2020-041656
Article Type:	Original research
Date Submitted by the Author:	17-Jun-2020
Complete List of Authors:	Newington, Lisa; University of Southampton Faculty of Medicine, MRC Versus Arthritis Centre for Musculoskeletal Health and Work, MRC Lifecourse Epidemiology Unit; Guy's and Saint Thomas' NHS Foundation Trust, Hand Therapy<br>Ntani, G; University of Southampton Faculty of Medicine, MRC Versus Arthritis Centre for Musculoskeletal Health and Work, MRC Lifecourse Epidemiology Unit<br>Warwick, David; University Hospital Southampton NHS Foundation Trust, Hand Surgery; University of Southampton Faculty of Medicine<br>Adams, Jo; University of Southampton, Centre for Innovation and Leadership in Health Sciences, School of Health Sciences, Faculty of Environment and Life Sciences; University of Southampton, Centre for Sport, Exercise and Osteoarthritis Research Versus Arthritis<br>Walker-bone, Karen; University of Southampton Faculty of Medicine, MRC Versus Arthritis Centre for Musculoskeletal Health and Work, MRC Lifecourse Epidemiology Unit
Keywords:	Hand & wrist < ORTHOPAEDIC & TRAUMA SURGERY, PRIMARY CARE, OCCUPATIONAL & INDUSTRIAL MEDICINE

SCHOLARONE™
Manuscripts

I, the Submitting Author has the right to grant and does grant on behalf of all authors of the Work (as defined in the below author licence), an exclusive licence and/or a non-exclusive licence for contributions from authors who are: i) UK Crown employees; ii) where BMJ has agreed a CC-BY licence shall apply, and/or iii) in accordance with the terms applicable for US Federal Government officers or employees acting as part of their official duties; on a worldwide, perpetual, irrevocable, royalty-free basis to BMJ Publishing Group Ltd ("BMJ") its licensees and where the relevant Journal is co-owned by BMJ to the co-owners of the Journal, to publish the Work in this journal and any other BMJ products and to exploit all rights, as set out in our licence.

The Submitting Author accepts and understands that any supply made under these terms is made by BMJ to the Submitting Author unless you are acting as an employee on behalf of your employer or a postgraduate student of an affiliated institution which is paying any applicable article publishing charge ("APC") for Open Access articles. Where the Submitting Author wishes to make the Work available on an Open Access basis (and intends to pay the relevant APC), the terms of reuse of such Open Access shall be governed by a Creative Commons licence – details of these licences and which Creative Commons licence will apply to this Work are set out in our licence referred to above.

Other than as permitted in any relevant BMJ Author's Self Archiving Policies, I confirm this Work has not been accepted for publication elsewhere, is not being considered for publication elsewhere and does not duplicate material already published. I confirm all authors consent to publication of this Work and authorise the granting of this licence.

For peer review only

TITLE

Sickness absence after carpal tunnel release: a multi-centre prospective cohort study

AUTHORS

Lisa Newington [1, 2] *

Georgia Ntani [1]

David Warwick [3]

Jo Adams [4, 5]

Karen Walker-Bone [1]

AFFILIATIONS

1. MRC Versus Arthritis Centre for Musculoskeletal Health and Work, MRC Lifecourse Epidemiology Unit, University of Southampton, Southampton, UK.
2. Hand Therapy, Guy's and St Thomas' NHS Foundation Trust, St Thomas' Hospital, London, UK.
3. Hand Surgery, University Hospital Southampton NHS Foundation Trust and Faculty of Medicine, University of Southampton, Southampton, UK.
4. Centre for Innovation and Leadership in Health Sciences, School of Health Sciences, Faculty of Environment and Life Sciences, University of Southampton, Southampton, UK.
5. Centre for Sport, Exercise and Osteoarthritis Research Versus Arthritis, University of Southampton, Southampton, UK.

*** Corresponding author**

Address: Hand Therapy, Guy's and St Thomas' NHS Foundation Trust, St Thomas' Hospital, London, SE1 7EH, UK.

Email: l.newington@nhs.net

Telephone: 0207 7188 4174

Fax: none

KEY WORDS

Carpal tunnel syndrome, carpal tunnel release, return to work, advice, sickness absence, cohort study

Word count: 3,228

For peer review only

TITLE

Sickness absence after carpal tunnel release: a multi-centre prospective cohort study

ABSTRACT

Objectives: To describe when patients return to different types of work after elective carpal tunnel release (CTR) surgery and identify the factors associated with the duration of sickness absence.

Design: Multi-centre prospective observational cohort study.

Setting and participants: Participants were recruited pre-operatively from 16 UK centres and clinical, occupational and demographic information were collected. Participants completed a weekly diary and questionnaires at four and 12 weeks post-operatively.

Outcomes: The main outcome was duration of work absence from date of surgery to date of first return to work.

Results: 254 participants were enrolled in the study and 201 provided follow-up data. Median duration of sickness absence was 20 days (range 1-99). Earlier return to work was associated with having surgery in primary care and a self-reported work role involving more than four hours of daily computer use. Being female and entitlement to more than a month of paid sick leave were both associated with longer work absences. Duration of work absence was strongly associated with the expected duration of leave, as reported by participants before surgery. Earlier return to work was not associated with poorer clinical outcomes reported 12 weeks after CTR.

Conclusions: There was wide variation in the duration of work absence after CTR across all occupational categories. A combination of occupational, demographic and clinical factors was

For peer review only

associated with the duration of work absence, illustrating the complexity of return to work decision-making. However, pre-operative expectations were strongly associated with the actual duration of leave. We found no evidence that earlier RTW was harmful. Clear, consistent advice from clinicians pre-operatively setting expectations of a prompt return to work could reduce unnecessary sickness absence after CTR. To enable this, clinicians need evidence-informed guidance about appropriate timescales for the safe return to different types of work.

STRENGTHS AND LIMITATIONS OF THIS STUDY

- This multi-centre, prospective study with a large sample size provides robust evidence to understand return to work issues after carpal tunnel release surgery.
- Participants were recruited from primary care, secondary care and private practice sites, representing the breath of locations where carpal tunnel release is performed in the UK.
- Work absence was the primary outcome and a clear definition was used for its duration with data collected contemporaneously to limit recall bias.
- A standardised method was used to categorise occupations and measure occupational exposures, although this relied on job title, which may not be a true reflection of actual occupational activity.
- All participants underwent open carpal tunnel release, however the method of carpal tunnel syndrome diagnosis was not independently verified and different case definitions for carpal tunnel syndrome may have been included.

For peer review only

INTRODUCTION

Carpal tunnel syndrome (CTS) occurs when the median nerve becomes compressed within the carpal tunnel at the wrist. Typical sensory symptoms include pain, paraesthesia and/or numbness in the thumb, index, middle and radial half of the ring finger; and motor symptoms include progressive wasting of the thenar muscles. CTS is often associated with marked functional difficulty[1] and treatment is targeted at reducing the median nerve compression by splinting, corticosteroid injection or carpal tunnel release (CTR) surgery[2, 3].

Recent estimates suggest that more than 90,000 CTR procedures will be performed annually in the English NHS by 2025[4], and as the peak incidence for CTS falls within the working lifetime[5], many of these patients will need to return to work after their CTR. However, there is currently no evidence on which to base recommendations about when it might be safe to return to functional activities, including work, after carpal tunnel release (CTR). Our previous survey of UK hand surgeons, primary care surgeons and hand therapists found that clinicians recommended a wide range of times to return to three specified job roles after CTR: 0-30 days for desk-based work (e.g. keyboard, mouse, writing and telephone); 1-56 days for repetitive light manual work (driving, delivery, stacking); and 1-90 days for heavy manual work (e.g. construction)[6]. However, there has not previously been a prospective study of CTR patients in the UK in which time to return to work was the primary outcome. Therefore, it is not known when UK patients return to different occupational activities after CTR or what influences the duration of work absence. It is also unclear whether earlier return to work has a detrimental effect on post-operative clinical outcomes. Possible consequences of returning to work too soon after CTR include wound dehiscence, infection, and delayed healing. Conversely, delayed return to work may increase the risk of progression to long-term sick leave[7] and produce a financial burden for the individual, employer or state.

For peer review only

A systematic review of the prognostic factors associated with return to work after CTR identified 11 studies which evaluated more than 90 potential prognostic factors[8]. Longer durations of work absence after CTR were found to be associated with: receipt of workers' compensation[9]; manual work[10-12]; longer expected durations of work absence[10]; being on sick leave before CTR surgery[13]; self-blame for the hand problem[13]; and beliefs that the symptoms were caused by work[12].

Much of the existing research has been conducted at single sites and involved small numbers of participants. Furthermore, very few studies have specifically explored the influence of a range of occupational factors. The current multi-centre prospective cohort study was designed to explore when patients returned to different types of work after CTR and the demographic, clinical and occupational factors associated with duration of work absence. We also investigated whether earlier return to work was associated with poorer clinical outcomes assessed at 12 weeks after CTR.

METHODS

This was a prospective cohort study and a convenience sample of participants were recruited from 16 sites in England and Wales between March 2017 and August 2018. Recruitment took place before CTR surgery, either at the time of listing for surgery, during pre-operative screening, or on the day of surgery. At each site, the patient CTR pathway and treatment was carried out as usual. Sites were NHS secondary care (hospital setting), NHS primary care and private hand surgery facilities, representing the range of UK healthcare facilities where CTR is performed. Eligibility criteria are shown in Table 1. Baseline demographic, general health and occupational information was collected via self-completed questionnaire at the time of

For peer review only

recruitment. The questionnaire also included standardised measures of CTS symptoms[14-16] and hand function[17]. Questionnaire content was informed by the clinical, demographic and occupational factors previously shown to be associated with the duration of work absence after CTR[8].

TABLE 1. Study eligibility criteria

**Self-selected by potential participants**
1. Aged over 18 and referred for carpal tunnel release surgery
2. Routinely work in paid employment for at least 20 hours per week
3. Plan to return to work after carpal tunnel release surgery
4. Have not previously had carpal tunnel release surgery on either hand
5. Have not previously had a serious injury to the same wrist/hand
**Assessed by recruiting clinician**
1. No planned surgical procedures for conditions other than carpal tunnel syndrome

Follow-up questionnaires were completed four and 12 weeks after CTR and collected information about return to work, work functioning, scar symptoms, CTS symptoms, and hand function. Study questionnaires are provided as supplementary files 1-2. Participants were also asked to complete a short weekly diary from the day after surgery until return to work, detailing whether they had returned to work that week, and if so, the date of return.

Surgical information was collected from the medical records by a member of the participant's clinical team. This included: date of CTR, operated hand(s), nature of anaesthetic, incision size, additional procedures, unexpected findings and suture material. Date, side of CTR and anaesthetic (general/local) were also reported by participants for cross-checking.

For peer review only

Public and patient involvement

This research was supported by a patient advisory group consisting of six individuals who had previously undergone CTR at different UK sites. Study questionnaires were developed in collaboration with the patient advisors and these individuals also provided their feedback on the preliminary findings.

Statistical methods

Comparisons were made between those who dropped out of the study before providing any follow-up data and those in the final study sample using pre-specified demographic, clinical and occupational variables (Table 2). Categorical variables were assessed using Chi^2 test, continuous variables were plotted and assessed using *t*-test if they were normally distributed or Wilcoxon rank-sum test if the distribution was skewed. There was no imputation for missing data.

Manual and non-manual work was coded from job title and industry using the UK Standard Occupational Classification[18, 19]. Return to work time was calculated from the date of surgery to the date of first return to work (as reported by participants).

A Cox proportional hazards model was used to explore the factors associated with return to work time. Baseline and operative variables were assessed in univariate analyses and those which were significant ($p<0.05$) were included as covariates in the final model. All regression analyses were adjusted for age and sex.

Participants were defined as having a poor outcome if they reported one or more of the following: global rating of change score of “worse”, “unchanged” or “slightly improved” (12

For peer review only

weeks after CTR)[20]; scar symptoms described as “unbearable”, “very troublesome” or “fairly troublesome” (12 weeks after CTR); use of antibiotics for an incision site infection after returning to work; and additional sick-leave related to the CTR after returning to work. The duration of work absence for those with/without poor outcomes were compared using Wilcoxon rank-sum test. In addition, participants were dichotomised to those who returned to work within/after seven, 14 and 28 days of surgery and the prevalence rates of poor outcomes were compared using Chi^2 for each time period. These time points were based on the median clinician-recommended return to work time that we reported previously[6].

RESULTS

A total of 254 individuals completed the baseline questionnaire and 201 (79%) provided follow-up data. Participant numbers and loss to follow-up for each study component are shown in Figure 1. Participant demographics and comparisons between those who remained in the study and those who dropped out before follow-up are shown in Table 2.

Figure 1. Participant numbers for each stage of the cohort study

TABLE 2. Participant demographics assessed at baseline in comparison with those lost to follow-up

	**Study participants n=201 (%)**	**Lost to follow-up n=53 (%)**	**P-value**
Mean age in years (SD)	52.0 (9.16)	44.4 (9.55)	**<0.001**
Sex			
Male	76 (37.8)	20 (37.7)	0.99
Female	125 (62.2)	33 (62.3)	
Body mass index (kg/m²)			
Normal (18.5-24.9)	48 (23.9)	9 (17.0)	0.51
Overweight (25-29.9)	66 (32.8)	16 (30.2)	
Obese (≥30)	73 (36.3)	22 (41.5)	

Smoking status			
Never smoked	109 (54.2)	26 (49.1)	0.58
Current/ex-smoker	90 (44.8)	27 (50.9)	
General health			
Excellent/very good/good	174 (86.6)	42 (79.3)	0.32
Fair/poor	26 (12.9)	11 (20.8)	
Number of comorbidities			
None	54 (26.9)	21 (39.6)	0.15
One	70 (34.8)	13 (24.5)	
Two or more	77 (38.3)	19 (35.9)	
Number of disabling comorbidities			
None	138 (68.7)	35 (66.0)	0.46
One	41 (20.4)	9 (17.0)	
Two or more	22 (11.0)	9 (17.0)	
Mean SF-36 mental health score (SD) [a]	65.6 (17.20)	60.3 (20.41)	**0.03**
Mean bilateral CTS-6 score (SD) [b]	2.8 (0.77)	3.0 (0.73)	0.95
Mean MHQ bilateral activities of daily living score (SD) [c]	68.8 (23.64)	55.7 (28.62)	**<0.001**
Mean MHQ work function score (SD) [c]	66.1 (22.26)	60.6 (22.61)	0.06
Type of job contract			
Employed (permanent contract)	164 (81.6)	37 (69.8)	0.23
Self-employed	31 (15.4)	13 (24.5)	
Employed (temporary or zero hours contract)	5 (2.5)	3 (5.7)	
Type of work [d]			
Manual	77 (39)	31 (58)	**0.01**
Non-manual	123 (61)	22 (42)	
Median level of job demand on hands/wrists (IQR) [e]	9 (7-10)	10 (7-10)	0.15
Job satisfaction			
Very satisfied	87 (43.3)	24 (45.3)	0.90
Satisfied/fairly satisfied	92 (45.8)	24 (45.3)	
Dissatisfied/very dissatisfied	20 (10.0)	5 (9.4)	
Median expected work absence in days (IQR)	14 (7-28)	14 (5-21)	0.46
Expected availability of sick pay			
≤1 month	50 (24.9)	21 (39.6)	**0.001**
>1 month	94 (46.8)	11 (20.8)	
Unsure	57 (28.4)	21 (39.6)	
Study site [f]			
NHS primary care	73 (36.3)	13 (24.5)	0.27
NHS Secondary care	101 (50.3)	32 (60.4)	
Private hand surgery facilities	27 (13.4)	8 (15.1)	

[a.] SF-36 mental health score ranges from 0-100, higher scores indicate better mental health[21].
[b.] CTS-6 symptom score ranges from 1-5, higher scores indicate more severe symptoms[14].
[c.] Michigan Hand Questionnaire ranges from 0-100, higher scores indicate better functioning[17].
[d.] Classified using the Office for National Statistics Standard Occupational Classification 2010[18, 19].
[e.] Job demand scale range 0-10, 10 indicating very demanding on hands/wrists[13].
[f.] Location where the carpal tunnel release surgery was performed. Surgery in primary care was performed by General Practitioners who had completed additional training.

For peer review only

Eighty-six participants (43%) were recruited pre-operatively on the day of their CTR. For the remaining 115 participants, the median time between recruitment and CTR was 14 days (interquartile range [IQR] 5-40). The first follow-up questionnaire was completed a median of 32 days after CTR (IQR 29-38) and the final questionnaire was completed a median of 92 days after CTR (IQR 86-105). All participants underwent open CTR as a day case, and all but two had unilateral surgery. Another two participants required median nerve neurolysis, and one participant was noted to have a vascular abnormality. Sixty-two percent of participants (n=125) were expecting to have a CTR for their other hand in the future. Other baseline demographic and surgical factors are shown in Table 3.

TABLE 3. Participant demographic and surgical factors

	Number of participants n=201 (%)
Age (years)	
26-40	23 (11.4)
41-55	101 (50.3)
≥ 55	77 (38.3)
Hand dominance	
Right	178 (88.6)
Left	18 (9.0)
Ambidextrous	5 (2.5)
Side of surgery [a]	
Dominant hand	134 (66.7)
Non-dominant hand	65 (32.3)
Bilateral surgery	2 (1.0)
Surgical speciality and grade	
Consultant (plastics/orthopaedics)	64 (31.8)
Registrar (plastics/orthopaedics)	33 (16.4)
General practitioner	62 (30.9)
Not reported	42 (20.9)
Incision type [b]	
Mini open incision	129 (64.2)
Traditional incision	2 (1.0)
Not reported	70 (34.8)
Suture material	
Absorbable	24 (11.9)
Non-absorbable	126 (62.7)
Not reported	51 (24.4)

[a.] Considered as the non-dominant hand for those who reported ambidexterity.
[b.] Mini open incision defined as distal to the distal wrist crease and traditional open excision extending proximally.

For peer review only

The majority of participants (62%) worked five days per week (range 2-7) and the median number of weekly paid work hours was 37.5 (IQR 31-45). Two participants (1%) did not return to work during the 12-week study period: one reported that they had been made redundant and the other that their job was no longer available. Four participants (2%) had incomplete data (missing return to work date or CTR date) meaning that the duration of work absence could not be calculated. These six individuals were not included in the analyses of return to work time, leaving a total sample size of 195. An additional five participants reported that they had not returned to work, but planned to do so in the future. These individuals were included in the return to work analysis, censored to the time of last follow-up.

The median duration of work absence after CTR was 20 days (IQR 12-33). Manual workers took longer to return than non-manual workers: 23.5 days (IQR 14-41) compared with 18 days (IQR 9-31). Those who were self-employed returned to work earlier than those who were employed: 13 days (IQR 6-19) compared with 22 days (IQR 14-38). Return to work times are shown in Figure 2. The majority of participants returned to work on a Monday (43%). Approximately 15% returned each day between Tuesday-Thursday, then ~5% returned each day from Friday-Sunday. More than half of participants (59%) reported that they needed to modify their work duties to some extent when they first returned to work. Of these, 62% had resumed full duties within five weeks.

Univariate analyses of the relationship between baseline factors and the duration of work absence found 17 factors (age- and sex-adjusted) that were significantly associated with time to return to work and were entered into the multivariate model, in which five factors remained significant (Table 4). Sensitivity analyses confirmed that these factors were independently significant in the model with no collinearity. Non-significant findings in the univariate analyses

For peer review only

are provided in supplementary file 3. Having surgery in primary care and having a job with more than four hours of daily computer use were both associated with earlier return to work than their respective reference categories. Being female and having access to more than a month of paid sick leave were both associated with longer durations of work absence than their respected reference categories. Compared to those who expected to return within a week, there was a sequential increase in the likelihood of longer durations of work absence for those expecting to return between 7-14 days, 15-30 days and >30 days (Table 4), which showed a significant gradient effect ($p<0.001$).

For peer review only

TABLE 4. Cox proportional hazards analyses of the association between baseline demographic, clinical and occupational factors and the duration of work absence after carpal tunnel release

				Univariable analyses			Multivariable analysis		
	**N**	**Median work absence (days)**	**Inter quartile range**	**Hazards ratio**	**95% Confidence intervals**	**P value**	**Hazard s ratio**	**95% Confidence intervals**	**P value**
Sex									
Male	72	17.5	8-31	1	-	-	1	-	-
Female	118	21.5	14-35	0.79	0.59, 1.06	0.12	0.56	0.36, 0.88	**0.01**
Age in years									
26-40	21	20	15-30	1.01	0.63, 1.63	0.96	1.44	0.82, 2.55	0.21
41-55	94	20	9-33	1	-	-	1	-	-
>55	75	17	12-35	1.03	0.76, 1.40	0.83	1.15	0.80, 1.65	0.44
Smoking status									
Never	105	16	12-31	1	-	-	1	-	-
Current/ex	83	23	13-41	0.74	0.56, 1.00	0.046	0.75	0.51, 1.09	0.13
Site									
NHS Primary care	72	19.5	13-33	1.18	0.87, 1.62	0.29	1.54	1.05, 2.25	**0.03**
NHS Secondary care	92	20	12-39.5	1	-	-	1	-	-
Private facilities	26	20	7-28	1.63	1.04, 2.54	0.03	1.46	0.87, 2.44	0.15
Afraid of long-term hand problems [a]									
No	105	19	12-31	1	-	-	1	-	-
Yes	82	20.5	13-42	0.69	0.51, 0.93	0.01	0.93	0.67, 1.30	0.68
CTS-6 score for side of surgery (tertiles) [b]									
Good (1-3.0)	65	16	10-27	1	-	-	1	-	-
Intermediate (3.2-3.8)	58	21.5	14-35	0.77	0.54, 1.10	0.15	1.19	0.77, 1.84	0.44
Poor (8.8-5)	59	24	13-41	0.67	0.47, 0.97	0.03	1.04	0.65, 1.66	0.87
Type of work contract									
Employed (permanent)	154	22	14-38	1	-	-	1	-	-
Self-employed	30	13	6-19	1.72	1.13, 2.61	0.01	1.19	0.67, 2.14	0.55
Zero hours/temporary	5	12	3-31	2.01	0.81, 5.00	0.13	0.73	0.25, 2.14	0.56
Duration of available sick pay									
≤1 month	49	16	12-29	1	-	-	1	-	-
>1 month	88	27	15-42	0.59	0.41, 0.85	0.004	0.46	0.28, 0.76	**0.002**
Unsure	53	16	10-23	1.19	0.80, 1.77	0.40	1.01	0.61, 1.66	0.97
Access to occupational health at work									
No	110	15.5	9-29	1.77	1.31, 2.38	<0.001	1.42	0.91, 2.19	0.12
Yes	79	25	16-42	1	-	-	1	-	-
Expected duration of leave after carpal tunnel release (days)									
<7	35	4	2-12	1	-	-	1	-	-
7-14	75	16	13-26	0.23	0.15, 0.36	<0.001	0.27	0.16, 0.45	**<0.001**
15-29	35	29	22-39	0.12	0.07, 0.19	<0.001	0.19	0.10, 0.37	**<0.001**
≥30	45	42	21-44	0.08	0.05, 0.14	<0.001	0.12	0.06, 0.23	**<0.001**
MHQ work functioning score (tertiles) [c]									
Poor (0-55)	67	20	13-35	0.68	0.47, 0.98	0.04	0.83	0.50, 1.40	0.49
Intermediate (60-80)	72	21	12.5-39.5	0.77	0.53, 1.10	0.15	0.81	0.50, 1.31	0.39
Good (81-100)	51	17	10-29	1	-	-	1	-	-
Job satisfaction [d]									
Satisfied	169	19	11-31	1	-	-	1	-	-
Dissatisfied	19	38	21-43	0.61	0.38, 0.99	0.04	0.67	0.38, 1.16	0.15
Believe that the hand problem was caused by work [e]									
No	112	19	13-31	1	-	-	1	-	-
Agree/strongly agree	76	23	10-42	0.62	0.46, 0.85	0.003	0.82	0.57, 1.17	0.28
Job is demanding on hands/wrists [a]									
No	35	16	6-27	1	-	-	1	-	-

For peer review only

Yes	155	20	13-38	0.61	0.42, 0.89	0.01	0.68	0.42, 1.12	0.13
Type of work [f]									
Non-manual	119	18	9-31	1	-	-	1	-	-
Manual	70	23.5	14-41	0.66	0.48, 0.89	0.01	0.97	0.57, 1.64	0.90
Work involves target-driven pay [g]									
No	149	19	12-31	1	-		1	-	-
Yes	31	22	13-45	0.61	0.41, 0.91	0.02	0.97	0.59, 1.61	0.91
Duration of computer use at work (hours) [g]									
<1	69	28	17-42	1	-	-	1	-	-
>1 to <4	33	16	10-31	2.20	1.43, 3.38	<0.001	1.01	0.56, 1.81	0.98
≥4	84	16	7-27	2.38	1.67, 3.38	<0.001	1.85	1.08, 3.16	**0.03**
Work involves lifting or carrying ≥10 kg [g]									
No	108	18.5	11-30	1	-	-	1	-	-
Yes	77	24	13-40	0.61	0.42, 0.86	0.01	0.80	0.48, 1.33	0.39
Work involves pushing/pulling a heavy weight [g]									
No	104	16	8.5-28.5	1	-	-	1	-	-
Yes	83	26	16-42	0.51	0.37, 0.70	<0.001	0.97	0.61, 1.55	0.90

Total sample size n=195. All analyses were adjusted for age and sex. All significant variables in the univariate analyses ($p<0.05$) were entered into the multivariable model.

[a.] Reported on a 0-10 scale, dichotomised to no (0-6) and yes (7-10)[13].

[b.] CTS-6 score [14] with data driven tertiles.

[c.] Michigan Hand Questionnaire work performance subscale scored from 0-100, higher scores indicate better perceived work performance[17]. Data driven tertiles.

[d.] Reported on a 5-point scale, dichotomised to satisfied (very satisfied/satisfied/fairly satisfied) and dissatisfied (dissatisfied/very dissatisfied).

[e.] Reported on a 5-point scale and dichotomised to agree/strongly agree and neither agree nor disagree/disagree/disagree strongly[22].

[f.] Classified using the Office for National Statistics Standard Occupational Classification 2010[18, 19].

[g.] As part of the normal working day[23].

For peer review only

Clinical outcomes after CTR are shown in Table 5. Using the definition outlined in the methods, a total of 46 participants (24%) were identified as having at least one poor outcome (CTS symptoms that were worse, unchanged or only slightly better; scar symptoms that were at least fairly troublesome; required post-operative antibiotics or had additional time off work after first return). Of these participants, the majority (n=38, 83%) reported only a single component of poor outcome. Three individuals defined as having a poor outcome had not returned to work at the point of last follow-up (as compared with two individuals in the rest of the study sample). For those who had returned to work, the median duration of work absence for those with a poor outcome was 22 days (IQR 12-42) compared with 19 days (IQR 12-32) for those without (Figure 2). This difference was not significant (Wilcoxon rank-sum, p=0.24).

TABLE 5. Clinical outcomes after carpal tunnel release

	**Mean score (standard deviation)**	
	Before surgery	12 weeks after surgery
CTS-6 (operated hand) [a]	3.3 (0.87)	1.2 (0.54)
MHQ function (operated hand) [b]	50 (22.1)	79 (19.4)
MHQ satisfaction with function (operated hand) [b]	38 (25.7)	82 (21.3)
MHQ bilateral activities of daily living [b]	69 (23.7)	88 (13.8)
MHQ activities of daily living (operated hand) [b]	65 (28.1)	87 (18.5)
	**Number of participants (%)**	
Global rating of change score		
Completely cured	-	65 (33.3)
Much better	-	98 (50.3)
Slightly better	-	13 (6.7)
Unchanged	-	2 (1.0)
Worse	-	5 (2.6)
Scar symptom severity	-	
Not at all troublesome	-	99 (50.8)
A little troublesome	-	63 (32.3)
Fairly troublesome	-	18 (9.2)
Very troublesome	-	2 (1.0)
Unbearable	-	0
Required post-operative antibiotics	-	10 (5.1)
Additional sick leave after first returning to work	-	12 (6.2)

Grey shading indicates the categories which were combined to define a poor surgical outcome.
[a.] CTS-6 assessment of carpal tunnel syndrome symptoms[14]. Range 1-5: 1 equals no symptoms.
[b.] MHQ Michigan Hand Questionnaire[17]. Range 0-100: 100 equals no deficit or dissatisfaction.

For peer review only - http://bmjopen.bmj.com/site/about/guidelines.xhtml

Figure 2. Duration of work absence after carpal tunnel release

There was no significant difference in the prevalence of a poor outcome among those who returned to work within or after seven days of CTR (20% versus 24%, Chi^2 p=0.63). Similarly, there was no significant difference in the prevalence of a poor outcome among those who returned to work within or after 14 days (19% versus 25%, Chi^2 p=0.31), or within or after 28 days of CTR (21% versus 27%, Chi2 p=0.33).

DISCUSSION

In this multi-site prospective cohort study, the median duration of work absence was 20 days (range 1-99), a duration similar to that reported by other European studies[24]. Earlier return to work was associated with typing for ≥4 hours at work (as compared with more physical workplace tasks) and undergoing surgery in primary care (as compared with secondary care or private practice). Pre-operative expectations about return to work were important significant predictors of actual return to work times. We found no evidence of poorer clinical outcomes in the first 12 weeks amongst those who returned to work earlier. At each time point, fewer manual workers had returned to work than non-manual workers and fewer employed workers had returned than self-employed (Figure 2). Both findings have been reported previously[10, 11, 25, 26], however, neither the type of work (manual/non-manual) nor the type of work contract (employed/self-employed) were significantly associated with the duration of work absence in the mutually adjusted model. These results illustrate the importance of considering the range of relevant demographic, clinical and occupational factors, which may have been confounders, moderators or mediators in previous studies.

For peer review only

For peer review only - http://bmjopen.bmj.com/site/about/guidelines.xhtml

Five variables were independently associated with longer duration of work absence in the final model. Two were occupational factors: infrequent computer use and availability of sick pay. Cowan et al.[10], recorded earlier return to work after CTR for desk-based workers and we have shown previously that UK hand surgeons and hand therapists report that they advise earlier return to desk-based workers[6]. The relationship between longer duration of work absence and availability of sick pay has also be reported previously for those with and without worker's compensation[9, 24]. It is plausible that financial necessity is driving earlier return to work for those with limited sick pay. Alternatively, those with access to paid leave might choose to take the maximum available duration.

Only one clinical factor was significantly associated with the duration of work absence: participants who had their CTR surgery in primary care were more likely to return to work earlier than those whose procedure took place in an NHS hospital setting. One possible reason is that patients with more complex disease may be more commonly referred to secondary, rather than primary, care for their CTR and these patients may require longer off work after their surgery. However, in the current study the proportion and degree of comorbidities, and the extent of pre-operative symptoms reported by participants were similar across all settings. Another possibility is that the patients' expectations of the surgery may be different: CTR performed in a primary care setting might be perceived by patients as being more minor than surgery in a hospital operating theatre. Alternatively, the general practice surgeons may have recommended earlier return to work than those based in a hospital, although the median expected duration of work absence for participants in the current study was the same across all settings.

For peer review only

For peer review only - http://bmjopen.bmj.com/site/about/guidelines.xhtml

Only one demographic factor was statistically significant: women were more likely to take longer to return to work than men. Whilst we found inconsistent data about the effect of gender on return to work after CTR in the literature[27, 28], female gender has been associated with longer periods of work absence for other health conditions[29]. Researchers should continue to include sex as a covariate in analyses of return to work outcomes, although there is currently insufficient evidence to support any difference in return to work recommendations after CTR based on sex. Further qualitative investigation is required in order to understand the context for this.

Finally, those who expected to return to work more quickly were significantly more likely to do so. It has been shown previously that patient expectations are a prominent determinant of return to work time, or other return to work outcomes, for musculoskeletal or mental health conditions[29-31]. The role of expectations on outcomes, including the expected and actual timing for return to work and driving after hand and wrist surgery, requires further exploration, particularly because expectations are a potentially modifiable characteristic which could be influenced by the advice provided by clinicians pre-operatively.

In total, approximately a quarter of participants in this study were considered to have a poor outcome using our composite definition. We chose to use a multi-component definition which was deliberately very stringent to minimise the chances of missing any harm caused by early return to work. Our rates of poorer outcomes were in fact similar to those reported in other CTR populations[20, 32, 33]. Importantly, we found no relationship between earlier return to work and occurrence of poor outcomes within 12 weeks of CTR in this cohort study.

For peer review only - http://bmjopen.bmj.com/site/about/guidelines.xhtml

There are a number of limitations of the current study, including the reliance on self-reported data. Work absence is not logged on a national database in the UK and therefore could only be obtained through self-report. To minimise errors of recall, date of return to work was determined contemporaneously. The recall duration for measures of function and symptoms was limited to a maximum of four weeks, consistent with the outcome measures used[14, 17]. We set out to recruit a large sample of working-aged adults undergoing CTR. Our prospectively recruited sample from 16 sites is one of the largest reported in the literature to date, with a good follow up response rate (79%), but it remains possible that we were under-powered to detect some of the factors which may have been associated with delayed return to work. The findings may also not be generalisable to working populations in regions outside of central and southern England and Wales or employed in other industries. The observed differences between those who completed the study and those who were lost to follow-up (younger, better mental health, more likely manual workers) may further limit the generalisability of the findings.

All participants were presumed to have CTS as diagnosed by their treating clinician. Many studies of CTS include nerve conduction study (NCS) findings as part of their eligibility criteria, although this was not possible in the current study because NCS are not routinely recommended for pre-operative diagnosis of CTS in the UK[2]. Our eligibility criteria required that only people undergoing their first CTR were included and reported upon in this study (so that previous experiences with CTR were not potential confounders). However, more than three quarters of the cohort reported bilateral symptoms. The possible impact of persisting CTS symptoms in the non-operated hand on return to work also needs to be considered.

For peer review only

For the current study, we considered both occupational title and self-reported occupational exposures collected in a standardised questionnaire format[13, 18, 19, 23]. Categorisation based on job title and industry may not accurately reflect the physical and/or psychosocial aspects of job role. There is a need for an agreed approach to identifying and recording key physical demands and psychosocial exposures of jobs to enable consistent exploration of their impact on work and clinical outcomes following surgery or other intervention. Approaches such as job exposure matrices[34] could facilitate this in future research.

In summary, this large multi-centre prospective cohort study investigated when participants return to work after CTR. Expectations about return to work (reported before surgery) were strongly associated with actual work absence, regardless of the job role or self-reported upper limb activities involved. Patient expectations can be influenced by many factors, but one of the most important is the advice provided by clinicians, in particular the surgeon. This suggests that clear, consistent advice could have an important effect on duration of sick leave. To date there is no evidence-based guidance informing clinicians what to advise about returning to different types of work after CTR. Further research is required to reach a consensus and explore whether the provision of targeted, consistent and standardised advice can alter the expected duration of work absence, reducing unnecessary sick leave, without causing adverse effects on clinical outcomes.

For peer review only

REFERENCES

1. Jerosch-Herold C, Mason R, Chojnowski AJ. A qualitative study of the experiences and expectations of surgery in patients with carpal tunnel syndrome. *J Hand Ther* 2008;21(1):54-61 doi:10.1197/j.jht.2007.09.001.
2. British Orthopaedic Association. Commissioning guide: Treatment of carpal tunnel syndrome. London: Royal College of Surgeons; 2017.
3. American Academy of Orthopaedic Surgeons. Management of carpal tunnel syndrome evidence-based clinical practice guideline. Illinois: American Academy of Orthopaedic Surgeons; 2016.
4. Bebbington E, Furniss D. Linear regression analysis of Hospital Episode Statistics predicts a large increase in demand for elective hand surgery in England. *J Plast Reconstr Aesthet Surg* 2015;68(2):243-51 doi:10.1016/j.bjps.2014.10.011.
5. Burton CL, Chen Y, Chesterton LS et al. Trends in the prevalence, incidence and surgical management of carpal tunnel syndrome between 1993 and 2013: an observational analysis of UK primary care records. *BMJ Open* 2018;8(6) doi:10.1136/bmjopen-2017-020166.
6. Newington L, Francis K, Ntani G et al. Return to work recommendations after carpal tunnel release: a survey of UK hand surgeons and hand therapists. *J Hand Surg Eur* 2018;43(8):875-78 doi:10.1177/1753193418786375.
7. Black C. Working for a healthier tomorrow. London, UK: Health Work and Wellbeing Programme 2008.
8. Peters S, Johnston V, Hines S et al. Prognostic factors for return-to-work following surgery for carpal tunnel syndrome: a systematic review. *JBI Database Syst Rev Implement Rep* 2016;14(9):135-216 doi:10.11124/jbisrir-2016-003099.
9. Dunn JC, Kusnezov NA, Koehler LR et al. Outcomes following carpal tunnel release in patients receiving workers' compensation: a systematic review. *Hand* 2018;13(2):137-42 doi:10.1177/1558944717701240.
10. Cowan J, Makanji H, Mudgal C et al. Determinants of return to work after carpal tunnel release. *J Hand Surg Am* 2012;37(1):18-27
11. De Kesel R, Donceel P, De Smet L. Factors influencing return to work after surgical treatment for carpal tunnel syndrome. *Occup Med* 2008;58(3):187-90

For peer review only - http://bmjopen.bmj.com/site/about/guidelines.xhtml

12. Parot-Schinkel E, Roquelaure Y, Ha C et al. Factors affecting return to work after carpal tunnel syndrome surgery in a large french cohort. *Arch of Phys Med Rehabil* 2011;92(11):1863-69 doi:http://dx.doi.org/10.1016/j.apmr.2011.06.001.
13. Hansen TB, Dalsgaard J, Meldgaard A et al. A prospective study of prognostic factors for duration of sick leave after endoscopic carpal tunnel release. *BMC Musculoskelet Disord* 2009;10:144
14. Atroshi I, Lyren PE, Ornstein E et al. The six-item CTS symptoms scale and palmar pain scale in carpal tunnel syndrome. *J Hand Surg Am* 2011;36(5):788-94 doi:10.1016/j.jhsa.2011.02.021.
15. Calfee RP, Dale AM, Ryan D et al. Performance of simplified scoring systems for hand diagrams in carpal tunnel syndrome screening. *J Hand Surg Am* 2012;37(1):10-17 doi:10.1016/j.jhsa.2011.08.016.
16. Katz JN, Stirrat CR. A self-administered hand diagram for the diagnosis of carpal tunnel. *J Hand Surg Am* 1990;15:360-63
17. University of Michigan Medical School. The Michigan Hand Questionnaire: http://mhq.lab.medicine.umich.edu/. Accessed 04/04/2019.
18. Office for National Statistics. Standard occupational classification for the UK: https://www.ons.gov.uk/methodology/classificationsandstandards/standardoccupationalclassificationsoc/soc2010. Accessed 08/06/2018.
19. Warwick Institute for Employment Research. Cascot: Computer assisted structured coding tool https://warwick.ac.uk/fac/soc/ier/software/cascot/. Accessed 15/05/2019.
20. Jerosch-Herold C, Shepstone L, Houghton J et al. Prognostic factors for response to treatment by corticosteroid injection or surgery in carpal tunnel syndrome (PaLMS study): a prospective multi-centre cohort study. *Muscle Nerve* 2019;60(1):32-40 doi:doi:10.1002/mus.26459.
21. Ware J, Sherbourne C. The MOS 36-item short-form health survey (SF-36): I. conceptual framework and item selection. *Medical Care* 1992;30:473-83
22. Palmer K, Calnan M, Wainwright D et al. Upper limb pain in primary care: health beliefs, somatic distress, consulting and patient satisfaction. *Family Practice* 2006;23(6):609-17 doi:10.1093/fampra/cml047.

For peer review only

For peer review only - http://bmjopen.bmj.com/site/about/guidelines.xhtml

23. Karasek R, Brisson C, Kawakami N et al. The Job Content Questionnaire (JCQ): an instrument for internationally comparative assessments of psychosocial job characteristics. *Journal Occup Health Psychol* 1998;3(4):322-55

24. Newington L, Stevens M, Warwick et al. Sickness absence after carpal tunnel release: a systematic review of the literature. *Scand J Work Environ Health* 2018;44(6):557-67 doi:10.5271/sjweh.3762

25. Atroshi I, Larsson G-U, Ornstein E et al. Outcomes of endoscopic surgery compared with open surgery for carpal tunnel syndrome among employed patients: randomised controlled trial. *BMJ* 2006;332(7556):1473 doi:10.1136/bmj.38863.632789.1F.

26. Chaise F, Bellemere P, Fril JP et al. Return-to-work interval and surgery for carpal tunnel syndrome. Results of a prospective series of 233 patients. *J Hand Surg Br* 2004;29(6):568-70

27. Atroshi I, Zhou C, Joud A et al. Sickness absence from work among persons with new physician-diagnosed carpal tunnel syndrome: a population-based matched-cohort study. *PLoS One* 2015;10(3):e0119795 doi:10.1371/journal.pone.0119795.

28. Bekkelund SI, Pierre-Jerome C, Torbergsen T et al. Impact of occupational variables in carpal tunnel syndrome. *Acta Neurol Scand* 2001;103(3):193-97

29. Cancelliere C, Donovan J, Stochkendahl MJ et al. Factors affecting return to work after injury or illness: best evidence synthesis of systematic reviews. *Chiropr Man Ther* 2016;24(1):32-32 doi:10.1186/s12998-016-0113-z.

30. Claréus B, Renström EA. Patients' return-to-work expectancy relates to their beliefs about their physician's opinion regarding return to work volition and ability. *J Pain Res* 2019;12:353-62 doi:10.2147/JPR.S179061.

31. Heymans MW, de Vet HC, Knol DL et al. Workers' beliefs and expectations affect return to work over 12 months. *Journal Occup Rehabil* 2006;16(4):685-95 doi:10.1007/s10926-006-9058-8.

32. Bland JDP. Treatment of carpal tunnel syndrome. *Muscle Nerve* 2007;36(2):167-71 doi:10.1002/mus.20802.

33. Louie D, Earp B, Blazar P. Long-term outcomes of carpal tunnel release: a critical review of the literature. *Hand* 2012;7(3):242-46 doi:10.1007/s11552-012-9429-x.

For peer review only - http://bmjopen.bmj.com/site/about/guidelines.xhtml

34. Dale AM, Zeringue A, Harris-Adamson C et al. General population job exposure matrix applied to a pooled study of prevalent carpal tunnel syndrome. *Am Journal Epidemiol* 2015;181(6):431-39

Conflicts of interest

LN was supported by a National Institute for Health Research (NIHR) Doctoral Research Fellowship, grant number DRF-2015-08-056. GN was supported by funding from the MRC Versus Arthritis Centre for Musculoskeletal Health and Work. The authors declare no other conflicts of interest.

Funding

LN was supported by a National Institute for Health Research (NIHR) Doctoral Research Fellowship, grant number DRF-2015-08-056. GN was supported by funding from the MRC Versus Arthritis Centre for Musculoskeletal Health and Work. The views expressed are those of the authors and not necessarily those of the funders, the NHS, the NIHR or the Department of Health.

Informed consent

Written informed consent was obtained from the patient(s) for their anonymised information to be published in this article.

Ethics approval

Full ethics approval was granted by the NHS Health Research Authority (IRAS 209840: 16/WA/0390) and University of Southampton (ERGO 25757) Ethics Committees.

For peer review only

Contributorship

The study was devised by LN, KWB, JA, DW and DC. LN completed data collection and analysis, with assistance from GN and KWB. All authors contributed to the interpretation of the data. LN wrote the first draft of the manuscript. All authors reviewed and edited the manuscript and approved the final version.

Acknowledgements

The authors would like to thank and acknowledge all the study participants who gave up their time to take part in this study. Thank you also to the Patient Advisory Group for their contribution to the development of the research question and study material, and their thoughtful feedback on the preliminary findings. Thank you to Prof David Coggon for his assistance with the initial research idea.

Thank you to the study sites and local principal investigators: Jeremy Bland at East Kent Hospitals University NHS Foundation Trust; Darren Roberts at Portsmouth Hospitals NHS Trust; Mike Taylor and Claire Zweifel at Mid Essex Hospital Services NHS Trust; Frances Clark at Tollgate Clinic; Will Mason at Gloucester Hospitals NHS Foundation Trust; Vasileios Kefalas and Petros Mikalef at Care UK Southampton NHS Treatment Centre; Peter Sharpe at Salisbury Medical Practice; David Warwick at University Hospital Southampton NHS Foundation Trust and Nuffield Health Wessex Hospital; Alistair Phillips at Southern Health NHS Foundation Trust; Nanda Pillai at St Luke's Surgery, Walsall; Tim Halsey and Effie Katsarma at Chelsea and Westminster Hospital NHS Foundation Trust; Claire Middleton at Royal Berkshire NHS Foundation Trust; Duncan Avis and Ian MacLeod at Hampshire Hospital NHS Foundation Trust; Nick Gape and Lisa Small at Cardiff and Vale University Health Board; Kristin Francis at HCA

For peer review only

Healthcare London Hand and Wrist Unit; and to all local team members who helped set up and recruit to the REACTS study.

For peer review only

For peer review only - http://bmjopen.bmj.com/site/about/guidelines.xhtml

Figure 1. Participant numbers for each stage of the cohort study

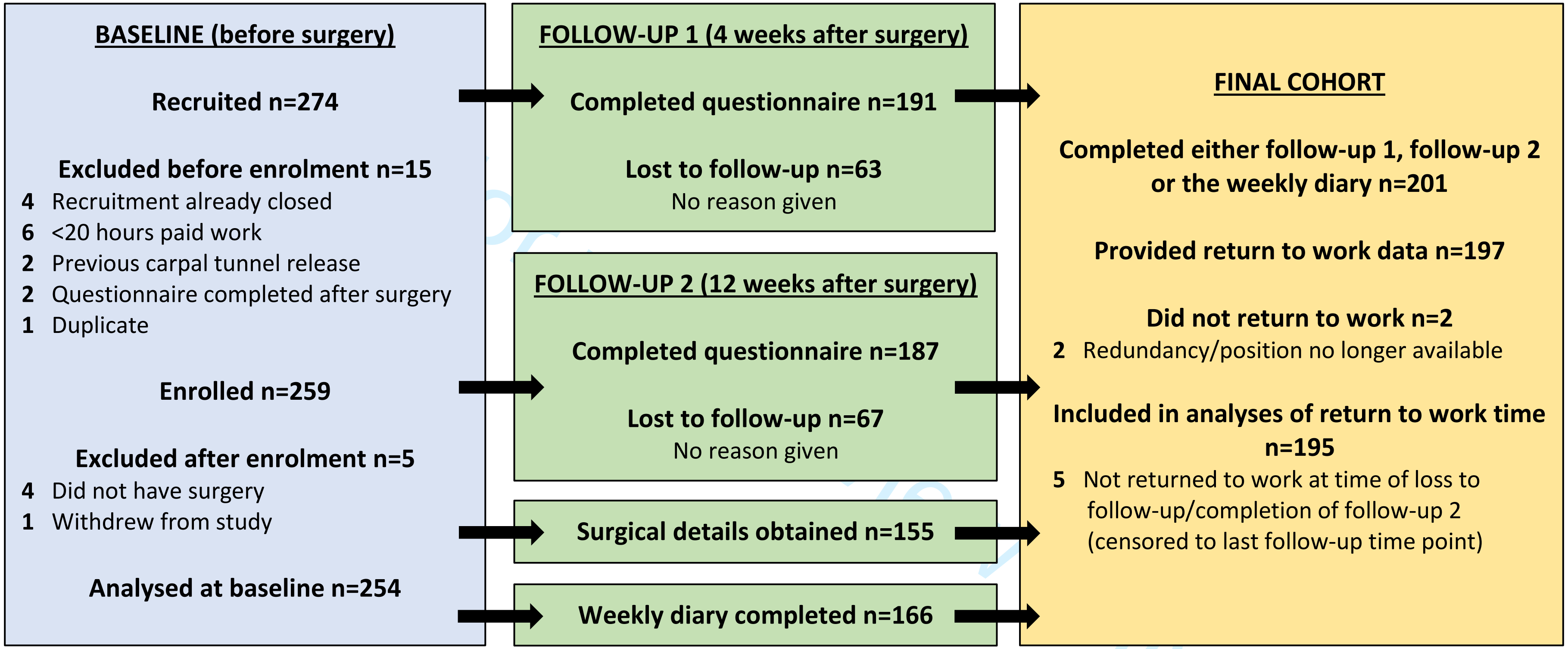

Figure 2. Duration of work absence after carpal tunnel release

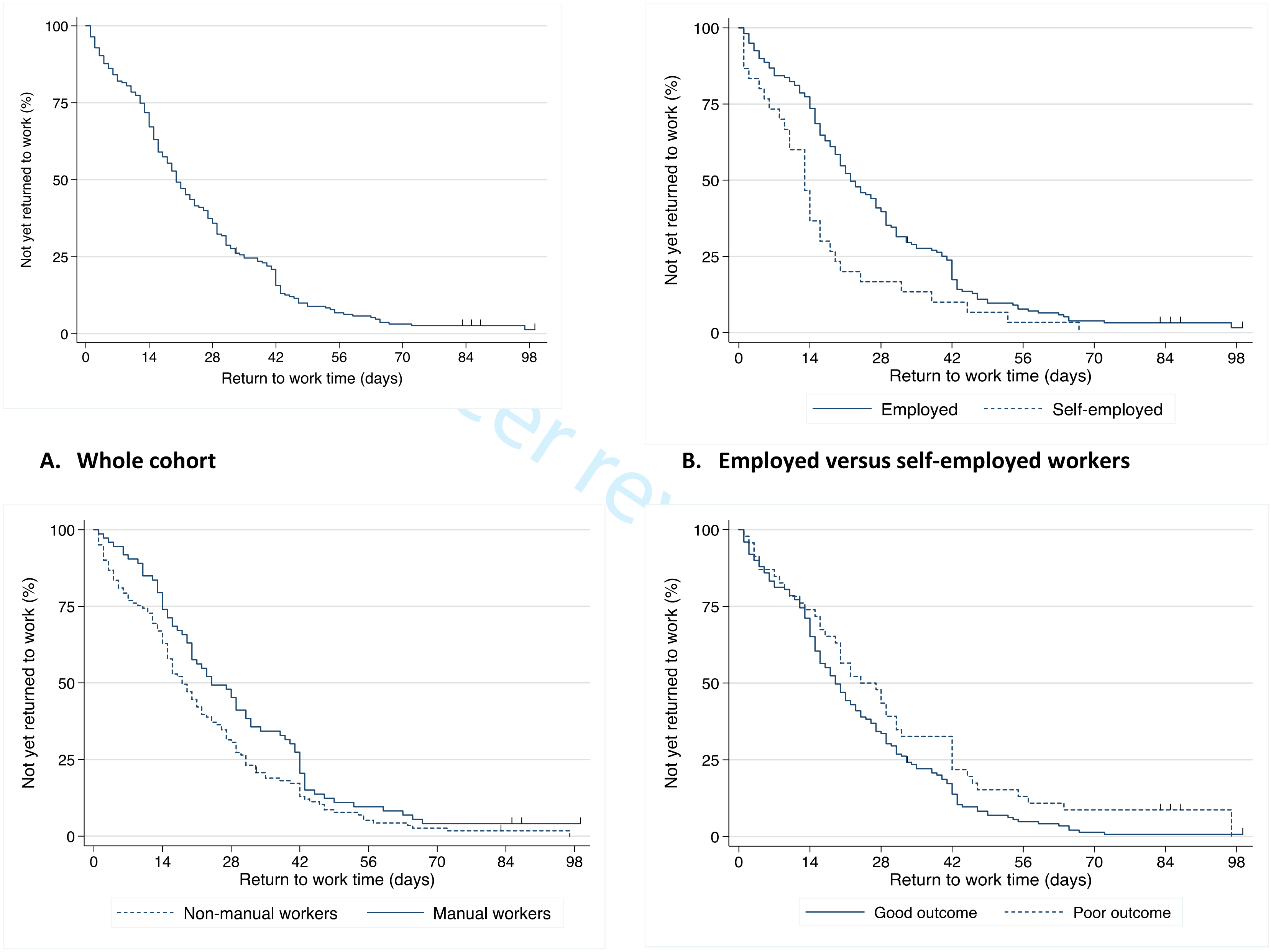

A. Whole cohort

B. Employed versus self-employed workers

C. Non-manual versus manual workers

D. With versus without a poor outcome

Vertical lines indicate right censoring for those who had not returned to work at the point of last follow-up

University Hospital Southampton NHS
NHS Foundation Trust

REACTS

Arthritis Research UK | MRC Medical Research Council | centre for musculoskeletal health & work

UNIVERSITY OF Southampton

Site | | | |

Please fill in today's date | | | | | | |
d d m m y y

Before Your Carpal Tunnel Release Surgery

REACTS

Return to Employment After Carpal Tunnel Release Surgery (REACTS)

ln@mrc.soton.ac.uk | 023 8077 7624
Arthritis Research UK – MRC Centre for Musculoskeletal Health and Work
MRC Lifecourse Epidemiology Unit, University of Southampton
Southampton General Hosptial (MP 95), SO16 6YD

IRAS reference: 209840

For peer review only

University Hospital Southampton NHS
NHS Foundation Trust

REACTS

Arthritis Research UK MRC Medical Research Council centre for musculoskeletal health & work

UNIVERSITY OF Southampton

CONSENT FORM (IRAS reference: 209840)

You should complete this form after you have read the Participant Information Sheet.

REACTS: Return to employment after carpal tunnel release surgery

Thank you for considering taking part in this research. If you have any questions arising from the Participant Information Sheet, please ask the research team before you decide whether to take part.

Please <u>initial</u> the boxes if you agree with each statement

1. I have read the Participant Information Sheet (version 2.0; 06.12.16) and have had the opportunity to ask questions about the study. ☐

2. I meet the criteria for being involved in this study: ☐
   - Aged over 18 and referred for carpal tunnel release surgery
   - Routinely work in paid employment for at least 20 hours per week
   - Plan to return to work after carpal tunnel release surgery
   - Have not previously had carpal tunnel release surgery on either hand
   - Have not previously had a serious injury to the same wrist/hand that will have the carpal tunnel release operation

3. I agree to take part in this research and agree for my data to be used for the purposes explained in the Participant Information Sheet (version 2.0; 06.12.16). I understand that this information will be handled in accordance with the terms of the UK Data Protection Act 1998. ☐
   a. I agree for the REACTS research team to access pre-operative test results concerning my hand and wrist symptoms. No other information will be accessed. ☐
   b. I agree for the REACTS research team to access my carpal tunnel release surgical record. No other information will be accessed. ☐

4. I understand that if I decide at any time during the research that I no longer wish to take part, I can notify the researchers and withdraw from the study immediately, without giving a reason. If I do, I understand that I can ask for any contribution I have already made to be removed from the study, up to the time when I have completed the final questionnaire. ☐

Signature ______________________________ Date ______ / ______ / ______

Name ______________________________ Phone ______________________
(please print) *(only to be used if we lose touch)*

Postal address ____

____

Email address ____
(please print)

For peer review only

For peer review only - http://bmjopen.bmj.com/site/about/guidelines.xhtml

ADDITIONAL QUESTIONS

Please circle one response for each question

I prefer to receive the next two questionnaires by	**Post**	**Email**	**Don't mind**
I prefer to receive correspondence about the study by	**Post**	**Email**	**Don't mind**
I would like to be notified of the findings from this research	**Yes**	**No**	
I am happy to be contacted about the next stage of the research, which will involve a one-off discussion with the lead researcher	**Yes**	**No**	
I am happy to be contacted about other studies related to this research	**Yes**	**No**	

When the research team receives your completed questionnaire and consent form, we will sign it below and return a copy to you for your records.

Researcher signature ______________________________________ Date ____ / ____ / ____

Researcher name ______________________________________

University of Southampton research supervisors:
Professor Karen Walker-Bone | Professor Jo Adams | Professor David Warwick

For peer review only

SECTION A: BACKGROUND

1 What is your date of birth?

d	d	m	m	y	y

2 Are you:

Male ☐ Female ☐ Other ☐

3 Are you:

Right handed ☐ Left handed ☐ Both ☐

4 Do you routinely carry out paid work for 20 hours or longer in a given week?

Yes ☐ No ☐

If no, *thank you for your interest in our study, however, we are only looking for individuals who carry out paid work for at least 20 hours per week. You* ***do not*** *need to complete the rest of the questionnaire, but please return it using the pre-paid envelope provided.*

5 When do you expect to have your carpal tunnel surgery?
Please enter the exact date if known, or provide the approximate month and year if unsure.

d	d	m	m	y	y

6 Which hand will be operated on?
If both hands please answer Question 6.1; if one hand, please move on to Question 7.

Right ☐ Left ☐ Both ☐

6.1 If both hands, which side will be operated on first?

Right ☐ Left ☐ Both sides operated on the same day ☐ Unsure ☐

7 Do you have access to an occupational health service through your place of work?

Yes ☐ No ☐ Unsure ☐

8 Do you expect to take any time off work following your surgery?
If yes, please answer Question 8.1; if no, please move on to Question 9.

Yes ☐ No ☐ Unsure ☐

8.1 If you do expect to take time off work, how long do you expect to take?
Please complete using days, weeks or months; whichever applies.

Days ☐ Weeks ☐ Months ☐

For peer review only - http://bmjopen.bmj.com/site/about/guidelines.xhtml

SECTION A: BACKGROUND

9 Have you been given any information about your operation?
If yes, please answer Question 9.1; if no, please move on to Question 10.

Yes ☐ No ☐

9.1 If yes, who provided this information? Please tick all that apply.

a) Your surgeon or a member of the surgical team ☐
b) Hospital nurse ☐
c) GP or practice nurse ☐
d) Hand therapist ☐
e) Physiotherapist or occupational therapist ☐
f) Occupational health nurse or doctor ☐
g) Employer ☐
h) Friend or family member ☐
i) Internet ☐
j) Other *(please specify)* ☐

....

10 Have you been given any information about returning to work after your surgery?
If yes, please answer the rest of Question 10; if no, please move on to Question 11.

Yes ☐ No ☐

10.1 If yes, who provided this information? Please tick all that apply.

a) Your surgeon or a member of the surgical team ☐
b) Hospital nurse ☐
c) GP or practice nurse ☐
d) Hand therapist ☐
e) Physiotherapist or occupational therapist ☐
f) Occupational health nurse or doctor ☐
g) Employer ☐
h) Friend or family member ☐
i) Internet ☐
j) Other *(please specify)* ☐

....

10.2 What advice were you given?
If this advice came from more than one source, please indicate who advised what.

....
....
....
....
....
....
....

For peer review only - http://bmjopen.bmj.com/site/about/guidelines.xhtml

SECTION B: WORK

12 What is your MAIN occupation at the moment (e.g. secretary, teacher, builder etc.)?

----

13 And in what industry do you work (e.g. farming, shipyard, car factory, shoe shop, hospital, insurance office etc)?

----

14 Which of the following best describes your present work situation for your MAIN occupation? Please tick one box.

a) Employed (permanent contract) ☐

b) Employed (temporary/renewable contract) ☐

c) Zero hours contract ☐

d) Self-employed ☐

e) Other *(please specify)* ☐

----

15 On average, how many *hours* per week do you normally work in your main occupation?

☐☐☐ hours

16 On average, how many *days* per week do you normally work in your main occupation?

☐ days

17 Do you have any other paid work?
If yes, please answer Question 17.1; if no, please move on to Question 18.

Yes ☐ No ☐

17.1 If yes, on average, how many hours a week do you work in other paid jobs? ☐☐ hours

18 Does an average day at work in your MAIN job normally involve any of the following? Please tick one box for each question.

		Yes	No
a)	Piecework in which you are paid according to the number of articles or tasks you or your team make or finish in the day?	☐	☐
b)	A target number of articles or tasks that you or your team are expected to make or finish in the day?	☐	☐
c)	Payment of a bonus if you make or finish more than an agreed number of articles/tasks in the day?	☐	☐
d)	Working to tight deadlines	☐	☐
e)	Use of a computer keyboard or mouse for longer than 1 hour in total?	☐	☐

For peer review only - http://bmjopen.bmj.com/site/about/guidelines.xhtml

SECTION B: WORK

		Yes	No
f)	Use of a computer keyboard or mouse for longer than 4 hours in total?	☐	☐
g)	Other tasks involving repeated movements of the wrist or fingers for longer than 4 hours in total? *(Please indicate which tasks)*	☐	☐

		Yes	No
h)	Working with a powered tool that makes your hand(s) or arm(s) vibrate (e.g. chain saw, pneumatic drill)?	☐	☐
i)	Working with your hand(s) above shoulder height for longer than 1 hour in total?	☐	☐
j)	Lifting or carrying weights of 5 kg (11 lbs) or more in one hand (e.g. a tool bag or heavy briefcase)?	☐	☐
k)	Lifting or carrying a weight of 10 kg (22 lbs) or more?	☐	☐
l)	Tasks involving pushing or pulling a heavy weight?	☐	☐
m)	Working for longer than two hours in total with your neck bent forward?	☐	☐
n)	Working for longer than half an hour in total with your neck twisted e.g. when looking to one side?	☐	☐
o)	Driving for more than an hour?	☐	☐

19 Do you find your MAIN job demanding on your hands/wrists?
Please circle one number, where 0 represents not at all, and 10 represents very much.

0 1 2 3 4 5 6 7 8 9 10

Not at all Very much

20 Does your MAIN employer (or boss/colleagues if self-employed) know about your hand/wrist problem?
If yes, please answer Question 20.1; if no, or not applicable, please move on to Question 21.

Yes ☐ No ☐ N/A self-employed and work alone ☐

20.1 Is your MAIN employer (or boss/colleagues if self-employed) supportive of your hand/wrist problem?
Please circle one number, where 0 represents not at all, and 10 represents very much

0 1 2 3 4 5 6 7 8 9 10

Not at all Very much

For peer review only - http://bmjopen.bmj.com/site/about/guidelines.xhtml

SECTION B: WORK

21 **The following questions refer to how you did in your MAIN job during the *past 4 weeks*.**
Please tick one box for each question.

How much of the time during the past 4 weeks …	**Always**	**Often**	**Sometimes**	**Rarely**	**Never**
a) Were you unable to do your work because of problems with your hand(s) / wrist(s)?	☐	☐	☐	☐	☐
b) Did you have to shorten your work day because of problems with your hand(s) / wrists(s)	☐	☐	☐	☐	☐
c) Did you have to take breaks at work because of problems with your hand(s) / wrists(s)?	☐	☐	☐	☐	☐
d) Did you get less done because of problems with your hand(s) / wrist(s)?	☐	☐	☐	☐	☐
e) Did you take longer to do the tasks in your work because of problems with your hand(s) / wrists(s)?	☐	☐	☐	☐	☐

22 **During the *past 4 weeks*, how much time have you missed from your MAIN job for the following reasons?**
Please write 0 if you have not missed any time from work during this period. You can answer in days or hours, whichever applies.

a) Time missed because of the problem with your hand(s)/wrist(s) ☐☐ Days ***or*** ☐☐ Hours

b) Time missed because of any other problem ☐☐ Days ***or*** ☐☐ Hours

23 **If you fell ill and were off work, how long could you get your normal full pay (excluding bonuses)?**
Please tick the option that best represents your MAIN job.

a) Less than one week ☐
b) 1 – 4 weeks ☐
c) 1 – 6 months ☐
d) More than 6 months ☐
e) Not sure ☐

24 **How satisfied are you with your MAIN job as a whole, taking everything into consideration?** This includes your salary, career possibilities, management, colleagues etc. Please tick one box.

a) Very satisfied ☐
b) Satisfied/fairly satisfied ☐
c) Dissatisfied ☐
d) Very dissatisfied ☐

For peer review only - http://bmjopen.bmj.com/site/about/guidelines.xhtml

SECTION C: GENERAL HEALTH

25 In general, would you say your health is:

a) Excellent ☐ d) Fair ☐

b) Very good ☐ e) Poor ☐

c) Good ☐

26 What is your height? Please answer in either feet and inches or centimetres.

☐ feet ☐☐ inches ***or*** ☐☐☐ cms

27 What is your weight? Please answer in either stones and pounds or kilograms.

☐☐ stones ☐☐ lbs ***or*** ☐☐ kgs

28 Do you, or have you ever, smoked regularly? Please tick one box.

a) I have never smoked regularly ☐ c) I regularly smoke ☐

b) I have smoked in the past, but do not currently smoke regularly ☐

29 The following is a list of common health problems. Please indicate if you currently have, or don't have, the problem listed in part 1. If you have the problem, please answer the corresponding question in part 2.

Please answer all questions in part 1.

	HEALTH PROBLEM	PART 1 Do you have the problem? NO	YES	PART 2 Does it limit your activities? NO	YES
			(if yes move to part 2) →		
a)	Heart disease	☐	☐	☐	☐
b)	High blood pressure	☐	☐	☐	☐
c)	Lung disease	☐	☐	☐	☐
d)	Diabetes	☐	☐	☐	☐
e)	Ulcer or stomach disease	☐	☐	☐	☐
f)	Kidney disease	☐	☐	☐	☐
g)	Liver disease	☐	☐	☐	☐
h)	Thyroid disease	☐	☐	☐	☐
i)	Anaemia or other blood disease	☐	☐	☐	☐
j)	Cancer	☐	☐	☐	☐

For peer review only - http://bmjopen.bmj.com/site/about/guidelines.xhtml

SECTION C: GENERAL HEALTH

HEALTH PROBLEM continued…	Do you have the problem? NO	YES (if yes move to part 2) →	Does it limit your activities? NO	YES
k) Depression	☐	☐	☐	☐
l) Osteoarthritis	☐	☐	☐	☐
m) Back pain	☐	☐	☐	☐
n) Rheumatoid arthritis	☐	☐	☐	☐

29.1 Please list any other medical problems that have not been mentioned.

	Does it limit your activities? NO	YES
o)	☐	☐
p)	☐	☐
q)	☐	☐

30 The following questions are about how you feel and how things have been with you during the *past 4 weeks*. For each question, please give the answer that comes closest to the way you have been feeling. Please tick one box for each row.

How much of the time during the past 4 weeks …	All of the time	Most of the time	A good bit of the time	Some of the time	A little bit of the time	None of the time
a) Did you feel full of 'get-up-and-go'?	☐	☐	☐	☐	☐	☐
b) Have you been a very nervous person?	☐	☐	☐	☐	☐	☐
c) Have you felt so down in the dumps that nothing could cheer you up?	☐	☐	☐	☐	☐	☐
d) Have you felt calm and peaceful?	☐	☐	☐	☐	☐	☐
e) Did you have a lot of energy?	☐	☐	☐	☐	☐	☐
f) Have you felt downhearted and blue?	☐	☐	☐	☐	☐	☐
g) Did you feel worn out?	☐	☐	☐	☐	☐	☐
h) Have you been a happy person?	☐	☐	☐	☐	☐	☐
i) Did you feel tired?	☐	☐	☐	☐	☐	☐

For peer review only - http://bmjopen.bmj.com/site/about/guidelines.xhtml

SECTION C: GENERAL HEALTH

31 **Below is a list of problems that people sometimes have. Please read each one carefully and tick the box that best describes how much that problem has distressed or bothered you during the *past 7 days*, including today?**
Please tick one box for each row.

	Not at all	A little bit	Moderately	Quite a bit	Extremely
a) Faintness or dizziness	☐	☐	☐	☐	☐
b) Pains in the heart or chest	☐	☐	☐	☐	☐
c) Nausea or upset stomach	☐	☐	☐	☐	☐
d) Trouble getting your breath	☐	☐	☐	☐	☐
e) Hot or cold spells	☐	☐	☐	☐	☐

For peer review only

For peer review only - http://bmjopen.bmj.com/site/about/guidelines.xhtml

SECTION D: HAND AND WRIST FUNCTION

32 **In the *past 7 days*, have you experienced any pain, tingling (pins and needles) or numbness (loss of sensation) in your RIGHT hand or wrist?**
Please mark where on your hand/wrist you experienced these symptoms using the key below.
If you do not have any symptoms in your right hand, please move on to Question 34.

Pain

Tingling or numbness

RIGHT HAND

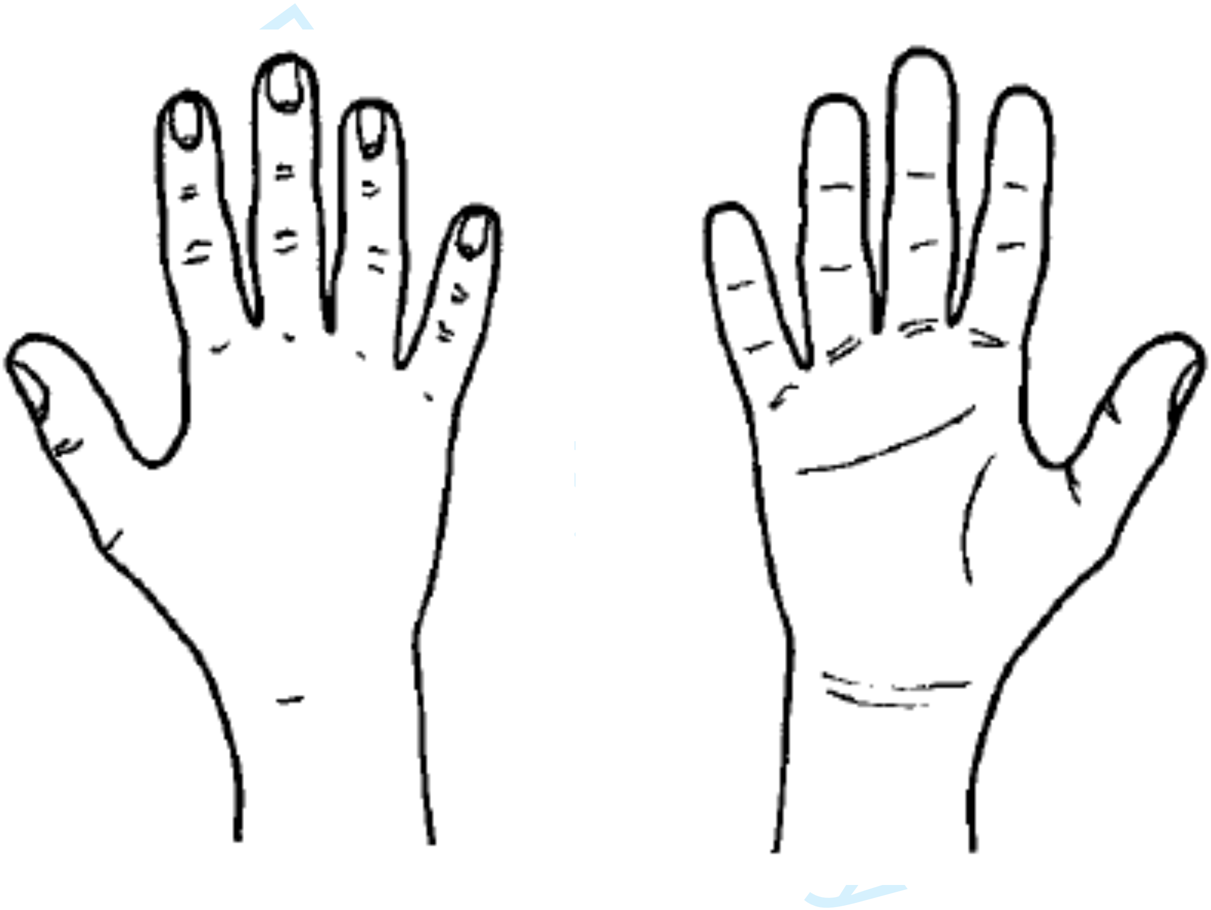

33 How long ago did the first of these symptoms begin? Please tick one box.

a) Less than 3 months ☐
b) 3 – 6 months ☐
c) 6 – 12 months ☐
d) More than a year ☐

For peer review only - http://bmjopen.bmj.com/site/about/guidelines.xhtml

SECTION D: HAND AND WRIST FUNCTION

34 **In the *past 7 days*, have you experienced any pain, tingling (pins and needles) or numbness (loss of sensation) in your LEFT hand or wrist?**
Please mark where on your hand/wrist you experienced these symptoms using the key below.
If you do not have any symptoms in your right hand, please move on to Question 36.

☐ Pain ☐ Tingling or numbness

LEFT HAND

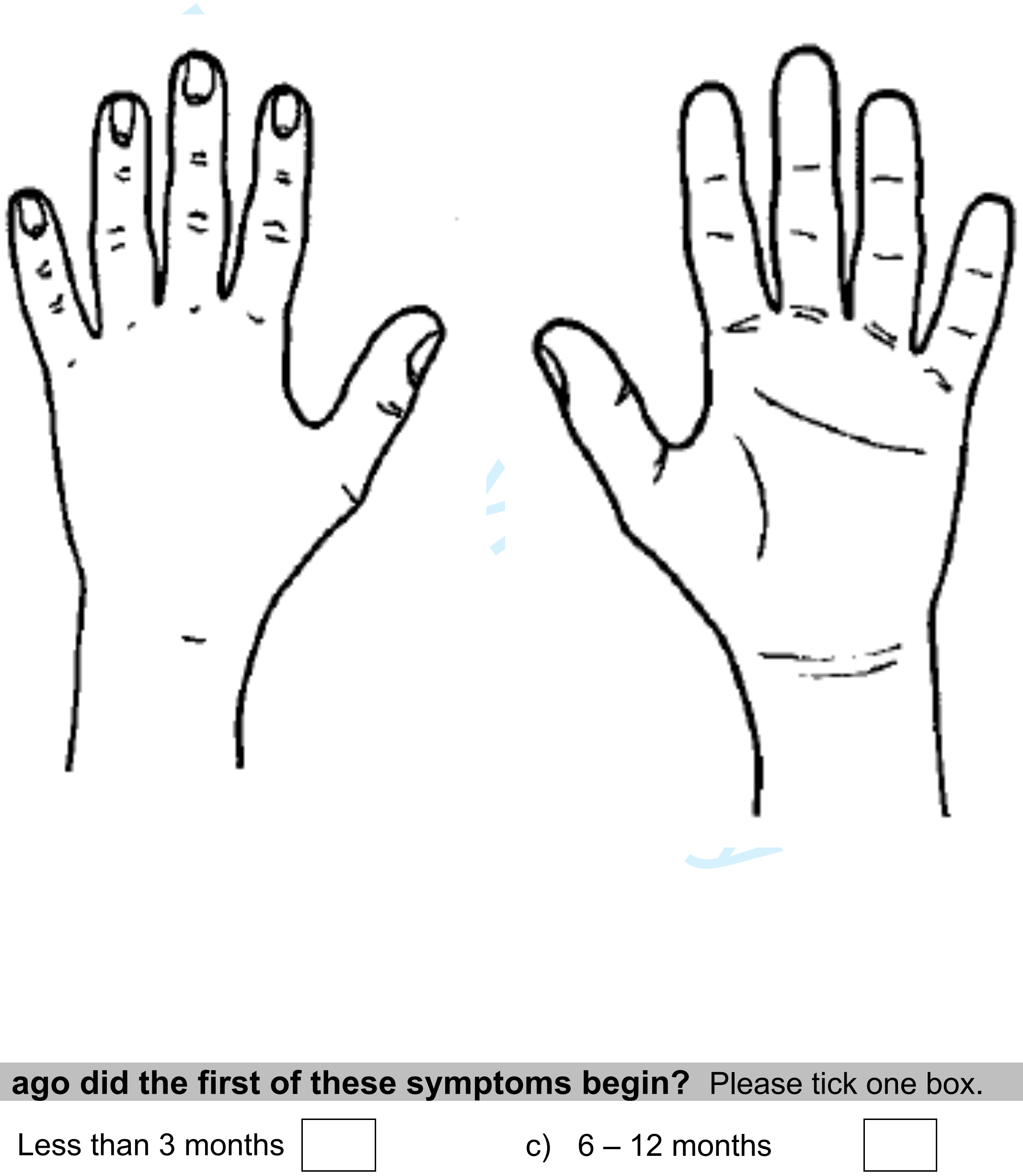

35 How long ago did the first of these symptoms begin? Please tick one box.

a) Less than 3 months ☐ c) 6 – 12 months ☐

b) 3 – 6 months ☐ d) More than a year ☐

For peer review only - http://bmjopen.bmj.com/site/about/guidelines.xhtml

SECTION D: HAND AND WRIST FUNCTION

36 **The following questions refer to your symptoms over the *last 7 days*.**
Please answer for each hand, even if you only have problems with one side.
Please tick one box for each row.

**36.1 How severe were the following symptoms in your RIGHT hand?**	**None**	**Mild**	**Moderate**	**Severe**	**Very severe**
a) Pain at night	☐	☐	☐	☐	☐
b) Pain during the daytime	☐	☐	☐	☐	☐
c) Numbness or tingling at night	☐	☐	☐	☐	☐
d) Numbness or tingling during the daytime	☐	☐	☐	☐	☐

**How often did the following symptoms in your RIGHT hand wake you up at night?**	**Never**	**Once**	**2 or 3 times**	**4 or 5 times**	**More than 5 times**
e) Pain	☐	☐	☐	☐	☐
f) Numbness or tingling	☐	☐	☐	☐	☐

**36.2 How severe were the following symptoms in your LEFT hand?**	**None**	**Mild**	**Moderate**	**Severe**	**Very severe**
a) Pain at night	☐	☐	☐	☐	☐
b) Pain during the daytime	☐	☐	☐	☐	☐
c) Numbness or tingling at night	☐	☐	☐	☐	☐
d) Numbness or tingling during the daytime	☐	☐	☐	☐	☐

**How often did the following symptoms in your LEFT hand wake you up at night?**	**Never**	**Once**	**2 or 3 times**	**4 or 5 times**	**More than 5 times**
e) Pain	☐	☐	☐	☐	☐
f) Numbness or tingling	☐	☐	☐	☐	☐

37 **This question refers to the appearance (look) of your hand during the *past 7 days*.** Please tick one box for each hand.

	**Strongly agree**	**Agree**	**Neither agree nor disagree**	**Disagree**	**Strongly disagree**
a) I am satisfied with the appearance (look) of my **RIGHT** hand	☐	☐	☐	☐	☐
b) I am satisfied with the appearance (look) of my **LEFT** hand	☐	☐	☐	☐	☐

For peer review only

SECTION D: HAND AND WRIST FUNCTION

38 Please answer the following questions on a scale of 0-10, where 0 represents not at all, and 10 represents very much. Please circle one number for each question.

38.1 Do you think that you will be able to use your hand normally 3 months after the operation?

0 1 2 3 4 5 6 7 8 9 10

38.2 Are you afraid of having long-term problems with your hand?

0 1 2 3 4 5 6 7 8 9 10

38.3 Do you blame yourself for your hand problem?

0 1 2 3 4 5 6 7 8 9 10

38.4 Are your family and friends supportive of your hand problem?

0 1 2 3 4 5 6 7 8 9 10

39 The following statements describe people's beliefs about their health problems. Please indicate whether you agree or disagree with them in relation to the problems you have with your hand(s) or wrist(s).
Please tick the box which most closely reflects how you feel for each statement.

	**Strongly agree**	**Agree**	**Neither agree or disagree**	**Disagree**	**Strongly Disagree**
a) Problems like this run in my family	☐	☐	☐	☐	☐
b) I think I was born with a weakness or underlying problem in this part of my body	☐	☐	☐	☐	☐
c) My problem was caused by work	☐	☐	☐	☐	☐
d) Work probably didn't cause my problem, but it made it worse	☐	☐	☐	☐	☐
e) I have a lot of stress in my life and that has made my problem a lot worse	☐	☐	☐	☐	☐
f) I think a lack of exercise probably contributed to my problem	☐	☐	☐	☐	☐
g) As you get older, parts of the body start to wear out and problems like mine are likely to occur	☐	☐	☐	☐	☐

For peer review only - http://bmjopen.bmj.com/site/about/guidelines.xhtml

SECTION D: HAND AND WRIST FUNCTION

40 We are interested in the types of thoughts and feelings that you have when you are in pain. The following statements describe different thoughts and feelings that may be associated with pain. Please indicate the degree to which you have these thoughts and feelings when you are experiencing pain.
Please tick one box for each statement.

	Not at all	To a slight degree	To a moderate degree	To a great degree	All of the time
a) I keep thinking about how badly I want the pain to stop	☐	☐	☐	☐	☐
b) It's terrible and I think it's never going to get any better	☐	☐	☐	☐	☐
c) I become afraid that the pain may get worse	☐	☐	☐	☐	☐
d) I anxiously want the pain to go away	☐	☐	☐	☐	☐

The following questions refer to the function of your hands/wrists during the ***past 7 days***. Please answer all questions for the right and left sides, even if you do not experience any problems. Please tick one box for each question.

**41 RIGHT SIDE**	**Very well**	**Well**	**Adequately**	**Poorly**	**Very poorly**
a) Overall, how well did your ***right*** hand work?	☐	☐	☐	☐	☐
b) How well did your ***right*** fingers move?	☐	☐	☐	☐	☐
c) How well did your ***right*** wrist move?	☐	☐	☐	☐	☐
	**Very good**	**Good**	**Fair**	**Poor**	**Very poor**
d) How was the strength in your ***right*** hand?	☐	☐	☐	☐	☐
e) How was the sensation (feeling) in your ***right*** hand?	☐	☐	☐	☐	☐

**42 LEFT SIDE**	**Very well**	**Well**	**Adequately**	**Poorly**	**Very poorly**
a) Overall, how well did your ***left*** hand work?	☐	☐	☐	☐	☐
b) How well did your ***left*** fingers move?	☐	☐	☐	☐	☐
c) How well did your ***left*** wrist move?	☐	☐	☐	☐	☐
	**Very good**	**Good**	**Fair**	**Poor**	**Very poor**
d) How was the strength in your ***left*** hand?	☐	☐	☐	☐	☐
e) How was the sensation (feeling) in your ***left*** hand?	☐	☐	☐	☐	☐

For peer review only

SECTION D: HAND AND WRIST FUNCTION

The following questions refer to the ability of your hands to do certain tasks during the ***past 7 days***. If you do not do a certain task, please estimate the difficulty you would have in performing it. Please tick one box for every activity.

43 How difficult was it for you to perform the following activities using your RIGHT HAND?

		Not at all difficult	A little difficult	Somewhat difficult	Moderately difficult	Very difficult
a)	Turn a door knob	☐	☐	☐	☐	☐
b)	Pick up a coin	☐	☐	☐	☐	☐
c)	Hold a glass of water	☐	☐	☐	☐	☐
d)	Turn a key in a lock	☐	☐	☐	☐	☐
e)	Hold a frying pan	☐	☐	☐	☐	☐

44 How difficult was it for you to perform the following activities using your LEFT HAND?

		Not at all difficult	A little difficult	Somewhat difficult	Moderately difficult	Very difficult
a)	Turn a door knob	☐	☐	☐	☐	☐
b)	Pick up a coin	☐	☐	☐	☐	☐
c)	Hold a glass of water	☐	☐	☐	☐	☐
d)	Turn a key in a lock	☐	☐	☐	☐	☐
e)	Hold a frying pan	☐	☐	☐	☐	☐

45 How difficult was it for you to perform the following activities using BOTH HANDS?

		Not at all difficult	A little difficult	Somewhat difficult	Moderately difficult	Very difficult
a)	Open a jar	☐	☐	☐	☐	☐
b)	Button a shirt/blouse	☐	☐	☐	☐	☐
c)	Eat with a knife/fork	☐	☐	☐	☐	☐
d)	Carry a grocery bag	☐	☐	☐	☐	☐
e)	Wash dishes	☐	☐	☐	☐	☐
f)	Wash your hair	☐	☐	☐	☐	☐
g)	Tie shoelaces/knots	☐	☐	☐	☐	☐

For peer review only - http://bmjopen.bmj.com/site/about/guidelines.xhtml

SECTION D: HAND AND WRIST FUNCTION

The following questions refer to your satisfaction with your hands/wrists during the ***past 7 days***. Please tick one box for each question

46 How satisfied were you with your RIGHT hand/wrist during the *past 7 days*?

	RIGHT HAND	Very satisfied	Somewhat satisfied	Neither satisfied or dissatisfied	Somewhat dissatisfied	Very dissatisfied
a)	Overall function of your hand					
b)	Movement of the fingers					
c)	Movement of your wrist					
d)	Strength of your hand					
e)	Pain level of your hand					
f)	Sensation (feeling) of your hand					

47 How satisfied were you with your LEFT hand/wrist during the *past 7 days*?

	LEFT HAND	Very satisfied	Somewhat satisfied	Neither satisfied or dissatisfied	Somewhat dissatisfied	Very dissatisfied
a)	Overall function of your hand					
b)	Movement of the fingers					
c)	Movement of your wrist					
d)	Strength of your hand					
e)	Pain level of your hand					
f)	Sensation (feeling) of your hand					

For peer review only

Thank you for completing this questionnaire!
Please return it to the REACTS team
using the pre-paid envelope.

REACTS

If you have any questions, or would like any additional information, please contact Lisa Newington on:
ln@mrc.soton.ac.uk | 023 8077 7624 | 07866 997732

For peer review only - http://bmjopen.bmj.com/site/about/guidelines.xhtml

REACTS ID:

One Month After Your Carpal Tunnel Release Surgery

Return to Employment After Carpal Tunnel Release Surgery (REACTS)

ln@mrc.soton.ac.uk | 023 8077 7624
Arthritis Research UK – MRC Centre for Musculoskeletal Health and Work
MRC Lifecourse Epidemiology Unit, University of Southampton
Southampton General Hosptial (MP 95), SO16 6YD

IRAS reference: 209840

For peer review only - http://bmjopen.bmj.com/site/about/guidelines.xhtml

SECTION A: ABOUT YOUR OPERATION

Please fill in today's date ☐☐☐☐☐☐☐☐
d d m m y y y y

1 What was the date of your carpal tunnel release surgery? ☐☐☐☐☐☐☐☐
d d m m y y y y

2 Which side was operated on? Please tick one box.

Right ☐ Left ☐ Both ☐

3 What type of anaesthetic did you have? Please tick one box.

a) General anaesthetic (you were sent to sleep) ☐

b) Local or regional anaesthetic (your arm was made numb, but you were still awake) ☐

c) Other *(please specify)* ☐

---

d) Unsure ☐

4 How long did you need to stay in the hospital/clinic after your operation?
Please tick one box (and specify the number of nights, if applicable).

a) I went home the same day ☐

b) I needed to stay overnight (one night only) ☐

c) I needed to stay for more than one night ☐

(Please specify for how long) ☐☐ nights

For peer review only

SECTION A: ABOUT YOUR OPERATION

5 Have you used any of the following services specifically for your operated hand(s) since your surgery?
Please give the number of visits for each service, and the date(s) attended, if known.

	I used this service in the NHS		I used this service privately	
	Number of visits	Dates attended, if known	Number of visits	Dates attended, if known
a) Your surgeon, or one of the surgical team				
b) GP or practice nurse				
c) Hospital nurse				
d) Pharmacist				
e) Hand therapist				
f) Other physiotherapist or occupational therapist				
g) Chiropractor or osteopath				
h) Occupational health nurse or doctor				
i) Accident and emergency (A&E) or minor injuries unit				
j) Other (*please specify*)				

For peer review only - http://bmjopen.bmj.com/site/about/guidelines.xhtml

SECTION A: ABOUT YOUR OPERATION

6 Have you taken any antibiotics for an infection in your surgical wound?
Please do not include any antibiotics you were prescribed at the time of your operation.

Yes ☐ No ☐

If yes, what date did you start taking the antibiotics? d d m m y y y y

7 Have you been admitted to hospital because of a problem with your operated hand(s)? If yes, please answer the rest of Question 7; if no, please move on to Question 8.

Yes ☐ No ☐

7.1 If yes, when were you first admitted? d d m m y y y y

7.2 How many nights did you stay in hospital?
Please answer 0 if you didn't stay overnight.
☐☐ nights

7.3 Did you require another operation? Yes ☐ No ☐

8 Have you been advised that you may need a carpal tunnel release for your other hand in the future?
If yes, please answer Question 8.1; if no, please move on to Question 9.

Yes ☐ No ☐

8.1 If yes, when are you expecting to have this surgery? Please tick one box.

a) In less than 2 months ☐
b) In 2-5 months ☐
c) In 6-11 months ☐
d) In more than a year ☐
e) Unsure ☐

9 If you would like to give us any other information about your operation, or the healthcare services you have used, please do so here:

For peer review only - http://bmjopen.bmj.com/site/about/guidelines.xhtml

SECTION B: WORK

10 Compared to before your surgery, which of the following best describes your current work situation? Please tick one box.

a) Returned to the same job, work duties and hours – **please go to Question 14** ☐

b) Returned to the same job, with altered duties or hours – **please go to Question 14** ☐

c) Started a new job – **please go to Question 11** ☐

d) Not yet returned to work, but plan to return in the future – **please go to Question 12** ☐

e) Do not plan to return to work – **please go to Question 13** ☐

11 Thinking about your new job:

11.1 What is your main occupation now (e.g. secretary, teacher, builder etc.)?

11.2 In what industry do you work (e.g. farming, shipyard, car factory, shoe shop, hospital, insurance office etc.)?

11.3 Did you change jobs because of your hand/wrist problem?
Please tick one box.

a) Yes, my hand/wrist problem was the main reason for my job change ☐

b) Yes, my hand/wrist problem was one of several reasons for my job change ☐

c) No, my job change was nothing to do with my hand/wrist problem ☐

d) Other, please specify ☐

Please go to Question 14

For peer review only

For peer review only - http://bmjopen.bmj.com/site/about/guidelines.xhtml

SECTION B: WORK

12 If you have not yet returned to work, when do you think you might be able to return? Please give an estimated date if you are unsure.

d	d	m	m	y	y	y	y

12.1 Have you discussed when to return to work with anyone?
If yes, please answer the rest of Question 12; if no, please move on to Question 21.

Yes ☐ No ☐

12.2 If yes, who have you discussed this with? Please tick all that apply.

a) Your surgeon or a member of the surgical team ☐
b) Hospital nurse ☐
c) GP or practice nurse ☐
d) Hand therapist ☐
e) Physiotherapist or occupational therapist ☐
f) Occupational health nurse or doctor ☐
g) Employer or manager (or colleagues if self-employed ☐
h) Friend or family member ☐
i) Other *(please specify)* ☐

12.3 Since your operation, have you been given any specific advice about when and how to return to work? This could include any activities to avoid or timescales to follow. Please list any advice here, including who gave you this advice:

Please go to Question 21

For peer review only

SECTION B: WORK

13 If you do not plan to return to work, what is the main reason for this decision?

Please tick one box.

a) Retirement ☐

b) Redundancy ☐

c) Position/work no longer available ☐

d) Unable to do your work because of your problem with your hand(s)/wrist(s) ☐

e) Unable to do your work because of any other problem ☐

f) Other *(please specify)* ☐

....

....

....

....

13.1 Have you been advised not to return to work by anyone?

If yes, please answer Question 13.2; if no, please move on to Question 21.

Yes ☐ No ☐

13.2 If yes, who by? Please tick all that apply.

a) Your surgeon or a member of the surgical team ☐

b) Hospital nurse ☐

c) GP or practice nurse ☐

d) Hand therapist ☐

e) Physiotherapist or occupational therapist ☐

f) Occupational health nurse or doctor ☐

g) Employer or manager (or colleagues if self-employed) ☐

h) Friend or family member ☐

i) Other *(please specify)* ☐

....

....

Please go to Question 21

For peer review only - http://bmjopen.bmj.com/site/about/guidelines.xhtml

For peer review only

SECTION B: WORK

14 When did you first return to work after your carpal tunnel release surgery?

d	d	m	m	y	y	y	y

15 How much work-time did you miss between the date of your surgery and the date you first returned to work?
Please include all work-time missed, even if this had been pre-arranged with your employer, or was taken as annual leave. You can answer in hours, days or weeks, whichever applies.

☐☐☐ hours ☐☐ days ☐☐ weeks

15.1 Was any of this time paid?
Please tick one box (and provide the amount of time, if applicable).

a) Yes, all of my time away from work was paid ☐

b) Yes, some of my time away from work was paid ☐
(please specify how much time was paid, you can use hours, days or weeks, whichever applies)

☐☐☐ hours ☐☐ days ☐☐ weeks

c) No, none of my time off was paid ☐

d) Not sure ☐

16 Since your surgery, have you discussed when to return to work with anyone?
If yes, please answer the rest of Question 16; if no, please move on to Question 17.

Yes ☐ No ☐

16.1 If yes, who did you discuss this with? Please tick all that apply.

a) Your surgeon or a member of the surgical team ☐

b) Hospital nurse ☐

c) GP or practice nurse ☐

d) Hand therapist ☐

e) Physiotherapist or occupational therapist ☐

f) Occupational health nurse or doctor ☐

g) Employer or manager (or colleagues if self-employed ☐

h) Friend or family member ☐

i) Other *(please specify)* ☐

For peer review only - http://bmjopen.bmj.com/site/about/guidelines.xhtml

SECTION B: WORK

16.2 Please list any advice you have been given (since your surgery) about when and how to return to work?
This could include any activities to avoid or timescales to follow. If this advice came from more than one place, please indicate who advised what.

....

....

....

....

....

....

....

17 Since returning to work after your operation, have you needed to take any time off work because of a problem with your operated hand(s)/wrist(s)?
If yes, please answer Question 17.1; if no, please move on to Question 18.

Yes ☐ No ☐

17.1 If yes, how much time did you take off work?
Please answer in days or hours, whichever applies.

☐☐☐ hours ☐☐ days ☐☐ weeks

18 When you first returned to work after your surgery, did you work shorter hours than would be normal for your job as a direct result of your operation?
If yes, please answer the rest of Question 18; if no, please move on to Question 19.

Yes ☐ No ☐

18.1 Have you since gone back to working full hours?
If yes, please answer Question 18.2; if no, please move on to Question 19.

Yes ☐ No ☐

18.2 If yes, when did you return to full working hours?
If you do not know the exact date, approximately how many weeks did you work reduced hours?

☐☐☐☐☐☐☐☐
d d m m y y y y

a) Less than a week ☐

b) 1 – 2 weeks ☐

c) More than 2 weeks, but less than 3 weeks ☐

d) 3 weeks or longer ☐

For peer review only - http://bmjopen.bmj.com/site/about/guidelines.xhtml

SECTION B: WORK

19 When you first returned to work after your surgery, did you need to alter or avoid any of your usual work duties as a direct result of your operation?
If yes, please answer the rest of Question 19; if no, please move on to Question 20.

Yes ☐ No ☐

19.1 Have you since gone back to full duties?
If yes, please answer Question 19.2; if no, please move on to Question 20.

Yes ☐ No ☐

19.2 If yes, when did you return to full working duties?
If you do not know the exact date, approximately how many weeks did you have altered work duties?

d	d	m	m	y	y	y	y

a) Less than a week ☐

b) 1 – 2 weeks ☐

c) More than 2 weeks, but less than 3 weeks ☐

d) 3 weeks or longer ☐

20 If you would like to give us any additional information about returning to work, please do so here:

For peer review only - http://bmjopen.bmj.com/site/about/guidelines.xhtml

SECTION C: HAND AND WRIST SYMPTOMS

21 **The following questions refer to your symptoms over the *__last 7 days__*.**
Please answer for each hand. Please tick one box for each row.

**21.1** How severe were the following symptoms in your <u>RIGHT</u> hand?	None	Mild	Moderate	Severe	Very severe
a) Pain at night	☐	☐	☐	☐	☐
b) Pain during the daytime	☐	☐	☐	☐	☐
c) Numbness or tingling at night	☐	☐	☐	☐	☐
d) Numbness or tingling during the daytime	☐	☐	☐	☐	☐

How often did the following symptoms in your <u>RIGHT</u> hand wake you up at night?	Never	Once	2 or 3 times	4 or 5 times	More than 5 times
e) Pain	☐	☐	☐	☐	☐
f) Numbness or tingling	☐	☐	☐	☐	☐

**21.2** How severe were the following symptoms in your <u>LEFT</u> hand?	None	Mild	Moderate	Severe	Very severe
a) Pain at night	☐	☐	☐	☐	☐
b) Pain during the daytime	☐	☐	☐	☐	☐
c) Numbness or tingling at night	☐	☐	☐	☐	☐
d) Numbness or tingling during the daytime	☐	☐	☐	☐	☐

How often did the following symptoms in your <u>LEFT</u> hand wake you up at night?	Never	Once	2 or 3 times	4 or 5 times	More than 5 times
e) Pain	☐	☐	☐	☐	☐
f) Numbness or tingling	☐	☐	☐	☐	☐

22 **This question refers to the appearance (look) of your hands during the *__past 7 days__*.** Please tick one box for each hand.

	Strongly agree	Agree	Neither agree nor disagree	Disagree	Strongly disagree
a) I am satisfied with the appearance (look) of my **<u>RIGHT</u>** hand	☐	☐	☐	☐	☐
b) I am satisfied with the appearance (look) of my **<u>LEFT</u>** hand	☐	☐	☐	☐	☐

For peer review only

For peer review only - http://bmjopen.bmj.com/site/about/guidelines.xhtml

SECTION C: HAND AND WRIST SYMPTOMS

23 How do you rate your symptoms in your operated hand(s) now, compared to before your surgery? Please tick one box.

a) Completely cured ☐ c) Unchanged ☐ e) Worse ☐

b) Much better ☐ d) Slightly better ☐

24 The following questions ask specifically about your scar. Please think about your scar over the ***past 7 days.***

24.1 Has your scar been itchy?
If yes, please continue; if no, please move on to Question 24.2

Yes ☐ No ☐

Yes, it was itchy:	Sometimes ☐	Often ☐	Always ☐
And when it was itchy, it was:	Slightly itchy ☐	Fairly itchy ☐	Very itchy ☐

24.2 Has your scar caused you pain?
If yes, please continue; if no, please move on to Question 24.3

Yes ☐ No ☐

Yes, it was painful:	Sometimes ☐	Often ☐	Always ☐
And when it hurt, it was:	Slightly painful ☐	Fairly painful ☐	Very painful ☐

24.3 Has your scar been uncomfortable?
If yes, please continue; if no, please move on to Question 24.4

Yes ☐ No ☐

Yes, it was uncomfortable:	Sometimes ☐	Often ☐	Always ☐
And when it was uncomfortable, it was:	Slightly uncomfortable ☐	Fairly uncomfortable ☐	Very uncomfortable ☐

For peer review only

SECTION C: HAND AND WRIST SYMPTOMS

24.4 Has your scar felt numb?
If yes, please continue; if no, please move on to Question 24.5

Yes ☐ No ☐

Yes, it was numb:	Sometimes ☐	Often ☐	Always ☐
And when it felt numb, it was:	Slightly numb ☐	Fairly numb ☐	Very numb ☐

24.5 Have you had odd sensations in your scar e.g. tightening, pulling or pins and needles? If yes, please continue; if no, please move on to Question 24.6

Yes ☐ No ☐

Yes, I have had odd sensations:	Sometimes ☐	Often ☐	Always ☐

24.6 Has your scar caught on things e.g. clothing?
If yes, please continue; if no, please move on to Question 24.7

Yes ☐ No ☐

Yes, it has caught on things:	Sometimes ☐	Often ☐	Always ☐

24.7 Overall, how troublesome are the symptoms from your scar?
Please tick one box.

Not at all troublesome	A little troublesome	Fairly troublesome	Very troublesome	Unbearable
☐	☐	☐	☐	☐

For peer review only - http://bmjopen.bmj.com/site/about/guidelines.xhtml

SECTION D: HAND AND WRIST FUNCTION

The following questions refer to the function of your hands/wrists during the ***past 7 days***. Please answer all questions for the right and left sides, even if you do not experience any problems. Please tick one box for each question.

**25 RIGHT SIDE**	**Very well**	**Well**	**Adequately**	**Poorly**	**Very poorly**
a) Overall, how well did your ***right*** hand work?	☐	☐	☐	☐	☐
b) How well did your ***right*** fingers move?	☐	☐	☐	☐	☐
c) How well did your ***right*** wrist move?	☐	☐	☐	☐	☐
	**Very good**	**Good**	**Fair**	**Poor**	**Very poor**
d) How was the strength in your ***right*** hand?	☐	☐	☐	☐	☐
e) How was the sensation (feeling) in your ***right*** hand?	☐	☐	☐	☐	☐

**26 LEFT SIDE**	**Very well**	**Well**	**Adequately**	**Poorly**	**Very poorly**
a) Overall, how well did your ***left*** hand work?	☐	☐	☐	☐	☐
b) How well did your ***left*** fingers move?	☐	☐	☐	☐	☐
c) How well did your ***left*** wrist move?	☐	☐	☐	☐	☐
	**Very good**	**Good**	**Fair**	**Poor**	**Very poor**
d) How was the strength in your ***left*** hand?	☐	☐	☐	☐	☐
e) How was the sensation (feeling) in your ***left*** hand?	☐	☐	☐	☐	☐

The following questions refer to the ability of your hands to do certain tasks during the ***past 7 days***. If you do not do a certain task, please estimate the difficulty you would have in performing it. Please tick one box for every activity.

27 How difficult was it for you to perform the following activities using your RIGHT HAND?

	**Not at all difficult**	**A little difficult**	**Somewhat difficult**	**Moderately difficult**	**Very difficult**
a) Turn a door knob	☐	☐	☐	☐	☐
b) Pick up a coin	☐	☐	☐	☐	☐
c) Hold a glass of water	☐	☐	☐	☐	☐
d) Turn a key in a lock	☐	☐	☐	☐	☐
e) Hold a frying pan	☐	☐	☐	☐	☐

For peer review only - http://bmjopen.bmj.com/site/about/guidelines.xhtml

SECTION D: HAND AND WRIST FUNCTION

28 How difficult was it for you to perform the following activities using your LEFT HAND?

		Not at all difficult	A little difficult	Somewhat difficult	Moderately difficult	Very difficult
a)	Turn a door knob	☐	☐	☐	☐	☐
b)	Pick up a coin	☐	☐	☐	☐	☐
c)	Hold a glass of water	☐	☐	☐	☐	☐
d)	Turn a key in a lock	☐	☐	☐	☐	☐
e)	Hold a frying pan	☐	☐	☐	☐	☐

29 How difficult was it for you to perform the following activities using BOTH HANDS?

		Not at all difficult	A little difficult	Somewhat difficult	Moderately difficult	Very difficult
a)	Open a jar	☐	☐	☐	☐	☐
b)	Button a shirt/blouse	☐	☐	☐	☐	☐
c)	Eat with a knife/fork	☐	☐	☐	☐	☐
d)	Carry a grocery bag	☐	☐	☐	☐	☐
e)	Wash dishes	☐	☐	☐	☐	☐
f)	Wash your hair	☐	☐	☐	☐	☐
g)	Tie shoelaces/knots	☐	☐	☐	☐	☐

The following questions refer to your satisfaction with your hands/wrists during the ***past 7 days***. Please tick one box for each question.

30 How satisfied were you with your RIGHT hand/wrist during the *past 7 days*?

	RIGHT HAND	Very satisfied	Somewhat satisfied	Neither satisfied or dissatisfied	Somewhat dissatisfied	Very dissatisfied
a)	Overall function of your hand	☐	☐	☐	☐	☐
b)	Movement of the fingers	☐	☐	☐	☐	☐
c)	Movement of your wrist	☐	☐	☐	☐	☐
d)	Strength of your hand	☐	☐	☐	☐	☐
e)	Pain level of your hand	☐	☐	☐	☐	☐
f)	Sensation (feeling) of your hand	☐	☐	☐	☐	☐

For peer review only - http://bmjopen.bmj.com/site/about/guidelines.xhtml

SECTION D: HAND AND WRIST FUNCTION

31 How satisfied were you with your LEFT hand/wrist during the *past 7 days*?

	LEFT HAND	Very satisfied	Somewhat satisfied	Neither satisfied or dissatisfied	Somewhat dissatisfied	Very dissatisfied
a)	Overall function of your hand	☐	☐	☐	☐	☐
b)	Movement of the fingers	☐	☐	☐	☐	☐
c)	Movement of your wrist	☐	☐	☐	☐	☐
d)	Strength of your hand	☐	☐	☐	☐	☐
e)	Pain level of your hand	☐	☐	☐	☐	☐
f)	Sensation (feeling) of your hand	☐	☐	☐	☐	☐

32 If you would like to give us any additional information about your hand and wrist function, please do so here:

For peer review only

Thank you for completing this questionnaire! Please return it to the REACTS team using the pre-paid envelope.

REACTS

If you have any questions or would like any additional information, please contact Lisa Newington on:

ln@mrc.soton.ac.uk | 023 8077 7624 | 07866 997732

For peer review only - http://bmjopen.bmj.com/site/about/guidelines.xhtml

SUPPLEMENTARY TABLES

1. Cox proportional hazards univariate analyses of the association between demographic and general health factors and the duration of work absence after carpal tunnel release (non-significant findings)

	N	Median time to return to work (days)	Inter quartile range	Hazards ratio [1]	95% Confidence intervals	P value
Body mass index (kg/m²)						
Normal (18.5-24.9)	47	20	12-33	1	-	-
Over (25.0-29.9)	64	19	12.5-29	0.98	0.65, 1.46	0.91
Obese (≥30)	67	21	10-40	0.88	0.60, 1.28	0.50
Surgery to dominant hand [2]						
Yes	128	19.5	12-33	1	-	-
No	62	22	12-35	0.83	0.61, 1.14	0.25
General health status						
Excellent/very good/ good	165	19	12-32	1	-	-
Fair/poor	24	28.5	12.5-42	0.74	0.47, 1.16	0.19
Number of comorbidities [3]						
None	49	18	7-32	1	-	-
One	67	24	14-42	0.87	0.59, 1.27	0.47
Two or more	74	19.5	10-29	1.11	0.76, 1.62	0.60
Number of disability comorbidities [3]						
None	130	19	9-33	1	-	-
One	39	26	13-31	0.75	0.52, 1.08	0.12
Two or more	21	20	14-31	0.81	0.50, 1.32	0.40
Number of somatising symptoms [4]						
None	96	19	10.5-35	1	-	-
One	51	22	13-34	0.98	0.70, 1.38	0.91
Two or more	42	19.5	14-31	0.84	0.58, 1.22	0.36
SF36 Mental health score (tertiles) [5]						
Poor (22.2-57.8)	60	24	16-39	0.71	0.50, 1.03	0.07
Intermediate (60.0-75.6)	68	18.5	11-36.5	0.88	0.62, 1.25	0.48
Good (77.8-97.8)	60	16.5	7-30	1	-	-

Total sample size n=195.

[1.] Adjusted for age and sex.

[2.] Considered and the non-dominant hand for those who reported ambidexterity (n=4) and dominant hand for those undergoing simultaneous bilateral surgery (n=2).

[3.] Adapted from the self-administered co-morbidity questionnaire [1].

[4.] Adapted from the four-dimensional symptom questionnaire [2].

[5.] Taken from the SF-36 with data-driven tertiles [3].

For peer review only

2. Cox proportional hazards univariate analyses of the association between health beliefs and the duration of work absence after carpal tunnel release (non-significant findings)

	N	Median time to return to work (days)	Inter quartile range	Hazards ratio [1]	95% Confidence intervals	P value
Believe will be unable to use hand normally in 3 months [2]						
No	168	20	12-32.5	1	-	-
Yes	20	20.5	14.5-40	1.04	0.65, 1.67	0.87
Blames self for hand problem [2]						
No	162	20	12-35	1	-	-
Yes	26	22	14-31	1.08	0.70, 1.66	0.73
Lack of support from family/friends [2]						
No	156	20	12-32.5	1	-	-
Yes	32	19.5	13-40	0.88	0.60, 1.29	0.51
Problem runs in the family/born with a weakness [3]						
No	134	20	12-35	1	-	-
Agree/strongly agree	54	23	12-31	0.88	0.63, 1.21	0.42
Stress made the problem worse [3]						
No	169	20	12-33	1	-	-
Agree/strongly agree	19	28	14-40	0.79	0.48, 1.28	0.33
Lack of exercise contributed to the problem [3]						
No	172	20	13-33	1	-	-
Agree/strongly agree	16	15	10.5-34.5	1.23	0.73, 2.06	0.43
Getting older contributes to the problem [3]						
No	79	26	14-42	1	-	-
Agree/strongly agree	108	16	10-29	1.28	0.94, 1.74	0.11
Work probably didn't cause the problem, but made it worse [3]						
No	100	20.5	12-35	1	-	-
Agree/strongly agree	86	19.5	13-31	1.01	0.75, 1.35	0.95
Pain catastrophisation to at least a moderate degree [4]						
No	133	19	10-33	1	-	-
Yes	55	22	15-31	0.81	0.59, 1.12	0.21

Total sample size n=195.

1. Adjusted for age and sex.
2. Reported on a 0-10 scale and dichotomised to no (0-6) and yes (7-10) [4].
3. Reported on a 5-point scale and dichotomised to agree/strongly agree and neither agree nor disagree/disagree/disagree strongly [5].
4. Pain catastrophizing scale dichotomised to those who reported catastrophising pain thoughts and feelings to at least a moderate degree in response to all questions and those who did not [6].

For peer review only

3. Cox proportional hazards univariate analyses of the association between clinical and surgical factors and the duration of work absence after carpal tunnel release (non-significant findings)

	N	Median time to return to work (days)	Inter quartile range	Hazards ratio [1]	95% Confidence intervals	P value
Katz hand diagram score for side of surgery [2]						
Classic/probable	126	22	14-38	1	-	-
Possible/unlikely	60	16.5	9.5-30.5	1.10	0.81, 1.51	0.54
Duration of symptoms						
≤ 1 year	47	19	10-31	1	-	-
> 1 year	141	20	13-34	0.86	0.62, 1.21	0.38
Nerve conduction studies performed						
No	71	21	14-35	1	-	-
Yes	79	20	11-33	1.14	0.83, 1.58	0.41
Type of suture material						
Non-absorbable	119	21	12-38	1	-	-
Absorbable	23	20	13-29	1.15	0.73, 1.83	0.55
MHQ function score for side of surgery (tertiles) [3]						
Poor (0-40)	79	20	13-41	0.80	0.57, 1.12	0.19
Intermediate (44-55)	48	19.5	13.5-33	0.81	0.55, 1.19	0.29
Good (60-100)	61	20	7-29	1	-	-
MHQ bilateral activities of daily living score (tertiles) [3]						
Poor (0-61)	72	19	12.5-33.5	0.96	0.66, 1.38	0.81
Intermediate (64-82)	55	21	13-38	0.70	0.49, 1.01	0.06
Good (83-100)	62	20	10-31	1	-	-
MHQ activities of daily living score for side of surgery (tertiles) [3]						
Poor (0-55)	63	20	12-42	0.81	0.56, 1.18	0.27
Intermediate (58-80)	63	20	14-35	0.86	0.61, 1.23	0.42
Good (85-100)	63	20	9-32	1	-	-
MHQ satisfaction score for side of surgery (tertiles) [3]						
Poor (0-25)	88	21.5	13-42	0.78	0.55, 1.11	0.16
Intermediate (29-50)	49	18	14-29	0.87	0.59, 1.28	0.48
Good (54-100)	53	20	9-31	1	-	-
Satisfaction with appearance for side of surgery [4]						
Satisfied	136	20	10.5-31.5	1	-	-
Dissatisfied	53	20	13-40	0.84	0.61, 1.16	0.29

Total sample size n=195.

1. Adjusted for age and sex.
2. Adapted from Calfee et al. [7].
3. Michigan Hand Questionnaire [8]. Data-driven tertiles.
4. Reported on a 5-point scale in response to the statement: I am satisfied with the appearance (look) of my hand. Dichotomised as satisfied (strongly agree/agree) and dissatisfied (neither agree nor disagree, disagree, strongly disagree).

For peer review only

4. Cox proportional hazards univariate analyses of the association between occupational factors and the duration of work absence after carpal tunnel release (non-significant findings)

	N	Median time to return to work (days)	Inter quartile range	Hazards ratio [1]	95% Confidence intervals	P value
Have more than one paid job						
No	177	20	12-32	1	-	-
Yes	12	26.5	19-42	0.61	0.34, 1.11	0.11
Total paid work hours per week [2]						
≤37.5	97	22	15-38	1	-	-
>37.5	93	16	6-29	1.25	0.88, 1.79	0.21
Number of work days per week						
<5	55	23	15-35	1	0.72, 1.39	0.98
5	116	19	10.5-33	1	-	-
>5	19	13	6-31	1.28	0.77, 2.14	0.34
Sick leave taken for this problem in the last month						
No	154	20	12-33	1	-	-
Yes	20	15	9.5-30	0.99	0.61, 1.60	0.95
Sick leave taken for another problem in the last month						
No	152	19	10.5-31	1	-	-
Yes	18	30	21-42	0.67	0.41, 1.09	0.10
Received advice about return to work after surgery						
Yes	134	19	12-31	1	-	-
No	53	22	14-41	0.92	0.67, 1.27	0.61
Required to work to tight deadlines [3]						
No	75	23	14-40	1	-	-
Yes	112	18	9.5-31	1.19	0.89, 1.61	0.24
Use power tools that make the hand/arm vibrate [3]						
No	145	19	12-32	1	-	-
Yes	38	22	14-42	0.68	0.44, 1.05	0.08
Work with hands above shoulder height for >1 hour [3]						
No	147	20	12-33	1	-	-
Yes	34	19.5	13-33	0.89	0.60, 1.32	0.56
Work with neck bent forward for >2 hours [3]						
No	114	21	13-39	1	-	-
Yes	73	18	8-31	1.19	0.88, 1.60	0.26
Work with neck twisted for >30 minutes [3]						
No	136	20	12-34	1	-	-
Yes	47	22	13-32	0.93	0.66, 1.32	0.70
Drive for >1 hour per day [3]						
No	115	21	13-38	1	-	-
Yes	70	16	7-31	1.22	0.88, 1.68	0.24

Total sample size n=195.

1. Adjusted for age and sex.

2. Dichotomised by the median for the sample population

3. As part of the normal working day [9].

For peer review only

References

1. Sangha O, Stucki G, Liang MH et al. The self-administered comorbidity questionnaire: a new method to assess comorbidity for clinical and health services research *Arthritis & Rheumatism* 2003;49(2):156-63.
2. Terluin B, van Marwijk HW, Adèr HJ et al. The Four-Dimensional Symptom Questionnaire (4DSQ): a validation study of a multidimensional self-report questionnaire to assess distress, depression, anxiety and somatization. *BMC Psychiatry* 2006;6(1):34.
3. Ware J, Sherbourne C. The MOS 36-item short-form health survey (SF-36): I. conceptual framework and item selection. *Medical Care* 1992;30:473-83.
4. Hansen TB, Dalsgaard J, Meldgaard A et al. A prospective study of prognostic factors for duration of sick leave after endoscopic carpal tunnel release. *BMC Musculoskelet Disord* 2009;10:144.
5. Palmer K, Calnan M, Wainwright D et al. Upper limb pain in primary care: health beliefs, somatic distress, consulting and patient satisfaction. *Family Practice* 2006;23(6):609-17.
6. Bot AG, Becker SJ, Bruijnzeel H et al. Creation of the abbreviated measures of the Pain Catastrophizing Scale and the Short Health Anxiety Inventory: the PCS-4 and SHAI-5. *J Musculoskelet Pain* 2014;22(2):145-51.
7. Calfee RP, Dale AM, Ryan D et al. Performance of simplified scoring systems for hand diagrams in carpal tunnel syndrome screening. *J Hand Surg Am* 2012;37(1):10-17.
8. University of Michigan Medical School. The Michigan Hand Questionnaire: http://mhq.lab.medicine.umich.edu/. Accessed 04/04/2019.
9. Karasek R, Brisson C, Kawakami N et al. The Job Content Questionnaire (JCQ): an instrument for internationally comparative assessments of psychosocial job characteristics. *J Occup Health Psychol* 1998;3(4):322-55.

For peer review only

STROBE checklist (cohort study)

Item	Recommendation	Location
**Title and abstract**		
Title	Indicate study design	Title
**Introduction**		
Background	Explain scientific background and rationale	Introduction
Objectives	State objectives and any pre-specified hypotheses	Introduction
**Methods**		
Study design	Present key elements of the study design early	Introduction & methods
Setting	Describe the setting, locations and relevant dates	Methods
Participants	Give eligibility criteria, sources and methods of selection of participants, and methods of follow-up	Methods & table 1
Variables	Clearly define all outcomes, exposures, predictors, potential confounders and effect modifiers	Methods & supplementary files 1-2
Data sources	Give sources of data and methods of assessment	Methods & tables 2-5
Bias	Describe and effects to address potential sources of bias	Methods & discussion
Study size	Explain how the study size was determined	Methods
Quantitative variables	Described how quantitative variables were handled in the analyses	Tables 2-5
Statistical methods	Described all statistical methods<br>Explain how missing data were addressed<br>Explain how lost to follow-up was addressed	Statistical methods & tables 2-5
**Results**		
Participants	Reported the number of individuals at each stage of the study<br>Give reasons for non-participation	Figure 1<br>Figure 1
Descriptive data	Give characteristics of study participants<br>Indicate number of participants for each variables of interest<br>Summarise follow-up time	Tables 2-5<br>Tables 2-5<br>Results
Outcome data	Report number of outcome events over time	Results
Main results	Give adjusted and unadjusted estimates and measures of precision. Make clear which confounders were adjusted for	Table 4 & supplementary file 3
Other analyses	Reported any subgroup analyses	Table 4 & supplementary file 3

For peer review only

BMJ Open

Sickness absence after carpal tunnel release: a multi-centre prospective cohort study

Journal:	*BMJ Open*
Manuscript ID	bmjopen-2020-041656.R1
Article Type:	Original research
Date Submitted by the Author:	09-Dec-2020
Complete List of Authors:	Newington, Lisa; University of Southampton Faculty of Medicine, MRC Versus Arthritis Centre for Musculoskeletal Health and Work, MRC Lifecourse Epidemiology Unit; Guy's and Saint Thomas' NHS Foundation Trust, Hand Therapy<br>Ntani, G; University of Southampton Faculty of Medicine, MRC Versus Arthritis Centre for Musculoskeletal Health and Work, MRC Lifecourse Epidemiology Unit<br>Warwick, David; University Hospital Southampton NHS Foundation Trust, Hand Surgery; University of Southampton Faculty of Medicine<br>Adams, Jo; University of Southampton, Centre for Innovation and Leadership in Health Sciences, School of Health Sciences, Faculty of Environment and Life Sciences; University of Southampton, Centre for Sport, Exercise and Osteoarthritis Research Versus Arthritis<br>Walker-bone, Karen; University of Southampton Faculty of Medicine, MRC Versus Arthritis Centre for Musculoskeletal Health and Work, MRC Lifecourse Epidemiology Unit
**Primary Subject Heading**:	Surgery
Secondary Subject Heading:	Epidemiology, General practice / Family practice, Occupational and environmental medicine
Keywords:	Hand & wrist < ORTHOPAEDIC & TRAUMA SURGERY, PRIMARY CARE, OCCUPATIONAL & INDUSTRIAL MEDICINE

SCHOLARONE™
Manuscripts

I, the Submitting Author has the right to grant and does grant on behalf of all authors of the Work (as defined in the below author licence), an exclusive licence and/or a non-exclusive licence for contributions from authors who are: i) UK Crown employees; ii) where BMJ has agreed a CC-BY licence shall apply, and/or iii) in accordance with the terms applicable for US Federal Government officers or employees acting as part of their official duties; on a worldwide, perpetual, irrevocable, royalty-free basis to BMJ Publishing Group Ltd ("BMJ") its licensees and where the relevant Journal is co-owned by BMJ to the co-owners of the Journal, to publish the Work in this journal and any other BMJ products and to exploit all rights, as set out in our licence.

The Submitting Author accepts and understands that any supply made under these terms is made by BMJ to the Submitting Author unless you are acting as an employee on behalf of your employer or a postgraduate student of an affiliated institution which is paying any applicable article publishing charge ("APC") for Open Access articles. Where the Submitting Author wishes to make the Work available on an Open Access basis (and intends to pay the relevant APC), the terms of reuse of such Open Access shall be governed by a Creative Commons licence – details of these licences and which Creative Commons licence will apply to this Work are set out in our licence referred to above.

Other than as permitted in any relevant BMJ Author's Self Archiving Policies, I confirm this Work has not been accepted for publication elsewhere, is not being considered for publication elsewhere and does not duplicate material already published. I confirm all authors consent to publication of this Work and authorise the granting of this licence.

For peer review only

TITLE

Sickness absence after carpal tunnel release: a multi-centre prospective cohort study

AUTHORS

Lisa Newington [1, 2] *

Georgia Ntani [1]

David Warwick [3]

Jo Adams [4, 5]

Karen Walker-Bone [1]

AFFILIATIONS

1. MRC Versus Arthritis Centre for Musculoskeletal Health and Work, MRC Lifecourse Epidemiology Unit, University of Southampton, Southampton, UK.
2. Hand Therapy, Guy's and St Thomas' NHS Foundation Trust, St Thomas' Hospital, London, UK.
3. Hand Surgery, University Hospital Southampton NHS Foundation Trust and Faculty of Medicine, University of Southampton, Southampton, UK.
4. Centre for Innovation and Leadership in Health Sciences, School of Health Sciences, Faculty of Environment and Life Sciences, University of Southampton, Southampton, UK.
5. Centre for Sport, Exercise and Osteoarthritis Research Versus Arthritis, University of Southampton, Southampton, UK.

*** Corresponding author**

Address: Hand Therapy, Guy's and St Thomas' NHS Foundation Trust, St Thomas' Hospital, London, SE1 7EH, UK.

Email: l.newington@nhs.net

Telephone: 0207 7188 4174

Fax: none

KEY WORDS

Carpal tunnel syndrome, carpal tunnel release, return to work, advice, sickness absence, cohort study

Word count: 3,713

For peer review only

TITLE

Sickness absence after carpal tunnel release: a multi-centre prospective cohort study

ABSTRACT

Objectives: To describe when patients return to different types of work after elective carpal tunnel release (CTR) surgery and identify the factors associated with the duration of sickness absence.

Design: Multi-centre prospective observational cohort study.

Setting and participants: Participants were recruited pre-operatively from 16 UK centres and clinical, occupational and demographic information were collected. Participants completed a weekly diary and questionnaires at four and 12 weeks post-operatively.

Outcomes: The main outcome was duration of work absence from date of surgery to date of first return to work.

Results: 254 participants were enrolled in the study and 201 provided follow-up data. Median duration of sickness absence was 20 days (range 1-99). Earlier return to work was associated with having surgery in primary care and a self-reported work role involving more than four hours of daily computer use. Being female and entitlement to more than a month of paid sick leave were both associated with longer work absences. Duration of work absence was strongly associated with the expected duration of leave, as reported by participants before surgery. Earlier return to work was not associated with poorer clinical outcomes reported 12 weeks after CTR.

For peer review only

Conclusions: There was wide variation in the duration of work absence after CTR across all occupational categories. A combination of occupational, demographic and clinical factors was associated with the duration of work absence, illustrating the complexity of return to work decision-making. However, pre-operative expectations were strongly associated with the actual duration of leave. We found no evidence that earlier return to work was harmful. Clear, consistent advice from clinicians pre-operatively setting expectations of a prompt return to work could reduce unnecessary sickness absence after CTR. To enable this, clinicians need evidence-informed guidance about appropriate timescales for the safe return to different types of work.

STRENGTHS AND LIMITATIONS OF THIS STUDY

- This multi-centre, prospective study with a large sample size provides robust evidence to understand return to work issues after carpal tunnel release surgery.
- Participants were recruited from primary care, secondary care and private practice sites, representing the breath of locations where carpal tunnel release is performed in the UK.
- Work absence was the primary outcome and a clear definition was used for its duration with data collected contemporaneously to limit recall bias.
- A standardised method was used to categorise occupations and measure occupational exposures, although this relied on job title, which may not be a true reflection of actual occupational activity.
- All participants underwent open carpal tunnel release, however the method of carpal tunnel syndrome diagnosis was not independently verified and different case definitions for carpal tunnel syndrome may have been included.

For peer review only

INTRODUCTION

Carpal tunnel syndrome (CTS) occurs when the median nerve becomes compressed within the carpal tunnel at the wrist. Typical sensory symptoms include pain, paraesthesia and/or numbness in the thumb, index, middle and radial half of the ring finger; and motor symptoms include progressive wasting of the thenar muscles. CTS is often associated with marked functional difficulty[1] and treatment is targeted at reducing the median nerve compression by splinting, corticosteroid injection or carpal tunnel release (CTR) surgery[2, 3].

Recent estimates suggest that more than 90,000 CTR procedures will be performed annually in the English NHS by 2025[4], and as the peak incidence for CTS falls within the working lifetime[5], many of these patients will need to return to work after their CTR. However, there is currently no evidence on which to base recommendations about when it might be safe to return to functional activities, including work, after carpal tunnel release (CTR). Our previous survey of UK hand surgeons, primary care surgeons and hand therapists found that clinicians recommended a wide range of times to return to three specified job roles after CTR: 0-30 days for desk-based work (e.g. keyboard, mouse, writing and telephone); 1-56 days for repetitive light manual work (driving, delivery, stacking); and 1-90 days for heavy manual work (e.g. construction)[6]. However, there has not previously been a prospective study of CTR patients in the UK in which time to return to work was the primary outcome. Therefore, it is not known when UK patients return to different occupational activities after CTR or what influences the duration of work absence. It is also unclear whether earlier return to work has a detrimental effect on post-operative clinical outcomes. Possible consequences of returning to work too soon after CTR include wound dehiscence, infection, and delayed healing. Conversely, delayed return to work may increase the risk of progression to long-term sick leave[7] and produce a financial burden for the individual, employer or state.

For peer review only

A systematic review of the prognostic factors associated with return to work after CTR identified 11 studies which evaluated more than 90 potential prognostic factors[8]. Longer durations of work absence after CTR were found to be associated with: receipt of workers' compensation[9]; manual work[10-12]; longer expected durations of work absence[10]; being on sick leave before CTR surgery[13]; self-blame for the hand problem[13]; and beliefs that the symptoms were caused by work[12].

Much of the existing research has been conducted at single sites and involved small numbers of participants. Furthermore, very few studies have specifically explored the influence of a range of occupational factors. The current multi-centre prospective cohort study was designed to explore when patients returned to different types of work after CTR and the demographic, clinical and occupational factors associated with duration of work absence. We also investigated whether earlier return to work was associated with poorer clinical outcomes assessed at 12 weeks after CTR.

METHODS

This was a prospective cohort study and a convenience sample of participants were recruited from 16 sites in England and Wales between March 2017 and August 2018. Recruitment took place before CTR surgery, either at the time of listing for surgery, during pre-operative screening, or on the day of surgery. At each site, the patient CTR pathway and treatment was carried out as usual. Sites were NHS secondary care (hospital setting), NHS primary care and private hand surgery facilities, representing the range of UK healthcare facilities where CTR is performed. Provision of CTR in the UK was explored through discussion with relevant national

For peer review only

organisations (British Society for Surgery of the Hand and Association for Surgeons in Primary Care). Sites were recruited through National Institute for Health Research infrastructure.

Eligibility criteria are shown in Table 1. Baseline demographic, general health and occupational information was collected via self-completed questionnaire at the time of recruitment. The questionnaire also included standardised measures of CTS symptoms[14-16] and hand function[17]. Questionnaire content was informed by the clinical, demographic and occupational factors previously identified in a systematic review of prognostic factors for return to work after CTR[8], and developed in collaboration with our patient advisory group. The reasoning for item inclusion is provided in supplementary file 1.

TABLE 1. Study eligibility criteria

**Self-selected by potential participants**
1. Aged over 18 and referred for carpal tunnel release surgery
2. Routinely work in paid employment for at least 20 hours per week
3. Plan to return to work after carpal tunnel release surgery
4. Have not previously had carpal tunnel release surgery on either hand
5. Have not previously had a serious injury to the same wrist/hand
**Assessed by recruiting clinician**
1. No planned surgical procedures for conditions other than carpal tunnel syndrome

Follow-up questionnaires were completed four and 12 weeks after CTR and collected information about return to work, work functioning, scar symptoms, CTS symptoms, and hand function. Study questionnaires are provided as supplementary files 2-3. Participants were also asked to complete a short weekly diary from the day after surgery until return to work, detailing whether they had returned to work that week, and if so, the date of return. Steps were taken to minimise loss to follow-up after recruitment. To maximise retention, we

For peer review only

incentivised with a shopping voucher on completion of the study (£10) and sent up to three reminders using a combination of post, email and text.

Surgical information was collected from the medical records by a member of the participant's clinical team. This included: date of CTR, operated hand(s), nature of anaesthetic, incision size, additional procedures, unexpected findings and suture material. Date, side of CTR and anaesthetic (general/local) were also reported by participants for cross-checking.

Public and patient involvement

This research was supported by a patient advisory group consisting of six individuals who had previously undergone CTR at different UK sites. Study questionnaires were developed in collaboration with the patient advisors and these individuals also provided their feedback on the preliminary findings.

Statistical methods

Comparisons were made between those who dropped out of the study before providing any follow-up data and those in the final study sample using pre-specified demographic, clinical and occupational variables (Table 2).

Manual and non-manual work was coded from job title and industry using the UK Standard Occupational Classification[18, 19]. Return to work time was calculated from the date of surgery to the date of first return to work (as reported by participants).

A Cox proportional hazards model was used to explore the factors associated with return to work time. Baseline and operative variables were assessed in univariable analyses and those

For peer review only

which were significant ($p<0.05$) were included as covariates in the final model. All regression analyses were adjusted for age and sex.

Participants were defined as having a poor outcome if they reported one or more of the following: global rating of change score of “worse”, “unchanged” or “slightly improved” (12 weeks after CTR)[20]; scar symptoms described as “unbearable”, “very troublesome” or “fairly troublesome” (12 weeks after CTR); use of antibiotics for an incision site infection after returning to work; and additional sick-leave related to the CTR after returning to work. The duration of work absence for those with/without poor outcomes were compared using Wilcoxon rank-sum test. In addition, participants were dichotomised to those who returned to work within/after seven, 14 and 28 days of surgery and the prevalence rates of poor outcomes were compared using Chi^2 for each time period. These time points were based on the median clinician-recommended return to work time that we reported previously[6].

There was no imputation for missing data. Missing values were coded as a separate category for each of the variables included, and participant numbers are provided for each variable in the accompanying tables.

RESULTS

A total of 254 individuals completed the baseline questionnaire and 201 (79%) provided follow-up data. Participant numbers and loss to follow-up for each study component are shown in Figure 1. Participant demographics and comparisons between those who remained in the study and those who dropped out before follow-up are shown in Table 2.

Figure 1. Participant numbers for each stage of the cohort study

For peer review only

TABLE 2. Participant demographics assessed at baseline in comparison with those lost to follow-up

	Study participants n=201 (%)	Lost to follow-up n=53 (%)
Mean age in years (SD)	52.0 (9.16)	44.4 (9.55)
Sex		
Male	76 (37.8)	20 (37.7)
Female	125 (62.2)	33 (62.3)
Body mass index (kg/m²)		
Normal (18.5-24.9)	48 (23.9)	9 (17.0)
Overweight (25-29.9)	66 (32.8)	16 (30.2)
Obese (≥30)	73 (36.3)	22 (41.5)
Smoking status		
Never smoked	109 (54.2)	26 (49.1)
Current/ex-smoker	90 (44.8)	27 (50.9)
General health		
Excellent/very good/good	174 (86.6)	42 (79.3)
Fair/poor	26 (12.9)	11 (20.8)
Number of comorbidities		
None	54 (26.9)	21 (39.6)
One	70 (34.8)	13 (24.5)
Two or more	77 (38.3)	19 (35.9)
Number of disabling comorbidities		
None	138 (68.7)	35 (66.0)
One	41 (20.4)	9 (17.0)
Two or more	22 (11.0)	9 (17.0)
Mean SF-36 mental health score (SD) [a]	65.6 (17.20)	60.3 (20.41)
Mean bilateral CTS-6 score (SD) [b]	2.8 (0.77)	3.0 (0.73)
Mean MHQ bilateral activities of daily living score (SD) [c]	68.8 (23.64)	55.7 (28.62)
Mean MHQ work function score (SD) [c]	66.1 (22.26)	60.6 (22.61)
Type of job contract		
Employed (permanent contract)	164 (81.6)	37 (69.8)
Self-employed	31 (15.4)	13 (24.5)
Employed (temporary or zero hours contract)	5 (2.5)	3 (5.7)
Type of work [d]		
Manual	77 (39)	31 (58)
Non-manual	123 (61)	22 (42)
Median level of job demand on hands/wrists (IQR) [e]	9 (7-10)	10 (7-10)
Job satisfaction		
Very satisfied	87 (43.3)	24 (45.3)
Satisfied/fairly satisfied	92 (45.8)	24 (45.3)
Dissatisfied/very dissatisfied	20 (10.0)	5 (9.4)
Median expected work absence in days (IQR)	14 (7-28)	14 (5-21)
Expected availability of sick pay		
≤1 month	50 (24.9)	21 (39.6)
>1 month	94 (46.8)	11 (20.8)
Unsure	57 (28.4)	21 (39.6)

For peer review only

Study site [f]		
NHS primary care	73 (36.3)	13 (24.5)
NHS Secondary care	101 (50.3)	32 (60.4)
Private hand surgery facilities	27 (13.4)	8 (15.1)

[a.] SF-36 mental health score ranges from 0-100, higher scores indicate better mental health[21].
[b.] CTS-6 symptom score ranges from 1-5, higher scores indicate more severe symptoms[14].
[c.] Michigan Hand Questionnaire ranges from 0-100, higher scores indicate better functioning[17].
[d.] Classified using the Office for National Statistics Standard Occupational Classification 2010[18, 19].
[e.] Job demand scale range 0-10, 10 indicating very demanding on hands/wrists[13].
[f.] Location where the carpal tunnel release surgery was performed. Surgery in primary care was performed by General Practitioners who had completed additional training.

Eighty-six participants (43%) were recruited pre-operatively on the day of their CTR. For the remaining 115 participants, the median time between recruitment and CTR was 14 days (interquartile range [IQR] 5-40). The first follow-up questionnaire was completed a median of 32 days after CTR (IQR 29-38) and the final questionnaire was completed a median of 92 days after CTR (IQR 86-105). All participants underwent open CTR as a day case, and all but two had unilateral surgery. Another two participants required median nerve neurolysis, and one participant was noted to have a vascular abnormality. Sixty-two percent of participants (n=125) were expecting to have a CTR for their other hand in the future. Other baseline demographic and surgical factors are shown in Table 3.

TABLE 3. Participant demographic and surgical factors

	Number of participants n=201 (%)
Age (years)	
26-40	23 (11.4)
41-55	101 (50.3)
≥ 55	77 (38.3)
Hand dominance	
Right	178 (88.6)
Left	18 (9.0)
Ambidextrous	5 (2.5)
Side of surgery [a]	
Dominant hand	134 (66.7)
Non-dominant hand	65 (32.3)
Bilateral surgery	2 (1.0)
Surgical speciality and grade	

For peer review only

Consultant (plastics/orthopaedics)	64 (31.8)
Registrar (plastics/orthopaedics)	33 (16.4)
General practitioner	62 (30.9)
Not reported	42 (20.9)
Incision type [b]	
Mini open incision	129 (64.2)
Traditional incision	2 (1.0)
Not reported	70 (34.8)
Suture material	
Absorbable	24 (11.9)
Non-absorbable	126 (62.7)
Not reported	51 (24.4)

[a.] Considered as the non-dominant hand for those who reported ambidexterity.
[b.] Mini open incision defined as distal to the distal wrist crease and traditional open excision extending proximally.

The majority of participants (62%) worked five days per week (range 2-7) and the median number of weekly paid work hours was 37.5 (IQR 31-45). Two participants (1%) did not return to work during the 12-week study period: one reported that they had been made redundant and the other that their job was no longer available. Four participants (2%) had incomplete data (missing return to work date or CTR date) meaning that the duration of work absence could not be calculated. These six individuals were not included in the analyses of return to work time, leaving a total sample size of 195. An additional five participants reported that they had not returned to work, but planned to do so in the future. These individuals were included in the return to work analysis, censored to the time of last follow-up.

The median duration of work absence after CTR was 20 days (IQR 12-33). Manual workers took longer to return than non-manual workers: 23.5 days (IQR 14-41) compared with 18 days (IQR 9-31). Those who were self-employed returned to work earlier than those who were employed: 13 days (IQR 6-19) compared with 22 days (IQR 14-38). Return to work times are shown in Figure 2. The majority of participants returned to work on a Monday (43%). Approximately 15% returned each day between Tuesday-Thursday, then ~5% returned each

For peer review only

day from Friday-Sunday. More than half of participants (59%) reported that they needed to modify their work duties to some extent when they first returned to work. Of these, 62% had resumed full duties within five weeks.

Univariable analyses of the relationship between baseline factors and the duration of work absence found 17 factors (age- and sex-adjusted) that were significantly associated with time to return to work and were entered into the multivariable model, in which five factors remained significant (Table 4). Sensitivity analyses confirmed that these factors remained independently significant in the model. Non-significant findings in the univariable analyses are provided in supplementary file 4. Having surgery in primary care and having a job with more than four hours of daily computer use were both associated with earlier return to work than their respective reference categories. Being female and having access to more than a month of paid sick leave were both associated with longer durations of work absence than their respected reference categories. Compared to those who expected to return within a week, there was a sequential increase in the likelihood of longer durations of work absence for those expecting to return between 7-14 days, 15-30 days and >30 days (Table 4), which showed a significant gradient effect ($p<0.001$).

For peer review only

TABLE 4. Cox proportional hazards analyses of the association between baseline demographic, clinical and occupational factors and the duration of work absence after carpal tunnel release

				Univariable analyses			Multivariable analysis		
	N	Median work absence (days)	Inter quartile range	Hazards ratio	95% Confidence intervals	P value	Hazard s ratio	95% Confidence intervals	P value
Sex									
Male	72	17.5	8-31	1	-	-	1	-	-
Female	118	21.5	14-35	0.79	0.59, 1.06	0.12	0.56	0.36, 0.88	**0.01**
Age in years									
26-40	21	20	15-30	1.01	0.63, 1.63	0.96	1.44	0.82, 2.55	0.21
41-55	94	20	9-33	1	-	-	1	-	-
>55	75	17	12-35	1.03	0.76, 1.40	0.83	1.15	0.80, 1.65	0.44
Smoking status									
Never	105	16	12-31	1	-	-	1	-	-
Current/ex	83	23	13-41	0.74	0.56, 1.00	0.046	0.75	0.51, 1.09	0.13
Site									
NHS Primary care	72	19.5	13-33	1.18	0.87, 1.62	0.29	1.54	1.05, 2.25	**0.03**
NHS Secondary care	92	20	12-39.5	1	-	-	1	-	-
Private facilities	26	20	7-28	1.63	1.04, 2.54	0.03	1.46	0.87, 2.44	0.15
Afraid of long-term hand problems [a]									
No	105	19	12-31	1	-	-	1	-	-
Yes	82	20.5	13-42	0.69	0.51, 0.93	0.01	0.93	0.67, 1.30	0.68
CTS-6 score for side of surgery (tertiles) [b]									
Good (1-3.0)	65	16	10-27	1	-	-	1	-	-
Intermediate (3.2-3.8)	58	21.5	14-35	0.77	0.54, 1.10	0.15	1.19	0.77, 1.84	0.44
Poor (3.8-5)	59	24	13-41	0.67	0.47, 0.97	0.03	1.04	0.65, 1.66	0.87
Type of work contract									
Employed (permanent)	154	22	14-38	1	-	-	1	-	-
Self-employed	30	13	6-19	1.72	1.13, 2.61	0.01	1.19	0.67, 2.14	0.55
Zero hours/temporary	5	12	3-31	2.01	0.81, 5.00	0.13	0.73	0.25, 2.14	0.56
Duration of available sick pay									
≤1 month	49	16	12-29	1	-	-	1	-	-
>1 month	88	27	15-42	0.59	0.41, 0.85	0.004	0.46	0.28, 0.76	**0.002**
Unsure	53	16	10-23	1.19	0.80, 1.77	0.40	1.01	0.61, 1.66	0.97
Access to occupational health at work									
No	110	15.5	9-29	1.77	1.31, 2.38	<0.001	1.42	0.91, 2.19	0.12
Yes	79	25	16-42	1	-	-	1	-	-
Expected duration of leave after carpal tunnel release (days)									
<7	35	4	2-12	1	-	-	1	-	-
7-14	75	16	13-26	0.23	0.15, 0.36	<0.001	0.27	0.16, 0.45	**<0.001**
15-29	35	29	22-39	0.12	0.07, 0.19	<0.001	0.19	0.10, 0.37	**<0.001**
≥30	45	42	21-44	0.08	0.05, 0.14	<0.001	0.12	0.06, 0.23	**<0.001**
MHQ work functioning score (tertiles) [c]									
Poor (0-55)	67	20	13-35	0.68	0.47, 0.98	0.04	0.83	0.50, 1.40	0.49
Intermediate (60-80)	72	21	12.5-39.5	0.77	0.53, 1.10	0.15	0.81	0.50, 1.31	0.39
Good (81-100)	51	17	10-29	1	-	-	1	-	-
Job satisfaction [d]									
Satisfied	169	19	11-31	1	-	-	1	-	-
Dissatisfied	19	38	21-43	0.61	0.38, 0.99	0.04	0.67	0.38, 1.16	0.15
Believe that the hand problem was caused by work [e]									
No	112	19	13-31	1	-	-	1	-	-
Agree/strongly agree	76	23	10-42	0.62	0.46, 0.85	0.003	0.82	0.57, 1.17	0.28
Job is demanding on hands/wrists [a]									
No	35	16	6-27	1	-	-	1	-	-

For peer review only

Yes	155	20	13-38	0.61	0.42, 0.89	0.01	0.68	0.42, 1.12	0.13
Type of work [f]									
Non-manual	119	18	9-31	1	-	-	1	-	-
Manual	70	23.5	14-41	0.66	0.48, 0.89	0.01	0.97	0.57, 1.64	0.90
Work involves target-driven pay [g]									
No	149	19	12-31	1	-		1	-	-
Yes	31	22	13-45	0.61	0.41, 0.91	0.02	0.97	0.59, 1.61	0.91
Duration of computer use at work (hours) [g]									
<1	69	28	17-42	1	-	-	1	-	-
>1 to <4	33	16	10-31	2.20	1.43, 3.38	<0.001	1.01	0.56, 1.81	0.98
≥4	84	16	7-27	2.38	1.67, 3.38	<0.001	1.85	1.08, 3.16	**0.03**
Work involves lifting or carrying ≥10 kg [g]									
No	108	18.5	11-30	1	-	-	1	-	-
Yes	77	24	13-40	0.61	0.42, 0.86	0.01	0.80	0.48, 1.33	0.39
Work involves pushing/pulling a heavy weight [g]									
No	104	16	8.5-28.5	1	-	-	1	-	-
Yes	83	26	16-42	0.51	0.37, 0.70	<0.001	0.97	0.61, 1.55	0.90

Total sample size n=195. All analyses were adjusted for age and sex. All significant variables in the univariable analyses (p<0.05) were entered into the multivariable model.

[a.] Reported on a 0-10 scale, dichotomised to no (0-6) and yes (7-10)[13].

[b.] CTS-6 score [14] with data driven tertiles.

[c.] Michigan Hand Questionnaire work performance subscale scored from 0-100, higher scores indicate better perceived work performance[17]. Data driven tertiles.

[d.] Reported on a 5-point scale, dichotomised to satisfied (very satisfied/satisfied/fairly satisfied) and dissatisfied (dissatisfied/very dissatisfied).

[e.] Reported on a 5-point scale and dichotomised to agree/strongly agree and neither agree nor disagree/disagree/disagree strongly[22].

[f.] Classified using the Office for National Statistics Standard Occupational Classification 2010[18, 19].

[g.] As part of the normal working day[23].

For peer review only

Clinical outcomes after CTR are shown in Table 5. Using the definition outlined in the methods, a total of 46 participants (24%) were identified as having at least one poor outcome (CTS symptoms that were worse, unchanged or only slightly better; scar symptoms that were at least fairly troublesome; required post-operative antibiotics or had additional time off work after first return). Of these participants, the majority (n=38, 83%) reported only a single component of poor outcome. Three individuals defined as having a poor outcome had not returned to work at the point of last follow-up (as compared with two individuals in the rest of the study sample). For those who had returned to work, the median duration of work absence for those with a poor outcome was 22 days (IQR 12-42) compared with 19 days (IQR 12-32) for those without (Figure 2). This difference was not significant (Wilcoxon rank-sum, p=0.24).

TABLE 5. Clinical outcomes after carpal tunnel release

	**Mean score (standard deviation)**	
	Before surgery	12 weeks after surgery
CTS-6 (operated hand) [a]	3.3 (0.87)	1.2 (0.54)
MHQ function (operated hand) [b]	50 (22.1)	79 (19.4)
MHQ satisfaction with function (operated hand) [b]	38 (25.7)	82 (21.3)
MHQ bilateral activities of daily living [b]	69 (23.7)	88 (13.8)
MHQ activities of daily living (operated hand) [b]	65 (28.1)	87 (18.5)
	**Number of participants (%)**	
Global rating of change score		
Completely cured	-	65 (33.3)
Much better	-	98 (50.3)
Slightly better	-	13 (6.7)
Unchanged	-	2 (1.0)
Worse	-	5 (2.6)
Scar symptom severity	-	
Not at all troublesome	-	99 (50.8)
A little troublesome	-	63 (32.3)
Fairly troublesome	-	18 (9.2)
Very troublesome	-	2 (1.0)
Unbearable	-	0
Required post-operative antibiotics	-	10 (5.1)
Additional sick leave after first returning to work	-	12 (6.2)

Grey shading indicates the categories which were combined to define a poor surgical outcome.
[a]. CTS-6 assessment of carpal tunnel syndrome symptoms[14]. Range 1-5: 1 equals no symptoms.
[b]. MHQ Michigan Hand Questionnaire[17]. Range 0-100: 100 equals no deficit or dissatisfaction.

For peer review only - http://bmjopen.bmj.com/site/about/guidelines.xhtml

Figure 2. Duration of work absence after carpal tunnel release

There was no significant difference in the prevalence of a poor outcome among those who returned to work within or after seven days of CTR (20% versus 24%, Chi^2 p=0.63). Similarly, there was no significant difference in the prevalence of a poor outcome among those who returned to work within or after 14 days (19% versus 25%, Chi^2 p=0.31), or within or after 28 days of CTR (21% versus 27%, Chi2 p=0.33).

DISCUSSION

In this multi-site prospective cohort study, the median duration of work absence was 20 days (range 1-99), a duration similar to that reported by other European studies[24]. Earlier return to work was associated with typing for ≥4 hours at work (as compared with more physical workplace tasks) and undergoing surgery in primary care (as compared with secondary care or private practice). Pre-operative expectations about return to work were important significant predictors of actual return to work times. We found no evidence of poorer clinical outcomes in the first 12 weeks amongst those who returned to work earlier. At each time point, fewer manual workers had returned to work than non-manual workers and fewer employed workers had returned than self-employed (Figure 2). Both findings have been reported previously[10, 11, 25, 26], however, neither the type of work (manual/non-manual) nor the type of work contract (employed/self-employed) were significantly associated with the duration of work absence in the mutually adjusted model. These results illustrate the importance of considering the range of relevant demographic, clinical and occupational factors, which may have been confounders, moderators or mediators in previous studies.

For peer review only

Five variables remained statistically significantly associated with longer duration of work absence in the final model. Two were occupational factors: infrequent computer use and availability of sick pay. Cowan et al.[10], recorded earlier return to work after CTR for desk-based workers and we have shown previously that UK hand surgeons and hand therapists report that they advise earlier return to desk-based workers[6]. The relationship between longer duration of work absence and availability of sick pay has also be reported previously for those with and without worker's compensation[9, 24]. It is plausible that financial necessity is driving earlier return to work for those with limited sick pay. Alternatively, those with access to paid leave might choose to take the maximum available duration.

Only one clinical factor was significantly associated with the duration of work absence: participants who had their CTR surgery in primary care were more likely to return to work earlier than those whose procedure took place in an NHS hospital setting. One possible reason is that patients with more complex disease may be more commonly referred to secondary, rather than primary, care for their CTR and these patients may require longer off work after their surgery. However, in the current study the proportion and degree of comorbidities, and the extent of pre-operative symptoms reported by participants were similar across all settings. Another possibility is that the patients' expectations of the surgery may be different: CTR performed in a primary care setting might be perceived by patients as being more minor than surgery in a hospital operating theatre. Alternatively, the general practice surgeons may have recommended earlier return to work than those based in a hospital, although the median expected duration of work absence for participants in the current study was the same across all settings. The inclusion of CTR performed in primary care is a strength of the study. CTR and

For peer review only - http://bmjopen.bmj.com/site/about/guidelines.xhtml

other surgical procedures, such as vasectomy and minor skin surgery, are regularly performed by trained General Practitioners in the UK[27].

Only one demographic factor was statistically significant: women were more likely to take longer to return to work than men. Whilst we found inconsistent data about the effect of gender on return to work after CTR in the literature[28, 29], female gender has been associated with longer periods of work absence for other health conditions[30]. Researchers should continue to include sex as a covariate in analyses of return to work outcomes, although there is currently insufficient evidence to support any difference in return to work recommendations after CTR based on sex. Further qualitative investigation is required in order to understand the context for this.

Finally, those who expected to return to work more quickly were significantly more likely to do so. It has been shown previously that patient expectations are a prominent determinant of return to work time, or other return to work outcomes, for musculoskeletal or mental health conditions[30-32]. The role of expectations on outcomes, including the expected and actual timing for return to work and driving after hand and wrist surgery, requires further exploration, particularly because expectations are a potentially modifiable characteristic which could be influenced by the advice provided by clinicians pre-operatively.

In total, approximately a quarter of participants in this study were considered to have a poor outcome using our composite definition. We chose to use a multi-component definition which was deliberately very stringent to minimise the chances of missing any harm caused by early return to work. Our rates of poorer outcomes were in fact similar to those reported in other

For peer review only - http://bmjopen.bmj.com/site/about/guidelines.xhtml

CTR populations[20, 33, 34]. Importantly, we found no relationship between earlier return to work and occurrence of poor outcomes within 12 weeks of CTR in this cohort study. We acknowledge that a longer follow-up duration would have aided the assessment of post-operative symptom resolution, however this was not possible with the resources available and was not a primary objective of the study.

There are a number of limitations of the current study, including the reliance on self-reported data. Work absence is not logged on a national database in the UK and therefore could only be obtained through self-report. To minimise errors of recall, date of return to work was determined contemporaneously. The recall duration for measures of function and symptoms was limited to a maximum of four weeks, consistent with the outcome measures used[14, 17]. We set out to recruit a large sample of working-aged adults undergoing CTR. Our prospectively recruited sample from 16 sites is one of the largest reported in the literature to date, with a good follow up response rate (79%), but it remains possible that we were under-powered to detect some of the factors which may have been associated with delayed return to work. Specifically, this could result where some levels of categorical variables of interest have lower prevalence, for example, the type of work contract (>80% of participants reported that they had a permanent work contract, compared with ~15% who were self-employed).

Following our *a priori* analysis plan, the association between each baseline variable and the duration of work absence was individually assessed in separate age- and sex-adjusted analyses. Only those variables which reached significance at the 5% level ($p<0.05$) were included in the multivariable model. In order to test the stability of our model, and to identify whether any potential associations had been missed, this was tested using 1% and 20% cut-offs. In both test

For peer review only - http://bmjopen.bmj.com/site/about/guidelines.xhtml

scenarios, the findings were similar to those presented in our final model (Table 4), suggesting that our model is robust. However, we acknowledge that alternative methods of selecting variables for inclusion (such as forward inclusion or backward elimination) may have yielded slightly different results, particularly for variables that were close to our significance cut-off of 5%.

The findings may not be generalisable to working populations in regions outside of central and southern England and Wales or employed in other industries. Steps were taken to include the main settings where CTR is performed in the UK, but we acknowledge that CTR may also be performed by other specialities. Individuals who chose to participate in the study may not be fully representative of the wider CTR population, and the observed differences between those who completed the study and those who were lost to follow-up (younger, poorer mental health, more likely manual workers) also limit generalisability.

Endoscopic CTR has been associated with earlier return to work than open CTR [35], however it was not possible to assess this in the current study. At present, endoscopic CTR is not routinely performed in the UK[6]. Anecdotally, most providers will not fund the extra cost of endoscopic CTR, which requires extra equipment, longer operating times and more experienced surgeons. Recruitment to the current study was not limited to patients undergoing open CTR, but no endoscopic procedures were performed during the study at any of our sites.

All participants were presumed to have CTS as diagnosed by their treating clinician. Many studies of CTS include nerve conduction study (NCS) findings as part of their eligibility criteria, although this was not possible in the current study because NCS are not routinely

For peer review only - http://bmjopen.bmj.com/site/about/guidelines.xhtml

recommended for pre-operative diagnosis of CTS in the UK[2]. Our eligibility criteria required that only people undergoing their first CTR were included and reported upon in this study (so that previous experiences with CTR were not potential confounders). However, more than three quarters of the cohort reported bilateral symptoms. The possible impact of persisting CTS symptoms in the non-operated hand on return to work also needs to be considered.

For the current study, we considered both occupational title and self-reported occupational exposures collected in a standardised questionnaire format[13, 18, 19, 23]. Categorisation based on job title and industry may not accurately reflect the physical and/or psychosocial aspects of job role. Furthermore, co-occurrence of occupational exposures may be more common in some types of jobs than in others, for example lifting >10kg and pushing or pulling a heavy weight.

There is a need for an agreed approach to identifying and recording key physical demands and psychosocial exposures of jobs to enable consistent exploration of their impact on work and clinical outcomes following surgery or other intervention. Approaches such as job exposure matrices[36] could facilitate this in future research.

In summary, this large multi-centre prospective cohort study investigated when participants return to work after CTR. Expectations about return to work (reported before surgery) were strongly associated with actual work absence, regardless of the job role or self-reported upper limb activities involved. Patient expectations can be influenced by many factors, but one of the most important is the advice provided by clinicians, in particular the surgeon. This suggests that clear, consistent advice could have an important effect on duration of sick leave. To date

For peer review only - http://bmjopen.bmj.com/site/about/guidelines.xhtml

there is no evidence-based guidance informing clinicians what to advise about returning to different types of work after CTR. Further research is required to reach a consensus and explore whether the provision of targeted, consistent and standardised advice can alter the expected duration of work absence, reducing unnecessary sick leave, without causing adverse effects on clinical outcomes.

REFERENCES

1. Jerosch-Herold C, Mason R, Chojnowski AJ. A qualitative study of the experiences and expectations of surgery in patients with carpal tunnel syndrome. *J Hand Ther* 2008;21(1):54-61 doi:10.1197/j.jht.2007.09.001.
2. British Orthopaedic Association. Commissioning guide: Treatment of carpal tunnel syndrome. London: Royal College of Surgeons; 2017.
3. American Academy of Orthopaedic Surgeons. Management of carpal tunnel syndrome evidence-based clinical practice guideline. Illinois: American Academy of Orthopaedic Surgeons; 2016.
4. Bebbington E, Furniss D. Linear regression analysis of Hospital Episode Statistics predicts a large increase in demand for elective hand surgery in England. *J Plast Reconstr Aesthet Surg* 2015;68(2):243-51 doi:10.1016/j.bjps.2014.10.011.
5. Burton CL, Chen Y, Chesterton LS et al. Trends in the prevalence, incidence and surgical management of carpal tunnel syndrome between 1993 and 2013: an observational analysis of UK primary care records. *BMJ Open* 2018;8(6) doi:10.1136/bmjopen-2017-020166.
6. Newington L, Francis K, Ntani G et al. Return to work recommendations after carpal tunnel release: a survey of UK hand surgeons and hand therapists. *J Hand Surg Eur* 2018;43(8):875-78 doi:10.1177/1753193418786375.
7. Black C. Working for a healthier tomorrow. London, UK: Health Work and Wellbeing Programme 2008.

For peer review only

8. Peters S, Johnston V, Hines S et al. Prognostic factors for return-to-work following surgery for carpal tunnel syndrome: a systematic review. *JBI Database Syst Rev Implement Rep* 2016;14(9):135-216 doi:10.11124/jbisrir-2016-003099.

9. Dunn JC, Kusnezov NA, Koehler LR et al. Outcomes following carpal tunnel release in patients receiving workers' compensation: a systematic review. *Hand* 2018;13(2):137-42 doi:10.1177/1558944717701240.

10. Cowan J, Makanji H, Mudgal C et al. Determinants of return to work after carpal tunnel release. *J Hand Surg Am* 2012;37(1):18-27

11. De Kesel R, Donceel P, De Smet L. Factors influencing return to work after surgical treatment for carpal tunnel syndrome. *Occup Med* 2008;58(3):187-90

12. Parot-Schinkel E, Roquelaure Y, Ha C et al. Factors affecting return to work after carpal tunnel syndrome surgery in a large french cohort. *Arch of Phys Med Rehabil* 2011;92(11):1863-69 doi:http://dx.doi.org/10.1016/j.apmr.2011.06.001.

13. Hansen TB, Dalsgaard J, Meldgaard A et al. A prospective study of prognostic factors for duration of sick leave after endoscopic carpal tunnel release. *BMC Musculoskelet Disord* 2009;10:144

14. Atroshi I, Lyren PE, Ornstein E et al. The six-item CTS symptoms scale and palmar pain scale in carpal tunnel syndrome. *J Hand Surg Am* 2011;36(5):788-94 doi:10.1016/j.jhsa.2011.02.021.

15. Calfee RP, Dale AM, Ryan D et al. Performance of simplified scoring systems for hand diagrams in carpal tunnel syndrome screening. *J Hand Surg Am* 2012;37(1):10-17 doi:10.1016/j.jhsa.2011.08.016.

16. Katz JN, Stirrat CR. A self-administered hand diagram for the diagnosis of carpal tunnel. *J Hand Surg Am* 1990;15:360-63

17. University of Michigan Medical School. The Michigan Hand Questionnaire: http://mhq.lab.medicine.umich.edu/. Accessed 04/04/2019.

18. Office for National Statistics. Standard occupational classification for the UK: https://www.ons.gov.uk/methodology/classificationsandstandards/standardoccupationalclassificationsoc/soc2010. Accessed 08/06/2018.

19. Warwick Institute for Employment Research. Cascot: Computer assisted structured coding tool: https://warwick.ac.uk/fac/soc/ier/software/cascot/. Accessed 15/05/2019.

For peer review only

20. Jerosch-Herold C, Shepstone L, Houghton J et al. Prognostic factors for response to treatment by corticosteroid injection or surgery in carpal tunnel syndrome (PaLMS study): a prospective multi-centre cohort study. *Muscle Nerve* 2019;60(1):32-40 doi:doi:10.1002/mus.26459.

21. Ware J, Sherbourne C. The MOS 36-item short-form health survey (SF-36): I. conceptual framework and item selection. *Medical Care* 1992;30:473-83

22. Palmer K, Calnan M, Wainwright D et al. Upper limb pain in primary care: health beliefs, somatic distress, consulting and patient satisfaction. *Family Practice* 2006;23(6):609-17 doi:10.1093/fampra/cml047.

23. Karasek R, Brisson C, Kawakami N et al. The Job Content Questionnaire (JCQ): an instrument for internationally comparative assessments of psychosocial job characteristics. *Journal Occup Health Psychol* 1998;3(4):322-55

24. Newington L, Stevens M, Warwick et al. Sickness absence after carpal tunnel release: a systematic review of the literature. *Scand J Work Environ Health* 2018;44(6):557-67 doi:10.5271/sjweh.3762

25. Atroshi I, Larsson G-U, Ornstein E et al. Outcomes of endoscopic surgery compared with open surgery for carpal tunnel syndrome among employed patients: randomised controlled trial. *BMJ* 2006;332(7556):1473 doi:10.1136/bmj.38863.632789.1F.

26. Chaise F, Bellemere P, Fril JP et al. Return-to-work interval and surgery for carpal tunnel syndrome. Results of a prospective series of 233 patients. *J Hand Surg Br* 2004;29(6):568-70

27. Association of Surgeons in Primary Care. Surgical proceedures in primary care: https://www.aspc-uk.net/. Accessed 24/11/2020

28. Atroshi I, Zhou C, Joud A et al. Sickness absence from work among persons with new physician-diagnosed carpal tunnel syndrome: a population-based matched-cohort study. *PLoS One* 2015;10(3):e0119795 doi:10.1371/journal.pone.0119795.

29. Bekkelund SI, Pierre-Jerome C, Torbergsen T et al. Impact of occupational variables in carpal tunnel syndrome. *Acta Neurol Scand* 2001;103(3):193-97

30. Cancelliere C, Donovan J, Stochkendahl MJ et al. Factors affecting return to work after injury or illness: best evidence synthesis of systematic reviews. *Chiropr Man Ther* 2016;24(1):32-32 doi:10.1186/s12998-016-0113-z.

For peer review only

31. Claréus B, Renström EA. Patients' return-to-work expectancy relates to their beliefs about their physician's opinion regarding return to work volition and ability. *J Pain Res* 2019;12:353-62 doi:10.2147/JPR.S179061.

32. Heymans MW, de Vet HC, Knol DL et al. Workers' beliefs and expectations affect return to work over 12 months. *Journal Occup Rehabil* 2006;16(4):685-95 doi:10.1007/s10926-006-9058-8.

33. Bland JDP. Treatment of carpal tunnel syndrome. *Muscle Nerve* 2007;36(2):167-71 doi:10.1002/mus.20802.

34. Louie D, Earp B, Blazar P. Long-term outcomes of carpal tunnel release: a critical review of the literature. *Hand* 2012;7(3):242-46 doi:10.1007/s11552-012-9429-x.

35. Li Y, Luo W, Wu G et al. Open versus endoscopic carpal tunnel release: a systematic review and meta-analysis of randomized controlled trials. *BMC Musculoskelet Disord* 2020;21:272 doi:10.1186/s12891-020-03306-1

36. Dale AM, Zeringue A, Harris-Adamson C et al. General population job exposure matrix applied to a pooled study of prevalent carpal tunnel syndrome. *Am Journal Epidemiol* 2015;181(6):431-39

Conflicts of interest

LN was supported by a National Institute for Health Research (NIHR) Doctoral Research Fellowship, grant number DRF-2015-08-056. GN was supported by funding from the MRC Versus Arthritis Centre for Musculoskeletal Health and Work. The authors declare no other conflicts of interest.

Funding

LN was supported by a National Institute for Health Research (NIHR) Doctoral Research Fellowship, grant number DRF-2015-08-056. GN was supported by funding from the MRC Versus Arthritis Centre for Musculoskeletal Health and Work. The views expressed are those of

For peer review only

the authors and not necessarily those of the funders, the NHS, the NIHR or the Department of Health.

Informed consent

Written informed consent was obtained from the patient(s) for their anonymised information to be published in this article.

Ethics approval

Full ethics approval was granted by the NHS Health Research Authority (IRAS 209840: 16/WA/0390) and University of Southampton (ERGO 25757) Ethics Committees.

Contributorship

The study was devised by LN, KWB, JA, DW and DC. LN completed data collection and analysis, with assistance from GN and KWB. All authors contributed to the interpretation of the data. LN wrote the first draft of the manuscript. All authors reviewed and edited the manuscript and approved the final version.

Acknowledgements

The authors would like to thank and acknowledge all the study participants who gave up their time to take part in this study. Thank you also to the Patient Advisory Group for their contribution to the development of the research question and study material, and their thoughtful feedback on the preliminary findings. Thank you to Prof David Coggon for his assistance with the initial research idea, and to the Association of Surgeons in Primary Care and British Society for Surgery of the Hand for supporting the study.

For peer review only

Thank you to the study sites and local principal investigators: Jeremy Bland at East Kent Hospitals University NHS Foundation Trust; Darren Roberts at Portsmouth Hospitals NHS Trust; Mike Taylor and Claire Zweifel at Mid Essex Hospital Services NHS Trust; Frances Clark at Tollgate Clinic; Will Mason at Gloucester Hospitals NHS Foundation Trust; Vasileios Kefalas and Petros Mikalef at Care UK Southampton NHS Treatment Centre; Peter Sharpe at Salisbury Medical Practice; David Warwick at University Hospital Southampton NHS Foundation Trust and Nuffield Health Wessex Hospital; Alistair Phillips at Southern Health NHS Foundation Trust; Nanda Pillai at St Luke's Surgery, Walsall; Tim Halsey and Effie Katsarma at Chelsea and Westminster Hospital NHS Foundation Trust; Claire Middleton at Royal Berkshire NHS Foundation Trust; Duncan Avis and Ian MacLeod at Hampshire Hospital NHS Foundation Trust; Nick Gape and Lisa Small at Cardiff and Vale University Health Board; Kristin Francis at HCA Healthcare London Hand and Wrist Unit; and to all local team members who helped set up and recruit to the REACTS study.

For peer review only

Figure 1. Participant numbers for each stage of the cohort study

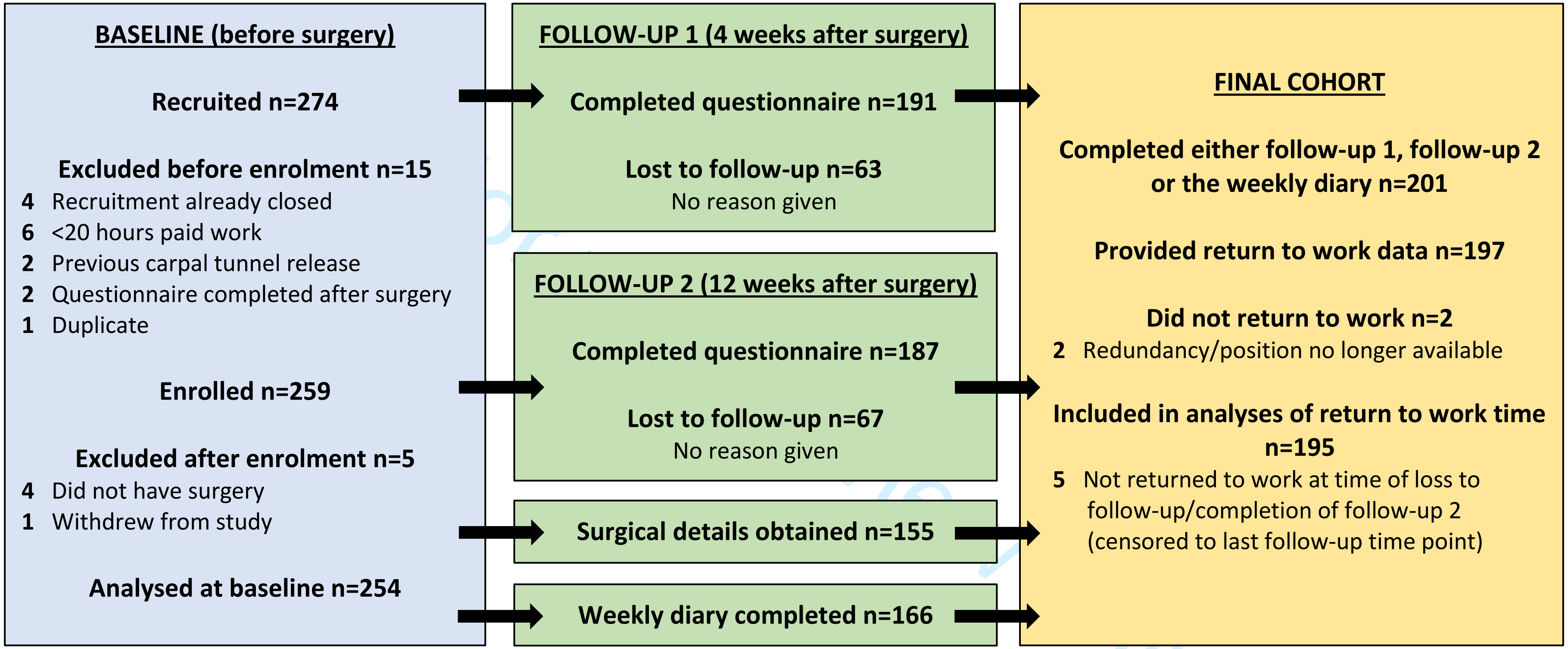

For peer review only - http://bmjopen.bmj.com/site/about/guidelines.xhtml

Figure 2. Duration of work absence after carpal tunnel release

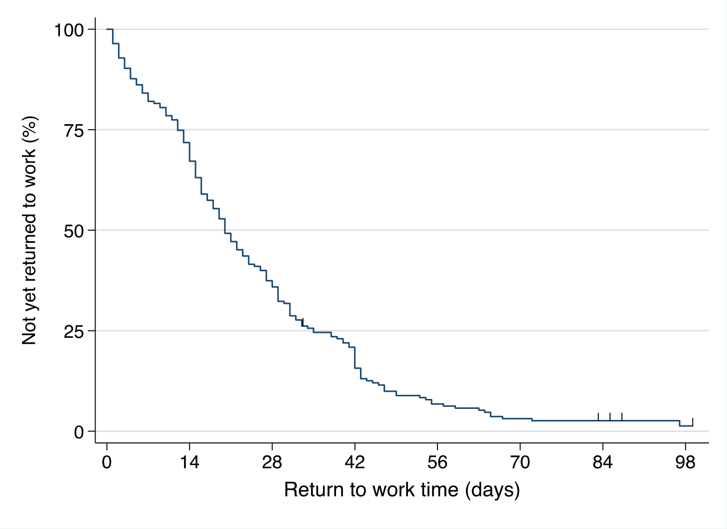

A. Whole cohort

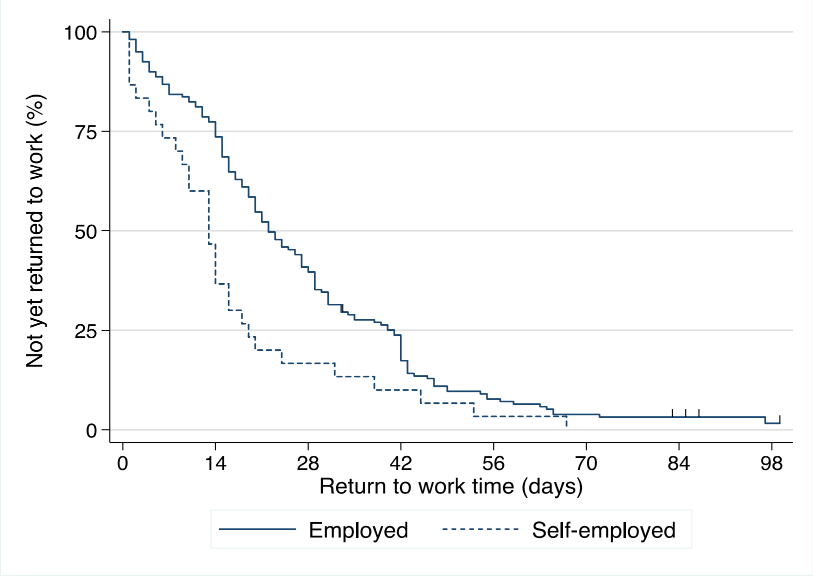

B. Employed versus self-employed workers

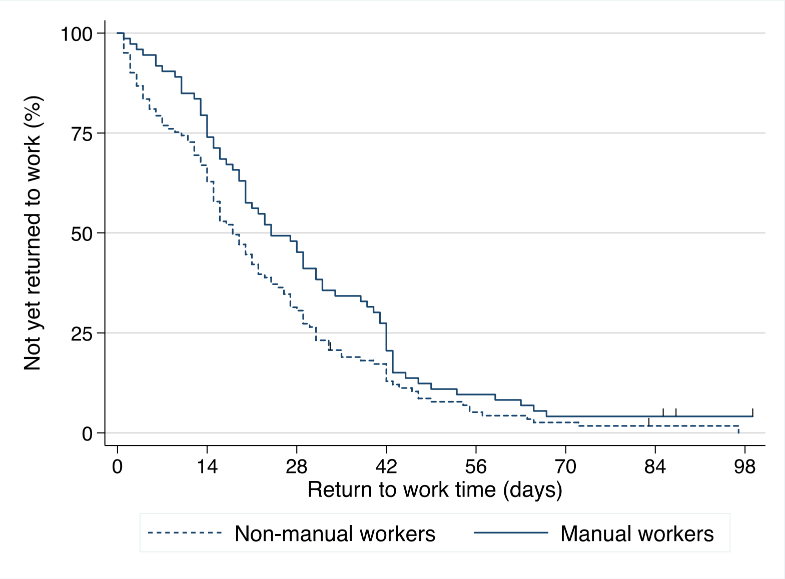

C. Non-manual versus manual workers

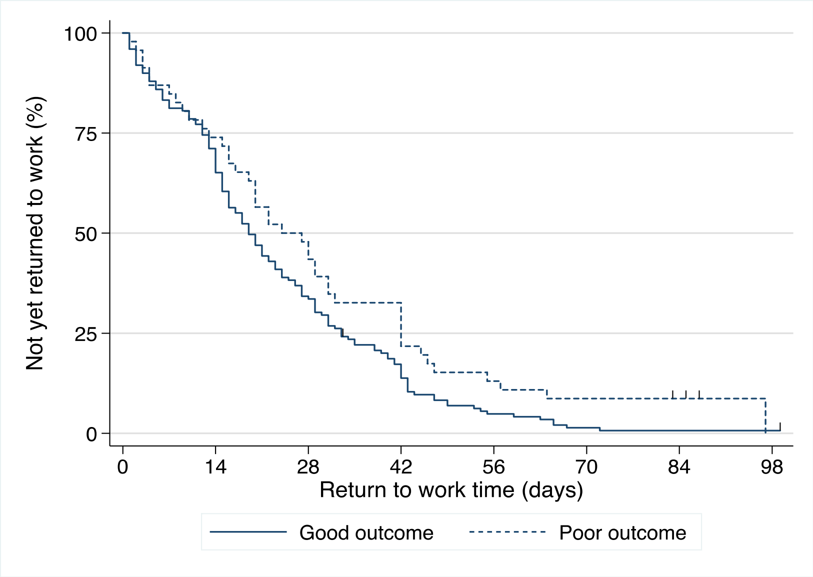

D. With versus without a poor outcome

Vertical lines indicate right censoring for those who had not returned to work at the point of last follow-up

Development of the REACTS study questionnaire: Reasoning for item inclusion

Questions were chosen to capture information on variables that might be expected to influence return to work after CTR.

Demographic factors

General demographic information was requested including date of birth, sex and hand dominance. Other studies have found that older age was associated with poorer work outcomes after CTR [1, 2], while no clear sex effect has been shown [3, 4]. Hand dominance in relation to side of surgery is rarely reported in CTR studies, however, surgery to the non-dominant hand has been linked to quicker and more complete resolution of CTS symptoms [5].

Carpal tunnel release planning

Information was collected on the expected date of CTR, side of surgery, availability of occupational health services and the patient's expectations about time off work post-surgery. Expected duration of work absence has been identified as a determinant of return to work time in previous CTR studies [2, 6].

Occupational factors

Participants were asked to list their main occupation and the industry in which they work (examples were provided to facilitate the response). This information was processed using the UK Office for National Statistics Standard Occupational Classification [7] and Computer Assisted Structured Coding tool (Cascot) [8] to generate manual and non-manual categories. Cases where the coding match was confirmed as less than 64% were reviewed by the lead researcher and coded by hand [8]. This was checked by the department data manager and any queries resolved through discussion.

Participants were asked to categorise their employment type as: employed (permanent contract), employed (temporary/renewable contract), zero hours contract and self-employed. Our systematic review found earlier return to work times for self-employed individuals compared to those who were employed [9]; but this was only investigated in two

For peer review only - http://bmjopen.bmj.com/site/about/guidelines.xhtml

studies [10, 11]. The additional sub-categories for employment type listed above and a separate question about sick-pay entitlement were included to allow the impact of work contract type to be explored [12]. Participants were also asked how many hours they usually worked each week and over how many days; this information was collected for the participant's main job and any other routine paid work. The total number of work hours per week was calculated by combining the hours for main and additional jobs.

Occupational activities that load the upper limb and potential work stressors were asked as a series of yes/no questions following the format of a recent multi-centre RCT exploring management of non-specific distal arm pain [13]. These questions originated in the Job Content Questionnaire, designed to assess psychological and physical aspects of work [14]. Activities included: computer use, tasks involving repeated wrist/finger movement, holding vibrating tools, lifting more than 5 or 10kgs, pushing/pulling a heavy weight, working with the neck flexed or rotated, and driving. Our systematic review found that manual workers took longer to return to work than non-manual workers [9] and these questions were used to determine the self-reported level of upper limb manual activity involved in each participant's job.

Potential psychosocial work stressors were also assessed. These included piecemeal work, activity targets and bonuses, and tight deadlines. As the first three items all concerned payment for results, these were combined for the analyses. Participants were also asked whether they found their main job demanding on their hands/wrist and whether their boss/colleagues were supportive. Both questions were scored on a 0-10 scale as reported previously in a study of sick leave duration after endoscopic CTR [15]. These were dichotomised as supportive (7-10) and neutral/unsupportive (0-6). A question about general job satisfaction was also included later in this section of the questionnaire, with the Likert response options: very satisfied, satisfied/fairly satisfied, dissatisfied and very dissatisfied [15, 16]. The last two options were condensed to give three categories for the analyses.

To assess self-reported work function, participants were asked to complete the work performance section of the Michigan Hand Questionnaire (MHQ) [17]. This patient reported outcome measure is frequently used in upper limb clinical practice and research and has

For peer review only - http://bmjopen.bmj.com/site/about/guidelines.xhtml

been validated for use with CTS and CTR populations [18]. Permission was granted for the MHQ to be used in the study. The questionnaire asked participants to recall how much difficulty they had with general work tasks over the past four weeks in relation to problems with their hands/wrists, for example: needing to shorten their working day, taking longer to complete tasks or needing to take breaks. Using the standard scoring, each question was completed on a Likert scale of: always, often, sometimes, rarely and never, and combined to give a score from 0-100, with 100 representing no problems with work functioning [19]. Participants were also asked whether they had taken any periods of sickness absence from work over the previous four weeks, both related to the hand/wrist problem, or for any other problem.

General health

Seven general health questions were included to capture information on comorbidities, physical and mental health and somatisation. Self-reported health was assessed using the first SF-36 question: In general, would you say your health is – excellent, very good, good, fair, poor [20]. This was taken from the original SF-36 version, which is free from licence charges and was dichotomised as excellent/very good/good and fair/poor for the analyses.

Participants were asked their height and weight to enable the calculation of BMI (body mass index; weight in kilograms/height in metres squared). This was categorised using standard WHO classification: underweight (BMI <18.5), normal weight (BMI 18.5-24.9), overweight (BMI 25.0-29.9) and obese (BMI ≥30.0) [21]. Smoking status was categorised as those who have never smoked regularly, those who have smoked in the past and those who regularly smoke, with the latter two categories combined for the analyses. Previous studies have found that obesity (BMI ≥30) was linked to poorer work outcomes [1] and smoking was linked to poorer clinical outcomes after CTR [22].

A list of common health problems and their impact on general activities was assessed using the Self-Administered Comorbidity Questionnaire [23]. Participants were asked first to select whether they have any of the 14 medical conditions and if so whether this limited their activities. All medical conditions were worded in an accessible format, as evaluated by the patient advisory group. Responses were analysed as the number of comorbidities and

For peer review only - http://bmjopen.bmj.com/site/about/guidelines.xhtml

the number of disabling comorbidities using the scale: 0, 1, ≥2. Mental health was assessed using the mental health and vitality questions from the licence-free version of the SF-36 [20]. One modification was made to change the wording of the question 'Did you feel full of pep?' to 'Did you feel full of get-up-and-go?' for the UK rather than US setting. The questions were used to calculate the summary score from 0-100 (where 100 represents no disability).

Somatisation was assessed using a subset of five questions from the Four-Dimensional Symptom Questionnaire [24] as previously reported in UK cohort studies of health and employment [25] and upper limb pain in primary care [26]. The number of symptoms that were rated by the participant as at least moderately distressing were used to create the analysis categories of: 0, 1, ≥2 symptoms.

Hand and wrist symptoms and function

Katz & Stirrat hand diagrams were included for the participants to indicate where on their hand(s) they experience pain and/or tingling and numbness [27]. This self-administered tool can be used clinically as part of the CTS diagnosis process using the scoring system modified by Calfee et al. [28]. A question on symptom duration was also included and categorised as less than 3 months, 3-6 months, 6-12 months and more than a year. This was dichotomised to ≤1 year and >1 year for analysis. All participants were expected to have clinically diagnosed CTS as they were undergoing CTR, but participants were asked to answer for both hands, so that symptoms in the non-operated hand were also assessed. This was used to define bilateral or unilateral symptoms. Hand diagram scores were dichotomised according to a stringent definition of CTS (classic and probable) and unlikely CTS (possible and unlikely).

The CTS-6 questionnaire was included to assess the severity of CTS symptoms [29]. This tool is a shorter version of the Boston Carpal Tunnel Questionnaire [30] and has been assessed for use pre- and post CTR surgery. The six questions explore the severity of pain and numbness, whether this occurs during the daytime or at night, and whether this wakes the individual. Participants were asked to complete these questions separately for each hand using a 5-point Likert scale. Using the standard scoring criteria, responses for each item

For peer review only - http://bmjopen.bmj.com/site/about/guidelines.xhtml

were combined to give a mean score ranging from 1-5, with 5 representing the highest severity of symptoms. If one response was missing (per hand) this was imputed using the mean score of the remaining responses [29].

Hand function was assessed using the MHQ sub-sections on unilateral hand function (asked for each hand), satisfaction with hand function (asked for each hand), and ability to perform unilateral and bilateral activities of daily living (ADLs) [17]. The MHQ summary question relating to the level of satisfaction with the appearance of each hand was also included. All questions were scored on the 5-point Likert scales provided and used the standard wording and scoring to enable comparison with other study populations. Possible scores for each sub-section range from 1-100, with 100 representing no problems or the highest level of satisfaction. Missing data were imputed according to the MHQ guidelines, which allow the scale to be calculated if more than 50% of the questions for each sub-section have been completed [19].

Health beliefs

The remaining questions related to health beliefs. Beliefs about the cause of symptoms and likely prognosis have been identified as key themes in health-seeking behaviour for CTS [31] and upper limb pain [26], and blaming oneself for the hand problem has been associated with long durations of sick leave after endoscopic CTR [15]. The participant’s expectations for being able to use the affected hand normally within 3 months of surgery, fear of long-term hand problems, blaming oneself for the hand problem and the perceived level of support available from friends and family were assessed. Responses were rated on a 0-10 scale as reported by Hansen et al. [15]. All responses were converted to a unidirectional scale with 10 being the best outcome, and were dichotomised as neutral/negative response 0-6 and positive response 7-10.

Participants were also asked to agree/disagree (via a 5-point Likert scale) with a series of seven questions about the believed cause of their symptoms. Using previously reported methods, the responses were dichotomised to those who agreed (agree/strongly agree) and those who did not agree (neither agree nor disagree/disagree/disagree strongly) with each statement [26]. The first two questions were combined to generate six items: 1) I think I was

For peer review only - http://bmjopen.bmj.com/site/about/guidelines.xhtml

born with a weakness in this part of my body/problems like this run in my family; 2) my problem was caused by work; 3) my problem probably wasn't caused by work, but work made it worse; 4) I have a lot of stress in my life and that has made my problem a lot worse; 5) a lack of exercise probably contributed to my problem; 6) as you get older, parts of the body wear out and problems like mine are likely [26].

Finally, the abbreviated Pain Catastrophizing Scale was included, which provides insight about the participant's pain beliefs [32]. Responses were dichotomised to those who reported catastrophising pain thoughts and feelings to at least a moderate degree in response to all questions, and those who did not.

References

1. Parot-Schinkel E, Roquelaure Y, Ha C et al. Factors affecting return to work after carpal tunnel syndrome surgery in a large french cohort. *Arch of Phys Med Rehabil* 2011;92(11):1863-69.
2. Peters S, Johnston V, Hines S et al. Prognostic factors for return-to-work following surgery for carpal tunnel syndrome: a systematic review. *JBI Database of Systematic Reviews and Implementation Reports* 2016;14(9):135-216.
3. Bekkelund SI, Pierre-Jerome C, Torbergsen T et al. Impact of occupational variables in carpal tunnel syndrome. *Acta Neurol Scand* 2001;103(3):193-97.
4. Atroshi I, Zhou C, Joud A et al. Sickness absence from work among persons with new physician-diagnosed carpal tunnel syndrome: a population-based matched-cohort study. *PLoS One* 2015;10(3):e0119795.
5. Tang Q, Lai W, Tay S. The effect of hand dominance on patient-reported outcomes of carpal tunnel release in patients with bilateral carpal tunnel syndrome. *Journal of Hand Surgey Asian Pacific Volume* 2017;22(3):303-08.
6. Cowan J, Makanji H, Mudgal C et al. Determinants of return to work after carpal tunnel release. *J Hand Surg Am* 2012;37(1):18-27.
7. Office for National Statistics. Standard occupational classification for the UK: https://www.ons.gov.uk/methodology/classificationsandstandards/standardoccupationalclassificationsoc/soc2010. Accessed 08/06/2018.
8. Warwick Institute for Employment Research. Cascot: Computer assisted structured coding tool https://warwick.ac.uk/fac/soc/ier/software/cascot/. Accessed 15/05/2019.
9. Newington L, Stevens M, Warwick D et al. Sickness absence after carpal tunnel release: a systematic review. *Scandinavian Journal of Work, Environment and Health* 2018;44(6):557-67.
10. De Kesel R, Donceel P, De Smet L. Factors influencing return to work after surgical treatment for carpal tunnel syndrome. *Occup Med* 2008;58(3):187-90.

For peer review only - http://bmjopen.bmj.com/site/about/guidelines.xhtml

11. Chaise F, Bellemere P, Fril JP et al. Return-to-work interval and surgery for carpal tunnel syndrome. Results of a prospective series of 233 patients. *J Hand Surg Br* 2004;29(6):568-70.
12. Tran M, Sokas RK. The gig economy and contingent work: an occupational health assessment. *Journal of Occupational and Environmental Medicine* 2017;59(4):E63-E66.
13. Jones GT, Mertens K, Macfarlane GJ et al. Maintained physical activity and physiotherapy in the management of distal upper limb pain - a protocol for a randomised controlled trial (the arm pain trial). *BMC Musculoskelet Disord* 2014;15:71.
14. Karasek R, Brisson C, Kawakami N et al. The Job Content Questionnaire (JCQ): an instrument for internationally comparative assessments of psychosocial job characteristics. *Journal of occupational health psychology* 1998;3(4):322-55.
15. Hansen TB, Dalsgaard J, Meldgaard A et al. A prospective study of prognostic factors for duration of sick leave after endoscopic carpal tunnel release. *BMC Musculoskelet Disord* 2009;10:144.
16. D'Angelo S, Coggon D, Harris EC et al. Job dissatisfaction and the older worker: baseline findings from the Health and Employment After Fifty study. *Occupational and environmental medicine* 2016;73(8):512-19.
17. Chung KC, Pillsbury MS, Walters MR et al. Reliability and validity testing of the Michigan Hand Outcomes Questionnaire. *J Hand Surg Am* 1998;23(4):575-87.
18. Hadi M, Gibbons E, Fitzpatrick R. A structured review of patient-reported outcome measures for procedures for carpal tunnel syndrome. UK: Patient-Reported Outcome Measures Group, University of Oxford; 2011.
19. University of Michigan Medical School. The Michigan Hand Questionnaire: http://mhq.lab.medicine.umich.edu/. Accessed 04/04/2019.
20. Ware J, Sherbourne C. The MOS 36-item short-form health survey (SF-36): I. conceptual framework and item selection. *Medical Care* 1992;30:473-83
21. World Health Organization. Obesity: preventing and managing the global epidemic. Report of a WHO consultation. *World Health Organization technical report series* 2000;894:1-253.
22. Dahlin E, Zimmerman M, Bjorkman A et al. Impact of smoking and preoperative electrophysiology on outcome after open carpal tunnel release. *Journal of Plastic Surgery & Hand Surgery* 2017;51(5):329-35.
23. Sangha O, Stucki G, Liang MH et al. The self-administered comorbidity questionnaire: a new method to assess comorbidity for clinical and health services research *Arthritis & Rheumatism* 2003;49(2):156-63.
24. Terluin B, van Marwijk HW, Adèr HJ et al. The Four-Dimensional Symptom Questionnaire (4DSQ): a validation study of a multidimensional self-report questionnaire to assess distress, depression, anxiety and somatization. *BMC Psychiatry* 2006;6(1):34.
25. Palmer KT, Walker-Bone K, Harris EC et al. Health and Employment after Fifty (HEAF): a new prospective cohort study. *BMC public health* 2015;15:1071.
26. Palmer K, Calnan M, Wainwright D et al. Upper limb pain in primary care: health beliefs, somatic distress, consulting and patient satisfaction. *Family Practice* 2006;23(6):609-17.

For peer review only - http://bmjopen.bmj.com/site/about/guidelines.xhtml

27. Katz JN, Stirrat CR. A self-administered hand diagram for the diagnosis of carpal tunnel. *J Hand Surg Am* 1990;15:360-63.
28. Calfee RP, Dale AM, Ryan D et al. Performance of simplified scoring systems for hand diagrams in carpal tunnel syndrome screening. *J Hand Surg Am* 2012;37(1):10-17.
29. Atroshi I, Lyren PE, Ornstein E et al. The six-item CTS symptoms scale and palmar pain scale in carpal tunnel syndrome. *J Hand Surg Am* 2011;36(5):788-94.
30. Levine DW, Simmons BP, Koris MJ et al. A self-administered questionnaire for the assessment of severity of symptoms and functional status in carpal tunnel syndrome. *J Bone Joint Surg Am* 1993;75:1585-92.
31. Martin H. Patients' health beliefs and adaptation to carpal tunnel syndrome based on duration of symptomatic presentation. *J Hand Ther* 2007;20(1):29-35.
32. Bot AG, Becker SJ, Bruijnzeel H et al. Creation of the abbreviated measures of the Pain Catastrophizing Scale and the Short Health Anxiety Inventory: the PCS-4 and SHAI-5. *Journal of Musculoskeletal Pain* 2014;22(2):145-51.

For peer review only

For peer review only - http://bmjopen.bmj.com/site/about/guidelines.xhtml

University Hospital Southampton NHS
NHS Foundation Trust

REACTS

Arthritis Research UK MRC Medical Research Council centre for musculoskeletal health & work

UNIVERSITY OF Southampton

Site | | |

Please fill in today's date | | | | | |
d d m m y y

Before Your Carpal Tunnel Release Surgery

REACTS

Return to Employment After Carpal Tunnel Release Surgery (REACTS)

ln@mrc.soton.ac.uk | 023 8077 7624
Arthritis Research UK – MRC Centre for Musculoskeletal Health and Work
MRC Lifecourse Epidemiology Unit, University of Southampton
Southampton General Hosptial (MP 95), SO16 6YD

IRAS reference: 209840

For peer review only - http://bmjopen.bmj.com/site/about/guidelines.xhtml

For peer review only

University Hospital Southampton NHS Foundation Trust · REACTS · Arthritis Research UK / MRC centre for musculoskeletal health & work · University of Southampton

CONSENT FORM (IRAS reference: 209840)

You should complete this form after you have read the Participant Information Sheet.

REACTS: Return to employment after carpal tunnel release surgery

Thank you for considering taking part in this research. If you have any questions arising from the Participant Information Sheet, please ask the research team before you decide whether to take part.

Please initial the boxes if you agree with each statement

1. I have read the Participant Information Sheet (version 2.0; 06.12.16) and have had the opportunity to ask questions about the study.	☐
2. I meet the criteria for being involved in this study:<br>- Aged over 18 and referred for carpal tunnel release surgery<br>- Routinely work in paid employment for at least 20 hours per week<br>- Plan to return to work after carpal tunnel release surgery<br>- Have not previously had carpal tunnel release surgery on either hand<br>- Have not previously had a serious injury to the same wrist/hand that will have the carpal tunnel release operation	☐
3. I agree to take part in this research and agree for my data to be used for the purposes explained in the Participant Information Sheet (version 2.0; 06.12.16). I understand that this information will be handled in accordance with the terms of the UK Data Protection Act 1998.	☐
a. I agree for the REACTS research team to access pre-operative test results concerning my hand and wrist symptoms. No other information will be accessed.	☐
b. I agree for the REACTS research team to access my carpal tunnel release surgical record. No other information will be accessed.	☐
4. I understand that if I decide at any time during the research that I no longer wish to take part, I can notify the researchers and withdraw from the study immediately, without giving a reason. If I do, I understand that I can ask for any contribution I have already made to be removed from the study, up to the time when I have completed the final questionnaire.	☐

Signature ________________________________ Date ______ / ______ / ______

Name ________________________________ Phone ____________________
(please print) *(only to be used if we lose touch)*

Postal address ____

____

Email address ____
(please print)

For peer review only - http://bmjopen.bmj.com/site/about/guidelines.xhtml

University Hospital Southampton NHS Foundation Trust | REACTS | Arthritis Research UK MRC centre for musculoskeletal health & work | University of Southampton

ADDITIONAL QUESTIONS

Please circle one response for each question

I prefer to receive the next two questionnaires by	**Post**	**Email**	**Don't mind**
I prefer to receive correspondence about the study by	**Post**	**Email**	**Don't mind**
I would like to be notified of the findings from this research	**Yes**	**No**	
I am happy to be contacted about the next stage of the research, which will involve a one-off discussion with the lead researcher	**Yes**	**No**	
I am happy to be contacted about other studies related to this research	**Yes**	**No**	

When the research team receives your completed questionnaire and consent form, we will sign it below and return a copy to you for your records.

Researcher signature ____ Date ____ / ____ / ____

Researcher name ____

University of Southampton research supervisors:
Professor Karen Walker-Bone | Professor Jo Adams | Professor David Warwick

For peer review only

SECTION A: BACKGROUND

1 What is your date of birth?

d	d	m	m	y	y

2 Are you:

Male ☐ Female ☐ Other ☐

3 Are you:

Right handed ☐ Left handed ☐ Both ☐

4 Do you routinely carry out paid work for 20 hours or longer in a given week?

Yes ☐ No ☐

***If no,** thank you for your interest in our study, however, we are only looking for individuals who carry out paid work for at least 20 hours per week. You **do not** need to complete the rest of the questionnaire, but please return it using the pre-paid envelope provided.*

5 When do you expect to have your carpal tunnel surgery?

Please enter the exact date if known, or provide the approximate month and year if unsure.

d	d	m	m	y	y

6 Which hand will be operated on?

If both hands please answer Question 6.1; if one hand, please move on to Question 7.

Right ☐ Left ☐ Both ☐

6.1 If both hands, which side will be operated on first?

Right ☐ Left ☐ Both sides operated on the same day ☐ Unsure ☐

7 Do you have access to an occupational health service through your place of work?

Yes ☐ No ☐ Unsure ☐

8 Do you expect to take any time off work following your surgery?

If yes, please answer Question 8.1; if no, please move on to Question 9.

Yes ☐ No ☐ Unsure ☐

8.1 If you do expect to take time off work, how long do you expect to take?

Please complete using days, weeks or months; whichever applies.

Days ☐ Weeks ☐ Months ☐

For peer review only - http://bmjopen.bmj.com/site/about/guidelines.xhtml

SECTION A: BACKGROUND

9 Have you been given any information about your operation?
If yes, please answer Question 9.1; if no, please move on to Question 10.

Yes ☐ No ☐

9.1 If yes, who provided this information? Please tick all that apply.

a) Your surgeon or a member of the surgical team ☐
b) Hospital nurse ☐
c) GP or practice nurse ☐
d) Hand therapist ☐
e) Physiotherapist or occupational therapist ☐
f) Occupational health nurse or doctor ☐
g) Employer ☐
h) Friend or family member ☐
i) Internet ☐
j) Other *(please specify)* ☐

....

10 Have you been given any information about returning to work after your surgery?
If yes, please answer the rest of Question 10; if no, please move on to Question 11.

Yes ☐ No ☐

10.1 If yes, who provided this information? Please tick all that apply.

a) Your surgeon or a member of the surgical team ☐
b) Hospital nurse ☐
c) GP or practice nurse ☐
d) Hand therapist ☐
e) Physiotherapist or occupational therapist ☐
f) Occupational health nurse or doctor ☐
g) Employer ☐
h) Friend or family member ☐
i) Internet ☐
j) Other *(please specify)* ☐

....

10.2 What advice were you given?
If this advice came from more than one source, please indicate who advised what.

....
....
....
....
....
....
....

For peer review only - http://bmjopen.bmj.com/site/about/guidelines.xhtml

SECTION B: WORK

12 **What is your MAIN occupation at the moment (e.g. secretary, teacher, builder etc.)?**

---

13 **And in what industry do you work (e.g. farming, shipyard, car factory, shoe shop, hospital, insurance office etc)?**

---

14 **Which of the following best describes your present work situation for your MAIN occupation?** Please tick one box.

a) Employed (permanent contract) ☐
b) Employed (temporary/renewable contract) ☐
c) Zero hours contract ☐
d) Self-employed ☐
e) Other *(please specify)* ☐

---

15 **On average, how many *hours* per week do you normally work in your main occupation?**

☐☐☐ hours

16 **On average, how many *days* per week do you normally work in your main occupation?**

☐ days

17 **Do you have any other paid work?**
If yes, please answer Question 17.1; if no, please move on to Question 18.

Yes ☐ No ☐

17.1 **If yes, on average, how many hours a week do you work in other paid jobs?** ☐☐ hours

18 **Does an average day at work in your MAIN job normally involve any of the following?** Please tick one box for each question.

		Yes	No
a)	Piecework in which you are paid according to the number of articles or tasks you or your team make or finish in the day?	☐	☐
b)	A target number of articles or tasks that you or your team are expected to make or finish in the day?	☐	☐
c)	Payment of a bonus if you make or finish more than an agreed number of articles/tasks in the day?	☐	☐
d)	Working to tight deadlines	☐	☐
e)	Use of a computer keyboard or mouse for longer than 1 hour in total?	☐	☐

For peer review only - http://bmjopen.bmj.com/site/about/guidelines.xhtml

SECTION B: WORK

		Yes	No
f)	Use of a computer keyboard or mouse for longer than 4 hours in total?	☐	☐
g)	Other tasks involving repeated movements of the wrist or fingers for longer than 4 hours in total? *(Please indicate which tasks)*	☐	☐
h)	Working with a powered tool that makes your hand(s) or arm(s) vibrate (e.g. chain saw, pneumatic drill)?	☐	☐
i)	Working with your hand(s) above shoulder height for longer than 1 hour in total?	☐	☐
j)	Lifting or carrying weights of 5 kg (11 lbs) or more in one hand (e.g. a tool bag or heavy briefcase)?	☐	☐
k)	Lifting or carrying a weight of 10 kg (22 lbs) or more?	☐	☐
l)	Tasks involving pushing or pulling a heavy weight?	☐	☐
m)	Working for longer than two hours in total with your neck bent forward?	☐	☐
n)	Working for longer than half an hour in total with your neck twisted e.g. when looking to one side?	☐	☐
o)	Driving for more than an hour?	☐	☐

19 Do you find your MAIN job demanding on your hands/wrists?
Please circle one number, where 0 represents not at all, and 10 represents very much.

0 1 2 3 4 5 6 7 8 9 10

Not at all — Very much

20 Does your MAIN employer (or boss/colleagues if self-employed) know about your hand/wrist problem?
If yes, please answer Question 20.1; if no, or not applicable, please move on to Question 21.

Yes ☐ No ☐ N/A self-employed and work alone ☐

20.1 Is your MAIN employer (or boss/colleagues if self-employed) supportive of your hand/wrist problem?
Please circle one number, where 0 represents not at all, and 10 represents very much

0 1 2 3 4 5 6 7 8 9 10

Not at all — Very much

For peer review only - http://bmjopen.bmj.com/site/about/guidelines.xhtml

SECTION B: WORK

21 **The following questions refer to how you did in your MAIN job during the *past 4 weeks*.**
Please tick one box for each question.

How much of the time during the past 4 weeks …	**Always**	**Often**	**Sometimes**	**Rarely**	**Never**
a) Were you unable to do your work because of problems with your hand(s) / wrist(s)?	☐	☐	☐	☐	☐
b) Did you have to shorten your work day because of problems with your hand(s) / wrists(s)	☐	☐	☐	☐	☐
c) Did you have to take breaks at work because of problems with your hand(s) / wrists(s)?	☐	☐	☐	☐	☐
d) Did you get less done because of problems with your hand(s) / wrist(s)?	☐	☐	☐	☐	☐
e) Did you take longer to do the tasks in your work because of problems with your hand(s) / wrists(s)?	☐	☐	☐	☐	☐

22 **During the *past 4 weeks*, how much time have you missed from your MAIN job for the following reasons?**
Please write 0 if you have not missed any time from work during this period. You can answer in days or hours, whichever applies.

a) Time missed because of the problem with your hand(s)/wrist(s) ☐☐ Days ***or*** ☐☐ Hours

b) Time missed because of any other problem ☐☐ Days ***or*** ☐☐ Hours

23 **If you fell ill and were off work, how long could you get your normal full pay (excluding bonuses)?**
Please tick the option that best represents your MAIN job.

a) Less than one week ☐
b) 1 – 4 weeks ☐
c) 1 – 6 months ☐
d) More than 6 months ☐
e) Not sure ☐

24 **How satisfied are you with your MAIN job as a whole, taking everything into consideration?** This includes your salary, career possibilities, management, colleagues etc. Please tick one box.

a) Very satisfied ☐
b) Satisfied/fairly satisfied ☐
c) Dissatisfied ☐
d) Very dissatisfied ☐

For peer review only - http://bmjopen.bmj.com/site/about/guidelines.xhtml

SECTION C: GENERAL HEALTH

25 In general, would you say your health is:

a) Excellent ☐ d) Fair ☐

b) Very good ☐ e) Poor ☐

c) Good ☐

26 What is your height? Please answer in either feet and inches or centimetres.

☐ feet ☐☐ inches ***or*** ☐☐☐ cms

27 What is your weight? Please answer in either stones and pounds or kilograms.

☐☐ stones ☐☐ lbs ***or*** ☐☐ kgs

28 Do you, or have you ever, smoked regularly? Please tick one box.

a) I have never smoked regularly ☐ c) I regularly smoke ☐

b) I have smoked in the past, but do not currently smoke regularly ☐

29 The following is a list of common health problems. Please indicate if you currently have, or don't have, the problem listed in part 1. If you have the problem, please answer the corresponding question in part 2.
Please answer all questions in part 1.

	**HEALTH PROBLEM**	**PART 1 Do you have the problem?** **NO**	**YES** *(if yes move to part 2)* →	**PART 2 Does it limit your activities?** **NO**	**YES**
a)	Heart disease	☐	☐	☐	☐
b)	High blood pressure	☐	☐	☐	☐
c)	Lung disease	☐	☐	☐	☐
d)	Diabetes	☐	☐	☐	☐
e)	Ulcer or stomach disease	☐	☐	☐	☐
f)	Kidney disease	☐	☐	☐	☐
g)	Liver disease	☐	☐	☐	☐
h)	Thyroid disease	☐	☐	☐	☐
i)	Anaemia or other blood disease	☐	☐	☐	☐
j)	Cancer	☐	☐	☐	☐

For peer review only - http://bmjopen.bmj.com/site/about/guidelines.xhtml

SECTION C: GENERAL HEALTH

HEALTH PROBLEM continued…	Do you have the problem? NO	YES (if yes move to part 2) →	Does it limit your activities? NO	YES
k) Depression	☐	☐	☐	☐
l) Osteoarthritis	☐	☐	☐	☐
m) Back pain	☐	☐	☐	☐
n) Rheumatoid arthritis	☐	☐	☐	☐

29.1 Please list any other medical problems that have not been mentioned.

	Does it limit your activities? NO	YES
o)	☐	☐
p)	☐	☐
q)	☐	☐

30 The following questions are about how you feel and how things have been with you during the *past 4 weeks*. For each question, please give the answer that comes closest to the way you have been feeling. Please tick one box for each row.

How much of the time during the past 4 weeks …	All of the time	Most of the time	A good bit of the time	Some of the time	A little bit of the time	None of the time
a) Did you feel full of 'get-up-and-go'?	☐	☐	☐	☐	☐	☐
b) Have you been a very nervous person?	☐	☐	☐	☐	☐	☐
c) Have you felt so down in the dumps that nothing could cheer you up?	☐	☐	☐	☐	☐	☐
d) Have you felt calm and peaceful?	☐	☐	☐	☐	☐	☐
e) Did you have a lot of energy?	☐	☐	☐	☐	☐	☐
f) Have you felt downhearted and blue?	☐	☐	☐	☐	☐	☐
g) Did you feel worn out?	☐	☐	☐	☐	☐	☐
h) Have you been a happy person?	☐	☐	☐	☐	☐	☐
i) Did you feel tired?	☐	☐	☐	☐	☐	☐

For peer review only - http://bmjopen.bmj.com/site/about/guidelines.xhtml

SECTION C: GENERAL HEALTH

31 **Below is a list of problems that people sometimes have. Please read each one carefully and tick the box that best describes how much that problem has distressed or bothered you during the *past 7 days*, including today?**
Please tick one box for each row.

		Not at all	A little bit	Moderately	Quite a bit	Extremely
a)	Faintness or dizziness	☐	☐	☐	☐	☐
b)	Pains in the heart or chest	☐	☐	☐	☐	☐
c)	Nausea or upset stomach	☐	☐	☐	☐	☐
d)	Trouble getting your breath	☐	☐	☐	☐	☐
e)	Hot or cold spells	☐	☐	☐	☐	☐

For peer review only

For peer review only - http://bmjopen.bmj.com/site/about/guidelines.xhtml

SECTION D: HAND AND WRIST FUNCTION

32 **In the *past 7 days*, have you experienced any pain, tingling (pins and needles) or numbness (loss of sensation) in your RIGHT hand or wrist?**
Please mark where on your hand/wrist you experienced these symptoms using the key below.
If you do not have any symptoms in your right hand, please move on to Question 34.

Pain | Tingling or numbness

RIGHT HAND

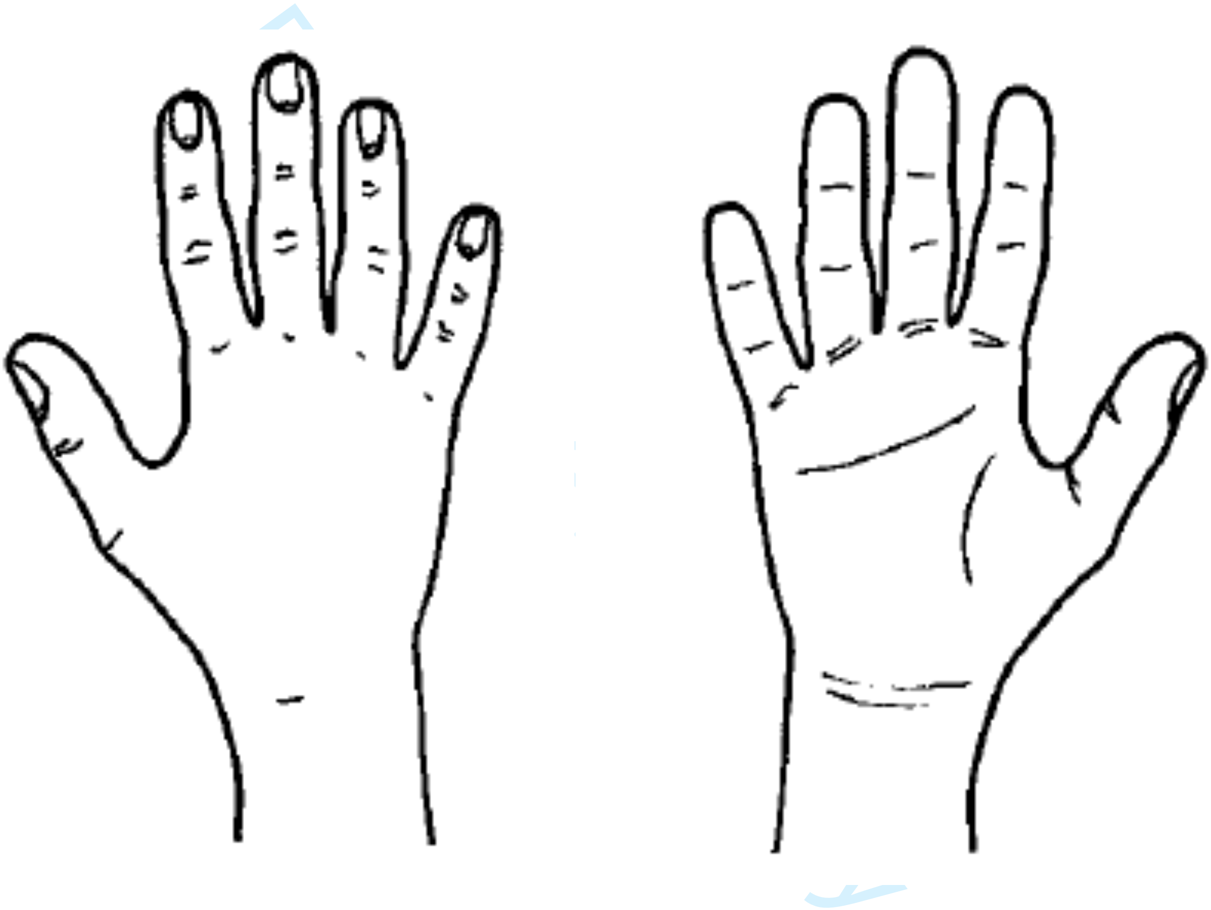

33 How long ago did the first of these symptoms begin? Please tick one box.

a) Less than 3 months ☐
b) 3 – 6 months ☐
c) 6 – 12 months ☐
d) More than a year ☐

For peer review only - http://bmjopen.bmj.com/site/about/guidelines.xhtml

SECTION D: HAND AND WRIST FUNCTION

34 **In the *past 7 days*, have you experienced any pain, tingling (pins and needles) or numbness (loss of sensation) in your LEFT hand or wrist?**
Please mark where on your hand/wrist you experienced these symptoms using the key below.
If you do not have any symptoms in your right hand, please move on to Question 36.

Pain ☐ Tingling or numbness ☐

LEFT HAND

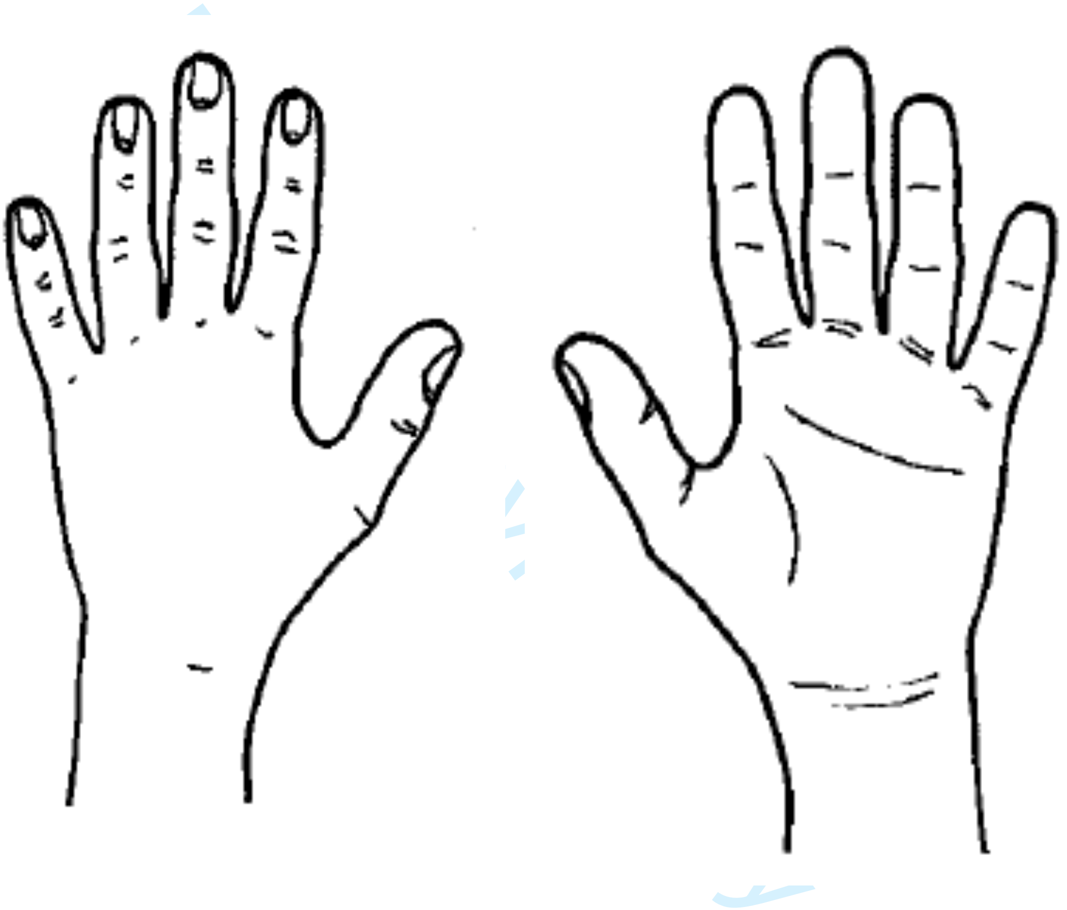

35 How long ago did the first of these symptoms begin? Please tick one box.

a) Less than 3 months ☐

b) 3 – 6 months ☐

c) 6 – 12 months ☐

d) More than a year ☐

For peer review only - http://bmjopen.bmj.com/site/about/guidelines.xhtml

SECTION D: HAND AND WRIST FUNCTION

36 **The following questions refer to your symptoms over the *last 7 days*.**
Please answer for each hand, even if you only have problems with one side.
Please tick one box for each row.

**36.1 How severe were the following symptoms in your RIGHT hand?**	**None**	**Mild**	**Moderate**	**Severe**	**Very severe**
a) Pain at night	☐	☐	☐	☐	☐
b) Pain during the daytime	☐	☐	☐	☐	☐
c) Numbness or tingling at night	☐	☐	☐	☐	☐
d) Numbness or tingling during the daytime	☐	☐	☐	☐	☐

**How often did the following symptoms in your RIGHT hand wake you up at night?**	**Never**	**Once**	**2 or 3 times**	**4 or 5 times**	**More than 5 times**
e) Pain	☐	☐	☐	☐	☐
f) Numbness or tingling	☐	☐	☐	☐	☐

**36.2 How severe were the following symptoms in your LEFT hand?**	**None**	**Mild**	**Moderate**	**Severe**	**Very severe**
a) Pain at night	☐	☐	☐	☐	☐
b) Pain during the daytime	☐	☐	☐	☐	☐
c) Numbness or tingling at night	☐	☐	☐	☐	☐
d) Numbness or tingling during the daytime	☐	☐	☐	☐	☐

**How often did the following symptoms in your LEFT hand wake you up at night?**	**Never**	**Once**	**2 or 3 times**	**4 or 5 times**	**More than 5 times**
e) Pain	☐	☐	☐	☐	☐
f) Numbness or tingling	☐	☐	☐	☐	☐

37 **This question refers to the appearance (look) of your hand during the *past 7 days*.** Please tick one box for each hand.

	**Strongly agree**	**Agree**	**Neither agree nor disagree**	**Disagree**	**Strongly disagree**
a) I am satisfied with the appearance (look) of my **RIGHT** hand	☐	☐	☐	☐	☐
b) I am satisfied with the appearance (look) of my **LEFT** hand	☐	☐	☐	☐	☐

For peer review only

For peer review only - http://bmjopen.bmj.com/site/about/guidelines.xhtml

SECTION D: HAND AND WRIST FUNCTION

38 Please answer the following questions on a scale of 0-10, where 0 represents not at all, and 10 represents very much. Please circle one number for each question.

38.1 Do you think that you will be able to use your hand normally 3 months after the operation?

0 1 2 3 4 5 6 7 8 9 10

38.2 Are you afraid of having long-term problems with your hand?

0 1 2 3 4 5 6 7 8 9 10

38.3 Do you blame yourself for your hand problem?

0 1 2 3 4 5 6 7 8 9 10

38.4 Are your family and friends supportive of your hand problem?

0 1 2 3 4 5 6 7 8 9 10

39 The following statements describe people's beliefs about their health problems. Please indicate whether you agree or disagree with them in relation to the problems you have with your hand(s) or wrist(s).
Please tick the box which most closely reflects how you feel for each statement.

	Strongly agree	Agree	Neither agree or disagree	Disagree	Strongly Disagree
a) Problems like this run in my family	☐	☐	☐	☐	☐
b) I think I was born with a weakness or underlying problem in this part of my body	☐	☐	☐	☐	☐
c) My problem was caused by work	☐	☐	☐	☐	☐
d) Work probably didn't cause my problem, but it made it worse	☐	☐	☐	☐	☐
e) I have a lot of stress in my life and that has made my problem a lot worse	☐	☐	☐	☐	☐
f) I think a lack of exercise probably contributed to my problem	☐	☐	☐	☐	☐
g) As you get older, parts of the body start to wear out and problems like mine are likely to occur	☐	☐	☐	☐	☐

For peer review only - http://bmjopen.bmj.com/site/about/guidelines.xhtml

SECTION D: HAND AND WRIST FUNCTION

40 We are interested in the types of thoughts and feelings that you have when you are in pain. The following statements describe different thoughts and feelings that may be associated with pain. Please indicate the degree to which you have these thoughts and feelings when you are experiencing pain.
Please tick one box for each statement.

	Not at all	To a slight degree	To a moderate degree	To a great degree	All of the time
a) I keep thinking about how badly I want the pain to stop	☐	☐	☐	☐	☐
b) It's terrible and I think it's never going to get any better	☐	☐	☐	☐	☐
c) I become afraid that the pain may get worse	☐	☐	☐	☐	☐
d) I anxiously want the pain to go away	☐	☐	☐	☐	☐

The following questions refer to the function of your hands/wrists during the ***past 7 days***. Please answer all questions for the right and left sides, even if you do not experience any problems. Please tick one box for each question.

**41 RIGHT SIDE**	Very well	Well	Adequately	Poorly	Very poorly
a) Overall, how well did your ***right*** hand work?	☐	☐	☐	☐	☐
b) How well did your ***right*** fingers move?	☐	☐	☐	☐	☐
c) How well did your ***right*** wrist move?	☐	☐	☐	☐	☐
	**Very good**	**Good**	**Fair**	**Poor**	**Very poor**
d) How was the strength in your ***right*** hand?	☐	☐	☐	☐	☐
e) How was the sensation (feeling) in your ***right*** hand?	☐	☐	☐	☐	☐

**42 LEFT SIDE**	Very well	Well	Adequately	Poorly	Very poorly
a) Overall, how well did your ***left*** hand work?	☐	☐	☐	☐	☐
b) How well did your ***left*** fingers move?	☐	☐	☐	☐	☐
c) How well did your ***left*** wrist move?	☐	☐	☐	☐	☐
	**Very good**	**Good**	**Fair**	**Poor**	**Very poor**
d) How was the strength in your ***left*** hand?	☐	☐	☐	☐	☐
e) How was the sensation (feeling) in your ***left*** hand?	☐	☐	☐	☐	☐

For peer review only

SECTION D: HAND AND WRIST FUNCTION

The following questions refer to the ability of your hands to do certain tasks during the ***past 7 days***. If you do not do a certain task, please estimate the difficulty you would have in performing it. Please tick one box for every activity.

43 How difficult was it for you to perform the following activities using your RIGHT HAND?

		Not at all difficult	A little difficult	Somewhat difficult	Moderately difficult	Very difficult
a)	Turn a door knob	☐	☐	☐	☐	☐
b)	Pick up a coin	☐	☐	☐	☐	☐
c)	Hold a glass of water	☐	☐	☐	☐	☐
d)	Turn a key in a lock	☐	☐	☐	☐	☐
e)	Hold a frying pan	☐	☐	☐	☐	☐

44 How difficult was it for you to perform the following activities using your LEFT HAND?

		Not at all difficult	A little difficult	Somewhat difficult	Moderately difficult	Very difficult
a)	Turn a door knob	☐	☐	☐	☐	☐
b)	Pick up a coin	☐	☐	☐	☐	☐
c)	Hold a glass of water	☐	☐	☐	☐	☐
d)	Turn a key in a lock	☐	☐	☐	☐	☐
e)	Hold a frying pan	☐	☐	☐	☐	☐

45 How difficult was it for you to perform the following activities using BOTH HANDS?

		Not at all difficult	A little difficult	Somewhat difficult	Moderately difficult	Very difficult
a)	Open a jar	☐	☐	☐	☐	☐
b)	Button a shirt/blouse	☐	☐	☐	☐	☐
c)	Eat with a knife/fork	☐	☐	☐	☐	☐
d)	Carry a grocery bag	☐	☐	☐	☐	☐
e)	Wash dishes	☐	☐	☐	☐	☐
f)	Wash your hair	☐	☐	☐	☐	☐
g)	Tie shoelaces/knots	☐	☐	☐	☐	☐

For peer review only - http://bmjopen.bmj.com/site/about/guidelines.xhtml

SECTION D: HAND AND WRIST FUNCTION

The following questions refer to your satisfaction with your hands/wrists during the ***past 7 days***. Please tick one box for each question

46 How satisfied were you with your RIGHT hand/wrist during the *past 7 days*?

	RIGHT HAND	Very satisfied	Somewhat satisfied	Neither satisfied or dissatisfied	Somewhat dissatisfied	Very dissatisfied
a)	Overall function of your hand					
b)	Movement of the fingers					
c)	Movement of your wrist					
d)	Strength of your hand					
e)	Pain level of your hand					
f)	Sensation (feeling) of your hand					

47 How satisfied were you with your LEFT hand/wrist during the *past 7 days*?

	LEFT HAND	Very satisfied	Somewhat satisfied	Neither satisfied or dissatisfied	Somewhat dissatisfied	Very dissatisfied
a)	Overall function of your hand					
b)	Movement of the fingers					
c)	Movement of your wrist					
d)	Strength of your hand					
e)	Pain level of your hand					
f)	Sensation (feeling) of your hand					

Thank you for completing this questionnaire!
Please return it to the REACTS team
using the pre-paid envelope.

If you have any questions, or would like any additional information, please contact Lisa Newington on:
ln@mrc.soton.ac.uk | 023 8077 7624 | 07866 997732

For peer review only

For peer review only - http://bmjopen.bmj.com/site/about/guidelines.xhtml

University Hospital Southampton NHS Foundation Trust

REACTS

Arthritis Research UK MRC centre for musculoskeletal health & work

UNIVERSITY OF Southampton

REACTS ID: [ ][ ] [ ][ ][ ]

One Month After Your Carpal Tunnel Release Surgery

REACTS

Return to Employment After Carpal Tunnel Release Surgery (REACTS)

ln@mrc.soton.ac.uk | 023 8077 7624
Arthritis Research UK – MRC Centre for Musculoskeletal Health and Work
MRC Lifecourse Epidemiology Unit, University of Southampton
Southampton General Hosptial (MP 95), SO16 6YD

IRAS reference: 209840

SECTION A: ABOUT YOUR OPERATION

Please fill in today's date ☐☐☐☐☐☐☐☐
d d m m y y y y

1 What was the date of your carpal tunnel release surgery? ☐☐☐☐☐☐☐☐
d d m m y y y y

2 Which side was operated on? Please tick one box.

Right ☐ Left ☐ Both ☐

3 What type of anaesthetic did you have? Please tick one box.

a) General anaesthetic (you were sent to sleep) ☐

b) Local or regional anaesthetic (your arm was made numb, but you were still awake) ☐

c) Other *(please specify)* ☐

----

d) Unsure ☐

4 How long did you need to stay in the hospital/clinic after your operation?
Please tick one box (and specify the number of nights, if applicable).

a) I went home the same day ☐

b) I needed to stay overnight (one night only) ☐

c) I needed to stay for more than one night ☐

(Please specify for how long) ☐☐ nights

For peer review only

SECTION A: ABOUT YOUR OPERATION

5 Have you used any of the following services specifically for your operated hand(s) since your surgery?
Please give the number of visits for each service, and the date(s) attended, if known.

	I used this service in the NHS		I used this service privately	
	Number of visits	Dates attended, if known	Number of visits	Dates attended, if known
a) Your surgeon, or one of the surgical team				
b) GP or practice nurse				
c) Hospital nurse				
d) Pharmacist				
e) Hand therapist				
f) Other physiotherapist or occupational therapist				
g) Chiropractor or osteopath				
h) Occupational health nurse or doctor				
i) Accident and emergency (A&E) or minor injuries unit				
j) Other (*please specify*)				

For peer review only - http://bmjopen.bmj.com/site/about/guidelines.xhtml

SECTION A: ABOUT YOUR OPERATION

6 Have you taken any antibiotics for an infection in your surgical wound?
Please do not include any antibiotics you were prescribed at the time of your operation.

Yes ☐ No ☐

If yes, what date did you start taking the antibiotics? ☐☐☐☐☐☐☐☐ d d m m y y y y

7 Have you been admitted to hospital because of a problem with your operated hand(s)? If yes, please answer the rest of Question 7; if no, please move on to Question 8.

Yes ☐ No ☐

7.1 If yes, when were you first admitted? ☐☐☐☐☐☐☐☐ d d m m y y y y

7.2 How many nights did you stay in hospital?
Please answer 0 if you didn't stay overnight.
☐☐ nights

7.3 Did you require another operation? Yes ☐ No ☐

8 Have you been advised that you may need a carpal tunnel release for your other hand in the future?
If yes, please answer Question 8.1; if no, please move on to Question 9.

Yes ☐ No ☐

8.1 If yes, when are you expecting to have this surgery? Please tick one box.

a) In less than 2 months ☐
b) In 2-5 months ☐
c) In 6-11 months ☐
d) In more than a year ☐
e) Unsure ☐

9 If you would like to give us any other information about your operation, or the healthcare services you have used, please do so here:

For peer review only - http://bmjopen.bmj.com/site/about/guidelines.xhtml

SECTION B: WORK

10 Compared to before your surgery, which of the following best describes your current work situation? Please tick one box.

a) Returned to the same job, work duties and hours – **please go to Question 14** ☐

b) Returned to the same job, with altered duties or hours – **please go to Question 14** ☐

c) Started a new job – **please go to Question 11** ☐

d) Not yet returned to work, but plan to return in the future – **please go to Question 12** ☐

e) Do not plan to return to work – **please go to Question 13** ☐

11 Thinking about your new job:

11.1 What is your main occupation now (e.g. secretary, teacher, builder etc.)?

11.2 In what industry do you work (e.g. farming, shipyard, car factory, shoe shop, hospital, insurance office etc.)?

11.3 Did you change jobs because of your hand/wrist problem? Please tick one box.

a) Yes, my hand/wrist problem was the main reason for my job change ☐

b) Yes, my hand/wrist problem was one of several reasons for my job change ☐

c) No, my job change was nothing to do with my hand/wrist problem ☐

d) Other, please specify ☐

Please go to Question 14

For peer review only

SECTION B: WORK

12 If you have not yet returned to work, when do you think you might be able to return? Please give an estimated date if you are unsure.

d	d	m	m	y	y	y	y

12.1 Have you discussed when to return to work with anyone?
If yes, please answer the rest of Question 12; if no, please move on to Question 21.

Yes ☐ No ☐

12.2 If yes, who have you discussed this with? Please tick all that apply.

a) Your surgeon or a member of the surgical team ☐
b) Hospital nurse ☐
c) GP or practice nurse ☐
d) Hand therapist ☐
e) Physiotherapist or occupational therapist ☐
f) Occupational health nurse or doctor ☐
g) Employer or manager (or colleagues if self-employed ☐
h) Friend or family member ☐
i) Other *(please specify)* ☐

12.3 Since your operation, have you been given any specific advice about when and how to return to work? This could include any activities to avoid or timescales to follow. Please list any advice here, including who gave you this advice:

Please go to Question 21

For peer review only

SECTION B: WORK

13 If you do not plan to return to work, what is the main reason for this decision?
Please tick one box.

a) Retirement ☐

b) Redundancy ☐

c) Position/work no longer available ☐

d) Unable to do your work because of your problem with your hand(s)/wrist(s) ☐

e) Unable to do your work because of any other problem ☐

f) Other *(please specify)* ☐

13.1 Have you been advised not to return to work by anyone?
If yes, please answer Question 13.2; if no, please move on to Question 21.

Yes ☐ No ☐

13.2 If yes, who by? Please tick all that apply.

a) Your surgeon or a member of the surgical team ☐

b) Hospital nurse ☐

c) GP or practice nurse ☐

d) Hand therapist ☐

e) Physiotherapist or occupational therapist ☐

f) Occupational health nurse or doctor ☐

g) Employer or manager (or colleagues if self-employed) ☐

h) Friend or family member ☐

i) Other *(please specify)* ☐

Please go to Question 21

For peer review only - http://bmjopen.bmj.com/site/about/guidelines.xhtml

For peer review only

SECTION B: WORK

14 When did you first return to work after your carpal tunnel release surgery?

☐☐☐☐☐☐☐☐
d d m m y y y y

15 How much work-time did you miss between the date of your surgery and the date you first returned to work?
Please include all work-time missed, even if this had been pre-arranged with your employer, or was taken as annual leave. You can answer in hours, days or weeks, whichever applies.

☐☐☐ hours ☐☐ days ☐☐ weeks

15.1 Was any of this time paid?
Please tick one box (and provide the amount of time, if applicable).

a) Yes, all of my time away from work was paid ☐

b) Yes, some of my time away from work was paid ☐
(please specify how much time was paid, you can use hours, days or weeks, whichever applies)

☐☐☐ hours ☐☐ days ☐☐ weeks

c) No, none of my time off was paid ☐

d) Not sure ☐

16 Since your surgery, have you discussed when to return to work with anyone?
If yes, please answer the rest of Question 16; if no, please move on to Question 17.

Yes ☐ No ☐

16.1 If yes, who did you discuss this with? Please tick all that apply.

a) Your surgeon or a member of the surgical team ☐

b) Hospital nurse ☐

c) GP or practice nurse ☐

d) Hand therapist ☐

e) Physiotherapist or occupational therapist ☐

f) Occupational health nurse or doctor ☐

g) Employer or manager (or colleagues if self-employed ☐

h) Friend or family member ☐

i) Other *(please specify)* ☐

....

....

For peer review only - http://bmjopen.bmj.com/site/about/guidelines.xhtml

SECTION B: WORK

16.2 Please list any advice you have been given (since your surgery) about when and how to return to work?
This could include any activities to avoid or timescales to follow. If this advice came from more than one place, please indicate who advised what.

----

17 Since returning to work after your operation, have you needed to take any time off work because of a problem with your operated hand(s)/wrist(s)?
If yes, please answer Question 17.1; if no, please move on to Question 18.

Yes ☐ No ☐

17.1 If yes, how much time did you take off work?
Please answer in days or hours, whichever applies.

☐☐☐ hours ☐☐ days ☐☐ weeks

18 When you first returned to work after your surgery, did you work shorter hours than would be normal for your job as a direct result of your operation?
If yes, please answer the rest of Question 18; if no, please move on to Question 19.

Yes ☐ No ☐

18.1 Have you since gone back to working full hours?
If yes, please answer Question 18.2; if no, please move on to Question 19.

Yes ☐ No ☐

18.2 If yes, when did you return to full working hours?
If you do not know the exact date, approximately how many weeks did you work reduced hours?

☐☐☐☐☐☐☐☐
d d m m y y y y

a) Less than a week ☐
b) 1 – 2 weeks ☐
c) More than 2 weeks, but less than 3 weeks ☐
d) 3 weeks or longer ☐

For peer review only - http://bmjopen.bmj.com/site/about/guidelines.xhtml

SECTION B: WORK

19 When you first returned to work after your surgery, did you need to alter or avoid any of your usual work duties as a direct result of your operation?
If yes, please answer the rest of Question 19; if no, please move on to Question 20.

Yes ☐ No ☐

19.1 Have you since gone back to full duties?
If yes, please answer Question 19.2; if no, please move on to Question 20.

Yes ☐ No ☐

19.2 If yes, when did you return to full working duties?
If you do not know the exact date, approximately how many weeks did you have altered work duties?

d	d	m	m	y	y	y	y

a) Less than a week ☐

b) 1 – 2 weeks ☐

c) More than 2 weeks, but less than 3 weeks ☐

d) 3 weeks or longer ☐

20 If you would like to give us any additional information about returning to work, please do so here:

For peer review only - http://bmjopen.bmj.com/site/about/guidelines.xhtml

SECTION C: HAND AND WRIST SYMPTOMS

21 **The following questions refer to your symptoms over the *last 7 days*.**
Please answer for each hand. Please tick one box for each row.

**21.1 How severe were the following symptoms in your RIGHT hand?**	**None**	**Mild**	**Moderate**	**Severe**	**Very severe**
a) Pain at night	☐	☐	☐	☐	☐
b) Pain during the daytime	☐	☐	☐	☐	☐
c) Numbness or tingling at night	☐	☐	☐	☐	☐
d) Numbness or tingling during the daytime	☐	☐	☐	☐	☐

**How often did the following symptoms in your RIGHT hand wake you up at night?**	**Never**	**Once**	**2 or 3 times**	**4 or 5 times**	**More than 5 times**
e) Pain	☐	☐	☐	☐	☐
f) Numbness or tingling	☐	☐	☐	☐	☐

**21.2 How severe were the following symptoms in your LEFT hand?**	**None**	**Mild**	**Moderate**	**Severe**	**Very severe**
a) Pain at night	☐	☐	☐	☐	☐
b) Pain during the daytime	☐	☐	☐	☐	☐
c) Numbness or tingling at night	☐	☐	☐	☐	☐
d) Numbness or tingling during the daytime	☐	☐	☐	☐	☐

**How often did the following symptoms in your LEFT hand wake you up at night?**	**Never**	**Once**	**2 or 3 times**	**4 or 5 times**	**More than 5 times**
e) Pain	☐	☐	☐	☐	☐
f) Numbness or tingling	☐	☐	☐	☐	☐

22 **This question refers to the appearance (look) of your hands during the *past 7 days*.** Please tick one box for each hand.

	**Strongly agree**	**Agree**	**Neither agree nor disagree**	**Disagree**	**Strongly disagree**
a) I am satisfied with the appearance (look) of my **RIGHT** hand	☐	☐	☐	☐	☐
b) I am satisfied with the appearance (look) of my **LEFT** hand	☐	☐	☐	☐	☐

For peer review only - http://bmjopen.bmj.com/site/about/guidelines.xhtml

SECTION C: HAND AND WRIST SYMPTOMS

23 How do you rate your symptoms in your operated hand(s) now, compared to before your surgery? Please tick one box.

a) Completely cured ☐
b) Much better ☐
c) Unchanged ☐
d) Slightly better ☐
e) Worse ☐

24 The following questions ask specifically about your scar. Please think about your scar over the ***past 7 days.***

24.1 Has your scar been itchy?
If yes, please continue; if no, please move on to Question 24.2

Yes ☐ No ☐

Yes, it was itchy:	Sometimes ☐	Often ☐	Always ☐
And when it was itchy, it was:	Slightly itchy ☐	Fairly itchy ☐	Very itchy ☐

24.2 Has your scar caused you pain?
If yes, please continue; if no, please move on to Question 24.3

Yes ☐ No ☐

Yes, it was painful:	Sometimes ☐	Often ☐	Always ☐
And when it hurt, it was:	Slightly painful ☐	Fairly painful ☐	Very painful ☐

24.3 Has your scar been uncomfortable?
If yes, please continue; if no, please move on to Question 24.4

Yes ☐ No ☐

Yes, it was uncomfortable:	Sometimes ☐	Often ☐	Always ☐
And when it was uncomfortable, it was:	Slightly uncomfortable ☐	Fairly uncomfortable ☐	Very uncomfortable ☐

For peer review only - http://bmjopen.bmj.com/site/about/guidelines.xhtml

SECTION C: HAND AND WRIST SYMPTOMS

24.4 Has your scar felt numb?
If yes, please continue; if no, please move on to Question 24.5

Yes ☐ No ☐

Yes, it was numb:	Sometimes ☐	Often ☐	Always ☐
And when it felt numb, it was:	Slightly numb ☐	Fairly numb ☐	Very numb ☐

24.5 Have you had odd sensations in your scar e.g. tightening, pulling or pins and needles? If yes, please continue; if no, please move on to Question 24.6

Yes ☐ No ☐

Yes, I have had odd sensations:	Sometimes ☐	Often ☐	Always ☐

24.6 Has your scar caught on things e.g. clothing?
If yes, please continue; if no, please move on to Question 24.7

Yes ☐ No ☐

Yes, it has caught on things:	Sometimes ☐	Often ☐	Always ☐

24.7 Overall, how troublesome are the symptoms from your scar?
Please tick one box.

Not at all troublesome	A little troublesome	Fairly troublesome	Very troublesome	Unbearable
☐	☐	☐	☐	☐

For peer review only - http://bmjopen.bmj.com/site/about/guidelines.xhtml

SECTION D: HAND AND WRIST FUNCTION

The following questions refer to the function of your hands/wrists during the ***past 7 days***. Please answer all questions for the right and left sides, even if you do not experience any problems. Please tick one box for each question.

25 RIGHT SIDE	Very well	Well	Adequately	Poorly	Very poorly
a) Overall, how well did your ***right*** hand work?	☐	☐	☐	☐	☐
b) How well did your ***right*** fingers move?	☐	☐	☐	☐	☐
c) How well did your ***right*** wrist move?	☐	☐	☐	☐	☐
	**Very good**	**Good**	**Fair**	**Poor**	**Very poor**
d) How was the strength in your ***right*** hand?	☐	☐	☐	☐	☐
e) How was the sensation (feeling) in your ***right*** hand?	☐	☐	☐	☐	☐

26 LEFT SIDE	Very well	Well	Adequately	Poorly	Very poorly
a) Overall, how well did your ***left*** hand work?	☐	☐	☐	☐	☐
b) How well did your ***left*** fingers move?	☐	☐	☐	☐	☐
c) How well did your ***left*** wrist move?	☐	☐	☐	☐	☐
	**Very good**	**Good**	**Fair**	**Poor**	**Very poor**
d) How was the strength in your ***left*** hand?	☐	☐	☐	☐	☐
e) How was the sensation (feeling) in your ***left*** hand?	☐	☐	☐	☐	☐

The following questions refer to the ability of your hands to do certain tasks during the ***past 7 days***. If you do not do a certain task, please estimate the difficulty you would have in performing it. Please tick one box for every activity.

27 How difficult was it for you to perform the following activities using your RIGHT HAND?

	Not at all difficult	A little difficult	Somewhat difficult	Moderately difficult	Very difficult
a) Turn a door knob	☐	☐	☐	☐	☐
b) Pick up a coin	☐	☐	☐	☐	☐
c) Hold a glass of water	☐	☐	☐	☐	☐
d) Turn a key in a lock	☐	☐	☐	☐	☐
e) Hold a frying pan	☐	☐	☐	☐	☐

For peer review only - http://bmjopen.bmj.com/site/about/guidelines.xhtml

SECTION D: HAND AND WRIST FUNCTION

28 How difficult was it for you to perform the following activities using your LEFT HAND?

		Not at all difficult	A little difficult	Somewhat difficult	Moderately difficult	Very difficult
a)	Turn a door knob	☐	☐	☐	☐	☐
b)	Pick up a coin	☐	☐	☐	☐	☐
c)	Hold a glass of water	☐	☐	☐	☐	☐
d)	Turn a key in a lock	☐	☐	☐	☐	☐
e)	Hold a frying pan	☐	☐	☐	☐	☐

29 How difficult was it for you to perform the following activities using BOTH HANDS?

		Not at all difficult	A little difficult	Somewhat difficult	Moderately difficult	Very difficult
a)	Open a jar	☐	☐	☐	☐	☐
b)	Button a shirt/blouse	☐	☐	☐	☐	☐
c)	Eat with a knife/fork	☐	☐	☐	☐	☐
d)	Carry a grocery bag	☐	☐	☐	☐	☐
e)	Wash dishes	☐	☐	☐	☐	☐
f)	Wash your hair	☐	☐	☐	☐	☐
g)	Tie shoelaces/knots	☐	☐	☐	☐	☐

The following questions refer to your satisfaction with your hands/wrists during the ***past 7 days***. Please tick one box for each question.

30 How satisfied were you with your RIGHT hand/wrist during the *past 7 days*?

	RIGHT HAND	Very satisfied	Somewhat satisfied	Neither satisfied or dissatisfied	Somewhat dissatisfied	Very dissatisfied
a)	Overall function of your hand	☐	☐	☐	☐	☐
b)	Movement of the fingers	☐	☐	☐	☐	☐
c)	Movement of your wrist	☐	☐	☐	☐	☐
d)	Strength of your hand	☐	☐	☐	☐	☐
e)	Pain level of your hand	☐	☐	☐	☐	☐
f)	Sensation (feeling) of your hand	☐	☐	☐	☐	☐

For peer review only - http://bmjopen.bmj.com/site/about/guidelines.xhtml

SECTION D: HAND AND WRIST FUNCTION

31 How satisfied were you with your LEFT hand/wrist during the *past 7 days*?

	LEFT HAND	Very satisfied	Somewhat satisfied	Neither satisfied or dissatisfied	Somewhat dissatisfied	Very dissatisfied
a)	Overall function of your hand	☐	☐	☐	☐	☐
b)	Movement of the fingers	☐	☐	☐	☐	☐
c)	Movement of your wrist	☐	☐	☐	☐	☐
d)	Strength of your hand	☐	☐	☐	☐	☐
e)	Pain level of your hand	☐	☐	☐	☐	☐
f)	Sensation (feeling) of your hand	☐	☐	☐	☐	☐

32 If you would like to give us any additional information about your hand and wrist function, please do so here:

Thank you for completing this questionnaire!
Please return it to the REACTS team
using the pre-paid envelope.

REACTS

If you have any questions or would like any additional information, please contact Lisa Newington on:
ln@mrc.soton.ac.uk | 023 8077 7624 | 07866 997732

For peer review only

For peer review only - http://bmjopen.bmj.com/site/about/guidelines.xhtml

SUPPLEMENTARY TABLES

1. Cox proportional hazards univariate analyses of the association between demographic and general health factors and the duration of work absence after carpal tunnel release (non-significant findings)

	N	Median time to return to work (days)	Inter quartile range	Hazards ratio [1]	95% Confidence intervals	P value
Body mass index (kg/m²)						
Normal (18.5-24.9)	47	20	12-33	1	-	-
Over (25.0-29.9)	64	19	12.5-29	0.98	0.65, 1.46	0.91
Obese (≥30)	67	21	10-40	0.88	0.60, 1.28	0.50
Surgery to dominant hand [2]						
Yes	128	19.5	12-33	1	-	-
No	62	22	12-35	0.83	0.61, 1.14	0.25
General health status						
Excellent/very good/ good	165	19	12-32	1	-	-
Fair/poor	24	28.5	12.5-42	0.74	0.47, 1.16	0.19
Number of comorbidities [3]						
None	49	18	7-32	1	-	-
One	67	24	14-42	0.87	0.59, 1.27	0.47
Two or more	74	19.5	10-29	1.11	0.76, 1.62	0.60
Number of disability comorbidities [3]						
None	130	19	9-33	1	-	-
One	39	26	13-31	0.75	0.52, 1.08	0.12
Two or more	21	20	14-31	0.81	0.50, 1.32	0.40
Number of somatising symptoms [4]						
None	96	19	10.5-35	1	-	-
One	51	22	13-34	0.98	0.70, 1.38	0.91
Two or more	42	19.5	14-31	0.84	0.58, 1.22	0.36
SF36 Mental health score (tertiles) [5]						
Poor (22.2-57.8)	60	24	16-39	0.71	0.50, 1.03	0.07
Intermediate (60.0-75.6)	68	18.5	11-36.5	0.88	0.62, 1.25	0.48
Good (77.8-97.8)	60	16.5	7-30	1	-	-

Total sample size n=195.

1. Adjusted for age and sex.

2. Considered and the non-dominant hand for those who reported ambidexterity (n=4) and dominant hand for those undergoing simultaneous bilateral surgery (n=2).

3. Adapted from the self-administered co-morbidity questionnaire [1].

4. Adapted from the four-dimensional symptom questionnaire [2].

5. Taken from the SF-36 with data-driven tertiles [3].

For peer review only

2. Cox proportional hazards univariate analyses of the association between health beliefs and the duration of work absence after carpal tunnel release (non-significant findings)

	N	Median time to return to work (days)	Inter quartile range	Hazards ratio [1]	95% Confidence intervals	P value
Believe will be unable to use hand normally in 3 months [2]						
No	168	20	12-32.5	1	-	-
Yes	20	20.5	14.5-40	1.04	0.65, 1.67	0.87
Blames self for hand problem [2]						
No	162	20	12-35	1	-	-
Yes	26	22	14-31	1.08	0.70, 1.66	0.73
Lack of support from family/friends [2]						
No	156	20	12-32.5	1	-	-
Yes	32	19.5	13-40	0.88	0.60, 1.29	0.51
Problem runs in the family/born with a weakness [3]						
No	134	20	12-35	1	-	-
Agree/strongly agree	54	23	12-31	0.88	0.63, 1.21	0.42
Stress made the problem worse [3]						
No	169	20	12-33	1	-	-
Agree/strongly agree	19	28	14-40	0.79	0.48, 1.28	0.33
Lack of exercise contributed to the problem [3]						
No	172	20	13-33	1	-	-
Agree/strongly agree	16	15	10.5-34.5	1.23	0.73, 2.06	0.43
Getting older contributes to the problem [3]						
No	79	26	14-42	1	-	-
Agree/strongly agree	108	16	10-29	1.28	0.94, 1.74	0.11
Work probably didn't cause the problem, but made it worse [3]						
No	100	20.5	12-35	1	-	-
Agree/strongly agree	86	19.5	13-31	1.01	0.75, 1.35	0.95
Pain catastrophisation to at least a moderate degree [4]						
No	133	19	10-33	1	-	-
Yes	55	22	15-31	0.81	0.59, 1.12	0.21

Total sample size n=195.

1. Adjusted for age and sex.

2. Reported on a 0-10 scale and dichotomised to no (0-6) and yes (7-10) [4].

3. Reported on a 5-point scale and dichotomised to agree/strongly agree and neither agree nor disagree/disagree/disagree strongly [5].

4. Pain catastrophizing scale dichotomised to those who reported catastrophising pain thoughts and feelings to at least a moderate degree in response to all questions and those who did not [6].

For peer review only

3. Cox proportional hazards univariate analyses of the association between clinical and surgical factors and the duration of work absence after carpal tunnel release (non-significant findings)

	N	Median time to return to work (days)	Inter quartile range	Hazards ratio [1]	95% Confidence intervals	P value
Katz hand diagram score for side of surgery [2]						
Classic/probable	126	22	14-38	1	-	-
Possible/unlikely	60	16.5	9.5-30.5	1.10	0.81, 1.51	0.54
Duration of symptoms						
≤ 1 year	47	19	10-31	1	-	-
> 1 year	141	20	13-34	0.86	0.62, 1.21	0.38
Nerve conduction studies performed						
No	71	21	14-35	1	-	-
Yes	79	20	11-33	1.14	0.83, 1.58	0.41
Type of suture material						
Non-absorbable	119	21	12-38	1	-	-
Absorbable	23	20	13-29	1.15	0.73, 1.83	0.55
MHQ function score for side of surgery (tertiles) [3]						
Poor (0-40)	79	20	13-41	0.80	0.57, 1.12	0.19
Intermediate (44-55)	48	19.5	13.5-33	0.81	0.55, 1.19	0.29
Good (60-100)	61	20	7-29	1	-	-
MHQ bilateral activities of daily living score (tertiles) [3]						
Poor (0-61)	72	19	12.5-33.5	0.96	0.66, 1.38	0.81
Intermediate (64-82)	55	21	13-38	0.70	0.49, 1.01	0.06
Good (83-100)	62	20	10-31	1	-	-
MHQ activities of daily living score for side of surgery (tertiles) [3]						
Poor (0-55)	63	20	12-42	0.81	0.56, 1.18	0.27
Intermediate (58-80)	63	20	14-35	0.86	0.61, 1.23	0.42
Good (85-100)	63	20	9-32	1	-	-
MHQ satisfaction score for side of surgery (tertiles) [3]						
Poor (0-25)	88	21.5	13-42	0.78	0.55, 1.11	0.16
Intermediate (29-50)	49	18	14-29	0.87	0.59, 1.28	0.48
Good (54-100)	53	20	9-31	1	-	-
Satisfaction with appearance for side of surgery [4]						
Satisfied	136	20	10.5-31.5	1	-	-
Dissatisfied	53	20	13-40	0.84	0.61, 1.16	0.29

Total sample size n=195.

1. Adjusted for age and sex.
2. Adapted from Calfee et al. [7].
3. Michigan Hand Questionnaire [8]. Data-driven tertiles.
4. Reported on a 5-point scale in response to the statement: I am satisfied with the appearance (look) of my hand. Dichotomised as satisfied (strongly agree/agree) and dissatisfied (neither agree nor disagree, disagree, strongly disagree).

For peer review only

4. Cox proportional hazards univariate analyses of the association between occupational factors and the duration of work absence after carpal tunnel release (non-significant findings)

	N	Median time to return to work (days)	Inter quartile range	Hazards ratio [1]	95% Confidence intervals	P value
Have more than one paid job						
No	177	20	12-32	1	-	-
Yes	12	26.5	19-42	0.61	0.34, 1.11	0.11
Total paid work hours per week [2]						
≤37.5	97	22	15-38	1	-	-
>37.5	93	16	6-29	1.25	0.88, 1.79	0.21
Number of work days per week						
<5	55	23	15-35	1	0.72, 1.39	0.98
5	116	19	10.5-33	1	-	-
>5	19	13	6-31	1.28	0.77, 2.14	0.34
Sick leave taken for this problem in the last month						
No	154	20	12-33	1	-	-
Yes	20	15	9.5-30	0.99	0.61, 1.60	0.95
Sick leave taken for another problem in the last month						
No	152	19	10.5-31	1	-	-
Yes	18	30	21-42	0.67	0.41, 1.09	0.10
Received advice about return to work after surgery						
Yes	134	19	12-31	1	-	-
No	53	22	14-41	0.92	0.67, 1.27	0.61
Required to work to tight deadlines [3]						
No	75	23	14-40	1	-	-
Yes	112	18	9.5-31	1.19	0.89, 1.61	0.24
Use power tools that make the hand/arm vibrate [3]						
No	145	19	12-32	1	-	-
Yes	38	22	14-42	0.68	0.44, 1.05	0.08
Work with hands above shoulder height for >1 hour [3]						
No	147	20	12-33	1	-	-
Yes	34	19.5	13-33	0.89	0.60, 1.32	0.56
Work with neck bent forward for >2 hours [3]						
No	114	21	13-39	1	-	-
Yes	73	18	8-31	1.19	0.88, 1.60	0.26
Work with neck twisted for >30 minutes [3]						
No	136	20	12-34	1	-	-
Yes	47	22	13-32	0.93	0.66, 1.32	0.70
Drive for >1 hour per day [3]						
No	115	21	13-38	1	-	-
Yes	70	16	7-31	1.22	0.88, 1.68	0.24

Total sample size n=195.
[1.] Adjusted for age and sex.
[2.] Dichotomised by the median for the sample population
[3.] As part of the normal working day [9].

For peer review only

References

1. Sangha O, Stucki G, Liang MH et al. The self-administered comorbidity questionnaire: a new method to assess comorbidity for clinical and health services research *Arthritis & Rheumatism* 2003;49(2):156-63.
2. Terluin B, van Marwijk HW, Adèr HJ et al. The Four-Dimensional Symptom Questionnaire (4DSQ): a validation study of a multidimensional self-report questionnaire to assess distress, depression, anxiety and somatization. *BMC Psychiatry* 2006;6(1):34.
3. Ware J, Sherbourne C. The MOS 36-item short-form health survey (SF-36): I. conceptual framework and item selection. *Medical Care* 1992;30:473-83.
4. Hansen TB, Dalsgaard J, Meldgaard A et al. A prospective study of prognostic factors for duration of sick leave after endoscopic carpal tunnel release. *BMC Musculoskelet Disord* 2009;10:144.
5. Palmer K, Calnan M, Wainwright D et al. Upper limb pain in primary care: health beliefs, somatic distress, consulting and patient satisfaction. *Family Practice* 2006;23(6):609-17.
6. Bot AG, Becker SJ, Bruijnzeel H et al. Creation of the abbreviated measures of the Pain Catastrophizing Scale and the Short Health Anxiety Inventory: the PCS-4 and SHAI-5. *J Musculoskelet Pain* 2014;22(2):145-51.
7. Calfee RP, Dale AM, Ryan D et al. Performance of simplified scoring systems for hand diagrams in carpal tunnel syndrome screening. *J Hand Surg Am* 2012;37(1):10-17.
8. University of Michigan Medical School. The Michigan Hand Questionnaire: http://mhq.lab.medicine.umich.edu/. Accessed 04/04/2019.
9. Karasek R, Brisson C, Kawakami N et al. The Job Content Questionnaire (JCQ): an instrument for internationally comparative assessments of psychosocial job characteristics. *J Occup Health Psychol* 1998;3(4):322-55.

For peer review only

STROBE checklist (cohort study)

Item	Recommendation	Location
**Title and abstract**		
Title	Indicate study design	Title
**Introduction**		
Background	Explain scientific background and rationale	Introduction
Objectives	State objectives and any pre-specified hypotheses	Introduction
**Methods**		
Study design	Present key elements of the study design early	Introduction & methods
Setting	Describe the setting, locations and relevant dates	Methods
Participants	Give eligibility criteria, sources and methods of selection of participants, and methods of follow-up	Methods & table 1
Variables	Clearly define all outcomes, exposures, predictors, potential confounders and effect modifiers	Methods & supplementary files 1-2
Data sources	Give sources of data and methods of assessment	Methods & tables 2-5
Bias	Describe and effects to address potential sources of bias	Methods & discussion
Study size	Explain how the study size was determined	Methods
Quantitative variables	Described how quantitative variables were handled in the analyses	Tables 2-5
Statistical methods	Described all statistical methods<br>Explain how missing data were addressed<br>Explain how lost to follow-up was addressed	Statistical methods & tables 2-5
**Results**		
Participants	Reported the number of individuals at each stage of the study<br>Give reasons for non-participation	Figure 1<br>Figure 1
Descriptive data	Give characteristics of study participants<br>Indicate number of participants for each variables of interest<br>Summarise follow-up time	Tables 2-5<br>Tables 2-5<br>Results
Outcome data	Report number of outcome events over time	Results
Main results	Give adjusted and unadjusted estimates and measures of precision. Make clear which confounders were adjusted for	Table 4 & supplementary file 3
Other analyses	Reported any subgroup analyses	Table 4 & supplementary file 3

For peer review only

BMJ Open

Sickness absence after carpal tunnel release: a multi-centre prospective cohort study

Journal:	*BMJ Open*
Manuscript ID	bmjopen-2020-041656.R2
Article Type:	Original research
Date Submitted by the Author:	09-Jan-2021
Complete List of Authors:	Newington, Lisa; University of Southampton Faculty of Medicine, MRC Versus Arthritis Centre for Musculoskeletal Health and Work, MRC Lifecourse Epidemiology Unit; Guy's and Saint Thomas' NHS Foundation Trust, Hand Therapy<br>Ntani, G; University of Southampton Faculty of Medicine, MRC Versus Arthritis Centre for Musculoskeletal Health and Work, MRC Lifecourse Epidemiology Unit<br>Warwick, David; University Hospital Southampton NHS Foundation Trust, Hand Surgery; University of Southampton Faculty of Medicine<br>Adams, Jo; University of Southampton, Centre for Innovation and Leadership in Health Sciences, School of Health Sciences, Faculty of Environment and Life Sciences; University of Southampton, Centre for Sport, Exercise and Osteoarthritis Research Versus Arthritis<br>Walker-bone, Karen; University of Southampton Faculty of Medicine, MRC Versus Arthritis Centre for Musculoskeletal Health and Work, MRC Lifecourse Epidemiology Unit
**Primary Subject Heading**:	Surgery
Secondary Subject Heading:	Epidemiology, General practice / Family practice, Occupational and environmental medicine
Keywords:	Hand & wrist < ORTHOPAEDIC & TRAUMA SURGERY, PRIMARY CARE, OCCUPATIONAL & INDUSTRIAL MEDICINE

SCHOLARONE™
Manuscripts

I, the Submitting Author has the right to grant and does grant on behalf of all authors of the Work (as defined in the below author licence), an exclusive licence and/or a non-exclusive licence for contributions from authors who are: i) UK Crown employees; ii) where BMJ has agreed a CC-BY licence shall apply, and/or iii) in accordance with the terms applicable for US Federal Government officers or employees acting as part of their official duties; on a worldwide, perpetual, irrevocable, royalty-free basis to BMJ Publishing Group Ltd ("BMJ") its licensees and where the relevant Journal is co-owned by BMJ to the co-owners of the Journal, to publish the Work in this journal and any other BMJ products and to exploit all rights, as set out in our licence.

The Submitting Author accepts and understands that any supply made under these terms is made by BMJ to the Submitting Author unless you are acting as an employee on behalf of your employer or a postgraduate student of an affiliated institution which is paying any applicable article publishing charge ("APC") for Open Access articles. Where the Submitting Author wishes to make the Work available on an Open Access basis (and intends to pay the relevant APC), the terms of reuse of such Open Access shall be governed by a Creative Commons licence – details of these licences and which Creative Commons licence will apply to this Work are set out in our licence referred to above.

Other than as permitted in any relevant BMJ Author's Self Archiving Policies, I confirm this Work has not been accepted for publication elsewhere, is not being considered for publication elsewhere and does not duplicate material already published. I confirm all authors consent to publication of this Work and authorise the granting of this licence.

For peer review only

TITLE

Sickness absence after carpal tunnel release: a multi-centre prospective cohort study

AUTHORS

Lisa Newington [1, 2] *

Georgia Ntani [1]

David Warwick [3]

Jo Adams [4, 5]

Karen Walker-Bone [1]

AFFILIATIONS

1. MRC Versus Arthritis Centre for Musculoskeletal Health and Work, MRC Lifecourse Epidemiology Unit, University of Southampton, Southampton, UK.
2. Hand Therapy, Guy's and St Thomas' NHS Foundation Trust, St Thomas' Hospital, London, UK.
3. Hand Surgery, University Hospital Southampton NHS Foundation Trust and Faculty of Medicine, University of Southampton, Southampton, UK.
4. Centre for Innovation and Leadership in Health Sciences, School of Health Sciences, Faculty of Environment and Life Sciences, University of Southampton, Southampton, UK.
5. Centre for Sport, Exercise and Osteoarthritis Research Versus Arthritis, University of Southampton, Southampton, UK.

*** Corresponding author**

Address: Hand Therapy, Guy's and St Thomas' NHS Foundation Trust, St Thomas' Hospital, London, SE1 7EH, UK.

Email: l.newington@nhs.net

Telephone: 0207 7188 4174

Fax: none

KEY WORDS

Carpal tunnel syndrome, carpal tunnel release, return to work, advice, sickness absence, cohort study

Word count: 3,999

For peer review only

TITLE

Sickness absence after carpal tunnel release: a multi-centre prospective cohort study

ABSTRACT

Objectives: To describe when patients return to different types of work after elective carpal tunnel release (CTR) surgery and identify the factors associated with the duration of sickness absence.

Design: Multi-centre prospective observational cohort study.

Setting and participants: Participants were recruited pre-operatively from 16 UK centres and clinical, occupational and demographic information were collected. Participants completed a weekly diary and questionnaires at four and 12 weeks post-operatively.

Outcomes: The main outcome was duration of work absence from date of surgery to date of first return to work.

Results: 254 participants were enrolled in the study and 201 provided follow-up data. Median duration of sickness absence was 20 days (range 1-99). Earlier return to work was associated with having surgery in primary care and a self-reported work role involving more than four hours of daily computer use. Being female and entitlement to more than a month of paid sick leave were both associated with longer work absences. Duration of work absence was strongly associated with the expected duration of leave, as reported by participants before surgery. Earlier return to work was not associated with poorer clinical outcomes reported 12 weeks after CTR.

For peer review only

Conclusions: There was wide variation in the duration of work absence after CTR across all occupational categories. A combination of occupational, demographic and clinical factors was associated with the duration of work absence, illustrating the complexity of return to work decision-making. However, pre-operative expectations were strongly associated with the actual duration of leave. We found no evidence that earlier return to work was harmful. Clear, consistent advice from clinicians pre-operatively setting expectations of a prompt return to work could reduce unnecessary sickness absence after CTR. To enable this, clinicians need evidence-informed guidance about appropriate timescales for the safe return to different types of work.

STRENGTHS AND LIMITATIONS OF THIS STUDY

- This multi-centre, prospective study with a large sample size provides robust evidence to understand return to work issues after carpal tunnel release surgery.
- Participants were recruited from primary care, secondary care and private practice sites, representing the breath of locations where carpal tunnel release is performed in the UK.
- Work absence was the primary outcome and a clear definition was used for its duration with data collected contemporaneously to limit recall bias.
- A standardised method was used to categorise occupations and measure occupational exposures, although this relied on job title, which may not be a true reflection of actual occupational activity.
- All participants underwent open carpal tunnel release, however the method of carpal tunnel syndrome diagnosis was not independently verified and different case definitions for carpal tunnel syndrome may have been included.

For peer review only

INTRODUCTION

Carpal tunnel syndrome (CTS) occurs when the median nerve becomes compressed within the carpal tunnel at the wrist. Typical sensory symptoms include pain, paraesthesia and/or numbness in the thumb, index, middle and radial half of the ring finger; and motor symptoms include progressive wasting of the thenar muscles. CTS is often associated with marked functional difficulty[1] and treatment is targeted at reducing the median nerve compression by splinting, corticosteroid injection or carpal tunnel release (CTR) surgery[2, 3].

Recent estimates suggest that more than 90,000 CTR procedures will be performed annually in the English NHS by 2025[4], and as the peak incidence for CTS falls within the working lifetime[5], many of these patients will need to return to work after their CTR. However, there is currently no evidence on which to base recommendations about when it might be safe to return to functional activities, including work, after carpal tunnel release (CTR). Our previous survey of UK hand surgeons, primary care surgeons and hand therapists found that clinicians recommended a wide range of times to return to three specified job roles after CTR: 0-30 days for desk-based work (e.g. keyboard, mouse, writing and telephone); 1-56 days for repetitive light manual work (driving, delivery, stacking); and 1-90 days for heavy manual work (e.g. construction)[6]. However, there has not previously been a prospective study of CTR patients in the UK in which time to return to work was the primary outcome. Therefore, it is not known when UK patients return to different occupational activities after CTR or what influences the duration of work absence. It is also unclear whether earlier return to work has a detrimental effect on post-operative clinical outcomes. Possible consequences of returning to work too soon after CTR include wound dehiscence, infection, and delayed healing. Conversely, delayed return to work may increase the risk of progression to long-term sick leave[7] and produce a financial burden for the individual, employer or state.

For peer review only

A systematic review of the prognostic factors associated with return to work after CTR identified 11 studies which evaluated more than 90 potential prognostic factors[8]. Longer durations of work absence after CTR were found to be associated with: receipt of workers' compensation[9]; manual work[10-12]; longer expected durations of work absence[10]; being on sick leave before CTR surgery[13]; self-blame for the hand problem[13]; and beliefs that the symptoms were caused by work[12].

Much of the existing research has been conducted at single sites and involved small numbers of participants. Furthermore, very few studies have specifically explored the influence of a range of occupational factors. The current multi-centre prospective cohort study was designed to explore when patients returned to different types of work after CTR and the demographic, clinical and occupational factors associated with duration of work absence. We also investigated whether earlier return to work was associated with poorer clinical outcomes assessed at 12 weeks after CTR.

METHODS

This was a prospective cohort study and a convenience sample of participants were recruited from 16 sites in England and Wales between March 2017 and August 2018 (ethics approval: IRAS 209840, 16/WA/0390). Recruitment took place before CTR surgery, either at the time of listing for surgery, during pre-operative screening, or on the day of surgery. At each site, the patient CTR pathway and treatment was carried out as usual. Sites were NHS secondary care (hospital setting), NHS primary care and private hand surgery facilities, representing the range of UK healthcare facilities where CTR is performed. Provision of CTR in the UK was explored through discussion with relevant national organisations (British Society for Surgery of the Hand

For peer review only

and Association for Surgeons in Primary Care). Sites were recruited through National Institute for Health Research infrastructure.

Eligibility criteria are shown in Table 1. Baseline demographic, general health and occupational information was collected via self-completed questionnaire at the time of recruitment. The questionnaire also included standardised measures of CTS symptoms[14-16] and hand function[17]. Questionnaire content was informed by the clinical, demographic and occupational factors previously identified in a systematic review of prognostic factors for return to work after CTR[8], and developed in collaboration with our patient advisory group. The reasoning for item inclusion is provided in supplementary file 1.

TABLE 1. Study eligibility criteria

**Self-selected by potential participants**
1. Aged over 18 and referred for carpal tunnel release surgery
2. Routinely work in paid employment for at least 20 hours per week
3. Plan to return to work after carpal tunnel release surgery
4. Have not previously had carpal tunnel release surgery on either hand
5. Have not previously had a serious injury to the same wrist/hand
**Assessed by recruiting clinician**
1. No planned surgical procedures for conditions other than carpal tunnel syndrome

Follow-up questionnaires were completed four and 12 weeks after CTR and collected information about return to work, work functioning, scar symptoms, CTS symptoms, and hand function. Study questionnaires are provided as supplementary files 2-3. Participants were also asked to complete a short weekly diary from the day after surgery until return to work, detailing whether they had returned to work that week, and if so, the date of return. Steps were taken to minimise loss to follow-up after recruitment. To maximise retention, we

For peer review only

incentivised with a shopping voucher on completion of the study (£10) and sent up to three reminders using a combination of post, email and text.

Surgical information was collected from the medical records by a member of the participant's clinical team. This included: date of CTR, operated hand(s), nature of anaesthetic, incision size, additional procedures, unexpected findings and suture material. Date, side of CTR and anaesthetic (general/local) were also reported by participants for cross-checking.

Public and patient involvement

This research was supported by a patient advisory group consisting of six individuals who had previously undergone CTR at different UK sites. Study questionnaires were developed in collaboration with the patient advisors and these individuals also provided their feedback on the preliminary findings.

Statistical methods

Comparisons were made between those who dropped out of the study before providing any follow-up data and those in the final study sample using pre-specified demographic, clinical and occupational variables (Table 2).

Manual and non-manual work was coded from job title and industry using the UK Standard Occupational Classification[18, 19]. Return to work time was calculated from the date of surgery to the date of first return to work (as reported by participants).

A Cox proportional hazards model was used to explore the factors associated with return to work time, and the assumptions of the model were tested. Baseline and operative variables

For peer review only

were assessed in univariable analyses and those which were significant ($p<0.05$) were included as covariates in the final model. All regression analyses were adjusted for age and sex.

Participants were defined as having a poor outcome if they reported one or more of the following: global rating of change score of "worse", "unchanged" or "slightly improved" (12 weeks after CTR)[20]; scar symptoms described as "unbearable", "very troublesome" or "fairly troublesome" (12 weeks after CTR); use of antibiotics for an incision site infection after returning to work; and additional sick-leave related to the CTR after returning to work. The duration of work absence for those with/without poor outcomes were compared using Wilcoxon rank-sum test. In addition, participants were dichotomised to those who returned to work within/after seven, 14 and 28 days of surgery and the prevalence rates of poor outcomes were compared using Chi^2 for each time period. These time points were based on the median clinician-recommended return to work time that we reported previously[6].

There was no imputation for missing data. Missing values were coded as a separate category for each of the variables included, and participant numbers are provided for each variable in the accompanying tables.

RESULTS

A total of 254 individuals completed the baseline questionnaire and 201 (79%) provided follow-up data. Participant numbers and loss to follow-up for each study component are shown in Figure 1. Participant demographics and comparisons between those who remained in the study and those who dropped out before follow-up are shown in Table 2.

Figure 1. Participant numbers for each stage of the cohort study

For peer review only

TABLE 2. Participant demographics assessed at baseline in comparison with those lost to follow-up

	**Study participants n=201 (%)**	**Lost to follow-up n=53 (%)**
Mean age in years (SD)	52.0 (9.16)	44.4 (9.55)
Sex		
Male	76 (37.8)	20 (37.7)
Female	125 (62.2)	33 (62.3)
Body mass index (kg/m²)		
Normal (18.5-24.9)	48 (23.9)	9 (17.0)
Overweight (25-29.9)	66 (32.8)	16 (30.2)
Obese (≥30)	73 (36.3)	22 (41.5)
Smoking status		
Never smoked	109 (54.2)	26 (49.1)
Current/ex-smoker	90 (44.8)	27 (50.9)
General health		
Excellent/very good/good	174 (86.6)	42 (79.3)
Fair/poor	26 (12.9)	11 (20.8)
Number of comorbidities		
None	54 (26.9)	21 (39.6)
One	70 (34.8)	13 (24.5)
Two or more	77 (38.3)	19 (35.9)
Number of disabling comorbidities		
None	138 (68.7)	35 (66.0)
One	41 (20.4)	9 (17.0)
Two or more	22 (11.0)	9 (17.0)
Mean SF-36 mental health score (SD) [a]	65.6 (17.20)	60.3 (20.41)
Mean bilateral CTS-6 score (SD) [b]	2.8 (0.77)	3.0 (0.73)
Mean MHQ bilateral activities of daily living score (SD) [c]	68.8 (23.64)	55.7 (28.62)
Mean MHQ work function score (SD) [c]	66.1 (22.26)	60.6 (22.61)
Type of job contract		
Employed (permanent contract)	164 (81.6)	37 (69.8)
Self-employed	31 (15.4)	13 (24.5)
Employed (temporary or zero hours contract)	5 (2.5)	3 (5.7)
Type of work [d]		
Manual	77 (39)	31 (58)
Non-manual	123 (61)	22 (42)
Median level of job demand on hands/wrists (IQR) [e]	9 (7-10)	10 (7-10)
Job satisfaction		
Very satisfied	87 (43.3)	24 (45.3)
Satisfied/fairly satisfied	92 (45.8)	24 (45.3)
Dissatisfied/very dissatisfied	20 (10.0)	5 (9.4)
Median expected work absence in days (IQR)	14 (7-28)	14 (5-21)
Expected availability of sick pay		
≤1 month	50 (24.9)	21 (39.6)
>1 month	94 (46.8)	11 (20.8)
Unsure	57 (28.4)	21 (39.6)

For peer review only

Study site [f]		
NHS primary care	73 (36.3)	13 (24.5)
NHS Secondary care	101 (50.3)	32 (60.4)
Private hand surgery facilities	27 (13.4)	8 (15.1)

[a.] SF-36 mental health score ranges from 0-100, higher scores indicate better mental health[21].
[b.] CTS-6 symptom score ranges from 1-5, higher scores indicate more severe symptoms[14].
[c.] Michigan Hand Questionnaire ranges from 0-100, higher scores indicate better functioning[17].
[d.] Classified using the Office for National Statistics Standard Occupational Classification 2010[18, 19].
[e.] Job demand scale range 0-10, 10 indicating very demanding on hands/wrists[13].
[f.] Location where the carpal tunnel release surgery was performed. Surgery in primary care was performed by General Practitioners who had completed additional training.

Eighty-six participants (43%) were recruited pre-operatively on the day of their CTR. For the remaining 115 participants, the median time between recruitment and CTR was 14 days (interquartile range [IQR] 5-40). The first follow-up questionnaire was completed a median of 32 days after CTR (IQR 29-38) and the final questionnaire was completed a median of 92 days after CTR (IQR 86-105). All participants underwent open CTR as a day case, and all but two had unilateral surgery. Another two participants required median nerve neurolysis, and one participant was noted to have a vascular abnormality. Sixty-two percent of participants (n=125) were expecting to have a CTR for their other hand in the future. Other baseline demographic and surgical factors are shown in Table 3.

TABLE 3. Participant demographic and surgical factors

	Number of participants n=201 (%)
Age (years)	
26-40	23 (11.4)
41-55	101 (50.3)
≥ 55	77 (38.3)
Hand dominance	
Right	178 (88.6)
Left	18 (9.0)
Ambidextrous	5 (2.5)
Side of surgery [a]	
Dominant hand	134 (66.7)
Non-dominant hand	65 (32.3)
Bilateral surgery	2 (1.0)
Surgical speciality and grade	

For peer review only

Consultant (plastics/orthopaedics)	64 (31.8)
Registrar (plastics/orthopaedics)	33 (16.4)
General practitioner	62 (30.9)
Not reported	42 (20.9)
Incision type [b]	
Mini open incision	129 (64.2)
Traditional incision	2 (1.0)
Not reported	70 (34.8)
Suture material	
Absorbable	24 (11.9)
Non-absorbable	126 (62.7)
Not reported	51 (24.4)

[a.] Considered as the non-dominant hand for those who reported ambidexterity.
[b.] Mini open incision defined as distal to the distal wrist crease and traditional open excision extending proximally.

The majority of participants (62%) worked five days per week (range 2-7) and the median number of weekly paid work hours was 37.5 (IQR 31-45). Two participants (1%) did not return to work during the 12-week study period: one reported that they had been made redundant and the other that their job was no longer available. Four participants (2%) had incomplete data (missing return to work date or CTR date) meaning that the duration of work absence could not be calculated. These six individuals were not included in the analyses of return to work time, leaving a total sample size of 195. An additional five participants reported that they had not returned to work, but planned to do so in the future. These individuals were included in the return to work analysis, censored to the time of last follow-up.

The median duration of work absence after CTR was 20 days (IQR 12-33). Manual workers took longer to return than non-manual workers: 23.5 days (IQR 14-41) compared with 18 days (IQR 9-31). Those who were self-employed returned to work earlier than those who were employed: 13 days (IQR 6-19) compared with 22 days (IQR 14-38). Return to work times are shown in Figure 2. The majority of participants returned to work on a Monday (43%). Approximately 15% returned each day between Tuesday-Thursday, then ~5% returned each

For peer review only

day from Friday-Sunday. More than half of participants (59%) reported that they needed to modify their work duties to some extent when they first returned to work. Of these, 62% had resumed full duties within five weeks.

Univariable analyses of the relationship between baseline factors and the duration of work absence found 17 factors (age- and sex-adjusted) that were significantly associated with time to return to work and were entered into the multivariable model, in which five factors remained significant (Table 4). Sensitivity analyses confirmed that these factors remained independently significant in the model. Non-significant findings in the univariable analyses are provided in supplementary file 4. Having surgery in primary care and having a job with more than four hours of daily computer use were both associated with earlier return to work than their respective reference categories. Being female and having access to more than a month of paid sick leave were both associated with longer durations of work absence than their respected reference categories. Compared to those who expected to return within a week, there was a sequential increase in the likelihood of longer durations of work absence for those expecting to return between 7-14 days, 15-30 days and >30 days (Table 4), which showed a significant gradient effect ($p<0.001$).

For peer review only

TABLE 4. Cox proportional hazards analyses of the association between baseline demographic, clinical and occupational factors and the duration of work absence after carpal tunnel release

				Univariable analyses			Multivariable analysis		
	N	Median work absence (days)	Inter quartile range	Hazards ratio	95% Confidence intervals	P value	Hazard s ratio	95% Confidence intervals	P value
Sex (censored: 5 females; no missing data)									
Male	72	17.5	8-31	1	-	-	1	-	-
Female	118	21.5	14-35	0.79	0.59, 1.06	0.12	0.56	0.36, 0.88	**0.01**
Age in years (censored: 1 aged 26-40, 3 aged 41-55, 1 aged >55; no missing data)									
26-40	21	20	15-30	1.01	0.63, 1.63	0.96	1.44	0.82, 2.55	0.21
41-55	94	20	9-33	1	-	-	1	-	-
>55	75	17	12-35	1.03	0.76, 1.40	0.83	1.15	0.80, 1.65	0.44
Smoking status (censored: 1 never, 4 current/ex; 2 missing)									
Never	105	16	12-31	1	-	-	1	-	-
Current/ex	83	23	13-41	0.74	0.56, 1.00	0.046	0.75	0.51, 1.09	0.13
Site (censored: 5 NHS secondary care; no missing data)									
NHS Primary care	72	19.5	13-33	1.18	0.87, 1.62	0.29	1.54	1.05, 2.25	**0.03**
NHS Secondary care	92	20	12-39.5	1	-	-	1	-	-
Private facilities	26	20	7-28	1.63	1.04, 2.54	0.03	1.46	0.87, 2.44	0.15
Afraid of long-term hand problems [a] (censored: 1 no, 4 yes; 3 missing)									
No	105	19	12-31	1	-	-	1	-	-
Yes	82	20.5	13-42	0.69	0.51, 0.93	0.01	0.93	0.67, 1.30	0.68
CTS-6 score for side of surgery (tertiles) [b] (censored: 2 intermediate, 3 poor; 8 missing)									
Good (1-3.0)	65	16	10-27	1	-	-	1	-	-
Intermediate (3.2-3.8)	58	21.5	14-35	0.77	0.54, 1.10	0.15	1.19	0.77, 1.84	0.44
Poor (3.8-5)	59	24	13-41	0.67	0.47, 0.97	0.03	1.04	0.65, 1.66	0.87
Type of work contract (censored: 5 employed, 1 missing)									
Employed (permanent)	154	22	14-38	1	-	-	1	-	-
Self-employed	30	13	6-19	1.72	1.13, 2.61	0.01	1.19	0.67, 2.14	0.55
Zero hours/temporary	5	12	3-31	2.01	0.81, 5.00	0.13	0.73	0.25, 2.14	0.56
Duration of available sick pay (censored: 4 >1 month, 1 unsure; no missing data)									
≤1 month	49	16	12-29	1	-	-	1	-	-
>1 month	88	27	15-42	0.59	0.41, 0.85	0.004	0.46	0.28, 0.76	**0.002**
Unsure	53	16	10-23	1.19	0.80, 1.77	0.40	1.01	0.61, 1.66	0.97
Access to occupational health at work (censored: 1 no, 4 yes; 1 missing)									
No	110	15.5	9-29	1.77	1.31, 2.38	<0.001	1.42	0.91, 2.19	0.12
Yes	79	25	16-42	1	-	-	1	-	-
Expected duration of leave after carpal tunnel release (days) (censored: 1 7-14, 2 15-29, 2 ≥30; no missing data)									
<7	35	4	2-12	1	-	-	1	-	-
7-14	75	16	13-26	0.23	0.15, 0.36	<0.001	0.27	0.16, 0.45	**<0.001**
15-29	35	29	22-39	0.12	0.07, 0.19	<0.001	0.19	0.10, 0.37	**<0.001**
≥30	45	42	21-44	0.08	0.05, 0.14	<0.001	0.12	0.06, 0.23	**<0.001**
MHQ work functioning score (tertiles) [c] (censored: 3 poor, 2 intermediate; no missing data)									
Poor (0-55)	67	20	13-35	0.68	0.47, 0.98	0.04	0.83	0.50, 1.40	0.49
Intermediate (60-80)	72	21	12.5-39.5	0.77	0.53, 1.10	0.15	0.81	0.50, 1.31	0.39
Good (81-100)	51	17	10-29	1	-	-	1	-	-
Job satisfaction [d] (censored: 5 satisfied; 1 missing)									
Satisfied	169	19	11-31	1	-	-	1	-	-
Dissatisfied	19	38	21-43	0.61	0.38, 0.99	0.04	0.67	0.38, 1.16	0.15
Believe that the hand problem was caused by work [e] (censored: 1 no, 4 yes; 2 missing)									
No	112	19	13-31	1	-	-	1	-	-
Agree/strongly agree	76	23	10-42	0.62	0.46, 0.85	0.003	0.82	0.57, 1.17	0.28
Job is demanding on hands/wrists [a] (censored: 5 yes; no missing data)									
No	35	16	6-27	1	-	-	1	-	-
Yes	155	20	13-38	0.61	0.42, 0.89	0.01	0.68	0.42, 1.12	0.13

For peer review only

Type of work [f] *(censored: 2 non-manual, 3 manual; 1 missing)*									
Non-manual	119	18	9-31	1	-	-	1	-	-
	70	23.5	14-41	0.66	0.48, 0.89	0.01	0.97	0.57, 1.64	0.90
Work involves target-driven pay [g] *(censored: 3 no, 2 yes; 10 missing)*									
No	149	19	12-31	1	-		1	-	-
Yes	31	22	13-45	0.61	0.41, 0.91	0.02	0.97	0.59, 1.61	0.91
Duration of computer use at work (hours) [g] *(censored: 5 <1; 4 missing)*									
<1	69	28	17-42	1	-	-	1	-	-
>1 to <4	33	16	10-31	2.20	1.43, 3.38	<0.001	1.01	0.56, 1.81	0.98
≥4	84	16	7-27	2.38	1.67, 3.38	<0.001	1.85	1.08, 3.16	**0.03**
Work involves lifting or carrying ≥10 kg [g] *(censored: 4 no, 1 yes; 5 missing)*									
No	108	18.5	11-30	1	-	-	1	-	-
Yes	77	24	13-40	0.61	0.42, 0.86	0.01	0.80	0.48, 1.33	0.39
Work involves pushing/pulling a heavy weight [g] *(censored: 2 no, 3 yes; 2 missing)*									
No	104	16	8.5-28.5	1	-	-	1	-	-
Yes	83	26	16-42	0.51	0.37, 0.70	<0.001	0.97	0.61, 1.55	0.90

Total sample size n=195. Median work absence relates to the 190 non-censored events. All analyses were adjusted for age and sex. All significant variables in the univariable analyses ($p<0.05$) were entered into the multivariable model.

[a.] Reported on a 0-10 scale, dichotomised to no (0-6) and yes (7-10)[13].

[b.] CTS-6 score [14] with data driven tertiles.

[c.] Michigan Hand Questionnaire work performance subscale scored from 0-100, higher scores indicate better perceived work performance[17]. Data driven tertiles.

[d.] Reported on a 5-point scale, dichotomised to satisfied (very satisfied/satisfied/fairly satisfied) and dissatisfied (dissatisfied/very dissatisfied).

[e.] Reported on a 5-point scale and dichotomised to agree/strongly agree and neither agree nor disagree/disagree/disagree strongly[22].

[f.] Classified using the Office for National Statistics Standard Occupational Classification 2010[18, 19].

[g.] As part of the normal working day[23].

For peer review only

Clinical outcomes after CTR are shown in Table 5. Using the definition outlined in the methods, a total of 46 participants (24%) were identified as having at least one poor outcome (CTS symptoms that were worse, unchanged or only slightly better; scar symptoms that were at least fairly troublesome; required post-operative antibiotics or had additional time off work after first return). Of these participants, the majority (n=38, 83%) reported only a single component of poor outcome. Three individuals defined as having a poor outcome had not returned to work at the point of last follow-up (as compared with two individuals in the rest of the study sample). For those who had returned to work, the median duration of work absence for those with a poor outcome was 22 days (IQR 12-42) compared with 19 days (IQR 12-32) for those without (Figure 2). This difference was not significant (Wilcoxon rank-sum, p=0.24).

TABLE 5. Clinical outcomes after carpal tunnel release

	**Mean score (standard deviation)**	
	Before surgery	12 weeks after surgery
CTS-6 (operated hand) [a]	3.3 (0.87)	1.2 (0.54)
MHQ function (operated hand) [b]	50 (22.1)	79 (19.4)
MHQ satisfaction with function (operated hand) [b]	38 (25.7)	82 (21.3)
MHQ bilateral activities of daily living [b]	69 (23.7)	88 (13.8)
MHQ activities of daily living (operated hand) [b]	65 (28.1)	87 (18.5)
	**Number of participants (%)**	
Global rating of change score		
Completely cured	-	65 (33.3)
Much better	-	98 (50.3)
Slightly better	-	13 (6.7)
Unchanged	-	2 (1.0)
Worse	-	5 (2.6)
Scar symptom severity	-	
Not at all troublesome	-	99 (50.8)
A little troublesome	-	63 (32.3)
Fairly troublesome	-	18 (9.2)
Very troublesome	-	2 (1.0)
Unbearable	-	0
Required post-operative antibiotics	-	10 (5.1)
Additional sick leave after first returning to work	-	12 (6.2)

Grey shading indicates the categories which were combined to define a poor surgical outcome.
[a.] CTS-6 assessment of carpal tunnel syndrome symptoms[14]. Range 1-5: 1 equals no symptoms.
[b.] MHQ Michigan Hand Questionnaire[17]. Range 0-100: 100 equals no deficit or dissatisfaction.

For peer review only

Figure 2. Duration of work absence after carpal tunnel release

There was no significant difference in the prevalence of a poor outcome among those who returned to work within or after seven days of CTR (20% versus 24%, Chi^2 p=0.63). Similarly, there was no significant difference in the prevalence of a poor outcome among those who returned to work within or after 14 days (19% versus 25%, Chi^2 p=0.31), or within or after 28 days of CTR (21% versus 27%, Chi2 p=0.33).

DISCUSSION

In this multi-site prospective cohort study, the median duration of work absence was 20 days (range 1-99), a duration similar to that reported by other European studies[24]. Earlier return to work was associated with typing for ≥4 hours at work (as compared with more physical workplace tasks) and undergoing surgery in primary care (as compared with secondary care or private practice). Pre-operative expectations about return to work were important significant predictors of actual return to work times. We found no evidence of poorer clinical outcomes in the first 12 weeks amongst those who returned to work earlier. At each time point, fewer manual workers had returned to work than non-manual workers and fewer employed workers had returned than self-employed (Figure 2). Both findings have been reported previously[10, 11, 25, 26], however, neither the type of work (manual/non-manual) nor the type of work contract (employed/self-employed) were significantly associated with the duration of work absence in the mutually adjusted model. These results illustrate the importance of considering the range of relevant demographic, clinical and occupational factors, which may have been confounders, moderators or mediators in previous studies. The reported model does not look to predict the duration of work absence for future CTR patients, rather to explore the association between different risk factors and work absence.

For peer review only - http://bmjopen.bmj.com/site/about/guidelines.xhtml

Five variables remained statistically significantly associated with longer duration of work absence in the final model. Two were occupational factors: infrequent computer use and availability of sick pay. Cowan et al.[10], recorded earlier return to work after CTR for desk-based workers and we have shown previously that UK hand surgeons and hand therapists report that they advise earlier return to desk-based workers[6]. The relationship between longer duration of work absence and availability of sick pay has also be reported previously for those with and without worker's compensation[9, 24]. It is plausible that financial necessity is driving earlier return to work for those with limited sick pay. Alternatively, those with access to paid leave might choose to take the maximum available duration.

Only one clinical factor was significantly associated with the duration of work absence: participants who had their CTR surgery in primary care were more likely to return to work earlier than those whose procedure took place in an NHS hospital setting. One possible reason is that patients with more complex disease may be more commonly referred to secondary, rather than primary, care for their CTR and these patients may require longer off work after their surgery. However, in the current study the proportion and degree of comorbidities, and the extent of pre-operative symptoms reported by participants were similar across all settings. Another possibility is that the patients' expectations of the surgery may be different: CTR performed in a primary care setting might be perceived by patients as being more minor than surgery in a hospital operating theatre. Alternatively, the general practice surgeons may have recommended earlier return to work than those based in a hospital, although the median expected duration of work absence for participants in the current study was the same across all settings. The inclusion of CTR performed in primary care is a strength of the study. We

For peer review only - http://bmjopen.bmj.com/site/about/guidelines.xhtml

acknowledge that hand surgeons may also provide CTR services in primary care, as visiting clinicians, however in the current study this was not the case. CTR and other surgical procedures, such as vasectomy and minor skin surgery, are regularly performed by trained general practitioners in the UK[27], and all primary care surgeons in the current study were general practitioners who already provided a CTR service.

Only one demographic factor was statistically significant: women were more likely to take longer to return to work than men. Whilst we found inconsistent data about the effect of gender on return to work after CTR in the literature[28, 29], female gender has been associated with longer periods of work absence for other health conditions[30]. Researchers should continue to include sex as a covariate in analyses of return to work outcomes, although there is currently insufficient evidence to support any difference in return to work recommendations after CTR based on sex. Further qualitative investigation is required in order to understand the context for this.

Finally, those who expected to return to work more quickly were significantly more likely to do so. It has been shown previously that patient expectations are a prominent determinant of return to work time, or other return to work outcomes, for musculoskeletal or mental health conditions[30-32]. The role of expectations on outcomes, including the expected and actual timing for return to work and driving after hand and wrist surgery, requires further exploration, particularly because expectations are a potentially modifiable characteristic which could be influenced by the advice provided by clinicians pre-operatively.

For peer review only - http://bmjopen.bmj.com/site/about/guidelines.xhtml

In total, approximately a quarter of participants in this study were considered to have a poor outcome using our composite definition. We chose to use a multi-component definition which was deliberately very stringent to minimise the chances of missing any harm caused by early return to work. Our rates of poorer outcomes were in fact similar to those reported in other CTR populations[20, 33, 34]. Importantly, we found no relationship between earlier return to work and occurrence of poor outcomes within 12 weeks of CTR in this cohort study. We acknowledge that a longer follow-up duration would have aided the assessment of post-operative symptom resolution, however this was not possible with the resources available and was not a primary objective of the study.

There are a number of limitations of the current study, including the reliance on self-reported data. Work absence is not logged on a national database in the UK and therefore could only be obtained through self-report. To minimise errors of recall, date of return to work was determined contemporaneously. The recall duration for measures of function and symptoms was limited to a maximum of four weeks, consistent with the outcome measures used[14, 17]. We set out to recruit a large sample of working-aged adults undergoing CTR. Our prospectively recruited sample from 16 sites is one of the largest reported in the literature to date, with a good follow up response rate (79%), but it remains possible that we were under-powered to detect some of the factors which may have been associated with delayed return to work. Specifically, this could result where some levels of categorical variables of interest have lower prevalence, for example, the type of work contract (>80% of participants reported that they had a permanent work contract, compared with ~15% who were self-employed). Furthermore, we acknowledge that the inclusion of a large number of variables in the development of the final model may result in model overfitting, thereby potentially limiting generalisability.

For peer review only

We took the approach not to impute values where data were missing. Overall, the amount of missing data was small and at the individual item-level (Table 4 and supplementary file 4). Missing data were coded as such, and included in the analysis. We acknowledge that the approach taken to missing data may have resulted in biased estimates, yet if such effects are present, they are likely to be minimal due to low levels of missing data.

Following our *a priori* analysis plan, the association between each baseline variable and the duration of work absence was individually assessed in separate age- and sex-adjusted analyses. Only those variables which reached significance at the 5% level ($p<0.05$) were included in the multivariable model. In order to test the stability of our model, and to identify whether any potential associations had been missed, this was tested using 1% and 20% cut-offs. In both test scenarios, the findings were similar to those presented in our final model (Table 4), suggesting that our model is robust. However, we acknowledge that alternative methods of selecting variables for inclusion (such as forward inclusion or backward elimination) may have yielded slightly different results, particularly for variables that were close to our significance cut-off of 5%.

The findings may not be generalisable to working populations in regions outside of central and southern England and Wales, who are employed in other industries, or managed with a different CTR patient pathway. Steps were taken to include the main settings where CTR is performed in the UK, but we acknowledge that CTR may also be performed by other specialities. Individuals who chose to participate in the study may not be fully representative of the wider CTR population, and the observed differences between those who completed the

For peer review only - http://bmjopen.bmj.com/site/about/guidelines.xhtml

study and those who were lost to follow-up (younger, poorer mental health, more likely manual workers) also limit generalisability.

Endoscopic CTR has been associated with earlier return to work than open CTR [35], however it was not possible to assess this in the current study. At present, endoscopic CTR is not routinely performed in the UK[6]. Anecdotally, most providers will not fund the extra cost of endoscopic CTR, which requires extra equipment, longer operating times and more experienced surgeons. Recruitment to the current study was not limited to patients undergoing open CTR, but no endoscopic procedures were performed during the study at any of our sites.

All participants were presumed to have CTS as diagnosed by their treating clinician. Many studies of CTS include nerve conduction study (NCS) findings as part of their eligibility criteria, although this was not possible in the current study because NCS are not routinely recommended for pre-operative diagnosis of CTS in the UK[2]. Our eligibility criteria required that only people undergoing their first CTR were included and reported upon in this study (so that previous experiences with CTR were not potential confounders). However, more than three quarters of the cohort reported bilateral symptoms. The possible impact of persisting CTS symptoms in the non-operated hand on return to work also needs to be considered.

For the current study, we considered both occupational title and self-reported occupational exposures collected in a standardised questionnaire format[13, 18, 19, 23]. Categorisation based on job title and industry may not accurately reflect the physical and/or psychosocial aspects of job role. Furthermore, co-occurrence of occupational exposures may be more

For peer review only

common in some types of jobs than in others, for example lifting >10kg and pushing or pulling a heavy weight.

There is a need for an agreed approach to identifying and recording key physical demands and psychosocial exposures of jobs to enable consistent exploration of their impact on work and clinical outcomes following surgery or other intervention. Approaches such as job exposure matrices[36] could facilitate this in future research.

In summary, this large multi-centre prospective cohort study investigated when participants return to work after CTR. Expectations about return to work (reported before surgery) were strongly associated with actual work absence, regardless of the job role or self-reported upper limb activities involved. Patient expectations can be influenced by many factors, but one of the most important is the advice provided by clinicians, in particular the surgeon. This suggests that clear, consistent advice could have an important effect on duration of sick leave. To date there is no evidence-based guidance informing clinicians what to advise about returning to different types of work after CTR. Further research is required to reach a consensus and explore whether the provision of targeted, consistent and standardised advice can alter the expected duration of work absence, reducing unnecessary sick leave, without causing adverse effects on clinical outcomes.

For peer review only

REFERENCES

1. Jerosch-Herold C, Mason R, Chojnowski AJ. A qualitative study of the experiences and expectations of surgery in patients with carpal tunnel syndrome. *J Hand Ther* 2008;21(1):54-61 doi:10.1197/j.jht.2007.09.001.
2. British Orthopaedic Association. Commissioning guide: Treatment of carpal tunnel syndrome. London: Royal College of Surgeons; 2017.
3. American Academy of Orthopaedic Surgeons. Management of carpal tunnel syndrome evidence-based clinical practice guideline. Illinois: American Academy of Orthopaedic Surgeons; 2016.
4. Bebbington E, Furniss D. Linear regression analysis of Hospital Episode Statistics predicts a large increase in demand for elective hand surgery in England. *J Plast Reconstr Aesthet Surg* 2015;68(2):243-51 doi:10.1016/j.bjps.2014.10.011.
5. Burton CL, Chen Y, Chesterton LS et al. Trends in the prevalence, incidence and surgical management of carpal tunnel syndrome between 1993 and 2013: an observational analysis of UK primary care records. *BMJ Open* 2018;8(6) doi:10.1136/bmjopen-2017-020166.
6. Newington L, Francis K, Ntani G et al. Return to work recommendations after carpal tunnel release: a survey of UK hand surgeons and hand therapists. *J Hand Surg Eur* 2018;43(8):875-78 doi:10.1177/1753193418786375.
7. Black C. Working for a healthier tomorrow. London, UK: Health Work and Wellbeing Programme 2008.
8. Peters S, Johnston V, Hines S et al. Prognostic factors for return-to-work following surgery for carpal tunnel syndrome: a systematic review. *JBI Database Syst Rev Implement Rep* 2016;14(9):135-216 doi:10.11124/jbisrir-2016-003099.
9. Dunn JC, Kusnezov NA, Koehler LR et al. Outcomes following carpal tunnel release in patients receiving workers' compensation: a systematic review. *Hand* 2018;13(2):137-42 doi:10.1177/1558944717701240.
10. Cowan J, Makanji H, Mudgal C et al. Determinants of return to work after carpal tunnel release. *J Hand Surg Am* 2012;37(1):18-27
11. De Kesel R, Donceel P, De Smet L. Factors influencing return to work after surgical treatment for carpal tunnel syndrome. *Occup Med* 2008;58(3):187-90

For peer review only

12. Parot-Schinkel E, Roquelaure Y, Ha C et al. Factors affecting return to work after carpal tunnel syndrome surgery in a large french cohort. *Arch of Phys Med Rehabil* 2011;92(11):1863-69 doi:http://dx.doi.org/10.1016/j.apmr.2011.06.001.
13. Hansen TB, Dalsgaard J, Meldgaard A et al. A prospective study of prognostic factors for duration of sick leave after endoscopic carpal tunnel release. *BMC Musculoskelet Disord* 2009;10:144
14. Atroshi I, Lyren PE, Ornstein E et al. The six-item CTS symptoms scale and palmar pain scale in carpal tunnel syndrome. *J Hand Surg Am* 2011;36(5):788-94 doi:10.1016/j.jhsa.2011.02.021.
15. Calfee RP, Dale AM, Ryan D et al. Performance of simplified scoring systems for hand diagrams in carpal tunnel syndrome screening. *J Hand Surg Am* 2012;37(1):10-17 doi:10.1016/j.jhsa.2011.08.016.
16. Katz JN, Stirrat CR. A self-administered hand diagram for the diagnosis of carpal tunnel. *J Hand Surg Am* 1990;15:360-63
17. University of Michigan Medical School. The Michigan Hand Questionnaire: http://mhq.lab.medicine.umich.edu/. Accessed 04/04/2019.
18. Office for National Statistics. Standard occupational classification for the UK: https://www.ons.gov.uk/methodology/classificationsandstandards/standardoccupationalclassificationsoc/soc2010. Accessed 08/06/2018.
19. Warwick Institute for Employment Research. Cascot: Computer assisted structured coding tool: https://warwick.ac.uk/fac/soc/ier/software/cascot/. Accessed 15/05/2019.
20. Jerosch-Herold C, Shepstone L, Houghton J et al. Prognostic factors for response to treatment by corticosteroid injection or surgery in carpal tunnel syndrome (PaLMS study): a prospective multi-centre cohort study. *Muscle Nerve* 2019;60(1):32-40 doi:doi:10.1002/mus.26459.
21. Ware J, Sherbourne C. The MOS 36-item short-form health survey (SF-36): I. conceptual framework and item selection. *Medical Care* 1992;30:473-83
22. Palmer K, Calnan M, Wainwright D et al. Upper limb pain in primary care: health beliefs, somatic distress, consulting and patient satisfaction. *Family Practice* 2006;23(6):609-17 doi:10.1093/fampra/cml047.

For peer review only - http://bmjopen.bmj.com/site/about/guidelines.xhtml

23. Karasek R, Brisson C, Kawakami N et al. The Job Content Questionnaire (JCQ): an instrument for internationally comparative assessments of psychosocial job characteristics. *Journal Occup Health Psychol* 1998;3(4):322-55

24. Newington L, Stevens M, Warwick et al. Sickness absence after carpal tunnel release: a systematic review of the literature. *Scand J Work Environ Health* 2018;44(6):557-67 doi:10.5271/sjweh.3762

25. Atroshi I, Larsson G-U, Ornstein E et al. Outcomes of endoscopic surgery compared with open surgery for carpal tunnel syndrome among employed patients: randomised controlled trial. *BMJ* 2006;332(7556):1473 doi:10.1136/bmj.38863.632789.1F.

26. Chaise F, Bellemere P, Fril JP et al. Return-to-work interval and surgery for carpal tunnel syndrome. Results of a prospective series of 233 patients. *J Hand Surg Br* 2004;29(6):568-70

27. Association of Surgeons in Primary Care. Surgical proceedures in primary care: https://www.aspc-uk.net/. Accessed 24/11/2020

28. Atroshi I, Zhou C, Joud A et al. Sickness absence from work among persons with new physician-diagnosed carpal tunnel syndrome: a population-based matched-cohort study. *PLoS One* 2015;10(3):e0119795 doi:10.1371/journal.pone.0119795.

29. Bekkelund SI, Pierre-Jerome C, Torbergsen T et al. Impact of occupational variables in carpal tunnel syndrome. *Acta Neurol Scand* 2001;103(3):193-97

30. Cancelliere C, Donovan J, Stochkendahl MJ et al. Factors affecting return to work after injury or illness: best evidence synthesis of systematic reviews. *Chiropr Man Ther* 2016;24(1):32-32 doi:10.1186/s12998-016-0113-z.

31. Claréus B, Renström EA. Patients' return-to-work expectancy relates to their beliefs about their physician's opinion regarding return to work volition and ability. *J Pain Res* 2019;12:353-62 doi:10.2147/JPR.S179061.

32. Heymans MW, de Vet HC, Knol DL et al. Workers' beliefs and expectations affect return to work over 12 months. *Journal Occup Rehabil* 2006;16(4):685-95 doi:10.1007/s10926-006-9058-8.

33. Bland JDP. Treatment of carpal tunnel syndrome. *Muscle Nerve* 2007;36(2):167-71 doi:10.1002/mus.20802.

34. Louie D, Earp B, Blazar P. Long-term outcomes of carpal tunnel release: a critical review of the literature. *Hand* 2012;7(3):242-46 doi:10.1007/s11552-012-9429-x.

For peer review only

35. Li Y, Luo W, Wu G et al. Open versus endoscopic carpal tunnel release: a systematic review and meta-analysis of randomized controlled trials. *BMC Musculoskelet Disord* 2020;21:272 doi:10.1186/s12891-020-03306-1

36. Dale AM, Zeringue A, Harris-Adamson C et al. General population job exposure matrix applied to a pooled study of prevalent carpal tunnel syndrome. *Am Journal Epidemiol* 2015;181(6):431-39

Conflicts of interest

LN was supported by a National Institute for Health Research (NIHR) Doctoral Research Fellowship, grant number DRF-2015-08-056. GN was supported by funding from the MRC Versus Arthritis Centre for Musculoskeletal Health and Work. The authors declare no other conflicts of interest.

Funding

LN was supported by a National Institute for Health Research (NIHR) Doctoral Research Fellowship, grant number DRF-2015-08-056. GN was supported by funding from the MRC Versus Arthritis Centre for Musculoskeletal Health and Work. The views expressed are those of the authors and not necessarily those of the funders, the NHS, the NIHR or the Department of Health.

Informed consent

Written informed consent was obtained from the patient(s) for their anonymised information to be published in this article.

Ethics approval

For peer review only

For peer review only - http://bmjopen.bmj.com/site/about/guidelines.xhtml

Full ethics approval was granted by the NHS Health Research Authority (IRAS 209840: 16/WA/0390) and University of Southampton (ERGO 25757) Ethics Committees.

Contributorship

The study was devised by LN, KWB, JA, DW and DC. LN completed data collection and analysis, with assistance from GN and KWB. All authors contributed to the interpretation of the data. LN wrote the first draft of the manuscript. All authors reviewed and edited the manuscript and approved the final version.

Acknowledgements

The authors would like to thank and acknowledge all the study participants who gave up their time to take part in this study. Thank you also to the Patient Advisory Group for their contribution to the development of the research question and study material, and their thoughtful feedback on the preliminary findings. Thank you to Prof David Coggon for his assistance with the initial research idea, and to the Association of Surgeons in Primary Care and British Society for Surgery of the Hand for supporting the study.

Thank you to the study sites and local principal investigators: Jeremy Bland at East Kent Hospitals University NHS Foundation Trust; Darren Roberts at Portsmouth Hospitals NHS Trust; Mike Taylor and Claire Zweifel at Mid Essex Hospital Services NHS Trust; Frances Clark at Tollgate Clinic; Will Mason at Gloucester Hospitals NHS Foundation Trust; Vasileios Kefalas and Petros Mikalef at Care UK Southampton NHS Treatment Centre; Peter Sharpe at Salisbury Medical Practice; David Warwick at University Hospital Southampton NHS Foundation Trust and Nuffield Health Wessex Hospital; Alistair Phillips at Southern Health NHS Foundation Trust; Nanda Pillai at St Luke’s Surgery, Walsall; Tim Halsey and Effie Katsarma at Chelsea and

For peer review only

Westminster Hospital NHS Foundation Trust; Claire Middleton at Royal Berkshire NHS Foundation Trust; Duncan Avis and Ian MacLeod at Hampshire Hospital NHS Foundation Trust; Nick Gape and Lisa Small at Cardiff and Vale University Health Board; Kristin Francis at HCA Healthcare London Hand and Wrist Unit; and to all local team members who helped set up and recruit to the REACTS study.

For peer review only

Figure 1. Participant numbers for each stage of the cohort study

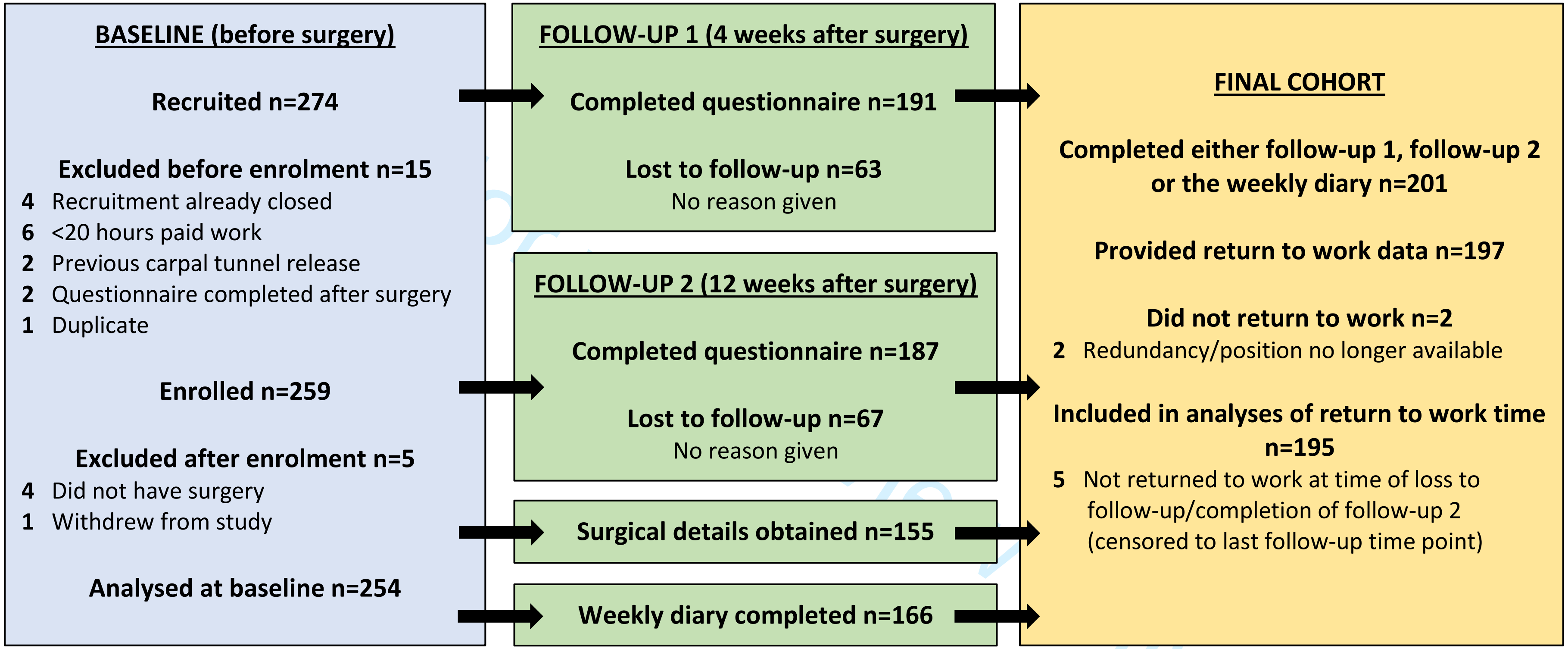

Figure 2. Duration of work absence after carpal tunnel release

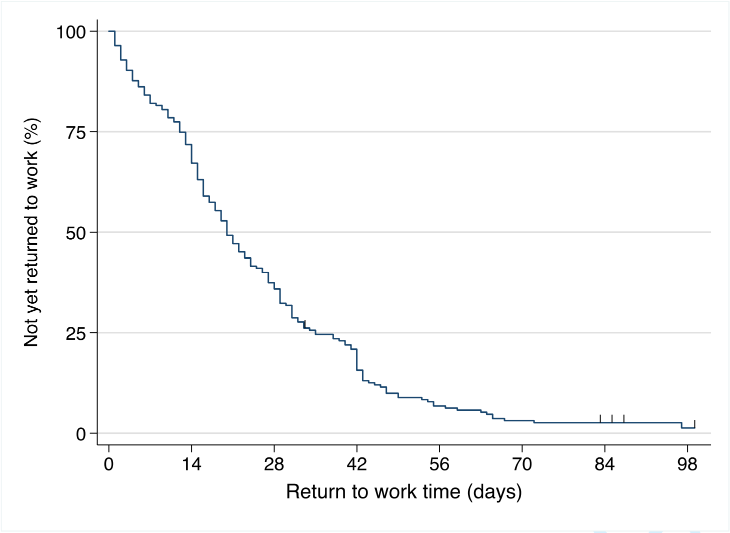

A. Whole cohort

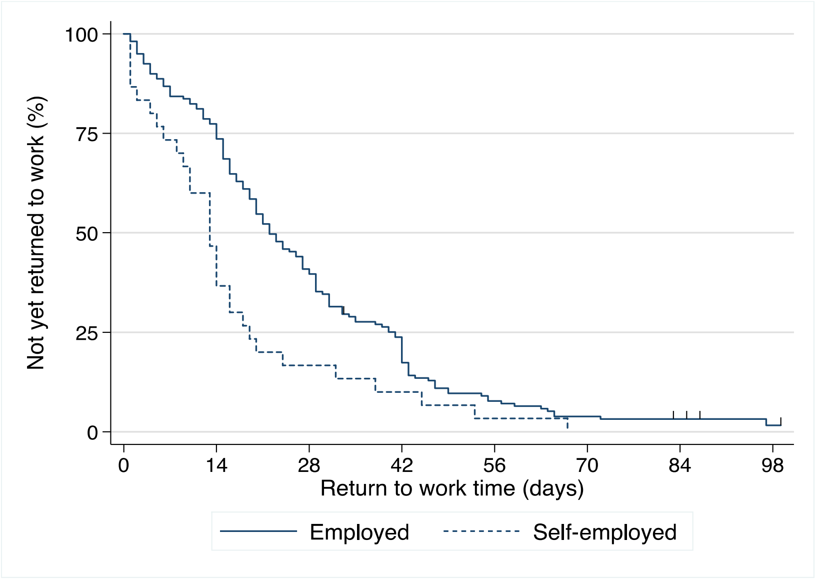

B. Employed versus self-employed workers

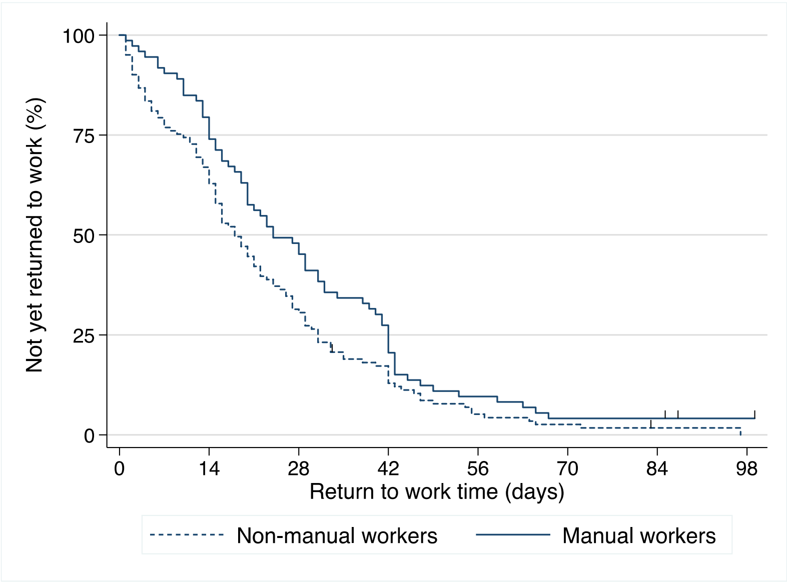

C. Non-manual versus manual workers

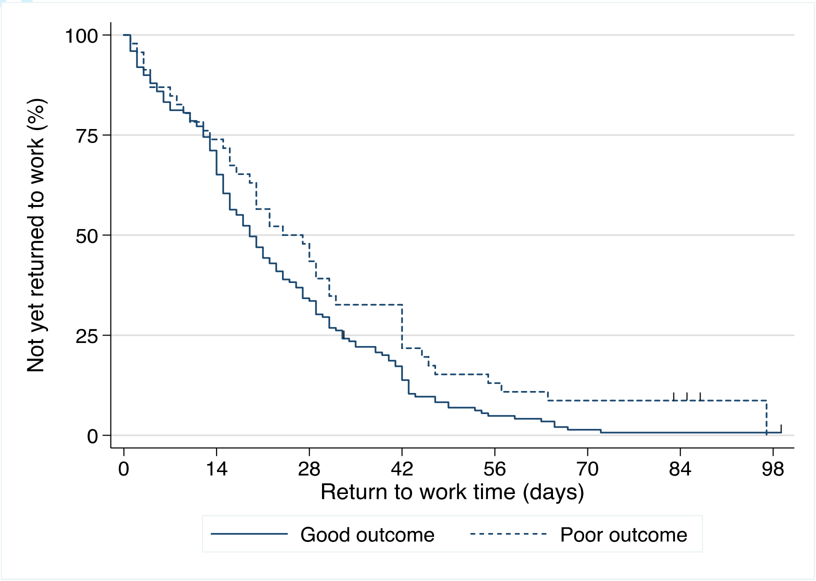

D. With versus without a poor outcome

Vertical lines indicate right censoring for those who had not returned to work at the point of last follow-up

Development of the REACTS study questionnaire: Reasoning for item inclusion

Questions were chosen to capture information on variables that might be expected to influence return to work after CTR.

Demographic factors

General demographic information was requested including date of birth, sex and hand dominance. Other studies have found that older age was associated with poorer work outcomes after CTR [1, 2], while no clear sex effect has been shown [3, 4]. Hand dominance in relation to side of surgery is rarely reported in CTR studies, however, surgery to the non-dominant hand has been linked to quicker and more complete resolution of CTS symptoms [5].

Carpal tunnel release planning

Information was collected on the expected date of CTR, side of surgery, availability of occupational health services and the patient's expectations about time off work post-surgery. Expected duration of work absence has been identified as a determinant of return to work time in previous CTR studies [2, 6].

Occupational factors

Participants were asked to list their main occupation and the industry in which they work (examples were provided to facilitate the response). This information was processed using the UK Office for National Statistics Standard Occupational Classification [7] and Computer Assisted Structured Coding tool (Cascot) [8] to generate manual and non-manual categories. Cases where the coding match was confirmed as less than 64% were reviewed by the lead researcher and coded by hand [8]. This was checked by the department data manager and any queries resolved through discussion.

Participants were asked to categorise their employment type as: employed (permanent contract), employed (temporary/renewable contract), zero hours contract and self-employed. Our systematic review found earlier return to work times for self-employed individuals compared to those who were employed [9]; but this was only investigated in two

For peer review only - http://bmjopen.bmj.com/site/about/guidelines.xhtml

studies [10, 11]. The additional sub-categories for employment type listed above and a separate question about sick-pay entitlement were included to allow the impact of work contract type to be explored [12]. Participants were also asked how many hours they usually worked each week and over how many days; this information was collected for the participant's main job and any other routine paid work. The total number of work hours per week was calculated by combining the hours for main and additional jobs.

Occupational activities that load the upper limb and potential work stressors were asked as a series of yes/no questions following the format of a recent multi-centre RCT exploring management of non-specific distal arm pain [13]. These questions originated in the Job Content Questionnaire, designed to assess psychological and physical aspects of work [14]. Activities included: computer use, tasks involving repeated wrist/finger movement, holding vibrating tools, lifting more than 5 or 10kgs, pushing/pulling a heavy weight, working with the neck flexed or rotated, and driving. Our systematic review found that manual workers took longer to return to work than non-manual workers [9] and these questions were used to determine the self-reported level of upper limb manual activity involved in each participant's job.

Potential psychosocial work stressors were also assessed. These included piecemeal work, activity targets and bonuses, and tight deadlines. As the first three items all concerned payment for results, these were combined for the analyses. Participants were also asked whether they found their main job demanding on their hands/wrist and whether their boss/colleagues were supportive. Both questions were scored on a 0-10 scale as reported previously in a study of sick leave duration after endoscopic CTR [15]. These were dichotomised as supportive (7-10) and neutral/unsupportive (0-6). A question about general job satisfaction was also included later in this section of the questionnaire, with the Likert response options: very satisfied, satisfied/fairly satisfied, dissatisfied and very dissatisfied [15, 16]. The last two options were condensed to give three categories for the analyses.

To assess self-reported work function, participants were asked to complete the work performance section of the Michigan Hand Questionnaire (MHQ) [17]. This patient reported outcome measure is frequently used in upper limb clinical practice and research and has

For peer review only - http://bmjopen.bmj.com/site/about/guidelines.xhtml

been validated for use with CTS and CTR populations [18]. Permission was granted for the MHQ to be used in the study. The questionnaire asked participants to recall how much difficulty they had with general work tasks over the past four weeks in relation to problems with their hands/wrists, for example: needing to shorten their working day, taking longer to complete tasks or needing to take breaks. Using the standard scoring, each question was completed on a Likert scale of: always, often, sometimes, rarely and never, and combined to give a score from 0-100, with 100 representing no problems with work functioning [19]. Participants were also asked whether they had taken any periods of sickness absence from work over the previous four weeks, both related to the hand/wrist problem, or for any other problem.

General health

Seven general health questions were included to capture information on comorbidities, physical and mental health and somatisation. Self-reported health was assessed using the first SF-36 question: In general, would you say your health is – excellent, very good, good, fair, poor [20]. This was taken from the original SF-36 version, which is free from licence charges and was dichotomised as excellent/very good/good and fair/poor for the analyses.

Participants were asked their height and weight to enable the calculation of BMI (body mass index; weight in kilograms/height in metres squared). This was categorised using standard WHO classification: underweight (BMI <18.5), normal weight (BMI 18.5-24.9), overweight (BMI 25.0-29.9) and obese (BMI ≥30.0) [21]. Smoking status was categorised as those who have never smoked regularly, those who have smoked in the past and those who regularly smoke, with the latter two categories combined for the analyses. Previous studies have found that obesity (BMI ≥30) was linked to poorer work outcomes [1] and smoking was linked to poorer clinical outcomes after CTR [22].

A list of common health problems and their impact on general activities was assessed using the Self-Administered Comorbidity Questionnaire [23]. Participants were asked first to select whether they have any of the 14 medical conditions and if so whether this limited their activities. All medical conditions were worded in an accessible format, as evaluated by the patient advisory group. Responses were analysed as the number of comorbidities and

For peer review only - http://bmjopen.bmj.com/site/about/guidelines.xhtml
For peer review only

the number of disabling comorbidities using the scale: 0, 1, ≥2. Mental health was assessed using the mental health and vitality questions from the licence-free version of the SF-36 [20]. One modification was made to change the wording of the question 'Did you feel full of pep?' to 'Did you feel full of get-up-and-go?' for the UK rather than US setting. The questions were used to calculate the summary score from 0-100 (where 100 represents no disability).

Somatisation was assessed using a subset of five questions from the Four-Dimensional Symptom Questionnaire [24] as previously reported in UK cohort studies of health and employment [25] and upper limb pain in primary care [26]. The number of symptoms that were rated by the participant as at least moderately distressing were used to create the analysis categories of: 0, 1, ≥2 symptoms.

Hand and wrist symptoms and function

Katz & Stirrat hand diagrams were included for the participants to indicate where on their hand(s) they experience pain and/or tingling and numbness [27]. This self-administered tool can be used clinically as part of the CTS diagnosis process using the scoring system modified by Calfee et al. [28]. A question on symptom duration was also included and categorised as less than 3 months, 3-6 months, 6-12 months and more than a year. This was dichotomised to ≤1 year and >1 year for analysis. All participants were expected to have clinically diagnosed CTS as they were undergoing CTR, but participants were asked to answer for both hands, so that symptoms in the non-operated hand were also assessed. This was used to define bilateral or unilateral symptoms. Hand diagram scores were dichotomised according to a stringent definition of CTS (classic and probable) and unlikely CTS (possible and unlikely).

The CTS-6 questionnaire was included to assess the severity of CTS symptoms [29]. This tool is a shorter version of the Boston Carpal Tunnel Questionnaire [30] and has been assessed for use pre- and post CTR surgery. The six questions explore the severity of pain and numbness, whether this occurs during the daytime or at night, and whether this wakes the individual. Participants were asked to complete these questions separately for each hand using a 5-point Likert scale. Using the standard scoring criteria, responses for each item

For peer review only - http://bmjopen.bmj.com/site/about/guidelines.xhtml

were combined to give a mean score ranging from 1-5, with 5 representing the highest severity of symptoms. If one response was missing (per hand) this was imputed using the mean score of the remaining responses [29].

Hand function was assessed using the MHQ sub-sections on unilateral hand function (asked for each hand), satisfaction with hand function (asked for each hand), and ability to perform unilateral and bilateral activities of daily living (ADLs) [17]. The MHQ summary question relating to the level of satisfaction with the appearance of each hand was also included. All questions were scored on the 5-point Likert scales provided and used the standard wording and scoring to enable comparison with other study populations. Possible scores for each sub-section range from 1-100, with 100 representing no problems or the highest level of satisfaction. Missing data were imputed according to the MHQ guidelines, which allow the scale to be calculated if more than 50% of the questions for each sub-section have been completed [19].

Health beliefs

The remaining questions related to health beliefs. Beliefs about the cause of symptoms and likely prognosis have been identified as key themes in health-seeking behaviour for CTS [31] and upper limb pain [26], and blaming oneself for the hand problem has been associated with long durations of sick leave after endoscopic CTR [15]. The participant's expectations for being able to use the affected hand normally within 3 months of surgery, fear of long-term hand problems, blaming oneself for the hand problem and the perceived level of support available from friends and family were assessed. Responses were rated on a 0-10 scale as reported by Hansen et al. [15]. All responses were converted to a unidirectional scale with 10 being the best outcome, and were dichotomised as neutral/negative response 0-6 and positive response 7-10.

Participants were also asked to agree/disagree (via a 5-point Likert scale) with a series of seven questions about the believed cause of their symptoms. Using previously reported methods, the responses were dichotomised to those who agreed (agree/strongly agree) and those who did not agree (neither agree nor disagree/disagree/disagree strongly) with each statement [26]. The first two questions were combined to generate six items: 1) I think I was

For peer review only - http://bmjopen.bmj.com/site/about/guidelines.xhtml

born with a weakness in this part of my body/problems like this run in my family; 2) my problem was caused by work; 3) my problem probably wasn't caused by work, but work made it worse; 4) I have a lot of stress in my life and that has made my problem a lot worse; 5) a lack of exercise probably contributed to my problem; 6) as you get older, parts of the body wear out and problems like mine are likely [26].

Finally, the abbreviated Pain Catastrophizing Scale was included, which provides insight about the participant's pain beliefs [32]. Responses were dichotomised to those who reported catastrophising pain thoughts and feelings to at least a moderate degree in response to all questions, and those who did not.

References

1. Parot-Schinkel E, Roquelaure Y, Ha C et al. Factors affecting return to work after carpal tunnel syndrome surgery in a large french cohort. *Arch of Phys Med Rehabil* 2011;92(11):1863-69.
2. Peters S, Johnston V, Hines S et al. Prognostic factors for return-to-work following surgery for carpal tunnel syndrome: a systematic review. *JBI Database of Systematic Reviews and Implementation Reports* 2016;14(9):135-216.
3. Bekkelund SI, Pierre-Jerome C, Torbergsen T et al. Impact of occupational variables in carpal tunnel syndrome. *Acta Neurol Scand* 2001;103(3):193-97.
4. Atroshi I, Zhou C, Joud A et al. Sickness absence from work among persons with new physician-diagnosed carpal tunnel syndrome: a population-based matched-cohort study. *PLoS One* 2015;10(3):e0119795.
5. Tang Q, Lai W, Tay S. The effect of hand dominance on patient-reported outcomes of carpal tunnel release in patients with bilateral carpal tunnel syndrome. *Journal of Hand Surgey Asian Pacific Volume* 2017;22(3):303-08.
6. Cowan J, Makanji H, Mudgal C et al. Determinants of return to work after carpal tunnel release. *J Hand Surg Am* 2012;37(1):18-27.
7. Office for National Statistics. Standard occupational classification for the UK: https://www.ons.gov.uk/methodology/classificationsandstandards/standardoccupationalclassificationsoc/soc2010. Accessed 08/06/2018.
8. Warwick Institute for Employment Research. Cascot: Computer assisted structured coding tool https://warwick.ac.uk/fac/soc/ier/software/cascot/. Accessed 15/05/2019.
9. Newington L, Stevens M, Warwick D et al. Sickness absence after carpal tunnel release: a systematic review. *Scandinavian Journal of Work, Environment and Health* 2018;44(6):557-67.
10. De Kesel R, Donceel P, De Smet L. Factors influencing return to work after surgical treatment for carpal tunnel syndrome. *Occup Med* 2008;58(3):187-90.

For peer review only - http://bmjopen.bmj.com/site/about/guidelines.xhtml

11. Chaise F, Bellemere P, Fril JP et al. Return-to-work interval and surgery for carpal tunnel syndrome. Results of a prospective series of 233 patients. *J Hand Surg Br* 2004;29(6):568-70.
12. Tran M, Sokas RK. The gig economy and contingent work: an occupational health assessment. *Journal of Occupational and Environmental Medicine* 2017;59(4):E63-E66.
13. Jones GT, Mertens K, Macfarlane GJ et al. Maintained physical activity and physiotherapy in the management of distal upper limb pain - a protocol for a randomised controlled trial (the arm pain trial). *BMC Musculoskelet Disord* 2014;15:71.
14. Karasek R, Brisson C, Kawakami N et al. The Job Content Questionnaire (JCQ): an instrument for internationally comparative assessments of psychosocial job characteristics. *Journal of occupational health psychology* 1998;3(4):322-55.
15. Hansen TB, Dalsgaard J, Meldgaard A et al. A prospective study of prognostic factors for duration of sick leave after endoscopic carpal tunnel release. *BMC Musculoskelet Disord* 2009;10:144.
16. D'Angelo S, Coggon D, Harris EC et al. Job dissatisfaction and the older worker: baseline findings from the Health and Employment After Fifty study. *Occupational and environmental medicine* 2016;73(8):512-19.
17. Chung KC, Pillsbury MS, Walters MR et al. Reliability and validity testing of the Michigan Hand Outcomes Questionnaire. *J Hand Surg Am* 1998;23(4):575-87.
18. Hadi M, Gibbons E, Fitzpatrick R. A structured review of patient-reported outcome measures for procedures for carpal tunnel syndrome. UK: Patient-Reported Outcome Measures Group, University of Oxford; 2011.
19. University of Michigan Medical School. The Michigan Hand Questionnaire: http://mhq.lab.medicine.umich.edu/. Accessed 04/04/2019.
20. Ware J, Sherbourne C. The MOS 36-item short-form health survey (SF-36): I. conceptual framework and item selection. *Medical Care* 1992;30:473-83
21. World Health Organization. Obesity: preventing and managing the global epidemic. Report of a WHO consultation. *World Health Organization technical report series* 2000;894:1-253.
22. Dahlin E, Zimmerman M, Bjorkman A et al. Impact of smoking and preoperative electrophysiology on outcome after open carpal tunnel release. *Journal of Plastic Surgery & Hand Surgery* 2017;51(5):329-35.
23. Sangha O, Stucki G, Liang MH et al. The self-administered comorbidity questionnaire: a new method to assess comorbidity for clinical and health services research *Arthritis & Rheumatism* 2003;49(2):156-63.
24. Terluin B, van Marwijk HW, Adèr HJ et al. The Four-Dimensional Symptom Questionnaire (4DSQ): a validation study of a multidimensional self-report questionnaire to assess distress, depression, anxiety and somatization. *BMC Psychiatry* 2006;6(1):34.
25. Palmer KT, Walker-Bone K, Harris EC et al. Health and Employment after Fifty (HEAF): a new prospective cohort study. *BMC public health* 2015;15:1071.
26. Palmer K, Calnan M, Wainwright D et al. Upper limb pain in primary care: health beliefs, somatic distress, consulting and patient satisfaction. *Family Practice* 2006;23(6):609-17.

For peer review only - http://bmjopen.bmj.com/site/about/guidelines.xhtml

27. Katz JN, Stirrat CR. A self-administered hand diagram for the diagnosis of carpal tunnel. *J Hand Surg Am* 1990;15:360-63.
28. Calfee RP, Dale AM, Ryan D et al. Performance of simplified scoring systems for hand diagrams in carpal tunnel syndrome screening. *J Hand Surg Am* 2012;37(1):10-17.
29. Atroshi I, Lyren PE, Ornstein E et al. The six-item CTS symptoms scale and palmar pain scale in carpal tunnel syndrome. *J Hand Surg Am* 2011;36(5):788-94.
30. Levine DW, Simmons BP, Koris MJ et al. A self-administered questionnaire for the assessment of severity of symptoms and functional status in carpal tunnel syndrome. *J Bone Joint Surg Am* 1993;75:1585-92.
31. Martin H. Patients' health beliefs and adaptation to carpal tunnel syndrome based on duration of symptomatic presentation. *J Hand Ther* 2007;20(1):29-35.
32. Bot AG, Becker SJ, Bruijnzeel H et al. Creation of the abbreviated measures of the Pain Catastrophizing Scale and the Short Health Anxiety Inventory: the PCS-4 and SHAI-5. *Journal of Musculoskeletal Pain* 2014;22(2):145-51.

For peer review only

For peer review only - http://bmjopen.bmj.com/site/about/guidelines.xhtml

Site | | | |

Please fill in today's date

d	d	m	m	y	y

Before Your Carpal Tunnel Release Surgery

REACTS

Return to Employment After Carpal Tunnel Release Surgery (REACTS)

ln@mrc.soton.ac.uk | 023 8077 7624
Arthritis Research UK – MRC Centre for Musculoskeletal Health and Work
MRC Lifecourse Epidemiology Unit, University of Southampton
Southampton General Hosptial (MP 95), SO16 6YD

IRAS reference: 209840

For peer review only

University Hospital Southampton NHS Foundation Trust REACTS Arthritis Research UK MRC centre for musculoskeletal health & work UNIVERSITY OF Southampton

CONSENT FORM (IRAS reference: 209840)

You should complete this form after you have read the Participant Information Sheet.

REACTS: Return to employment after carpal tunnel release surgery

Thank you for considering taking part in this research. If you have any questions arising from the Participant Information Sheet, please ask the research team before you decide whether to take part.

Please initial the boxes if you agree with each statement

1. I have read the Participant Information Sheet (version 2.0; 06.12.16) and have had the opportunity to ask questions about the study. ☐

2. I meet the criteria for being involved in this study: ☐
   - Aged over 18 and referred for carpal tunnel release surgery
   - Routinely work in paid employment for at least 20 hours per week
   - Plan to return to work after carpal tunnel release surgery
   - Have not previously had carpal tunnel release surgery on either hand
   - Have not previously had a serious injury to the same wrist/hand that will have the carpal tunnel release operation

3. I agree to take part in this research and agree for my data to be used for the purposes explained in the Participant Information Sheet (version 2.0; 06.12.16). I understand that this information will be handled in accordance with the terms of the UK Data Protection Act 1998. ☐
   a. I agree for the REACTS research team to access pre-operative test results concerning my hand and wrist symptoms. No other information will be accessed. ☐
   b. I agree for the REACTS research team to access my carpal tunnel release surgical record. No other information will be accessed. ☐

4. I understand that if I decide at any time during the research that I no longer wish to take part, I can notify the researchers and withdraw from the study immediately, without giving a reason. If I do, I understand that I can ask for any contribution I have already made to be removed from the study, up to the time when I have completed the final questionnaire. ☐

Signature ________________________________ Date ______ / ______ / ______

Name ________________________________ Phone ________________
(please print) *(only to be used if we lose touch)*

Postal address ____

____

Email address ____
(please print)

For peer review only - http://bmjopen.bmj.com/site/about/guidelines.xhtml

University Hospital Southampton NHS Foundation Trust | REACTS | Arthritis Research UK / MRC centre for musculoskeletal health & work | University of Southampton

ADDITIONAL QUESTIONS

Please circle one response for each question

I prefer to receive the next two questionnaires by	**Post**	**Email**	**Don't mind**
I prefer to receive correspondence about the study by	**Post**	**Email**	**Don't mind**
I would like to be notified of the findings from this research	**Yes**	**No**	
I am happy to be contacted about the next stage of the research, which will involve a one-off discussion with the lead researcher	**Yes**	**No**	
I am happy to be contacted about other studies related to this research	**Yes**	**No**	

When the research team receives your completed questionnaire and consent form, we will sign it below and return a copy to you for your records.

Researcher signature ______________________________________ Date ____ / ____ / ____

Researcher name ______________________________________

University of Southampton research supervisors:
Professor Karen Walker-Bone | Professor Jo Adams | Professor David Warwick

For peer review only

SECTION A: BACKGROUND

1 What is your date of birth?

d	d	m	m	y	y

2 Are you:

Male ☐ Female ☐ Other ☐

3 Are you:

Right handed ☐ Left handed ☐ Both ☐

4 Do you routinely carry out paid work for 20 hours or longer in a given week?

Yes ☐ No ☐

If no, *thank you for your interest in our study, however, we are only looking for individuals who carry out paid work for at least 20 hours per week. You* ***do not*** *need to complete the rest of the questionnaire, but please return it using the pre-paid envelope provided.*

5 When do you expect to have your carpal tunnel surgery?
Please enter the exact date if known, or provide the approximate month and year if unsure.

d	d	m	m	y	y

6 Which hand will be operated on?
If both hands please answer Question 6.1; if one hand, please move on to Question 7.

Right ☐ Left ☐ Both ☐

6.1 If both hands, which side will be operated on first?

Right ☐ Left ☐ Both sides operated on the same day ☐ Unsure ☐

7 Do you have access to an occupational health service through your place of work?

Yes ☐ No ☐ Unsure ☐

8 Do you expect to take any time off work following your surgery?
If yes, please answer Question 8.1; if no, please move on to Question 9.

Yes ☐ No ☐ Unsure ☐

8.1 If you do expect to take time off work, how long do you expect to take?
Please complete using days, weeks or months; whichever applies.

Days ☐ Weeks ☐ Months ☐

For peer review only

For peer review only - http://bmjopen.bmj.com/site/about/guidelines.xhtml

SECTION A: BACKGROUND

9 Have you been given any information about your operation?
If yes, please answer Question 9.1; if no, please move on to Question 10.

Yes ☐ No ☐

9.1 If yes, who provided this information? Please tick all that apply.

a) Your surgeon or a member of the surgical team ☐
b) Hospital nurse ☐
c) GP or practice nurse ☐
d) Hand therapist ☐
e) Physiotherapist or occupational therapist ☐
f) Occupational health nurse or doctor ☐
g) Employer ☐
h) Friend or family member ☐
i) Internet ☐
j) Other *(please specify)* ☐

....

10 Have you been given any information about returning to work after your surgery?
If yes, please answer the rest of Question 10; if no, please move on to Question 11.

Yes ☐ No ☐

10.1 If yes, who provided this information? Please tick all that apply.

a) Your surgeon or a member of the surgical team ☐
b) Hospital nurse ☐
c) GP or practice nurse ☐
d) Hand therapist ☐
e) Physiotherapist or occupational therapist ☐
f) Occupational health nurse or doctor ☐
g) Employer ☐
h) Friend or family member ☐
i) Internet ☐
j) Other *(please specify)* ☐

....

10.2 What advice were you given?
If this advice came from more than one source, please indicate who advised what.

....
....
....
....
....
....
....

For peer review only

SECTION B: WORK

12 What is your MAIN occupation at the moment (e.g. secretary, teacher, builder etc.)?

----

13 And in what industry do you work (e.g. farming, shipyard, car factory, shoe shop, hospital, insurance office etc)?

----

14 Which of the following best describes your present work situation for your MAIN occupation? Please tick one box.

a) Employed (permanent contract) ☐

b) Employed (temporary/renewable contract) ☐

c) Zero hours contract ☐

d) Self-employed ☐

e) Other *(please specify)* ☐

----

15 On average, how many *hours* per week do you normally work in your main occupation?

☐☐☐ hours

16 On average, how many *days* per week do you normally work in your main occupation?

☐ days

17 Do you have any other paid work?
If yes, please answer Question 17.1; if no, please move on to Question 18.

Yes ☐ No ☐

17.1 If yes, on average, how many hours a week do you work in other paid jobs? ☐☐ hours

18 Does an average day at work in your MAIN job normally involve any of the following? Please tick one box for each question.

		**Yes**	**No**
a)	Piecework in which you are paid according to the number of articles or tasks you or your team make or finish in the day?	☐	☐
b)	A target number of articles or tasks that you or your team are expected to make or finish in the day?	☐	☐
c)	Payment of a bonus if you make or finish more than an agreed number of articles/tasks in the day?	☐	☐
d)	Working to tight deadlines	☐	☐
e)	Use of a computer keyboard or mouse for longer than 1 hour in total?	☐	☐

For peer review only - http://bmjopen.bmj.com/site/about/guidelines.xhtml

SECTION B: WORK

		Yes	No
f)	Use of a computer keyboard or mouse for longer than 4 hours in total?	☐	☐
g)	Other tasks involving repeated movements of the wrist or fingers for longer than 4 hours in total? *(Please indicate which tasks)*	☐	☐

		Yes	No
h)	Working with a powered tool that makes your hand(s) or arm(s) vibrate (e.g. chain saw, pneumatic drill)?	☐	☐
i)	Working with your hand(s) above shoulder height for longer than 1 hour in total?	☐	☐
j)	Lifting or carrying weights of 5 kg (11 lbs) or more in one hand (e.g. a tool bag or heavy briefcase)?	☐	☐
k)	Lifting or carrying a weight of 10 kg (22 lbs) or more?	☐	☐
l)	Tasks involving pushing or pulling a heavy weight?	☐	☐
m)	Working for longer than two hours in total with your neck bent forward?	☐	☐
n)	Working for longer than half an hour in total with your neck twisted e.g. when looking to one side?	☐	☐
o)	Driving for more than an hour?	☐	☐

19 Do you find your MAIN job demanding on your hands/wrists?
Please circle one number, where 0 represents not at all, and 10 represents very much.

0 1 2 3 4 5 6 7 8 9 10

Not at all Very much

20 Does your MAIN employer (or boss/colleagues if self-employed) know about your hand/wrist problem?
If yes, please answer Question 20.1; if no, or not applicable, please move on to Question 21.

Yes ☐ No ☐ N/A self-employed and work alone ☐

20.1 Is your MAIN employer (or boss/colleagues if self-employed) supportive of your hand/wrist problem?
Please circle one number, where 0 represents not at all, and 10 represents very much

0 1 2 3 4 5 6 7 8 9 10

Not at all Very much

For peer review only - http://bmjopen.bmj.com/site/about/guidelines.xhtml

SECTION B: WORK

21 **The following questions refer to how you did in your MAIN job during the *past 4 weeks*.**
Please tick one box for each question.

How much of the time during the past 4 weeks …	**Always**	**Often**	**Sometimes**	**Rarely**	**Never**
a) Were you unable to do your work because of problems with your hand(s) / wrist(s)?	☐	☐	☐	☐	☐
b) Did you have to shorten your work day because of problems with your hand(s) / wrists(s)	☐	☐	☐	☐	☐
c) Did you have to take breaks at work because of problems with your hand(s) / wrists(s)?	☐	☐	☐	☐	☐
d) Did you get less done because of problems with your hand(s) / wrist(s)?	☐	☐	☐	☐	☐
e) Did you take longer to do the tasks in your work because of problems with your hand(s) / wrists(s)?	☐	☐	☐	☐	☐

22 **During the *past 4 weeks*, how much time have you missed from your MAIN job for the following reasons?**
Please write 0 if you have not missed any time from work during this period. You can answer in days or hours, whichever applies.

a) Time missed because of the problem with your hand(s)/wrist(s) ☐☐ Days ***or*** ☐☐ Hours

b) Time missed because of any other problem ☐☐ Days ***or*** ☐☐ Hours

23 **If you fell ill and were off work, how long could you get your normal full pay (excluding bonuses)?**
Please tick the option that best represents your MAIN job.

a) Less than one week ☐
b) 1 – 4 weeks ☐
c) 1 – 6 months ☐
d) More than 6 months ☐
e) Not sure ☐

24 **How satisfied are you with your MAIN job as a whole, taking everything into consideration?** This includes your salary, career possibilities, management, colleagues etc. Please tick one box.

a) Very satisfied ☐
b) Satisfied/fairly satisfied ☐
c) Dissatisfied ☐
d) Very dissatisfied ☐

For peer review only - http://bmjopen.bmj.com/site/about/guidelines.xhtml

SECTION C: GENERAL HEALTH

25 In general, would you say your health is:

a) Excellent ☐
b) Very good ☐
c) Good ☐
d) Fair ☐
e) Poor ☐

26 What is your height? Please answer in either feet and inches or centimetres.

☐ feet ☐☐ inches *or* ☐☐☐ cms

27 What is your weight? Please answer in either stones and pounds or kilograms.

☐☐ stones ☐☐ lbs *or* ☐☐ kgs

28 Do you, or have you ever, smoked regularly? Please tick one box.

a) I have never smoked regularly ☐
b) I have smoked in the past, but do not currently smoke regularly ☐
c) I regularly smoke ☐

29 The following is a list of common health problems. Please indicate if you currently have, or don't have, the problem listed in part 1. If you have the problem, please answer the corresponding question in part 2.

Please answer all questions in part 1.

	HEALTH PROBLEM	PART 1 Do you have the problem? NO	YES *(if yes move to part 2)* →	PART 2 Does it limit your activities? NO	YES
a)	Heart disease	☐	☐	☐	☐
b)	High blood pressure	☐	☐	☐	☐
c)	Lung disease	☐	☐	☐	☐
d)	Diabetes	☐	☐	☐	☐
e)	Ulcer or stomach disease	☐	☐	☐	☐
f)	Kidney disease	☐	☐	☐	☐
g)	Liver disease	☐	☐	☐	☐
h)	Thyroid disease	☐	☐	☐	☐
i)	Anaemia or other blood disease	☐	☐	☐	☐
j)	Cancer	☐	☐	☐	☐

For peer review only

SECTION C: GENERAL HEALTH

HEALTH PROBLEM continued…	Do you have the problem? NO	YES (if yes move to part 2)	Does it limit your activities? NO	YES
k) Depression	☐	☐	☐	☐
l) Osteoarthritis	☐	☐	☐	☐
m) Back pain	☐	☐	☐	☐
n) Rheumatoid arthritis	☐	☐	☐	☐

29.1 Please list any other medical problems that have not been mentioned.

	Does it limit your activities? NO	YES
o)	☐	☐
p)	☐	☐
q)	☐	☐

30 The following questions are about how you feel and how things have been with you during the *past 4 weeks*. For each question, please give the answer that comes closest to the way you have been feeling. Please tick one box for each row.

How much of the time during the past 4 weeks …	All of the time	Most of the time	A good bit of the time	Some of the time	A little bit of the time	None of the time
a) Did you feel full of 'get-up-and-go'?	☐	☐	☐	☐	☐	☐
b) Have you been a very nervous person?	☐	☐	☐	☐	☐	☐
c) Have you felt so down in the dumps that nothing could cheer you up?	☐	☐	☐	☐	☐	☐
d) Have you felt calm and peaceful?	☐	☐	☐	☐	☐	☐
e) Did you have a lot of energy?	☐	☐	☐	☐	☐	☐
f) Have you felt downhearted and blue?	☐	☐	☐	☐	☐	☐
g) Did you feel worn out?	☐	☐	☐	☐	☐	☐
h) Have you been a happy person?	☐	☐	☐	☐	☐	☐
i) Did you feel tired?	☐	☐	☐	☐	☐	☐

For peer review only - http://bmjopen.bmj.com/site/about/guidelines.xhtml

SECTION C: GENERAL HEALTH

31 **Below is a list of problems that people sometimes have. Please read each one carefully and tick the box that best describes how much that problem has distressed or bothered you during the *past 7 days*, including today?**
Please tick one box for each row.

		Not at all	A little bit	Moderately	Quite a bit	Extremely
a)	Faintness or dizziness	☐	☐	☐	☐	☐
b)	Pains in the heart or chest	☐	☐	☐	☐	☐
c)	Nausea or upset stomach	☐	☐	☐	☐	☐
d)	Trouble getting your breath	☐	☐	☐	☐	☐
e)	Hot or cold spells	☐	☐	☐	☐	☐

For peer review only

For peer review only - http://bmjopen.bmj.com/site/about/guidelines.xhtml

SECTION D: HAND AND WRIST FUNCTION

32 **In the *past 7 days*, have you experienced any pain, tingling (pins and needles) or numbness (loss of sensation) in your RIGHT hand or wrist?**
Please mark where on your hand/wrist you experienced these symptoms using the key below.
If you do not have any symptoms in your right hand, please move on to Question 34.

Pain ☐ Tingling or numbness ☐

RIGHT HAND

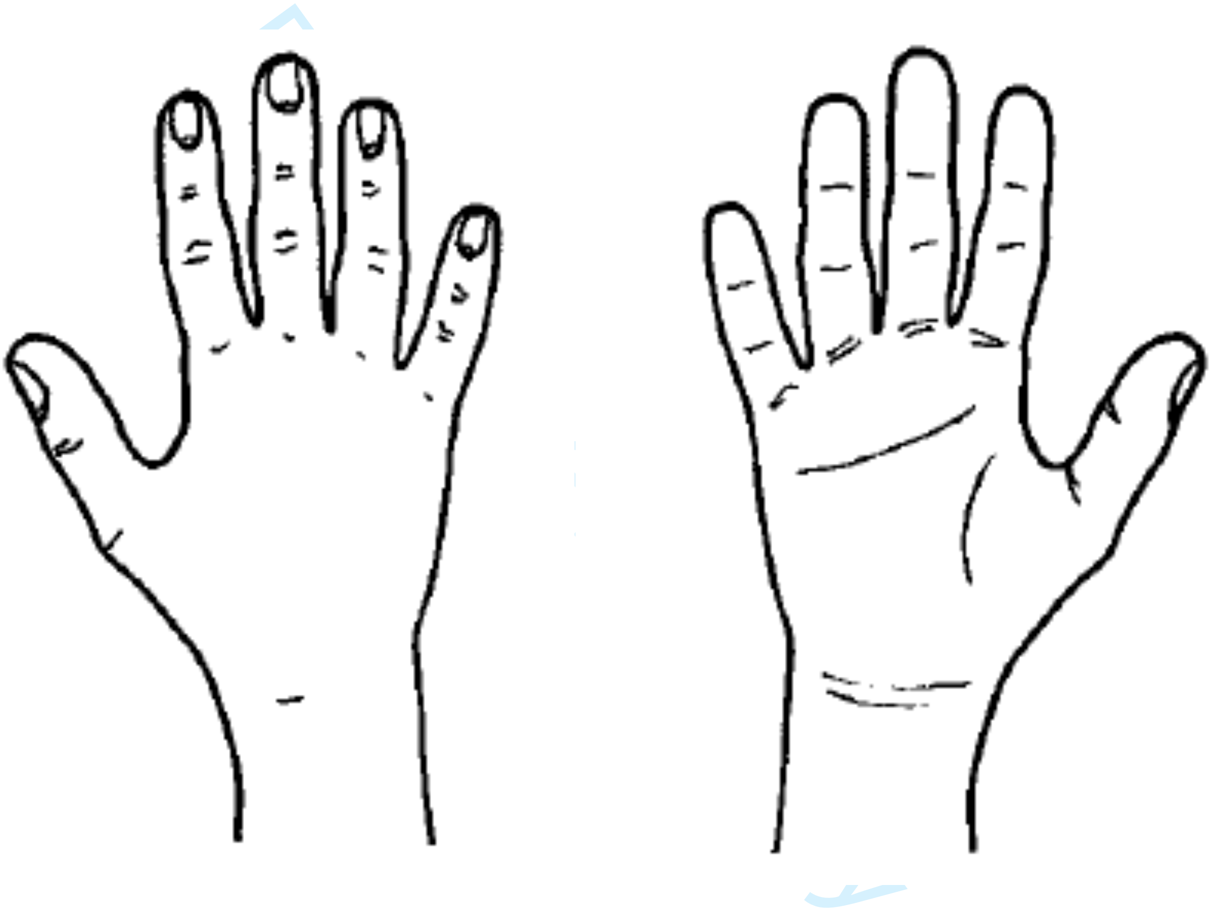

33 How long ago did the first of these symptoms begin? Please tick one box.

a) Less than 3 months ☐
c) 6 – 12 months ☐
b) 3 – 6 months ☐
d) More than a year ☐

For peer review only - http://bmjopen.bmj.com/site/about/guidelines.xhtml

SECTION D: HAND AND WRIST FUNCTION

34 **In the *past 7 days*, have you experienced any pain, tingling (pins and needles) or numbness (loss of sensation) in your LEFT hand or wrist?**
Please mark where on your hand/wrist you experienced these symptoms using the key below.
If you do not have any symptoms in your right hand, please move on to Question 36.

Pain Tingling or numbness

LEFT HAND

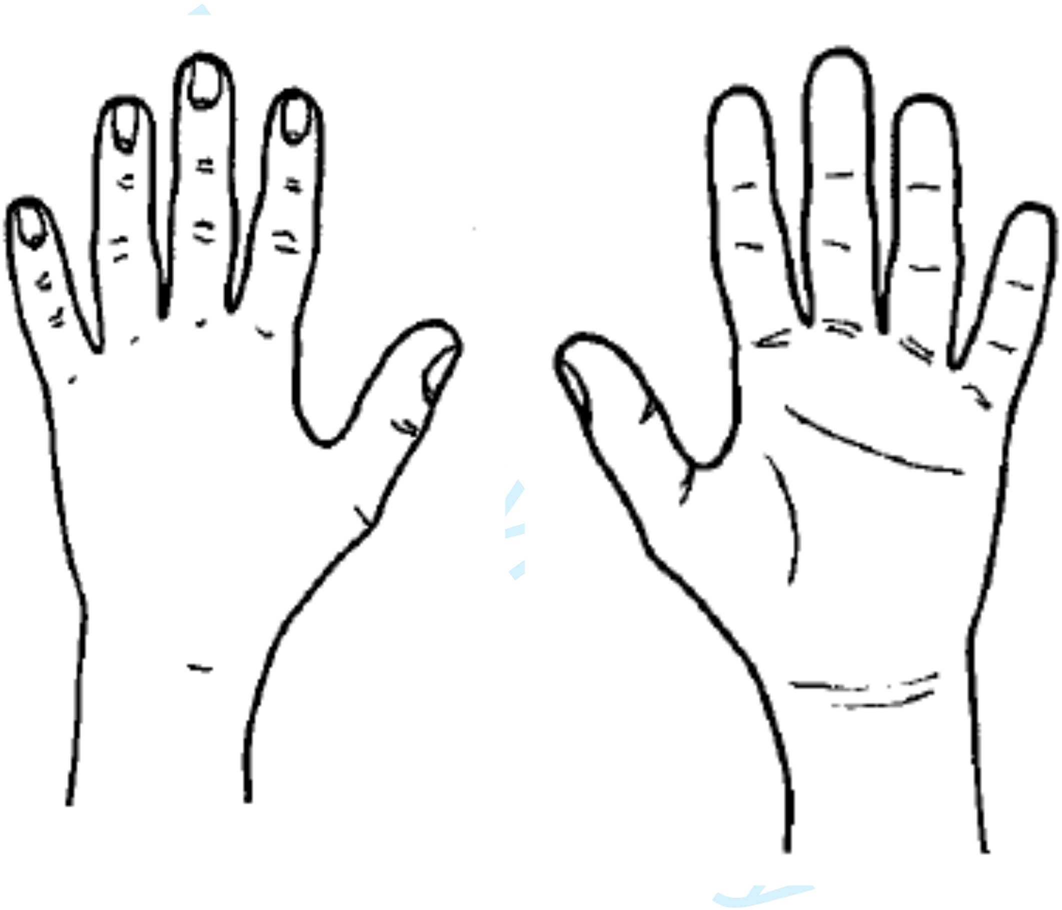

35 How long ago did the first of these symptoms begin? Please tick one box.

a) Less than 3 months	☐	c) 6 – 12 months	☐
b) 3 – 6 months	☐	d) More than a year	☐

For peer review only - http://bmjopen.bmj.com/site/about/guidelines.xhtml

SECTION D: HAND AND WRIST FUNCTION

36 **The following questions refer to your symptoms over the *last 7 days*.**
Please answer for each hand, even if you only have problems with one side.
Please tick one box for each row.

**36.1 How severe were the following symptoms in your RIGHT hand?**	**None**	**Mild**	**Moderate**	**Severe**	**Very severe**
a) Pain at night	☐	☐	☐	☐	☐
b) Pain during the daytime	☐	☐	☐	☐	☐
c) Numbness or tingling at night	☐	☐	☐	☐	☐
d) Numbness or tingling during the daytime	☐	☐	☐	☐	☐

**How often did the following symptoms in your RIGHT hand wake you up at night?**	**Never**	**Once**	**2 or 3 times**	**4 or 5 times**	**More than 5 times**
e) Pain	☐	☐	☐	☐	☐
f) Numbness or tingling	☐	☐	☐	☐	☐

**36.2 How severe were the following symptoms in your LEFT hand?**	**None**	**Mild**	**Moderate**	**Severe**	**Very severe**
a) Pain at night	☐	☐	☐	☐	☐
b) Pain during the daytime	☐	☐	☐	☐	☐
c) Numbness or tingling at night	☐	☐	☐	☐	☐
d) Numbness or tingling during the daytime	☐	☐	☐	☐	☐

**How often did the following symptoms in your LEFT hand wake you up at night?**	**Never**	**Once**	**2 or 3 times**	**4 or 5 times**	**More than 5 times**
e) Pain	☐	☐	☐	☐	☐
f) Numbness or tingling	☐	☐	☐	☐	☐

37 **This question refers to the appearance (look) of your hand during the *past 7 days*.** Please tick one box for each hand.

	**Strongly agree**	**Agree**	**Neither agree nor disagree**	**Disagree**	**Strongly disagree**
a) I am satisfied with the appearance (look) of my **RIGHT** hand	☐	☐	☐	☐	☐
b) I am satisfied with the appearance (look) of my **LEFT** hand	☐	☐	☐	☐	☐

For peer review only

SECTION D: HAND AND WRIST FUNCTION

38 Please answer the following questions on a scale of 0-10, where 0 represents not at all, and 10 represents very much. Please circle one number for each question.

38.1 Do you think that you will be able to use your hand normally 3 months after the operation?

0 1 2 3 4 5 6 7 8 9 10

38.2 Are you afraid of having long-term problems with your hand?

0 1 2 3 4 5 6 7 8 9 10

38.3 Do you blame yourself for your hand problem?

0 1 2 3 4 5 6 7 8 9 10

38.4 Are your family and friends supportive of your hand problem?

0 1 2 3 4 5 6 7 8 9 10

39 The following statements describe people's beliefs about their health problems. Please indicate whether you agree or disagree with them in relation to the problems you have with your hand(s) or wrist(s).
Please tick the box which most closely reflects how you feel for each statement.

	Strongly agree	Agree	Neither agree or disagree	Disagree	Strongly Disagree
a) Problems like this run in my family	☐	☐	☐	☐	☐
b) I think I was born with a weakness or underlying problem in this part of my body	☐	☐	☐	☐	☐
c) My problem was caused by work	☐	☐	☐	☐	☐
d) Work probably didn't cause my problem, but it made it worse	☐	☐	☐	☐	☐
e) I have a lot of stress in my life and that has made my problem a lot worse	☐	☐	☐	☐	☐
f) I think a lack of exercise probably contributed to my problem	☐	☐	☐	☐	☐
g) As you get older, parts of the body start to wear out and problems like mine are likely to occur	☐	☐	☐	☐	☐

For peer review only - http://bmjopen.bmj.com/site/about/guidelines.xhtml

SECTION D: HAND AND WRIST FUNCTION

40 We are interested in the types of thoughts and feelings that you have when you are in pain. The following statements describe different thoughts and feelings that may be associated with pain. Please indicate the degree to which you have these thoughts and feelings when you are experiencing pain.
Please tick one box for each statement.

	Not at all	To a slight degree	To a moderate degree	To a great degree	All of the time
a) I keep thinking about how badly I want the pain to stop	☐	☐	☐	☐	☐
b) It's terrible and I think it's never going to get any better	☐	☐	☐	☐	☐
c) I become afraid that the pain may get worse	☐	☐	☐	☐	☐
d) I anxiously want the pain to go away	☐	☐	☐	☐	☐

The following questions refer to the function of your hands/wrists during the ***past 7 days***. Please answer all questions for the right and left sides, even if you do not experience any problems. Please tick one box for each question.

**41 RIGHT SIDE**	Very well	Well	Adequately	Poorly	Very poorly
a) Overall, how well did your ***right*** hand work?	☐	☐	☐	☐	☐
b) How well did your ***right*** fingers move?	☐	☐	☐	☐	☐
c) How well did your ***right*** wrist move?	☐	☐	☐	☐	☐
	**Very good**	**Good**	**Fair**	**Poor**	**Very poor**
d) How was the strength in your ***right*** hand?	☐	☐	☐	☐	☐
e) How was the sensation (feeling) in your ***right*** hand?	☐	☐	☐	☐	☐

**42 LEFT SIDE**	Very well	Well	Adequately	Poorly	Very poorly
a) Overall, how well did your ***left*** hand work?	☐	☐	☐	☐	☐
b) How well did your ***left*** fingers move?	☐	☐	☐	☐	☐
c) How well did your ***left*** wrist move?	☐	☐	☐	☐	☐
	**Very good**	**Good**	**Fair**	**Poor**	**Very poor**
d) How was the strength in your ***left*** hand?	☐	☐	☐	☐	☐
e) How was the sensation (feeling) in your ***left*** hand?	☐	☐	☐	☐	☐

For peer review only

For peer review only - http://bmjopen.bmj.com/site/about/guidelines.xhtml

SECTION D: HAND AND WRIST FUNCTION

The following questions refer to the ability of your hands to do certain tasks during the ***past 7 days***. If you do not do a certain task, please estimate the difficulty you would have in performing it. Please tick one box for every activity.

43 How difficult was it for you to perform the following activities using your RIGHT HAND?

	Not at all difficult	A little difficult	Somewhat difficult	Moderately difficult	Very difficult
a) Turn a door knob	☐	☐	☐	☐	☐
b) Pick up a coin	☐	☐	☐	☐	☐
c) Hold a glass of water	☐	☐	☐	☐	☐
d) Turn a key in a lock	☐	☐	☐	☐	☐
e) Hold a frying pan	☐	☐	☐	☐	☐

44 How difficult was it for you to perform the following activities using your LEFT HAND?

	Not at all difficult	A little difficult	Somewhat difficult	Moderately difficult	Very difficult
a) Turn a door knob	☐	☐	☐	☐	☐
b) Pick up a coin	☐	☐	☐	☐	☐
c) Hold a glass of water	☐	☐	☐	☐	☐
d) Turn a key in a lock	☐	☐	☐	☐	☐
e) Hold a frying pan	☐	☐	☐	☐	☐

45 How difficult was it for you to perform the following activities using BOTH HANDS?

	Not at all difficult	A little difficult	Somewhat difficult	Moderately difficult	Very difficult
a) Open a jar	☐	☐	☐	☐	☐
b) Button a shirt/blouse	☐	☐	☐	☐	☐
c) Eat with a knife/fork	☐	☐	☐	☐	☐
d) Carry a grocery bag	☐	☐	☐	☐	☐
e) Wash dishes	☐	☐	☐	☐	☐
f) Wash your hair	☐	☐	☐	☐	☐
g) Tie shoelaces/knots	☐	☐	☐	☐	☐

For peer review only

SECTION D: HAND AND WRIST FUNCTION

The following questions refer to your satisfaction with your hands/wrists during the ***past 7 days***. Please tick one box for each question

46 How satisfied were you with your RIGHT hand/wrist during the *past 7 days*?

	RIGHT HAND	Very satisfied	Somewhat satisfied	Neither satisfied or dissatisfied	Somewhat dissatisfied	Very dissatisfied
a)	Overall function of your hand	☐	☐	☐	☐	☐
b)	Movement of the fingers	☐	☐	☐	☐	☐
c)	Movement of your wrist	☐	☐	☐	☐	☐
d)	Strength of your hand	☐	☐	☐	☐	☐
e)	Pain level of your hand	☐	☐	☐	☐	☐
f)	Sensation (feeling) of your hand	☐	☐	☐	☐	☐

47 How satisfied were you with your LEFT hand/wrist during the *past 7 days*?

	LEFT HAND	Very satisfied	Somewhat satisfied	Neither satisfied or dissatisfied	Somewhat dissatisfied	Very dissatisfied
a)	Overall function of your hand	☐	☐	☐	☐	☐
b)	Movement of the fingers	☐	☐	☐	☐	☐
c)	Movement of your wrist	☐	☐	☐	☐	☐
d)	Strength of your hand	☐	☐	☐	☐	☐
e)	Pain level of your hand	☐	☐	☐	☐	☐
f)	Sensation (feeling) of your hand	☐	☐	☐	☐	☐

Thank you for completing this questionnaire!
Please return it to the REACTS team
using the pre-paid envelope.

REACTS

If you have any questions, or would like any additional information, please contact Lisa Newington on:
ln@mrc.soton.ac.uk | 023 8077 7624 | 07866 997732

For peer review only - http://bmjopen.bmj.com/site/about/guidelines.xhtml

REACTS ID: | | | | | |

One Month After Your Carpal Tunnel Release Surgery

Return to Employment After Carpal Tunnel Release Surgery (REACTS)

ln@mrc.soton.ac.uk | 023 8077 7624
Arthritis Research UK – MRC Centre for Musculoskeletal Health and Work
MRC Lifecourse Epidemiology Unit, University of Southampton
Southampton General Hosptial (MP 95), SO16 6YD

IRAS reference: 209840

For peer review only - http://bmjopen.bmj.com/site/about/guidelines.xhtml

SECTION A: ABOUT YOUR OPERATION

Please fill in today's date ☐☐☐☐☐☐☐☐
d d m m y y y y

1 What was the date of your carpal tunnel release surgery? ☐☐☐☐☐☐☐☐
d d m m y y y y

2 Which side was operated on? Please tick one box.

Right ☐ Left ☐ Both ☐

3 What type of anaesthetic did you have? Please tick one box.

a) General anaesthetic (you were sent to sleep) ☐

b) Local or regional anaesthetic (your arm was made numb, but you were still awake) ☐

c) Other *(please specify)* ☐

----

d) Unsure ☐

4 How long did you need to stay in the hospital/clinic after your operation?
Please tick one box (and specify the number of nights, if applicable).

a) I went home the same day ☐

b) I needed to stay overnight (one night only) ☐

c) I needed to stay for more than one night ☐

(Please specify for how long) ☐☐ nights

For peer review only

SECTION A: ABOUT YOUR OPERATION

5 Have you used any of the following services specifically for your operated hand(s) since your surgery?
Please give the number of visits for each service, and the date(s) attended, if known.

	I used this service in the NHS		I used this service privately	
	Number of visits	Dates attended, if known	Number of visits	Dates attended, if known
a) Your surgeon, or one of the surgical team				
b) GP or practice nurse				
c) Hospital nurse				
d) Pharmacist				
e) Hand therapist				
f) Other physiotherapist or occupational therapist				
g) Chiropractor or osteopath				
h) Occupational health nurse or doctor				
i) Accident and emergency (A&E) or minor injuries unit				
j) Other (*please specify*)				

For peer review only - http://bmjopen.bmj.com/site/about/guidelines.xhtml

SECTION A: ABOUT YOUR OPERATION

6 Have you taken any antibiotics for an infection in your surgical wound?
Please do not include any antibiotics you were prescribed at the time of your operation.

Yes ☐ No ☐

If yes, what date did you start taking the antibiotics? ☐☐☐☐☐☐☐☐ d d m m y y y y

7 Have you been admitted to hospital because of a problem with your operated hand(s)? If yes, please answer the rest of Question 7; if no, please move on to Question 8.

Yes ☐ No ☐

7.1 If yes, when were you first admitted? ☐☐☐☐☐☐☐☐ d d m m y y y y

7.2 How many nights did you stay in hospital?
Please answer 0 if you didn't stay overnight.
☐☐ nights

7.3 Did you require another operation? Yes ☐ No ☐

8 Have you been advised that you may need a carpal tunnel release for your other hand in the future?
If yes, please answer Question 8.1; if no, please move on to Question 9.

Yes ☐ No ☐

8.1 If yes, when are you expecting to have this surgery? Please tick one box.

a) In less than 2 months ☐
b) In 2-5 months ☐
c) In 6-11 months ☐
d) In more than a year ☐
e) Unsure ☐

9 If you would like to give us any other information about your operation, or the healthcare services you have used, please do so here:

For peer review only - http://bmjopen.bmj.com/site/about/guidelines.xhtml

SECTION B: WORK

10 Compared to before your surgery, which of the following best describes your current work situation? Please tick one box.

a) Returned to the same job, work duties and hours – **please go to Question 14** ☐

b) Returned to the same job, with altered duties or hours – **please go to Question 14** ☐

c) Started a new job – **please go to Question 11** ☐

d) Not yet returned to work, but plan to return in the future – **please go to Question 12** ☐

e) Do not plan to return to work – **please go to Question 13** ☐

11 Thinking about your new job:

11.1 What is your main occupation now (e.g. secretary, teacher, builder etc.)?

11.2 In what industry do you work (e.g. farming, shipyard, car factory, shoe shop, hospital, insurance office etc.)?

11.3 Did you change jobs because of your hand/wrist problem? Please tick one box.

a) Yes, my hand/wrist problem was the main reason for my job change ☐

b) Yes, my hand/wrist problem was one of several reasons for my job change ☐

c) No, my job change was nothing to do with my hand/wrist problem ☐

d) Other, please specify ☐

Please go to Question 14

For peer review only - http://bmjopen.bmj.com/site/about/guidelines.xhtml

SECTION B: WORK

12 If you have not yet returned to work, when do you think you might be able to return? Please give an estimated date if you are unsure.

d	d	m	m	y	y	y	y

12.1 Have you discussed when to return to work with anyone?
If yes, please answer the rest of Question 12; if no, please move on to Question 21.

Yes ☐ No ☐

12.2 If yes, who have you discussed this with? Please tick all that apply.

a) Your surgeon or a member of the surgical team ☐
b) Hospital nurse ☐
c) GP or practice nurse ☐
d) Hand therapist ☐
e) Physiotherapist or occupational therapist ☐
f) Occupational health nurse or doctor ☐
g) Employer or manager (or colleagues if self-employed ☐
h) Friend or family member ☐
i) Other *(please specify)* ☐

12.3 Since your operation, have you been given any specific advice about when and how to return to work? This could include any activities to avoid or timescales to follow. Please list any advice here, including who gave you this advice:

Please go to Question 21

For peer review only - http://bmjopen.bmj.com/site/about/guidelines.xhtml

For peer review only

SECTION B: WORK

13 If you do not plan to return to work, what is the main reason for this decision?
Please tick one box.

a) Retirement ☐
b) Redundancy ☐
c) Position/work no longer available ☐
d) Unable to do your work because of your problem with your hand(s)/wrist(s) ☐
e) Unable to do your work because of any other problem ☐
f) Other *(please specify)* ☐

....

....

....

....

13.1 Have you been advised not to return to work by anyone?
If yes, please answer Question 13.2; if no, please move on to Question 21.

Yes ☐ No ☐

13.2 If yes, who by? Please tick all that apply.

a) Your surgeon or a member of the surgical team ☐
b) Hospital nurse ☐
c) GP or practice nurse ☐
d) Hand therapist ☐
e) Physiotherapist or occupational therapist ☐
f) Occupational health nurse or doctor ☐
g) Employer or manager (or colleagues if self-employed) ☐
h) Friend or family member ☐
i) Other *(please specify)* ☐

....

....

Please go to Question 21

For peer review only - http://bmjopen.bmj.com/site/about/guidelines.xhtml
For peer review only

SECTION B: WORK

14 When did you first return to work after your carpal tunnel release surgery?

d d m m y y y y

15 How much work-time did you miss between the date of your surgery and the date you first returned to work?
Please include all work-time missed, even if this had been pre-arranged with your employer, or was taken as annual leave. You can answer in hours, days or weeks, whichever applies.

hours days weeks

15.1 Was any of this time paid?
Please tick one box (and provide the amount of time, if applicable).

a) Yes, all of my time away from work was paid

b) Yes, some of my time away from work was paid
(please specify how much time was paid, you can use hours, days or weeks, whichever applies)

hours days weeks

c) No, none of my time off was paid

d) Not sure

16 Since your surgery, have you discussed when to return to work with anyone?
If yes, please answer the rest of Question 16; if no, please move on to Question 17.

Yes No

16.1 If yes, who did you discuss this with? Please tick all that apply.

a) Your surgeon or a member of the surgical team

b) Hospital nurse

c) GP or practice nurse

d) Hand therapist

e) Physiotherapist or occupational therapist

f) Occupational health nurse or doctor

g) Employer or manager (or colleagues if self-employed

h) Friend or family member

i) Other *(please specify)*

For peer review only - http://bmjopen.bmj.com/site/about/guidelines.xhtml

SECTION B: WORK

16.2 Please list any advice you have been given (since your surgery) about when and how to return to work?
This could include any activities to avoid or timescales to follow. If this advice came from more than one place, please indicate who advised what.

----

17 Since returning to work after your operation, have you needed to take any time off work because of a problem with your operated hand(s)/wrist(s)?
If yes, please answer Question 17.1; if no, please move on to Question 18.

Yes ☐ No ☐

17.1 If yes, how much time did you take off work?
Please answer in days or hours, whichever applies.

☐☐☐ hours ☐☐ days ☐☐ weeks

18 When you first returned to work after your surgery, did you work shorter hours than would be normal for your job as a direct result of your operation?
If yes, please answer the rest of Question 18; if no, please move on to Question 19.

Yes ☐ No ☐

18.1 Have you since gone back to working full hours?
If yes, please answer Question 18.2; if no, please move on to Question 19.

Yes ☐ No ☐

18.2 If yes, when did you return to full working hours?
If you do not know the exact date, approximately how many weeks did you work reduced hours?

☐☐☐☐☐☐☐☐
d d m m y y y y

a) Less than a week ☐

b) 1 – 2 weeks ☐

c) More than 2 weeks, but less than 3 weeks ☐

d) 3 weeks or longer ☐

For peer review only - http://bmjopen.bmj.com/site/about/guidelines.xhtml
For peer review only

SECTION B: WORK

19 **When you first returned to work after your surgery, did you need to alter or avoid any of your usual work duties as a direct result of your operation?**
If yes, please answer the rest of Question 19; if no, please move on to Question 20.

Yes ☐ No ☐

19.1 **Have you since gone back to full duties?**
If yes, please answer Question 19.2; if no, please move on to Question 20.

Yes ☐ No ☐

19.2 **If yes, when did you return to full working duties?**
If you do not know the exact date, approximately how many weeks did you have altered work duties?

d	d	m	m	y	y	y	y

a) Less than a week ☐

b) 1 – 2 weeks ☐

c) More than 2 weeks, but less than 3 weeks ☐

d) 3 weeks or longer ☐

20 **If you would like to give us any additional information about returning to work, please do so here:**

For peer review only

SECTION C: HAND AND WRIST SYMPTOMS

21 The following questions refer to your symptoms over the ***last 7 days***.
Please answer for each hand. Please tick one box for each row.

**21.1 How severe were the following symptoms in your RIGHT hand?**	**None**	**Mild**	**Moderate**	**Severe**	**Very severe**
a) Pain at night	☐	☐	☐	☐	☐
b) Pain during the daytime	☐	☐	☐	☐	☐
c) Numbness or tingling at night	☐	☐	☐	☐	☐
d) Numbness or tingling during the daytime	☐	☐	☐	☐	☐

**How often did the following symptoms in your RIGHT hand wake you up at night?**	**Never**	**Once**	**2 or 3 times**	**4 or 5 times**	**More than 5 times**
e) Pain	☐	☐	☐	☐	☐
f) Numbness or tingling	☐	☐	☐	☐	☐

**21.2 How severe were the following symptoms in your LEFT hand?**	**None**	**Mild**	**Moderate**	**Severe**	**Very severe**
a) Pain at night	☐	☐	☐	☐	☐
b) Pain during the daytime	☐	☐	☐	☐	☐
c) Numbness or tingling at night	☐	☐	☐	☐	☐
d) Numbness or tingling during the daytime	☐	☐	☐	☐	☐

**How often did the following symptoms in your LEFT hand wake you up at night?**	**Never**	**Once**	**2 or 3 times**	**4 or 5 times**	**More than 5 times**
e) Pain	☐	☐	☐	☐	☐
f) Numbness or tingling	☐	☐	☐	☐	☐

22 This question refers to the appearance (look) of your hands during the ***past 7 days***. Please tick one box for each hand.

	**Strongly agree**	**Agree**	**Neither agree nor disagree**	**Disagree**	**Strongly disagree**
a) I am satisfied with the appearance (look) of my **RIGHT** hand	☐	☐	☐	☐	☐
b) I am satisfied with the appearance (look) of my **LEFT** hand	☐	☐	☐	☐	☐

For peer review only

SECTION C: HAND AND WRIST SYMPTOMS

23 How do you rate your symptoms in your operated hand(s) now, compared to before your surgery? Please tick one box.

a) Completely cured ☐
b) Much better ☐
c) Unchanged ☐
d) Slightly better ☐
e) Worse ☐

24 The following questions ask specifically about your scar. Please think about your scar over the ***past 7 days.***

24.1 Has your scar been itchy?
If yes, please continue; if no, please move on to Question 24.2

Yes ☐ No ☐

Yes, it was itchy:	Sometimes ☐	Often ☐	Always ☐
And when it was itchy, it was:	Slightly itchy ☐	Fairly itchy ☐	Very itchy ☐

24.2 Has your scar caused you pain?
If yes, please continue; if no, please move on to Question 24.3

Yes ☐ No ☐

Yes, it was painful:	Sometimes ☐	Often ☐	Always ☐
And when it hurt, it was:	Slightly painful ☐	Fairly painful ☐	Very painful ☐

24.3 Has your scar been uncomfortable?
If yes, please continue; if no, please move on to Question 24.4

Yes ☐ No ☐

Yes, it was uncomfortable:	Sometimes ☐	Often ☐	Always ☐
And when it was uncomfortable, it was:	Slightly uncomfortable ☐	Fairly uncomfortable ☐	Very uncomfortable ☐

For peer review only - http://bmjopen.bmj.com/site/about/guidelines.xhtml

SECTION C: HAND AND WRIST SYMPTOMS

24.4 Has your scar felt numb?
If yes, please continue; if no, please move on to Question 24.5

Yes ☐ No ☐

Yes, it was numb:	Sometimes ☐	Often ☐	Always ☐
And when it felt numb, it was:	Slightly numb ☐	Fairly numb ☐	Very numb ☐

24.5 Have you had odd sensations in your scar e.g. tightening, pulling or pins and needles? If yes, please continue; if no, please move on to Question 24.6

Yes ☐ No ☐

Yes, I have had odd sensations:	Sometimes ☐	Often ☐	Always ☐

24.6 Has your scar caught on things e.g. clothing?
If yes, please continue; if no, please move on to Question 24.7

Yes ☐ No ☐

Yes, it has caught on things:	Sometimes ☐	Often ☐	Always ☐

24.7 Overall, how troublesome are the symptoms from your scar?
Please tick one box.

Not at all troublesome	A little troublesome	Fairly troublesome	Very troublesome	Unbearable
☐	☐	☐	☐	☐

For peer review only

SECTION D: HAND AND WRIST FUNCTION

The following questions refer to the function of your hands/wrists during the ***past 7 days***. Please answer all questions for the right and left sides, even if you do not experience any problems. Please tick one box for each question.

**25 RIGHT SIDE**	**Very well**	**Well**	**Adequately**	**Poorly**	**Very poorly**
a) Overall, how well did your ***right*** hand work?					
b) How well did your ***right*** fingers move?					
c) How well did your ***right*** wrist move?					
	**Very good**	**Good**	**Fair**	**Poor**	**Very poor**
d) How was the strength in your ***right*** hand?					
e) How was the sensation (feeling) in your ***right*** hand?					

**26 LEFT SIDE**	**Very well**	**Well**	**Adequately**	**Poorly**	**Very poorly**
a) Overall, how well did your ***left*** hand work?					
b) How well did your ***left*** fingers move?					
c) How well did your ***left*** wrist move?					
	**Very good**	**Good**	**Fair**	**Poor**	**Very poor**
d) How was the strength in your ***left*** hand?					
e) How was the sensation (feeling) in your ***left*** hand?					

The following questions refer to the ability of your hands to do certain tasks during the ***past 7 days***. If you do not do a certain task, please estimate the difficulty you would have in performing it. Please tick one box for every activity.

27 How difficult was it for you to perform the following activities using your RIGHT HAND?

	**Not at all difficult**	**A little difficult**	**Somewhat difficult**	**Moderately difficult**	**Very difficult**
a) Turn a door knob					
b) Pick up a coin					
c) Hold a glass of water					
d) Turn a key in a lock					
e) Hold a frying pan					

For peer review only - http://bmjopen.bmj.com/site/about/guidelines.xhtml

SECTION D: HAND AND WRIST FUNCTION

28 How difficult was it for you to perform the following activities using your LEFT HAND?

		Not at all difficult	A little difficult	Somewhat difficult	Moderately difficult	Very difficult
a)	Turn a door knob					
b)	Pick up a coin					
c)	Hold a glass of water					
d)	Turn a key in a lock					
e)	Hold a frying pan					

29 How difficult was it for you to perform the following activities using BOTH HANDS?

		Not at all difficult	A little difficult	Somewhat difficult	Moderately difficult	Very difficult
a)	Open a jar					
b)	Button a shirt/blouse					
c)	Eat with a knife/fork					
d)	Carry a grocery bag					
e)	Wash dishes					
f)	Wash your hair					
g)	Tie shoelaces/knots					

The following questions refer to your satisfaction with your hands/wrists during the ***past 7 days***. Please tick one box for each question.

30 How satisfied were you with your RIGHT hand/wrist during the *past 7 days*?

	RIGHT HAND	Very satisfied	Somewhat satisfied	Neither satisfied or dissatisfied	Somewhat dissatisfied	Very dissatisfied
a)	Overall function of your hand					
b)	Movement of the fingers					
c)	Movement of your wrist					
d)	Strength of your hand					
e)	Pain level of your hand					
f)	Sensation (feeling) of your hand					

For peer review only - http://bmjopen.bmj.com/site/about/guidelines.xhtml

SECTION D: HAND AND WRIST FUNCTION

31 How satisfied were you with your LEFT hand/wrist during the *past 7 days*?

	LEFT HAND	Very satisfied	Somewhat satisfied	Neither satisfied or dissatisfied	Somewhat dissatisfied	Very dissatisfied
a)	Overall function of your hand	☐	☐	☐	☐	☐
b)	Movement of the fingers	☐	☐	☐	☐	☐
c)	Movement of your wrist	☐	☐	☐	☐	☐
d)	Strength of your hand	☐	☐	☐	☐	☐
e)	Pain level of your hand	☐	☐	☐	☐	☐
f)	Sensation (feeling) of your hand	☐	☐	☐	☐	☐

32 If you would like to give us any additional information about your hand and wrist function, please do so here:

Thank you for completing this questionnaire!
Please return it to the REACTS team
using the pre-paid envelope.

REACTS

If you have any questions or would like any additional information, please contact Lisa Newington on:
ln@mrc.soton.ac.uk | 023 8077 7624 | 07866 997732

For peer review only - http://bmjopen.bmj.com/site/about/guidelines.xhtml

SUPPLEMENTARY TABLES

1. Cox proportional hazards univariate analyses of the association between demographic and general health factors and the duration of work absence after carpal tunnel release (non-significant findings)

	N	Median time to return to work (days)	Inter quartile range	Hazards ratio [1]	95% Confidence intervals	P value
Body mass index (kg/m²) (censored 1 normal, 1 overweight, 2 obese; 13 missing)						
Normal (18.5-24.9)	47	20	12-33	1	-	-
Over (25.0-29.9)	64	19	12.5-29	0.98	0.65, 1.46	0.91
Obese (≥30)	67	21	10-40	0.88	0.60, 1.28	0.50
Surgery to dominant hand [2] (censored 3 yes, 2 no; no missing data)						
Yes	128	19.5	12-33	1	-	-
No	62	22	12-35	0.83	0.61, 1.14	0.25
General health status (censored 5 excellent; 1 missing)						
Excellent/very good/ good	165	19	12-32	1	-	-
Fair/poor	24	28.5	12.5-42	0.74	0.47, 1.16	0.19
Number of comorbidities [3] (censored 3 none, 1 one; 1 missing)						
None	49	18	7-32	1	-	-
One	67	24	14-42	0.87	0.59, 1.27	0.47
Two or more	74	19.5	10-29	1.11	0.76, 1.62	0.60
Number of disability comorbidities [3] (censored 3 none, 2 one; no missing data)						
None	130	19	9-33	1	-	-
One	39	26	13-31	0.75	0.52, 1.08	0.12
Two or more	21	20	14-31	0.81	0.50, 1.32	0.40
Number of somatising symptoms [4] (censored 1 none, 1 one, 3 two or more; 1 missing)						
None	96	19	10.5-35	1	-	-
One	51	22	13-34	0.98	0.70, 1.38	0.91
Two or more	42	19.5	14-31	0.84	0.58, 1.22	0.36
SF36 Mental health score (tertiles) [5] (censored 2 poor, 1 intermediate, 2 good; 2 missing)						
Poor (22.2-57.8)	60	24	16-39	0.71	0.50, 1.03	0.07
Intermediate (60.0-75.6)	68	18.5	11-36.5	0.88	0.62, 1.25	0.48
Good (77.8-97.8)	60	16.5	7-30	1	-	-

Total sample size n=195. Median duration of work absence relates to the 190 non-censored events.

1. Adjusted for age and sex.

2. Considered and the non-dominant hand for those who reported ambidexterity (n=4) and dominant hand for those undergoing simultaneous bilateral surgery (n=2).

3. Adapted from the self-administered co-morbidity questionnaire [1].

4. Adapted from the four-dimensional symptom questionnaire [2].

5. Taken from the SF-36 with data-driven tertiles [3].

For peer review only

2. Cox proportional hazards univariate analyses of the association between health beliefs and the duration of work absence after carpal tunnel release (non-significant findings)

	N	Median time to return to work (days)	Inter quartile range	Hazards ratio [1]	95% Confidence intervals	P value
Believe will be unable to use hand normally in 3 months [2] (censored 5 no; 2 missing)						
No	168	20	12-32.5	1	-	-
Yes	20	20.5	14.5-40	1.04	0.65, 1.67	0.87
Blames self for hand problem [2] (censored 5 no; 2 missing)						
No	162	20	12-35	1	-	-
Yes	26	22	14-31	1.08	0.70, 1.66	0.73
Lack of support from family/friends [2] (censored 4 no, 1 yes; 2 missing)						
No	156	20	12-32.5	1	-	-
Yes	32	19.5	13-40	0.88	0.60, 1.29	0.51
Problem runs in the family/born with a weakness [3] (censored 3 no, 2 agree/strongly agree; 2 missing)						
No	134	20	12-35	1	-	-
Agree/strongly agree	54	23	12-31	0.88	0.63, 1.21	0.42
Stress made the problem worse [3] (censored 5 no; 2 missing)						
No	169	20	12-33	1	-	-
Agree/strongly agree	19	28	14-40	0.79	0.48, 1.28	0.33
Lack of exercise contributed to the problem [3] (censored 5 no; 2 missing)						
No	172	20	13-33	1	-	-
Agree/strongly agree	16	15	10.5-34.5	1.23	0.73, 2.06	0.43
Getting older contributes to the problem [3] (censored 3 no, 2 yes; 3 missing)						
No	79	26	14-42	1	-	-
Agree/strongly agree	108	16	10-29	1.28	0.94, 1.74	0.11
Work probably didn't cause the problem, but made it worse [3] (censored 2 no, 3 yes; 4 missing)						
No	100	20.5	12-35	1	-	-
Agree/strongly agree	86	19.5	13-31	1.01	0.75, 1.35	0.95
Pain catastrophisation to at least a moderate degree [4] (censored 3 no, 2 yes; missing 2)						
No	133	19	10-33	1	-	-
Yes	55	22	15-31	0.81	0.59, 1.12	0.21

Total sample size n=195. Median duration of work absence relates to the 190 non-censored events.

1. Adjusted for age and sex.

2. Reported on a 0-10 scale and dichotomised to no (0-6) and yes (7-10) [4].

3. Reported on a 5-point scale and dichotomised to agree/strongly agree and neither agree nor disagree/disagree/disagree strongly [5].

4. Pain catastrophizing scale dichotomised to those who reported catastrophising pain thoughts and feelings to at least a moderate degree in response to all questions and those who did not [6].

For peer review only

3. Cox proportional hazards univariate analyses of the association between clinical and surgical factors and the duration of work absence after carpal tunnel release (non-significant findings)

	N	Median time to return to work (days)	Inter quartile range	Hazards ratio [1]	95% Confidence intervals	P value
Katz hand diagram score for side of surgery [2] (censored 3 classics/probable, 2 possible/unlikely; 4 missing)						
Classic/probable	126	22	14-38	1	-	-
Possible/unlikely	60	16.5	9.5-30.5	1.10	0.81, 1.51	0.54
Duration of symptoms (censored 1 ≤ 1 year, 4 > 1 year; 2 missing)						
≤ 1 year	47	19	10-31	1	-	-
> 1 year	141	20	13-34	0.86	0.62, 1.21	0.38
Nerve conduction studies performed (censored 3 no, 2 yes; 40 missing)						
No	71	21	14-35	1	-	-
Yes	79	20	11-33	1.14	0.83, 1.58	0.41
Type of suture material (censored 4 non-absorbable; 49 missing)						
Non-absorbable	119	21	12-38	1	-	-
Absorbable	23	20	13-29	1.15	0.73, 1.83	0.55
MHQ function score for side of surgery (tertiles) [3] (censored 3 poor, 1 intermediate, 1 good; 2 missing)						
Poor (0-40)	79	20	13-41	0.80	0.57, 1.12	0.19
Intermediate (44-55)	48	19.5	13.5-33	0.81	0.55, 1.19	0.29
Good (60-100)	61	20	7-29	1	-	-
MHQ bilateral activities of daily living score (tertiles) [3] (censored 1 poor, 4 intermediate; 1 missing)						
Poor (0-61)	72	19	12.5-33.5	0.96	0.66, 1.38	0.81
Intermediate (64-82)	55	21	13-38	0.70	0.49, 1.01	0.06
Good (83-100)	62	20	10-31	1	-	-
MHQ activities of daily living score, side of surgery (tertiles) [3] (censored 2 poor, 3 intermediate; 1 missing)						
Poor (0-55)	63	20	12-42	0.81	0.56, 1.18	0.27
Intermediate (58-80)	63	20	14-35	0.86	0.61, 1.23	0.42
Good (85-100)	63	20	9-32	1	-	-
MHQ satisfaction score for side of surgery (tertiles) [3] (censored 3 poor, 2 intermediate; no missing data)						
Poor (0-25)	88	21.5	13-42	0.78	0.55, 1.11	0.16
Intermediate (29-50)	49	18	14-29	0.87	0.59, 1.28	0.48
Good (54-100)	53	20	9-31	1	-	-
Satisfaction with appearance for side of surgery [4] (censored 2 satisfied, 3 dissatisfied; 1 missing)						
Satisfied	136	20	10.5-31.5	1	-	-
Dissatisfied	53	20	13-40	0.84	0.61, 1.16	0.29

Total sample size n=195. Median duration of work absence relates to the 190 non-censored events.

1. Adjusted for age and sex.

2. Adapted from Calfee et al. [7].

3. Michigan Hand Questionnaire [8]. Data-driven tertiles.

4. Reported on a 5-point scale in response to the statement: I am satisfied with the appearance (look) of my hand. Dichotomised as satisfied (strongly agree/agree) and dissatisfied (neither agree nor disagree, disagree, strongly disagree).

For peer review only

4. Cox proportional hazards univariate analyses of the association between occupational factors and the duration of work absence after carpal tunnel release (non-significant findings)

	N	Median time to return to work (days)	Inter quartile range	Hazards ratio [1]	95% Confidence intervals	P value
Have more than one paid job (censored 4 no, 1 yes; 1 missing)						
No	177	20	12-32	1	-	-
Yes	12	26.5	19-42	0.61	0.34, 1.11	0.11
Total paid work hours per week [2] (censored 4 ≤37.5, 1 >37.5; no missing data)						
≤37.5	97	22	15-38	1	-	-
>37.5	93	16	6-29	1.25	0.88, 1.79	0.21
Number of work days per week (censored 1 <5, 4 5; no missing data)						
<5	55	23	15-35	1	0.72, 1.39	0.98
5	116	19	10.5-33	1	-	-
>5	19	13	6-31	1.28	0.77, 2.14	0.34
Sick leave taken for this problem in the last month (censored 5 no; 16 missing)						
No	154	20	12-33	1	-	-
Yes	20	15	9.5-30	0.99	0.61, 1.60	0.95
Sick leave taken for another problem in the last month (censored 4 no, 1 yes; 20 missing)						
No	152	19	10.5-31	1	-	-
Yes	18	30	21-42	0.67	0.41, 1.09	0.10
Received advice about return to work after surgery (censored 5 yes; 3 missing)						
Yes	134	19	12-31	1	-	-
No	53	22	14-41	0.92	0.67, 1.27	0.61
Required to work to tight deadlines [3] (censored 3 no, 2 yes; missing 3)						
No	75	23	14-40	1	-	-
Yes	112	18	9.5-31	1.19	0.89, 1.61	0.24
Use power tools that make the hand/arm vibrate [3] (censored 5 no; missing 7)						
No	145	19	12-32	1	-	-
Yes	38	22	14-42	0.68	0.44, 1.05	0.08
Work with hands above shoulder height for >1 hour [3] (censored 4 no, 1 yes; missing 9)						
No	147	20	12-33	1	-	-
Yes	34	19.5	13-33	0.89	0.60, 1.32	0.56
Work with neck bent forward for >2 hours [3] (censored 3 no, 2 yes; missing 3)						
No	114	21	13-39	1	-	-
Yes	73	18	8-31	1.19	0.88, 1.60	0.26
Work with neck twisted for >30 minutes [3] (censored 4 no, 1 yes; missing 7)						
No	136	20	12-34	1	-	-
Yes	47	22	13-32	0.93	0.66, 1.32	0.70
Drive for >1 hour per day [3] (censored 5 no; missing 5)						
No	115	21	13-38	1	-	-
Yes	70	16	7-31	1.22	0.88, 1.68	0.24

Total sample size n=195. Median duration of work absence relates to the 190 non-censored events.

1. Adjusted for age and sex.

2. Dichotomised by the median for the sample population

3. As part of the normal working day [9].

For peer review only

References

1. Sangha O, Stucki G, Liang MH et al. The self-administered comorbidity questionnaire: a new method to assess comorbidity for clinical and health services research *Arthritis & Rheumatism* 2003;49(2):156-63.
2. Terluin B, van Marwijk HW, Adèr HJ et al. The Four-Dimensional Symptom Questionnaire (4DSQ): a validation study of a multidimensional self-report questionnaire to assess distress, depression, anxiety and somatization. *BMC Psychiatry* 2006;6(1):34.
3. Ware J, Sherbourne C. The MOS 36-item short-form health survey (SF-36): I. conceptual framework and item selection. *Medical Care* 1992;30:473-83.
4. Hansen TB, Dalsgaard J, Meldgaard A et al. A prospective study of prognostic factors for duration of sick leave after endoscopic carpal tunnel release. *BMC Musculoskelet Disord* 2009;10:144.
5. Palmer K, Calnan M, Wainwright D et al. Upper limb pain in primary care: health beliefs, somatic distress, consulting and patient satisfaction. *Family Practice* 2006;23(6):609-17.
6. Bot AG, Becker SJ, Bruijnzeel H et al. Creation of the abbreviated measures of the Pain Catastrophizing Scale and the Short Health Anxiety Inventory: the PCS-4 and SHAI-5. *J Musculoskelet Pain* 2014;22(2):145-51.
7. Calfee RP, Dale AM, Ryan D et al. Performance of simplified scoring systems for hand diagrams in carpal tunnel syndrome screening. *J Hand Surg Am* 2012;37(1):10-17.
8. University of Michigan Medical School. The Michigan Hand Questionnaire: http://mhq.lab.medicine.umich.edu/. Accessed 04/04/2019.
9. Karasek R, Brisson C, Kawakami N et al. The Job Content Questionnaire (JCQ): an instrument for internationally comparative assessments of psychosocial job characteristics. *J Occup Health Psychol* 1998;3(4):322-55.

For peer review only

STROBE checklist (cohort study)

Item	Recommendation	Location
**Title and abstract**		
Title	Indicate study design	Title
**Introduction**		
Background	Explain scientific background and rationale	Introduction
Objectives	State objectives and any pre-specified hypotheses	Introduction
**Methods**		
Study design	Present key elements of the study design early	Introduction & methods
Setting	Describe the setting, locations and relevant dates	Methods
Participants	Give eligibility criteria, sources and methods of selection of participants, and methods of follow-up	Methods & table 1
Variables	Clearly define all outcomes, exposures, predictors, potential confounders and effect modifiers	Methods & supplementary files 1-2
Data sources	Give sources of data and methods of assessment	Methods & tables 2-5
Bias	Describe and effects to address potential sources of bias	Methods & discussion
Study size	Explain how the study size was determined	Methods
Quantitative variables	Described how quantitative variables were handled in the analyses	Tables 2-5
Statistical methods	Described all statistical methods<br>Explain how missing data were addressed<br>Explain how lost to follow-up was addressed	Statistical methods & tables 2-5
**Results**		
Participants	Reported the number of individuals at each stage of the study<br>Give reasons for non-participation	Figure 1<br>Figure 1
Descriptive data	Give characteristics of study participants<br>Indicate number of participants for each variables of interest<br>Summarise follow-up time	Tables 2-5<br>Tables 2-5<br>Results
Outcome data	Report number of outcome events over time	Results
Main results	Give adjusted and unadjusted estimates and measures of precision. Make clear which confounders were adjusted for	Table 4 & supplementary file 3
Other analyses	Reported any subgroup analyses	Table 4 & supplementary file 3

For peer review only

Sickness absence after carpal tunnel release: a multi-centre prospective cohort study

Journal:	*BMJ Open*
Manuscript ID	bmjopen-2020-041656.R3
Article Type:	Original research
Date Submitted by the Author:	18-Jan-2021
Complete List of Authors:	Newington, Lisa; University of Southampton Faculty of Medicine, MRC Versus Arthritis Centre for Musculoskeletal Health and Work, MRC Lifecourse Epidemiology Unit; Guy's and Saint Thomas' NHS Foundation Trust, Hand Therapy<br>Ntani, G; University of Southampton Faculty of Medicine, MRC Versus Arthritis Centre for Musculoskeletal Health and Work, MRC Lifecourse Epidemiology Unit<br>Warwick, David; University Hospital Southampton NHS Foundation Trust, Hand Surgery; University of Southampton Faculty of Medicine<br>Adams, Jo; University of Southampton, Centre for Innovation and Leadership in Health Sciences, School of Health Sciences, Faculty of Environment and Life Sciences; University of Southampton, Centre for Sport, Exercise and Osteoarthritis Research Versus Arthritis<br>Walker-bone, Karen; University of Southampton Faculty of Medicine, MRC Versus Arthritis Centre for Musculoskeletal Health and Work, MRC Lifecourse Epidemiology Unit
**Primary Subject Heading**:	Surgery
Secondary Subject Heading:	Epidemiology, General practice / Family practice, Occupational and environmental medicine
Keywords:	Hand & wrist < ORTHOPAEDIC & TRAUMA SURGERY, PRIMARY CARE, OCCUPATIONAL & INDUSTRIAL MEDICINE

SCHOLARONE™
Manuscripts

I, the Submitting Author has the right to grant and does grant on behalf of all authors of the Work (as defined in the below author licence), an exclusive licence and/or a non-exclusive licence for contributions from authors who are: i) UK Crown employees; ii) where BMJ has agreed a CC-BY licence shall apply, and/or iii) in accordance with the terms applicable for US Federal Government officers or employees acting as part of their official duties; on a worldwide, perpetual, irrevocable, royalty-free basis to BMJ Publishing Group Ltd ("BMJ") its licensees and where the relevant Journal is co-owned by BMJ to the co-owners of the Journal, to publish the Work in this journal and any other BMJ products and to exploit all rights, as set out in our licence.

The Submitting Author accepts and understands that any supply made under these terms is made by BMJ to the Submitting Author unless you are acting as an employee on behalf of your employer or a postgraduate student of an affiliated institution which is paying any applicable article publishing charge ("APC") for Open Access articles. Where the Submitting Author wishes to make the Work available on an Open Access basis (and intends to pay the relevant APC), the terms of reuse of such Open Access shall be governed by a Creative Commons licence – details of these licences and which Creative Commons licence will apply to this Work are set out in our licence referred to above.

Other than as permitted in any relevant BMJ Author's Self Archiving Policies, I confirm this Work has not been accepted for publication elsewhere, is not being considered for publication elsewhere and does not duplicate material already published. I confirm all authors consent to publication of this Work and authorise the granting of this licence.

For peer review only

TITLE

Sickness absence after carpal tunnel release: a multi-centre prospective cohort study

AUTHORS

Lisa Newington [1, 2] *

Georgia Ntani [1]

David Warwick [3]

Jo Adams [4, 5]

Karen Walker-Bone [1]

AFFILIATIONS

1. MRC Versus Arthritis Centre for Musculoskeletal Health and Work, MRC Lifecourse Epidemiology Unit, University of Southampton, Southampton, UK.
2. Hand Therapy, Guy's and St Thomas' NHS Foundation Trust, St Thomas' Hospital, London, UK.
3. Hand Surgery, University Hospital Southampton NHS Foundation Trust and Faculty of Medicine, University of Southampton, Southampton, UK.
4. Centre for Innovation and Leadership in Health Sciences, School of Health Sciences, Faculty of Environment and Life Sciences, University of Southampton, Southampton, UK.
5. Centre for Sport, Exercise and Osteoarthritis Research Versus Arthritis, University of Southampton, Southampton, UK.

*** Corresponding author**

Address: Hand Therapy, Guy's and St Thomas' NHS Foundation Trust, St Thomas' Hospital, London, SE1 7EH, UK.

Email: l.newington@nhs.net

Telephone: 0207 7188 4174

Fax: none

KEY WORDS

Carpal tunnel syndrome, carpal tunnel release, return to work, advice, sickness absence, cohort study

Word count: 4,042

For peer review only

TITLE

Sickness absence after carpal tunnel release: a multi-centre prospective cohort study

ABSTRACT

Objectives: To describe when patients return to different types of work after elective carpal tunnel release (CTR) surgery and identify the factors associated with the duration of sickness absence.

Design: Multi-centre prospective observational cohort study.

Setting and participants: Participants were recruited pre-operatively from 16 UK centres and clinical, occupational and demographic information were collected. Participants completed a weekly diary and questionnaires at four and 12 weeks post-operatively.

Outcomes: The main outcome was duration of work absence from date of surgery to date of first return to work.

Results: 254 participants were enrolled in the study and 201 provided follow-up data. Median duration of sickness absence was 20 days (range 1-99). Earlier return to work was associated with having surgery in primary care and a self-reported work role involving more than four hours of daily computer use. Being female and entitlement to more than a month of paid sick leave were both associated with longer work absences. Duration of work absence was strongly associated with the expected duration of leave, as reported by participants before surgery. Earlier return to work was not associated with poorer clinical outcomes reported 12 weeks after CTR.

For peer review only

Conclusions: There was wide variation in the duration of work absence after CTR across all occupational categories. A combination of occupational, demographic and clinical factors was associated with the duration of work absence, illustrating the complexity of return to work decision-making. However, pre-operative expectations were strongly associated with the actual duration of leave. We found no evidence that earlier return to work was harmful. Clear, consistent advice from clinicians pre-operatively setting expectations of a prompt return to work could reduce unnecessary sickness absence after CTR. To enable this, clinicians need evidence-informed guidance about appropriate timescales for the safe return to different types of work.

STRENGTHS AND LIMITATIONS OF THIS STUDY

- This multi-centre, prospective study with a large sample size provides robust evidence to understand return to work issues after carpal tunnel release surgery.
- Participants were recruited from primary care, secondary care and private practice sites, representing the breath of locations where carpal tunnel release is performed in the UK.
- Work absence was the primary outcome and a clear definition was used for its duration with data collected contemporaneously to limit recall bias.
- A standardised method was used to categorise occupations and measure occupational exposures, although this relied on job title, which may not be a true reflection of actual occupational activity.
- All participants underwent open carpal tunnel release, however the method of carpal tunnel syndrome diagnosis was not independently verified and different case definitions for carpal tunnel syndrome may have been included.

For peer review only

INTRODUCTION

Carpal tunnel syndrome (CTS) occurs when the median nerve becomes compressed within the carpal tunnel at the wrist. Typical sensory symptoms include pain, paraesthesia and/or numbness in the thumb, index, middle and radial half of the ring finger; and motor symptoms include progressive wasting of the thenar muscles. CTS is often associated with marked functional difficulty[1] and treatment is targeted at reducing the median nerve compression by splinting, corticosteroid injection or carpal tunnel release (CTR) surgery[2, 3].

Recent estimates suggest that more than 90,000 CTR procedures will be performed annually in the English NHS by 2025[4], and as the peak incidence for CTS falls within the working lifetime[5], many of these patients will need to return to work after their CTR. However, there is currently no evidence on which to base recommendations about when it might be safe to return to functional activities, including work, after carpal tunnel release (CTR). Our previous survey of UK hand surgeons, primary care surgeons and hand therapists found that clinicians recommended a wide range of times to return to three specified job roles after CTR: 0-30 days for desk-based work (e.g. keyboard, mouse, writing and telephone); 1-56 days for repetitive light manual work (driving, delivery, stacking); and 1-90 days for heavy manual work (e.g. construction)[6]. However, there has not previously been a prospective study of CTR patients in the UK in which time to return to work was the primary outcome. Therefore, it is not known when UK patients return to different occupational activities after CTR or what influences the duration of work absence. It is also unclear whether earlier return to work has a detrimental effect on post-operative clinical outcomes. Possible consequences of returning to work too soon after CTR include wound dehiscence, infection, and delayed healing. Conversely, delayed return to work may increase the risk of progression to long-term sick leave[7] and produce a financial burden for the individual, employer or state.

A systematic review of the prognostic factors associated with return to work after CTR identified 11 studies which evaluated more than 90 potential prognostic factors[8]. Longer durations of work absence after CTR were found to be associated with: receipt of workers' compensation[9]; manual work[10-12]; longer expected durations of work absence[10]; being on sick leave before CTR surgery[13]; self-blame for the hand problem[13]; and beliefs that the symptoms were caused by work[12].

Much of the existing research has been conducted at single sites and involved small numbers of participants. Furthermore, very few studies have specifically explored the influence of a range of occupational factors. The current multi-centre prospective cohort study was designed to explore when patients returned to different types of work after CTR and the demographic, clinical and occupational factors associated with duration of work absence. We also investigated whether earlier return to work was associated with poorer clinical outcomes assessed at 12 weeks after CTR.

METHODS

This was a prospective cohort study and a convenience sample of participants were recruited from 16 sites in England and Wales between March 2017 and August 2018 (ethics approval: IRAS 209840, 16/WA/0390). Recruitment took place before CTR surgery, either at the time of listing for surgery, during pre-operative screening, or on the day of surgery. At each site, the patient CTR pathway and treatment was carried out as usual. Sites were NHS secondary care (hospital setting), NHS primary care and private hand surgery facilities, representing the range of UK healthcare facilities where CTR is performed. Provision of CTR in the UK was explored through discussion with relevant national organisations (British Society for Surgery of the Hand

For peer review only

and Association for Surgeons in Primary Care). Sites were recruited through National Institute for Health Research infrastructure.

Eligibility criteria are shown in Table 1. Baseline demographic, general health and occupational information was collected via self-completed questionnaire at the time of recruitment. The questionnaire also included standardised measures of CTS symptoms[14-16] and hand function[17]. Questionnaire content was informed by the clinical, demographic and occupational factors previously identified in a systematic review of prognostic factors for return to work after CTR[8], and developed in collaboration with our patient advisory group. The reasoning for item inclusion is provided in supplementary file 1.

TABLE 1. Study eligibility criteria

**Self-selected by potential participants**
1. Aged over 18 and referred for carpal tunnel release surgery
2. Routinely work in paid employment for at least 20 hours per week
3. Plan to return to work after carpal tunnel release surgery
4. Have not previously had carpal tunnel release surgery on either hand
5. Have not previously had a serious injury to the same wrist/hand
**Assessed by recruiting clinician**
1. No planned surgical procedures for conditions other than carpal tunnel syndrome

Follow-up questionnaires were completed four and 12 weeks after CTR and collected information about return to work, work functioning, scar symptoms, CTS symptoms, and hand function. Study questionnaires are provided as supplementary files 2-3. Participants were also asked to complete a short weekly diary from the day after surgery until return to work, detailing whether they had returned to work that week, and if so, the date of return. Steps were taken to minimise loss to follow-up after recruitment. To maximise retention, we

For peer review only

incentivised with a shopping voucher on completion of the study (£10) and sent up to three reminders using a combination of post, email and text.

Surgical information was collected from the medical records by a member of the participant's clinical team. This included: date of CTR, operated hand(s), nature of anaesthetic, incision size, additional procedures, unexpected findings and suture material. Date, side of CTR and anaesthetic (general/local) were also reported by participants for cross-checking.

Public and patient involvement

This research was supported by a patient advisory group consisting of six individuals who had previously undergone CTR at different UK sites. Study questionnaires were developed in collaboration with the patient advisors and these individuals also provided their feedback on the preliminary findings.

Statistical methods

Comparisons were made between those who dropped out of the study before providing any follow-up data and those in the final study sample using pre-specified demographic, clinical and occupational variables (Table 2).

Manual and non-manual work was coded from job title and industry using the UK Standard Occupational Classification[18, 19]. Return to work time was calculated from the date of surgery to the date of first return to work (as reported by participants).

A Cox proportional hazards model was used to explore the factors associated with return to work time, and the assumptions of the model were tested. Baseline and operative variables

For peer review only

were assessed in univariable analyses and those which were significant ($p<0.05$) were included as covariates in the final model. All regression analyses were adjusted for age and sex.

Participants were defined as having a poor outcome if they reported one or more of the following: global rating of change score of “worse”, “unchanged” or “slightly improved” (12 weeks after CTR)[20]; scar symptoms described as “unbearable”, “very troublesome” or “fairly troublesome” (12 weeks after CTR); use of antibiotics for an incision site infection after returning to work; and additional sick-leave related to the CTR after returning to work. The duration of work absence for those with/without poor outcomes were compared using Wilcoxon rank-sum test. In addition, participants were dichotomised to those who returned to work within/after seven, 14 and 28 days of surgery and the prevalence rates of poor outcomes were compared using Chi^2 for each time period. These time points were based on the median clinician-recommended return to work time that we reported previously[6].

There was no imputation for missing data. Missing values were coded as a separate category for each of the variables included, and participant numbers are provided for each variable in the accompanying tables.

RESULTS

A total of 254 individuals completed the baseline questionnaire and 201 (79%) provided follow-up data. Participant numbers and loss to follow-up for each study component are shown in Figure 1. Participant demographics and comparisons between those who remained in the study and those who dropped out before follow-up are shown in Table 2.

Figure 1. Participant numbers for each stage of the cohort study

For peer review only

TABLE 2. Participant demographics assessed at baseline in comparison with those lost to follow-up

	**Study participants n=201 (%)**	**Lost to follow-up n=53 (%)**
Mean age in years (SD)	52.0 (9.16)	44.4 (9.55)
Sex		
Male	76 (37.8)	20 (37.7)
Female	125 (62.2)	33 (62.3)
Body mass index (kg/m^2)		
Normal (18.5-24.9)	48 (23.9)	9 (17.0)
Overweight (25-29.9)	66 (32.8)	16 (30.2)
Obese (≥30)	73 (36.3)	22 (41.5)
Smoking status		
Never smoked	109 (54.2)	26 (49.1)
Current/ex-smoker	90 (44.8)	27 (50.9)
General health		
Excellent/very good/good	174 (86.6)	42 (79.3)
Fair/poor	26 (12.9)	11 (20.8)
Number of comorbidities		
None	54 (26.9)	21 (39.6)
One	70 (34.8)	13 (24.5)
Two or more	77 (38.3)	19 (35.9)
Number of disabling comorbidities		
None	138 (68.7)	35 (66.0)
One	41 (20.4)	9 (17.0)
Two or more	22 (11.0)	9 (17.0)
Mean SF-36 mental health score (SD) [a]	65.6 (17.20)	60.3 (20.41)
Mean bilateral CTS-6 score (SD) [b]	2.8 (0.77)	3.0 (0.73)
Mean MHQ bilateral activities of daily living score (SD) [c]	68.8 (23.64)	55.7 (28.62)
Mean MHQ work function score (SD) [c]	66.1 (22.26)	60.6 (22.61)
Type of job contract		
Employed (permanent contract)	164 (81.6)	37 (69.8)
Self-employed	31 (15.4)	13 (24.5)
Employed (temporary or zero hours contract)	5 (2.5)	3 (5.7)
Type of work [d]		
Manual	77 (39)	31 (58)
Non-manual	123 (61)	22 (42)
Median level of job demand on hands/wrists (IQR) [e]	9 (7-10)	10 (7-10)
Job satisfaction		
Very satisfied	87 (43.3)	24 (45.3)
Satisfied/fairly satisfied	92 (45.8)	24 (45.3)
Dissatisfied/very dissatisfied	20 (10.0)	5 (9.4)
Median expected work absence in days (IQR)	14 (7-28)	14 (5-21)
Expected availability of sick pay		
≤1 month	50 (24.9)	21 (39.6)
>1 month	94 (46.8)	11 (20.8)
Unsure	57 (28.4)	21 (39.6)

For peer review only

Study site [f]		
NHS primary care	73 (36.3)	13 (24.5)
NHS Secondary care	101 (50.3)	32 (60.4)
Private hand surgery facilities	27 (13.4)	8 (15.1)

[a.] SF-36 mental health score ranges from 0-100, higher scores indicate better mental health[21].
[b.] CTS-6 symptom score ranges from 1-5, higher scores indicate more severe symptoms[14].
[c.] Michigan Hand Questionnaire ranges from 0-100, higher scores indicate better functioning[17].
[d.] Classified using the Office for National Statistics Standard Occupational Classification 2010[18, 19].
[e.] Job demand scale range 0-10, 10 indicating very demanding on hands/wrists[13].
[f.] Location where the carpal tunnel release surgery was performed. Surgery in primary care was performed by General Practitioners who had completed additional training.

Eighty-six participants (43%) were recruited pre-operatively on the day of their CTR. For the remaining 115 participants, the median time between recruitment and CTR was 14 days (interquartile range [IQR] 5-40). The first follow-up questionnaire was completed a median of 32 days after CTR (IQR 29-38) and the final questionnaire was completed a median of 92 days after CTR (IQR 86-105). All participants underwent open CTR as a day case, and all but two had unilateral surgery. Another two participants required median nerve neurolysis, and one participant was noted to have a vascular abnormality. Sixty-two percent of participants (n=125) were expecting to have a CTR for their other hand in the future. Other baseline demographic and surgical factors are shown in Table 3.

TABLE 3. Participant demographic and surgical factors

	Number of participants n=201 (%)
Age (years)	
26-40	23 (11.4)
41-55	101 (50.3)
≥ 55	77 (38.3)
Hand dominance	
Right	178 (88.6)
Left	18 (9.0)
Ambidextrous	5 (2.5)
Side of surgery [a]	
Dominant hand	134 (66.7)
Non-dominant hand	65 (32.3)
Bilateral surgery	2 (1.0)
Surgical speciality and grade	

For peer review only

Consultant (plastics/orthopaedics)	64 (31.8)
Registrar (plastics/orthopaedics)	33 (16.4)
General practitioner	62 (30.9)
Not reported	42 (20.9)
Incision type [b]	
Mini open incision	129 (64.2)
Traditional incision	2 (1.0)
Not reported	70 (34.8)
Suture material	
Absorbable	24 (11.9)
Non-absorbable	126 (62.7)
Not reported	51 (24.4)

[a.] Considered as the non-dominant hand for those who reported ambidexterity.
[b.] Mini open incision defined as distal to the distal wrist crease and traditional open excision extending proximally.

The majority of participants (62%) worked five days per week (range 2-7) and the median number of weekly paid work hours was 37.5 (IQR 31-45). Two participants (1%) did not return to work during the 12-week study period: one reported that they had been made redundant and the other that their job was no longer available. Four participants (2%) had incomplete data (missing return to work date or CTR date) meaning that the duration of work absence could not be calculated. These six individuals were not included in the analyses of return to work time, leaving a total sample size of 195. An additional five participants reported that they had not returned to work, but planned to do so in the future. These individuals were included in the return to work analysis, censored to the time of last follow-up.

The median duration of work absence after CTR was 20 days (IQR 12-33). Manual workers took longer to return than non-manual workers: 23.5 days (IQR 14-41) compared with 18 days (IQR 9-31). Those who were self-employed returned to work earlier than those who were employed: 13 days (IQR 6-19) compared with 22 days (IQR 14-38). Return to work times are shown in Figure 2. The majority of participants returned to work on a Monday (43%). Approximately 15% returned each day between Tuesday-Thursday, then ~5% returned each

For peer review only

day from Friday-Sunday. More than half of participants (59%) reported that they needed to modify their work duties to some extent when they first returned to work. Of these, 62% had resumed full duties within five weeks.

Univariable analyses of the relationship between baseline factors and the duration of work absence found 17 factors (age- and sex-adjusted) that were significantly associated with time to return to work and were entered into the multivariable model, in which five factors remained significant (Table 4). Sensitivity analyses confirmed that these factors remained independently significant in the model. Non-significant findings in the univariable analyses are provided in supplementary file 4. Having surgery in primary care and having a job with more than four hours of daily computer use were both associated with earlier return to work than their respective reference categories. Being female and having access to more than a month of paid sick leave were both associated with longer durations of work absence than their respected reference categories. Compared to those who expected to return within a week, there was a sequential increase in the likelihood of longer durations of work absence for those expecting to return between 7-14 days, 15-30 days and >30 days (Table 4), which showed a significant gradient effect ($p<0.001$). The assessment of R^2 indicated that 46% of variation in the duration of work absence was explained by the model ($R^2=0.46$, 95% CI 0.37-0.53).

For peer review only

TABLE 4. Cox proportional hazards analyses of the association between baseline demographic, clinical and occupational factors and the duration of work absence after carpal tunnel release

				Univariable analyses			Multivariable analysis		
	N	Median work absence (days)	Inter quartile range	Hazards ratio	95% Confidence intervals	P value	Hazards ratio	95% Confidence intervals	P value
Sex (censored: 5 females; no missing data)									
Male	72	17.5	8-31	1	-	-	1	-	-
Female	118	21.5	14-35	0.79	0.59, 1.06	0.12	0.56	0.36, 0.88	**0.01**
Age in years (censored: 1 aged 26-40, 3 aged 41-55, 1 aged >55; no missing data)									
26-40	21	20	15-30	1.01	0.63, 1.63	0.96	1.44	0.82, 2.55	0.21
41-55	94	20	9-33	1	-	-	1	-	-
>55	75	17	12-35	1.03	0.76, 1.40	0.83	1.15	0.80, 1.65	0.44
Smoking status (censored: 1 never, 4 current/ex; 2 missing)									
Never	105	16	12-31	1	-	-	1	-	-
Current/ex	83	23	13-41	0.74	0.56, 1.00	0.046	0.75	0.51, 1.09	0.13
Site (censored: 5 NHS secondary care; no missing data)									
NHS Primary care	72	19.5	13-33	1.18	0.87, 1.62	0.29	1.54	1.05, 2.25	**0.03**
NHS Secondary care	92	20	12-39.5	1	-	-	1	-	-
Private facilities	26	20	7-28	1.63	1.04, 2.54	0.03	1.46	0.87, 2.44	0.15
Afraid of long-term hand problems [a] *(censored: 1 no, 4 yes; 3 missing)*									
No	105	19	12-31	1	-	-	1	-	-
Yes	82	20.5	13-42	0.69	0.51, 0.93	0.01	0.93	0.67, 1.30	0.68
CTS-6 score for side of surgery (tertiles) [b] *(censored: 2 intermediate, 3 poor; 8 missing)*									
Good (1-3.0)	65	16	10-27	1	-	-	1	-	-
Intermediate (3.2-3.8)	58	21.5	14-35	0.77	0.54, 1.10	0.15	1.19	0.77, 1.84	0.44
Poor (3.8-5)	59	24	13-41	0.67	0.47, 0.97	0.03	1.04	0.65, 1.66	0.87
Type of work contract (censored: 5 employed, 1 missing)									
Employed (permanent)	154	22	14-38	1	-	-	1	-	-
Self-employed	30	13	6-19	1.72	1.13, 2.61	0.01	1.19	0.67, 2.14	0.55
Zero hours/temporary	5	12	3-31	2.01	0.81, 5.00	0.13	0.73	0.25, 2.14	0.56
Duration of available sick pay (censored: 4 >1 month, 1 unsure; no missing data)									
≤1 month	49	16	12-29	1	-	-	1	-	-
>1 month	88	27	15-42	0.59	0.41, 0.85	0.004	0.46	0.28, 0.76	**0.002**
Unsure	53	16	10-23	1.19	0.80, 1.77	0.40	1.01	0.61, 1.66	0.97
Access to occupational health at work (censored: 1 no, 4 yes; 1 missing)									
No	110	15.5	9-29	1.77	1.31, 2.38	<0.001	1.42	0.91, 2.19	0.12
Yes	79	25	16-42	1	-	-	1	-	-
Expected duration of leave after carpal tunnel release (days) (censored: 1 7-14, 2 15-29, 2 ≥30; no missing data)									
<7	35	4	2-12	1	-	-	1	-	-
7-14	75	16	13-26	0.23	0.15, 0.36	<0.001	0.27	0.16, 0.45	**<0.001**
15-29	35	29	22-39	0.12	0.07, 0.19	<0.001	0.19	0.10, 0.37	**<0.001**
≥30	45	42	21-44	0.08	0.05, 0.14	<0.001	0.12	0.06, 0.23	**<0.001**
MHQ work functioning score (tertiles) [c] *(censored: 3 poor, 2 intermediate; no missing data)*									
Poor (0-55)	67	20	13-35	0.68	0.47, 0.98	0.04	0.83	0.50, 1.40	0.49
Intermediate (60-80)	72	21	12.5-39.5	0.77	0.53, 1.10	0.15	0.81	0.50, 1.31	0.39
Good (81-100)	51	17	10-29	1	-	-	1	-	-
Job satisfaction [d] *(censored: 5 satisfied; 1 missing)*									
Satisfied	169	19	11-31	1	-	-	1	-	-
Dissatisfied	19	38	21-43	0.61	0.38, 0.99	0.04	0.67	0.38, 1.16	0.15
Believe that the hand problem was caused by work [e] *(censored: 1 no, 4 yes; 2 missing)*									
No	112	19	13-31	1	-	-	1	-	-
Agree/strongly agree	76	23	10-42	0.62	0.46, 0.85	0.003	0.82	0.57, 1.17	0.28
Job is demanding on hands/wrists [a] *(censored: 5 yes; no missing data)*									
No	35	16	6-27	1	-	-	1	-	-
Yes	155	20	13-38	0.61	0.42, 0.89	0.01	0.68	0.42, 1.12	0.13

For peer review only

Type of work [f] (censored: 2 non-manual, 3 manual; 1 missing)									
Non-manual	119	18	9-31	1	-	-	1	-	-
	70	23.5	14-41	0.66	0.48, 0.89	0.01	0.97	0.57, 1.64	0.90
Work involves target-driven pay [g] (censored: 3 no, 2 yes; 10 missing)									
No	149	19	12-31	1	-		1	-	-
Yes	31	22	13-45	0.61	0.41, 0.91	0.02	0.97	0.59, 1.61	0.91
Duration of computer use at work (hours) [g] (censored: 5 <1; 4 missing)									
<1	69	28	17-42	1	-	-	1	-	-
>1 to <4	33	16	10-31	2.20	1.43, 3.38	<0.001	1.01	0.56, 1.81	0.98
≥4	84	16	7-27	2.38	1.67, 3.38	<0.001	1.85	1.08, 3.16	**0.03**
Work involves lifting or carrying ≥10 kg [g] (censored: 4 no, 1 yes; 5 missing)									
No	108	18.5	11-30	1	-	-	1	-	-
Yes	77	24	13-40	0.61	0.42, 0.86	0.01	0.80	0.48, 1.33	0.39
Work involves pushing/pulling a heavy weight [g] (censored: 2 no, 3 yes; 2 missing)									
No	104	16	8.5-28.5	1	-	-	1	-	-
Yes	83	26	16-42	0.51	0.37, 0.70	<0.001	0.97	0.61, 1.55	0.90

Total sample size n=195. Median work absence relates to the 190 non-censored events. All analyses were adjusted for age and sex. All significant variables in the univariable analyses ($p<0.05$) were entered into the multivariable model.

[a.] Reported on a 0-10 scale, dichotomised to no (0-6) and yes (7-10)[13].

[b.] CTS-6 score [14] with data driven tertiles.

[c.] Michigan Hand Questionnaire work performance subscale scored from 0-100, higher scores indicate better perceived work performance[17]. Data driven tertiles.

[d.] Reported on a 5-point scale, dichotomised to satisfied (very satisfied/satisfied/fairly satisfied) and dissatisfied (dissatisfied/very dissatisfied).

[e.] Reported on a 5-point scale and dichotomised to agree/strongly agree and neither agree nor disagree/disagree/disagree strongly[22].

[f.] Classified using the Office for National Statistics Standard Occupational Classification 2010[18, 19].

[g.] As part of the normal working day[23].

For peer review only

Clinical outcomes after CTR are shown in Table 5. Using the definition outlined in the methods, a total of 46 participants (24%) were identified as having at least one poor outcome (CTS symptoms that were worse, unchanged or only slightly better; scar symptoms that were at least fairly troublesome; required post-operative antibiotics or had additional time off work after first return). Of these participants, the majority (n=38, 83%) reported only a single component of poor outcome. Three individuals defined as having a poor outcome had not returned to work at the point of last follow-up (as compared with two individuals in the rest of the study sample). For those who had returned to work, the median duration of work absence for those with a poor outcome was 22 days (IQR 12-42) compared with 19 days (IQR 12-32) for those without (Figure 2). This difference was not significant (Wilcoxon rank-sum, p=0.24).

TABLE 5. Clinical outcomes after carpal tunnel release

	**Mean score (standard deviation)**	
	Before surgery	12 weeks after surgery
CTS-6 (operated hand) [a]	3.3 (0.87)	1.2 (0.54)
MHQ function (operated hand) [b]	50 (22.1)	79 (19.4)
MHQ satisfaction with function (operated hand) [b]	38 (25.7)	82 (21.3)
MHQ bilateral activities of daily living [b]	69 (23.7)	88 (13.8)
MHQ activities of daily living (operated hand) [b]	65 (28.1)	87 (18.5)
	**Number of participants (%)**	
Global rating of change score		
Completely cured	-	65 (33.3)
Much better	-	98 (50.3)
Slightly better	-	13 (6.7)
Unchanged	-	2 (1.0)
Worse	-	5 (2.6)
Scar symptom severity	-	
Not at all troublesome	-	99 (50.8)
A little troublesome	-	63 (32.3)
Fairly troublesome	-	18 (9.2)
Very troublesome	-	2 (1.0)
Unbearable	-	0
Required post-operative antibiotics	-	10 (5.1)
Additional sick leave after first returning to work	-	12 (6.2)

Grey shading indicates the categories which were combined to define a poor surgical outcome.
[a.] CTS-6 assessment of carpal tunnel syndrome symptoms[14]. Range 1-5: 1 equals no symptoms.
[b.] MHQ Michigan Hand Questionnaire[17]. Range 0-100: 100 equals no deficit or dissatisfaction.

For peer review only - http://bmjopen.bmj.com/site/about/guidelines.xhtml

Figure 2. Duration of work absence after carpal tunnel release

There was no significant difference in the prevalence of a poor outcome among those who returned to work within or after seven days of CTR (20% versus 24%, Chi^2 $p=0.63$). Similarly, there was no significant difference in the prevalence of a poor outcome among those who returned to work within or after 14 days (19% versus 25%, Chi^2 $p=0.31$), or within or after 28 days of CTR (21% versus 27%, Chi2 p=0.33).

DISCUSSION

In this multi-site prospective cohort study, the median duration of work absence was 20 days (range 1-99), a duration similar to that reported by other European studies[24]. Earlier return to work was associated with typing for ≥4 hours at work (as compared with more physical workplace tasks) and undergoing surgery in primary care (as compared with secondary care or private practice). Pre-operative expectations about return to work were important significant predictors of actual return to work times. We found no evidence of poorer clinical outcomes in the first 12 weeks amongst those who returned to work earlier. At each time point, fewer manual workers had returned to work than non-manual workers and fewer employed workers had returned than self-employed (Figure 2). Both findings have been reported previously[10, 11, 25, 26], however, neither the type of work (manual/non-manual) nor the type of work contract (employed/self-employed) were significantly associated with the duration of work absence in the mutually adjusted model. These results illustrate the importance of considering the range of relevant demographic, clinical and occupational factors, which may have been confounders, moderators or mediators in previous studies. The reported model has not been developed to predict the duration of work absence for future CTR patients, rather to explore and identify important risk factors for consideration in future research.

For peer review only - http://bmjopen.bmj.com/site/about/guidelines.xhtml

Five variables remained statistically significantly associated with longer duration of work absence in the final model. Two were occupational factors: infrequent computer use and availability of sick pay. Cowan et al.[10], recorded earlier return to work after CTR for desk-based workers and we have shown previously that UK hand surgeons and hand therapists report that they advise earlier return to desk-based workers[6]. The relationship between longer duration of work absence and availability of sick pay has also be reported previously for those with and without worker's compensation[9, 24]. It is plausible that financial necessity is driving earlier return to work for those with limited sick pay. Alternatively, those with access to paid leave might choose to take the maximum available duration.

Only one clinical factor was significantly associated with the duration of work absence: participants who had their CTR surgery in primary care were more likely to return to work earlier than those whose procedure took place in an NHS hospital setting. One possible reason is that patients with more complex disease may be more commonly referred to secondary, rather than primary, care for their CTR and these patients may require longer off work after their surgery. However, in the current study the proportion and degree of comorbidities, and the extent of pre-operative symptoms reported by participants were similar across all settings. Another possibility is that the patients' expectations of the surgery may be different: CTR performed in a primary care setting might be perceived by patients as being more minor than surgery in a hospital operating theatre. Alternatively, the general practice surgeons may have recommended earlier return to work than those based in a hospital, although the median expected duration of work absence for participants in the current study was the same across all settings. The inclusion of CTR performed in primary care is a strength of the study. We

For peer review only - http://bmjopen.bmj.com/site/about/guidelines.xhtml

acknowledge that hand surgeons may also provide CTR services in primary care, as visiting clinicians, however in the current study this was not the case. CTR and other surgical procedures, such as vasectomy and minor skin surgery, are regularly performed by trained general practitioners in the UK[27], and all primary care surgeons in the current study were general practitioners who already provided a CTR service.

Only one demographic factor was statistically significant: women were more likely to take longer to return to work than men. Whilst we found inconsistent data about the effect of gender on return to work after CTR in the literature[28, 29], female gender has been associated with longer periods of work absence for other health conditions[30]. Researchers should continue to include sex as a covariate in analyses of return to work outcomes, although there is currently insufficient evidence to support any difference in return to work recommendations after CTR based on sex. Further qualitative investigation is required in order to understand the context for this.

Finally, those who expected to return to work more quickly were significantly more likely to do so. It has been shown previously that patient expectations are a prominent determinant of return to work time, or other return to work outcomes, for musculoskeletal or mental health conditions[30-32]. The role of expectations on outcomes, including the expected and actual timing for return to work and driving after hand and wrist surgery, requires further exploration, particularly because expectations are a potentially modifiable characteristic which could be influenced by the advice provided by clinicians pre-operatively.

For peer review only - http://bmjopen.bmj.com/site/about/guidelines.xhtml

In total, approximately a quarter of participants in this study were considered to have a poor outcome using our composite definition. We chose to use a multi-component definition which was deliberately very stringent to minimise the chances of missing any harm caused by early return to work. Our rates of poorer outcomes were in fact similar to those reported in other CTR populations[20, 33, 34]. Importantly, we found no relationship between earlier return to work and occurrence of poor outcomes within 12 weeks of CTR in this cohort study. We acknowledge that a longer follow-up duration would have aided the assessment of post-operative symptom resolution, however this was not possible with the resources available and was not a primary objective of the study.

There are a number of limitations of the current study, including the reliance on self-reported data. Work absence is not logged on a national database in the UK and therefore could only be obtained through self-report. To minimise errors of recall, date of return to work was determined contemporaneously. The recall duration for measures of function and symptoms was limited to a maximum of four weeks, consistent with the outcome measures used[14, 17]. We set out to recruit a large sample of working-aged adults undergoing CTR. Our prospectively recruited sample from 16 sites is one of the largest reported in the literature to date, with a good follow up response rate (79%), but it remains possible that we were under-powered to detect some of the factors which may have been associated with delayed return to work. Specifically, this could result where some levels of categorical variables of interest have lower prevalence, for example, the type of work contract (>80% of participants reported that they had a permanent work contract, compared with ~15% who were self-employed). Furthermore, we acknowledge that the inclusion of a large number of variables in the development of the final model may result in model overfitting, thereby potentially limiting generalisability.

For peer review only - http://bmjopen.bmj.com/site/about/guidelines.xhtml

We took the approach not to impute values where data were missing. Overall, the amount of missing data was small and at the individual item-level (Table 4 and supplementary file 4). Missing data were coded as such, and included in the analysis. We acknowledge that the approach taken to missing data may have resulted in biased estimates, yet if such effects are present, they are likely to be minimal due to low levels of missing data.

Following our *a priori* analysis plan, the association between each baseline variable and the duration of work absence was individually assessed in separate age- and sex-adjusted analyses. Only those variables which reached significance at the 5% level ($p<0.05$) were included in the multivariable model. In order to test the stability of our model, and to identify whether any potential associations had been missed, this was tested using 1% and 20% cut-offs. In both test scenarios, the findings were similar to those presented in our final model (Table 4), suggesting that our model is robust. However, we acknowledge that alternative methods of selecting variables for inclusion (such as forward inclusion or backward elimination) may have yielded slightly different results, particularly for variables that were close to our significance cut-off of 5%.

The findings may not be generalisable to working populations in regions outside of central and southern England and Wales, who are employed in other industries, or managed with a different CTR patient pathway. Steps were taken to include the main settings where CTR is performed in the UK, but we acknowledge that CTR may also be performed by other specialities. Individuals who chose to participate in the study may not be fully representative of the wider CTR population, and the observed differences between those who completed the study and those who were lost to follow-up (younger, poorer mental health, more likely

For peer review only - http://bmjopen.bmj.com/site/about/guidelines.xhtml

For peer review only

manual workers) also limit generalisability. Furthermore, we acknowledge that our model explained only 46% of variation in the duration of work absence.

Endoscopic CTR has been associated with earlier return to work than open CTR [35], however it was not possible to assess this in the current study. At present, endoscopic CTR is not routinely performed in the UK[6]. Anecdotally, most providers will not fund the extra cost of endoscopic CTR, which requires extra equipment, longer operating times and more experienced surgeons. Recruitment to the current study was not limited to patients undergoing open CTR, but no endoscopic procedures were performed during the study at any of our sites.

All participants were presumed to have CTS as diagnosed by their treating clinician. Many studies of CTS include nerve conduction study (NCS) findings as part of their eligibility criteria, although this was not possible in the current study because NCS are not routinely recommended for pre-operative diagnosis of CTS in the UK[2]. Our eligibility criteria required that only people undergoing their first CTR were included and reported upon in this study (so that previous experiences with CTR were not potential confounders). However, more than three quarters of the cohort reported bilateral symptoms. The possible impact of persisting CTS symptoms in the non-operated hand on return to work also needs to be considered.

For the current study, we considered both occupational title and self-reported occupational exposures collected in a standardised questionnaire format[13, 18, 19, 23]. Categorisation based on job title and industry may not accurately reflect the physical and/or psychosocial aspects of job role. Furthermore, co-occurrence of occupational exposures may be more

For peer review only - http://bmjopen.bmj.com/site/about/guidelines.xhtml

common in some types of jobs than in others, for example lifting >10kg and pushing or pulling a heavy weight.

There is a need for an agreed approach to identifying and recording key physical demands and psychosocial exposures of jobs to enable consistent exploration of their impact on work and clinical outcomes following surgery or other intervention. Approaches such as job exposure matrices[36] could facilitate this in future research.

In summary, this large multi-centre prospective cohort study investigated when participants return to work after CTR. Expectations about return to work (reported before surgery) were strongly associated with actual work absence, regardless of the job role or self-reported upper limb activities involved. Patient expectations can be influenced by many factors, but one of the most important is the advice provided by clinicians, in particular the surgeon. This suggests that clear, consistent advice could have an important effect on duration of sick leave. To date there is no evidence-based guidance informing clinicians what to advise about returning to different types of work after CTR. Further research is required to reach a consensus and explore whether the provision of targeted, consistent and standardised advice can alter the expected duration of work absence, reducing unnecessary sick leave, without causing adverse effects on clinical outcomes.

For peer review only

REFERENCES

1. Jerosch-Herold C, Mason R, Chojnowski AJ. A qualitative study of the experiences and expectations of surgery in patients with carpal tunnel syndrome. *J Hand Ther* 2008;21(1):54-61 doi:10.1197/j.jht.2007.09.001.
2. British Orthopaedic Association. Commissioning guide: Treatment of carpal tunnel syndrome. London: Royal College of Surgeons; 2017.
3. American Academy of Orthopaedic Surgeons. Management of carpal tunnel syndrome evidence-based clinical practice guideline. Illinois: American Academy of Orthopaedic Surgeons; 2016.
4. Bebbington E, Furniss D. Linear regression analysis of Hospital Episode Statistics predicts a large increase in demand for elective hand surgery in England. *J Plast Reconstr Aesthet Surg* 2015;68(2):243-51 doi:10.1016/j.bjps.2014.10.011.
5. Burton CL, Chen Y, Chesterton LS et al. Trends in the prevalence, incidence and surgical management of carpal tunnel syndrome between 1993 and 2013: an observational analysis of UK primary care records. *BMJ Open* 2018;8(6) doi:10.1136/bmjopen-2017-020166.
6. Newington L, Francis K, Ntani G et al. Return to work recommendations after carpal tunnel release: a survey of UK hand surgeons and hand therapists. *J Hand Surg Eur* 2018;43(8):875-78 doi:10.1177/1753193418786375.
7. Black C. Working for a healthier tomorrow. London, UK: Health Work and Wellbeing Programme 2008.
8. Peters S, Johnston V, Hines S et al. Prognostic factors for return-to-work following surgery for carpal tunnel syndrome: a systematic review. *JBI Database Syst Rev Implement Rep* 2016;14(9):135-216 doi:10.11124/jbisrir-2016-003099.
9. Dunn JC, Kusnezov NA, Koehler LR et al. Outcomes following carpal tunnel release in patients receiving workers' compensation: a systematic review. *Hand* 2018;13(2):137-42 doi:10.1177/1558944717701240.
10. Cowan J, Makanji H, Mudgal C et al. Determinants of return to work after carpal tunnel release. *J Hand Surg Am* 2012;37(1):18-27
11. De Kesel R, Donceel P, De Smet L. Factors influencing return to work after surgical treatment for carpal tunnel syndrome. *Occup Med* 2008;58(3):187-90

For peer review only

12. Parot-Schinkel E, Roquelaure Y, Ha C et al. Factors affecting return to work after carpal tunnel syndrome surgery in a large french cohort. *Arch of Phys Med Rehabil* 2011;92(11):1863-69 doi:http://dx.doi.org/10.1016/j.apmr.2011.06.001.

13. Hansen TB, Dalsgaard J, Meldgaard A et al. A prospective study of prognostic factors for duration of sick leave after endoscopic carpal tunnel release. *BMC Musculoskelet Disord* 2009;10:144

14. Atroshi I, Lyren PE, Ornstein E et al. The six-item CTS symptoms scale and palmar pain scale in carpal tunnel syndrome. *J Hand Surg Am* 2011;36(5):788-94 doi:10.1016/j.jhsa.2011.02.021.

15. Calfee RP, Dale AM, Ryan D et al. Performance of simplified scoring systems for hand diagrams in carpal tunnel syndrome screening. *J Hand Surg Am* 2012;37(1):10-17 doi:10.1016/j.jhsa.2011.08.016.

16. Katz JN, Stirrat CR. A self-administered hand diagram for the diagnosis of carpal tunnel. *J Hand Surg Am* 1990;15:360-63

17. University of Michigan Medical School. The Michigan Hand Questionnaire: http://mhq.lab.medicine.umich.edu/. Accessed 04/04/2019.

18. Office for National Statistics. Standard occupational classification for the UK: https://www.ons.gov.uk/methodology/classificationsandstandards/standardoccupationalclassificationsoc/soc2010. Accessed 08/06/2018.

19. Warwick Institute for Employment Research. Cascot: Computer assisted structured coding tool: https://warwick.ac.uk/fac/soc/ier/software/cascot/. Accessed 15/05/2019.

20. Jerosch-Herold C, Shepstone L, Houghton J et al. Prognostic factors for response to treatment by corticosteroid injection or surgery in carpal tunnel syndrome (PaLMS study): a prospective multi-centre cohort study. *Muscle Nerve* 2019;60(1):32-40 doi:doi:10.1002/mus.26459.

21. Ware J, Sherbourne C. The MOS 36-item short-form health survey (SF-36): I. conceptual framework and item selection. *Medical Care* 1992;30:473-83

22. Palmer K, Calnan M, Wainwright D et al. Upper limb pain in primary care: health beliefs, somatic distress, consulting and patient satisfaction. *Family Practice* 2006;23(6):609-17 doi:10.1093/fampra/cml047.

For peer review only

23. Karasek R, Brisson C, Kawakami N et al. The Job Content Questionnaire (JCQ): an instrument for internationally comparative assessments of psychosocial job characteristics. *Journal Occup Health Psychol* 1998;3(4):322-55
24. Newington L, Stevens M, Warwick et al. Sickness absence after carpal tunnel release: a systematic review of the literature. *Scand J Work Environ Health* 2018;44(6):557-67 doi:10.5271/sjweh.3762
25. Atroshi I, Larsson G-U, Ornstein E et al. Outcomes of endoscopic surgery compared with open surgery for carpal tunnel syndrome among employed patients: randomised controlled trial. *BMJ* 2006;332(7556):1473 doi:10.1136/bmj.38863.632789.1F.
26. Chaise F, Bellemere P, Fril JP et al. Return-to-work interval and surgery for carpal tunnel syndrome. Results of a prospective series of 233 patients. *J Hand Surg Br* 2004;29(6):568-70
27. Association of Surgeons in Primary Care. Surgical proceedures in primary care: https://www.aspc-uk.net/. Accessed 24/11/2020
28. Atroshi I, Zhou C, Joud A et al. Sickness absence from work among persons with new physician-diagnosed carpal tunnel syndrome: a population-based matched-cohort study. *PLoS One* 2015;10(3):e0119795 doi:10.1371/journal.pone.0119795.
29. Bekkelund SI, Pierre-Jerome C, Torbergsen T et al. Impact of occupational variables in carpal tunnel syndrome. *Acta Neurol Scand* 2001;103(3):193-97
30. Cancelliere C, Donovan J, Stochkendahl MJ et al. Factors affecting return to work after injury or illness: best evidence synthesis of systematic reviews. *Chiropr Man Ther* 2016;24(1):32-32 doi:10.1186/s12998-016-0113-z.
31. Claréus B, Renström EA. Patients' return-to-work expectancy relates to their beliefs about their physician's opinion regarding return to work volition and ability. *J Pain Res* 2019;12:353-62 doi:10.2147/JPR.S179061.
32. Heymans MW, de Vet HC, Knol DL et al. Workers' beliefs and expectations affect return to work over 12 months. *Journal Occup Rehabil* 2006;16(4):685-95 doi:10.1007/s10926-006-9058-8.
33. Bland JDP. Treatment of carpal tunnel syndrome. *Muscle Nerve* 2007;36(2):167-71 doi:10.1002/mus.20802.
34. Louie D, Earp B, Blazar P. Long-term outcomes of carpal tunnel release: a critical review of the literature. *Hand* 2012;7(3):242-46 doi:10.1007/s11552-012-9429-x.

For peer review only

35. Li Y, Luo W, Wu G et al. Open versus endoscopic carpal tunnel release: a systematic review and meta-analysis of randomized controlled trials. *BMC Musculoskelet Disord* 2020;21:272 doi:10.1186/s12891-020-03306-1

36. Dale AM, Zeringue A, Harris-Adamson C et al. General population job exposure matrix applied to a pooled study of prevalent carpal tunnel syndrome. *Am Journal Epidemiol* 2015;181(6):431-39

Conflicts of interest

LN was supported by a National Institute for Health Research (NIHR) Doctoral Research Fellowship, grant number DRF-2015-08-056. GN was supported by funding from the MRC Versus Arthritis Centre for Musculoskeletal Health and Work. The authors declare no other conflicts of interest.

Funding

LN was supported by a National Institute for Health Research (NIHR) Doctoral Research Fellowship, grant number DRF-2015-08-056. GN was supported by funding from the MRC Versus Arthritis Centre for Musculoskeletal Health and Work. The views expressed are those of the authors and not necessarily those of the funders, the NHS, the NIHR or the Department of Health.

Informed consent

Written informed consent was obtained from the patient(s) for their anonymised information to be published in this article.

Ethics approval

For peer review only

Full ethics approval was granted by the NHS Health Research Authority (IRAS 209840: 16/WA/0390) and University of Southampton (ERGO 25757) Ethics Committees.

Contributorship

The study was devised by LN, KWB, JA, DW and DC. LN completed data collection and analysis, with assistance from GN and KWB. All authors contributed to the interpretation of the data. LN wrote the first draft of the manuscript. All authors reviewed and edited the manuscript and approved the final version.

Acknowledgements

The authors would like to thank and acknowledge all the study participants who gave up their time to take part in this study. Thank you also to the Patient Advisory Group for their contribution to the development of the research question and study material, and their thoughtful feedback on the preliminary findings. Thank you to Prof David Coggon for his assistance with the initial research idea, and to the Association of Surgeons in Primary Care and British Society for Surgery of the Hand for supporting the study.

Thank you to the study sites and local principal investigators: Jeremy Bland at East Kent Hospitals University NHS Foundation Trust; Darren Roberts at Portsmouth Hospitals NHS Trust; Mike Taylor and Claire Zweifel at Mid Essex Hospital Services NHS Trust; Frances Clark at Tollgate Clinic; Will Mason at Gloucester Hospitals NHS Foundation Trust; Vasileios Kefalas and Petros Mikalef at Care UK Southampton NHS Treatment Centre; Peter Sharpe at Salisbury Medical Practice; David Warwick at University Hospital Southampton NHS Foundation Trust and Nuffield Health Wessex Hospital; Alistair Phillips at Southern Health NHS Foundation Trust; Nanda Pillai at St Luke's Surgery, Walsall; Tim Halsey and Effie Katsarma at Chelsea and

For peer review only

Westminster Hospital NHS Foundation Trust; Claire Middleton at Royal Berkshire NHS Foundation Trust; Duncan Avis and Ian MacLeod at Hampshire Hospital NHS Foundation Trust; Nick Gape and Lisa Small at Cardiff and Vale University Health Board; Kristin Francis at HCA Healthcare London Hand and Wrist Unit; and to all local team members who helped set up and recruit to the REACTS study.

Data availability statement

Data are available on reasonable request. Deidentified participant data are available from the Data Manager, MRC Lifecourse Epidemiology Unit, University of Southampton (email: mrcleu@mrc.soton.ac.uk). The study protocol and statistical analysis plans are available from: https://eprints.soton.ac.uk/438720/.

For peer review only

Figure 1. Participant numbers for each stage of the cohort study

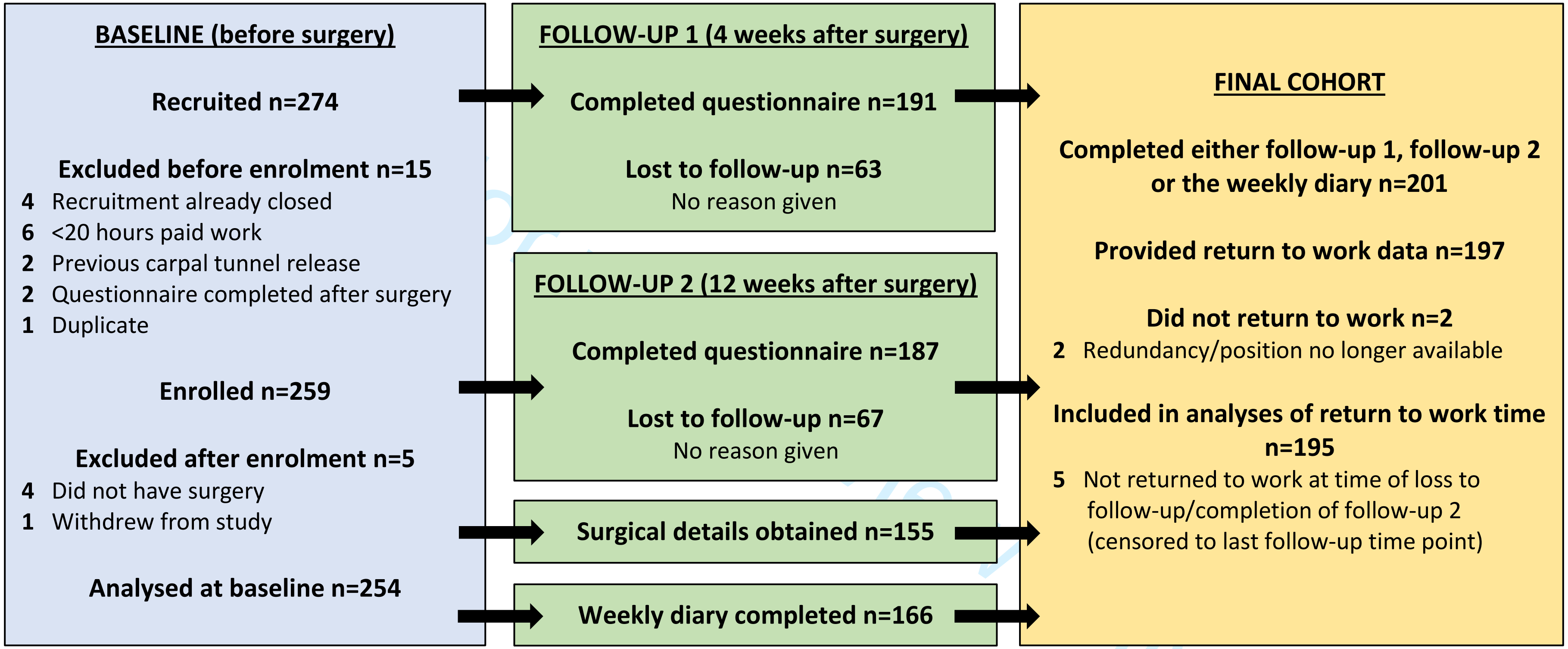

For peer review only - http://bmjopen.bmj.com/site/about/guidelines.xhtml

Figure 2. Duration of work absence after carpal tunnel release

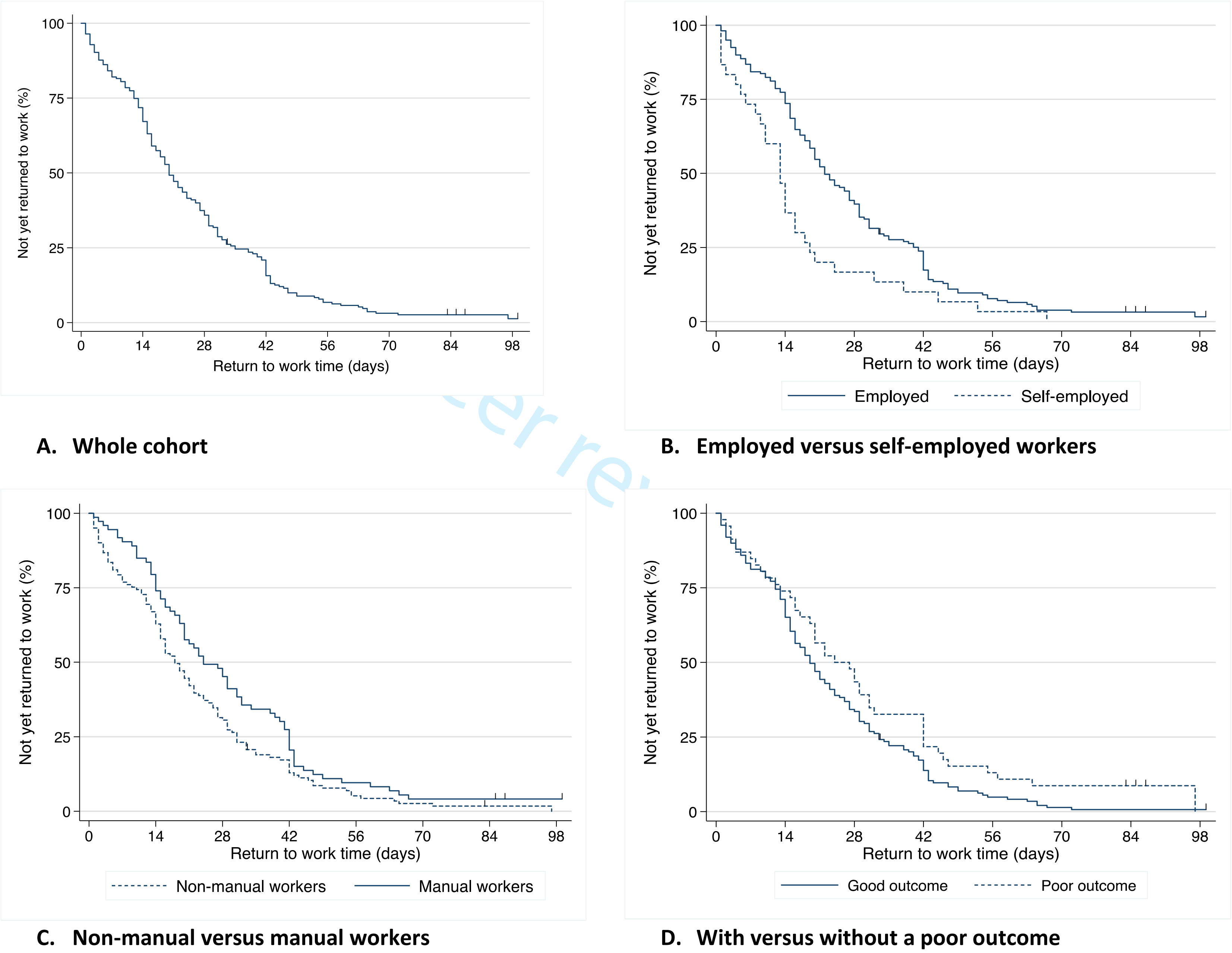

Vertical lines indicate right censoring for those who had not returned to work at the point of last follow-up

Development of the REACTS study questionnaire: Reasoning for item inclusion

Questions were chosen to capture information on variables that might be expected to influence return to work after CTR.

Demographic factors

General demographic information was requested including date of birth, sex and hand dominance. Other studies have found that older age was associated with poorer work outcomes after CTR [1, 2], while no clear sex effect has been shown [3, 4]. Hand dominance in relation to side of surgery is rarely reported in CTR studies, however, surgery to the non-dominant hand has been linked to quicker and more complete resolution of CTS symptoms [5].

Carpal tunnel release planning

Information was collected on the expected date of CTR, side of surgery, availability of occupational health services and the patient's expectations about time off work post-surgery. Expected duration of work absence has been identified as a determinant of return to work time in previous CTR studies [2, 6].

Occupational factors

Participants were asked to list their main occupation and the industry in which they work (examples were provided to facilitate the response). This information was processed using the UK Office for National Statistics Standard Occupational Classification [7] and Computer Assisted Structured Coding tool (Cascot) [8] to generate manual and non-manual categories. Cases where the coding match was confirmed as less than 64% were reviewed by the lead researcher and coded by hand [8]. This was checked by the department data manager and any queries resolved through discussion.

Participants were asked to categorise their employment type as: employed (permanent contract), employed (temporary/renewable contract), zero hours contract and self-employed. Our systematic review found earlier return to work times for self-employed individuals compared to those who were employed [9]; but this was only investigated in two

For peer review only - http://bmjopen.bmj.com/site/about/guidelines.xhtml

studies [10, 11]. The additional sub-categories for employment type listed above and a separate question about sick-pay entitlement were included to allow the impact of work contract type to be explored [12]. Participants were also asked how many hours they usually worked each week and over how many days; this information was collected for the participant's main job and any other routine paid work. The total number of work hours per week was calculated by combining the hours for main and additional jobs.

Occupational activities that load the upper limb and potential work stressors were asked as a series of yes/no questions following the format of a recent multi-centre RCT exploring management of non-specific distal arm pain [13]. These questions originated in the Job Content Questionnaire, designed to assess psychological and physical aspects of work [14]. Activities included: computer use, tasks involving repeated wrist/finger movement, holding vibrating tools, lifting more than 5 or 10kgs, pushing/pulling a heavy weight, working with the neck flexed or rotated, and driving. Our systematic review found that manual workers took longer to return to work than non-manual workers [9] and these questions were used to determine the self-reported level of upper limb manual activity involved in each participant's job.

Potential psychosocial work stressors were also assessed. These included piecemeal work, activity targets and bonuses, and tight deadlines. As the first three items all concerned payment for results, these were combined for the analyses. Participants were also asked whether they found their main job demanding on their hands/wrist and whether their boss/colleagues were supportive. Both questions were scored on a 0-10 scale as reported previously in a study of sick leave duration after endoscopic CTR [15]. These were dichotomised as supportive (7-10) and neutral/unsupportive (0-6). A question about general job satisfaction was also included later in this section of the questionnaire, with the Likert response options: very satisfied, satisfied/fairly satisfied, dissatisfied and very dissatisfied [15, 16]. The last two options were condensed to give three categories for the analyses.

To assess self-reported work function, participants were asked to complete the work performance section of the Michigan Hand Questionnaire (MHQ) [17]. This patient reported outcome measure is frequently used in upper limb clinical practice and research and has

For peer review only - http://bmjopen.bmj.com/site/about/guidelines.xhtml

been validated for use with CTS and CTR populations [18]. Permission was granted for the MHQ to be used in the study. The questionnaire asked participants to recall how much difficulty they had with general work tasks over the past four weeks in relation to problems with their hands/wrists, for example: needing to shorten their working day, taking longer to complete tasks or needing to take breaks. Using the standard scoring, each question was completed on a Likert scale of: always, often, sometimes, rarely and never, and combined to give a score from 0-100, with 100 representing no problems with work functioning [19]. Participants were also asked whether they had taken any periods of sickness absence from work over the previous four weeks, both related to the hand/wrist problem, or for any other problem.

General health

Seven general health questions were included to capture information on comorbidities, physical and mental health and somatisation. Self-reported health was assessed using the first SF-36 question: In general, would you say your health is – excellent, very good, good, fair, poor [20]. This was taken from the original SF-36 version, which is free from licence charges and was dichotomised as excellent/very good/good and fair/poor for the analyses.

Participants were asked their height and weight to enable the calculation of BMI (body mass index; weight in kilograms/height in metres squared). This was categorised using standard WHO classification: underweight (BMI <18.5), normal weight (BMI 18.5-24.9), overweight (BMI 25.0-29.9) and obese (BMI ≥30.0) [21]. Smoking status was categorised as those who have never smoked regularly, those who have smoked in the past and those who regularly smoke, with the latter two categories combined for the analyses. Previous studies have found that obesity (BMI ≥30) was linked to poorer work outcomes [1] and smoking was linked to poorer clinical outcomes after CTR [22].

A list of common health problems and their impact on general activities was assessed using the Self-Administered Comorbidity Questionnaire [23]. Participants were asked first to select whether they have any of the 14 medical conditions and if so whether this limited their activities. All medical conditions were worded in an accessible format, as evaluated by the patient advisory group. Responses were analysed as the number of comorbidities and

For peer review only - http://bmjopen.bmj.com/site/about/guidelines.xhtml

For peer review only

the number of disabling comorbidities using the scale: 0, 1, ≥2. Mental health was assessed using the mental health and vitality questions from the licence-free version of the SF-36 [20]. One modification was made to change the wording of the question 'Did you feel full of pep?' to 'Did you feel full of get-up-and-go?' for the UK rather than US setting. The questions were used to calculate the summary score from 0-100 (where 100 represents no disability).

Somatisation was assessed using a subset of five questions from the Four-Dimensional Symptom Questionnaire [24] as previously reported in UK cohort studies of health and employment [25] and upper limb pain in primary care [26]. The number of symptoms that were rated by the participant as at least moderately distressing were used to create the analysis categories of: 0, 1, ≥2 symptoms.

Hand and wrist symptoms and function

Katz & Stirrat hand diagrams were included for the participants to indicate where on their hand(s) they experience pain and/or tingling and numbness [27]. This self-administered tool can be used clinically as part of the CTS diagnosis process using the scoring system modified by Calfee et al. [28]. A question on symptom duration was also included and categorised as less than 3 months, 3-6 months, 6-12 months and more than a year. This was dichotomised to ≤1 year and >1 year for analysis. All participants were expected to have clinically diagnosed CTS as they were undergoing CTR, but participants were asked to answer for both hands, so that symptoms in the non-operated hand were also assessed. This was used to define bilateral or unilateral symptoms. Hand diagram scores were dichotomised according to a stringent definition of CTS (classic and probable) and unlikely CTS (possible and unlikely).

The CTS-6 questionnaire was included to assess the severity of CTS symptoms [29]. This tool is a shorter version of the Boston Carpal Tunnel Questionnaire [30] and has been assessed for use pre- and post CTR surgery. The six questions explore the severity of pain and numbness, whether this occurs during the daytime or at night, and whether this wakes the individual. Participants were asked to complete these questions separately for each hand using a 5-point Likert scale. Using the standard scoring criteria, responses for each item

For peer review only - http://bmjopen.bmj.com/site/about/guidelines.xhtml

For peer review only

were combined to give a mean score ranging from 1-5, with 5 representing the highest severity of symptoms. If one response was missing (per hand) this was imputed using the mean score of the remaining responses [29].

Hand function was assessed using the MHQ sub-sections on unilateral hand function (asked for each hand), satisfaction with hand function (asked for each hand), and ability to perform unilateral and bilateral activities of daily living (ADLs) [17]. The MHQ summary question relating to the level of satisfaction with the appearance of each hand was also included. All questions were scored on the 5-point Likert scales provided and used the standard wording and scoring to enable comparison with other study populations. Possible scores for each sub-section range from 1-100, with 100 representing no problems or the highest level of satisfaction. Missing data were imputed according to the MHQ guidelines, which allow the scale to be calculated if more than 50% of the questions for each sub-section have been completed [19].

Health beliefs

The remaining questions related to health beliefs. Beliefs about the cause of symptoms and likely prognosis have been identified as key themes in health-seeking behaviour for CTS [31] and upper limb pain [26], and blaming oneself for the hand problem has been associated with long durations of sick leave after endoscopic CTR [15]. The participant's expectations for being able to use the affected hand normally within 3 months of surgery, fear of long-term hand problems, blaming oneself for the hand problem and the perceived level of support available from friends and family were assessed. Responses were rated on a 0-10 scale as reported by Hansen et al. [15]. All responses were converted to a unidirectional scale with 10 being the best outcome, and were dichotomised as neutral/negative response 0-6 and positive response 7-10.

Participants were also asked to agree/disagree (via a 5-point Likert scale) with a series of seven questions about the believed cause of their symptoms. Using previously reported methods, the responses were dichotomised to those who agreed (agree/strongly agree) and those who did not agree (neither agree nor disagree/disagree/disagree strongly) with each statement [26]. The first two questions were combined to generate six items: 1) I think I was

For peer review only - http://bmjopen.bmj.com/site/about/guidelines.xhtml

born with a weakness in this part of my body/problems like this run in my family; 2) my problem was caused by work; 3) my problem probably wasn't caused by work, but work made it worse; 4) I have a lot of stress in my life and that has made my problem a lot worse; 5) a lack of exercise probably contributed to my problem; 6) as you get older, parts of the body wear out and problems like mine are likely [26].

Finally, the abbreviated Pain Catastrophizing Scale was included, which provides insight about the participant's pain beliefs [32]. Responses were dichotomised to those who reported catastrophising pain thoughts and feelings to at least a moderate degree in response to all questions, and those who did not.

References

1. Parot-Schinkel E, Roquelaure Y, Ha C et al. Factors affecting return to work after carpal tunnel syndrome surgery in a large french cohort. *Arch of Phys Med Rehabil* 2011;92(11):1863-69.
2. Peters S, Johnston V, Hines S et al. Prognostic factors for return-to-work following surgery for carpal tunnel syndrome: a systematic review. *JBI Database of Systematic Reviews and Implementation Reports* 2016;14(9):135-216.
3. Bekkelund SI, Pierre-Jerome C, Torbergsen T et al. Impact of occupational variables in carpal tunnel syndrome. *Acta Neurol Scand* 2001;103(3):193-97.
4. Atroshi I, Zhou C, Joud A et al. Sickness absence from work among persons with new physician-diagnosed carpal tunnel syndrome: a population-based matched-cohort study. *PLoS One* 2015;10(3):e0119795.
5. Tang Q, Lai W, Tay S. The effect of hand dominance on patient-reported outcomes of carpal tunnel release in patients with bilateral carpal tunnel syndrome. *Journal of Hand Surgey Asian Pacific Volume* 2017;22(3):303-08.
6. Cowan J, Makanji H, Mudgal C et al. Determinants of return to work after carpal tunnel release. *J Hand Surg Am* 2012;37(1):18-27.
7. Office for National Statistics. Standard occupational classification for the UK: https://www.ons.gov.uk/methodology/classificationsandstandards/standardoccupationalclassificationsoc/soc2010. Accessed 08/06/2018.
8. Warwick Institute for Employment Research. Cascot: Computer assisted structured coding tool https://warwick.ac.uk/fac/soc/ier/software/cascot/. Accessed 15/05/2019.
9. Newington L, Stevens M, Warwick D et al. Sickness absence after carpal tunnel release: a systematic review. *Scandinavian Journal of Work, Environment and Health* 2018;44(6):557-67.
10. De Kesel R, Donceel P, De Smet L. Factors influencing return to work after surgical treatment for carpal tunnel syndrome. *Occup Med* 2008;58(3):187-90.

For peer review only - http://bmjopen.bmj.com/site/about/guidelines.xhtml

11. Chaise F, Bellemere P, Fril JP et al. Return-to-work interval and surgery for carpal tunnel syndrome. Results of a prospective series of 233 patients. *J Hand Surg Br* 2004;29(6):568-70.
12. Tran M, Sokas RK. The gig economy and contingent work: an occupational health assessment. *Journal of Occupational and Environmental Medicine* 2017;59(4):E63-E66.
13. Jones GT, Mertens K, Macfarlane GJ et al. Maintained physical activity and physiotherapy in the management of distal upper limb pain - a protocol for a randomised controlled trial (the arm pain trial). *BMC Musculoskelet Disord* 2014;15:71.
14. Karasek R, Brisson C, Kawakami N et al. The Job Content Questionnaire (JCQ): an instrument for internationally comparative assessments of psychosocial job characteristics. *Journal of occupational health psychology* 1998;3(4):322-55.
15. Hansen TB, Dalsgaard J, Meldgaard A et al. A prospective study of prognostic factors for duration of sick leave after endoscopic carpal tunnel release. *BMC Musculoskelet Disord* 2009;10:144.
16. D'Angelo S, Coggon D, Harris EC et al. Job dissatisfaction and the older worker: baseline findings from the Health and Employment After Fifty study. *Occupational and environmental medicine* 2016;73(8):512-19.
17. Chung KC, Pillsbury MS, Walters MR et al. Reliability and validity testing of the Michigan Hand Outcomes Questionnaire. *J Hand Surg Am* 1998;23(4):575-87.
18. Hadi M, Gibbons E, Fitzpatrick R. A structured review of patient-reported outcome measures for procedures for carpal tunnel syndrome. UK: Patient-Reported Outcome Measures Group, University of Oxford; 2011.
19. University of Michigan Medical School. The Michigan Hand Questionnaire: http://mhq.lab.medicine.umich.edu/. Accessed 04/04/2019.
20. Ware J, Sherbourne C. The MOS 36-item short-form health survey (SF-36): I. conceptual framework and item selection. *Medical Care* 1992;30:473-83
21. World Health Organization. Obesity: preventing and managing the global epidemic. Report of a WHO consultation. *World Health Organization technical report series* 2000;894:1-253.
22. Dahlin E, Zimmerman M, Bjorkman A et al. Impact of smoking and preoperative electrophysiology on outcome after open carpal tunnel release. *Journal of Plastic Surgery & Hand Surgery* 2017;51(5):329-35.
23. Sangha O, Stucki G, Liang MH et al. The self-administered comorbidity questionnaire: a new method to assess comorbidity for clinical and health services research *Arthritis & Rheumatism* 2003;49(2):156-63.
24. Terluin B, van Marwijk HW, Adèr HJ et al. The Four-Dimensional Symptom Questionnaire (4DSQ): a validation study of a multidimensional self-report questionnaire to assess distress, depression, anxiety and somatization. *BMC Psychiatry* 2006;6(1):34.
25. Palmer KT, Walker-Bone K, Harris EC et al. Health and Employment after Fifty (HEAF): a new prospective cohort study. *BMC public health* 2015;15:1071.
26. Palmer K, Calnan M, Wainwright D et al. Upper limb pain in primary care: health beliefs, somatic distress, consulting and patient satisfaction. *Family Practice* 2006;23(6):609-17.

For peer review only - http://bmjopen.bmj.com/site/about/guidelines.xhtml

27. Katz JN, Stirrat CR. A self-administered hand diagram for the diagnosis of carpal tunnel. *J Hand Surg Am* 1990;15:360-63.
28. Calfee RP, Dale AM, Ryan D et al. Performance of simplified scoring systems for hand diagrams in carpal tunnel syndrome screening. *J Hand Surg Am* 2012;37(1):10-17.
29. Atroshi I, Lyren PE, Ornstein E et al. The six-item CTS symptoms scale and palmar pain scale in carpal tunnel syndrome. *J Hand Surg Am* 2011;36(5):788-94.
30. Levine DW, Simmons BP, Koris MJ et al. A self-administered questionnaire for the assessment of severity of symptoms and functional status in carpal tunnel syndrome. *J Bone Joint Surg Am* 1993;75:1585-92.
31. Martin H. Patients' health beliefs and adaptation to carpal tunnel syndrome based on duration of symptomatic presentation. *J Hand Ther* 2007;20(1):29-35.
32. Bot AG, Becker SJ, Bruijnzeel H et al. Creation of the abbreviated measures of the Pain Catastrophizing Scale and the Short Health Anxiety Inventory: the PCS-4 and SHAI-5. *Journal of Musculoskeletal Pain* 2014;22(2):145-51.

For peer review only

University Hospital Southampton NHS Foundation Trust | REACTS | Arthritis Research UK MRC centre for musculoskeletal health & work | UNIVERSITY OF Southampton

Site

Please fill in today's date

d d m m y y

Before Your Carpal Tunnel Release Surgery

REACTS

Return to Employment After Carpal Tunnel Release Surgery (REACTS)

ln@mrc.soton.ac.uk | 023 8077 7624
Arthritis Research UK – MRC Centre for Musculoskeletal Health and Work
MRC Lifecourse Epidemiology Unit, University of Southampton
Southampton General Hosptial (MP 95), SO16 6YD

IRAS reference: 209840

For peer review only

University Hospital Southampton NHS Foundation Trust
REACTS
Arthritis Research UK MRC centre for musculoskeletal health & work
University of Southampton

CONSENT FORM (IRAS reference: 209840)

You should complete this form after you have read the Participant Information Sheet.

REACTS: Return to employment after carpal tunnel release surgery

Thank you for considering taking part in this research. If you have any questions arising from the Participant Information Sheet, please ask the research team before you decide whether to take part.

Please initial the boxes if you agree with each statement

1. I have read the Participant Information Sheet (version 2.0; 06.12.16) and have had the opportunity to ask questions about the study. ☐

2. I meet the criteria for being involved in this study: ☐
   - Aged over 18 and referred for carpal tunnel release surgery
   - Routinely work in paid employment for at least 20 hours per week
   - Plan to return to work after carpal tunnel release surgery
   - Have not previously had carpal tunnel release surgery on either hand
   - Have not previously had a serious injury to the same wrist/hand that will have the carpal tunnel release operation

3. I agree to take part in this research and agree for my data to be used for the purposes explained in the Participant Information Sheet (version 2.0; 06.12.16). I understand that this information will be handled in accordance with the terms of the UK Data Protection Act 1998. ☐
   a. I agree for the REACTS research team to access pre-operative test results concerning my hand and wrist symptoms. No other information will be accessed. ☐
   b. I agree for the REACTS research team to access my carpal tunnel release surgical record. No other information will be accessed. ☐

4. I understand that if I decide at any time during the research that I no longer wish to take part, I can notify the researchers and withdraw from the study immediately, without giving a reason. If I do, I understand that I can ask for any contribution I have already made to be removed from the study, up to the time when I have completed the final questionnaire. ☐

Signature ______________________ Date ____ / ____ / ____

Name ______________________ Phone ______________________
(please print) *(only to be used if we lose touch)*

Postal address ______________________

____

Email address ______________________
(please print)

For peer review only

University Hospital Southampton NHS Foundation Trust | REACTS | Arthritis Research UK MRC centre for musculoskeletal health & work | UNIVERSITY OF Southampton

ADDITIONAL QUESTIONS

Please circle one response for each question

I prefer to receive the next two questionnaires by	**Post**	**Email**	**Don't mind**
I prefer to receive correspondence about the study by	**Post**	**Email**	**Don't mind**
I would like to be notified of the findings from this research	**Yes**	**No**	
I am happy to be contacted about the next stage of the research, which will involve a one-off discussion with the lead researcher	**Yes**	**No**	
I am happy to be contacted about other studies related to this research	**Yes**	**No**	

When the research team receives your completed questionnaire and consent form, we will sign it below and return a copy to you for your records.

Researcher signature ____ Date ____ / ____ / ____

Researcher name ____

University of Southampton research supervisors:
Professor Karen Walker-Bone | Professor Jo Adams | Professor David Warwick

For peer review only

SECTION A: BACKGROUND

1 What is your date of birth?

d d m m y y

2 Are you:

Male ☐ Female ☐ Other ☐

3 Are you:

Right handed ☐ Left handed ☐ Both ☐

4 Do you routinely carry out paid work for 20 hours or longer in a given week?

Yes ☐ No ☐

If no, *thank you for your interest in our study, however, we are only looking for individuals who carry out paid work for at least 20 hours per week. You* ***do not*** *need to complete the rest of the questionnaire, but please return it using the pre-paid envelope provided.*

5 When do you expect to have your carpal tunnel surgery?
Please enter the exact date if known, or provide the approximate month and year if unsure.

d d m m y y

6 Which hand will be operated on?
If both hands please answer Question 6.1; if one hand, please move on to Question 7.

Right ☐ Left ☐ Both ☐

6.1 If both hands, which side will be operated on first?

Right ☐ Left ☐ Both sides operated on the same day ☐ Unsure ☐

7 Do you have access to an occupational health service through your place of work?

Yes ☐ No ☐ Unsure ☐

8 Do you expect to take any time off work following your surgery?
If yes, please answer Question 8.1; if no, please move on to Question 9.

Yes ☐ No ☐ Unsure ☐

8.1 If you do expect to take time off work, how long do you expect to take?
Please complete using days, weeks or months; whichever applies.

Days ☐ Weeks ☐ Months ☐

For peer review only - http://bmjopen.bmj.com/site/about/guidelines.xhtml

SECTION A: BACKGROUND

9 Have you been given any information about your operation?
If yes, please answer Question 9.1; if no, please move on to Question 10.

Yes ☐ No ☐

9.1 If yes, who provided this information? Please tick all that apply.

a) Your surgeon or a member of the surgical team ☐
b) Hospital nurse ☐
c) GP or practice nurse ☐
d) Hand therapist ☐
e) Physiotherapist or occupational therapist ☐
f) Occupational health nurse or doctor ☐
g) Employer ☐
h) Friend or family member ☐
i) Internet ☐
j) Other *(please specify)* ☐

....

10 Have you been given any information about returning to work after your surgery?
If yes, please answer the rest of Question 10; if no, please move on to Question 11.

Yes ☐ No ☐

10.1 If yes, who provided this information? Please tick all that apply.

a) Your surgeon or a member of the surgical team ☐
b) Hospital nurse ☐
c) GP or practice nurse ☐
d) Hand therapist ☐
e) Physiotherapist or occupational therapist ☐
f) Occupational health nurse or doctor ☐
g) Employer ☐
h) Friend or family member ☐
i) Internet ☐
j) Other *(please specify)* ☐

....

10.2 What advice were you given?
If this advice came from more than one source, please indicate who advised what.

....
....
....
....
....
....
....

For peer review only - http://bmjopen.bmj.com/site/about/guidelines.xhtml

SECTION B: WORK

12 **What is your MAIN occupation at the moment (e.g. secretary, teacher, builder etc.)?**

----

13 **And in what industry do you work (e.g. farming, shipyard, car factory, shoe shop, hospital, insurance office etc)?**

----

14 **Which of the following best describes your present work situation for your MAIN occupation?** Please tick one box.

a) Employed (permanent contract) ☐
b) Employed (temporary/renewable contract) ☐
c) Zero hours contract ☐
d) Self-employed ☐
e) Other *(please specify)* ☐

----

15 **On average, how many *hours* per week do you normally work in your main occupation?**

☐☐☐ hours

16 **On average, how many *days* per week do you normally work in your main occupation?**

☐ days

17 **Do you have any other paid work?**
If yes, please answer Question 17.1; if no, please move on to Question 18.

Yes ☐ No ☐

17.1 **If yes, on average, how many hours a week do you work in other paid jobs?** ☐☐ hours

18 **Does an average day at work in your MAIN job normally involve any of the following?** Please tick one box for each question.

		Yes	No
a)	Piecework in which you are paid according to the number of articles or tasks you or your team make or finish in the day?	☐	☐
b)	A target number of articles or tasks that you or your team are expected to make or finish in the day?	☐	☐
c)	Payment of a bonus if you make or finish more than an agreed number of articles/tasks in the day?	☐	☐
d)	Working to tight deadlines	☐	☐
e)	Use of a computer keyboard or mouse for longer than 1 hour in total?	☐	☐

For peer review only

SECTION B: WORK

		Yes	No
f)	Use of a computer keyboard or mouse for longer than 4 hours in total?	☐	☐
g)	Other tasks involving repeated movements of the wrist or fingers for longer than 4 hours in total? *(Please indicate which tasks)*	☐	☐

		Yes	No
h)	Working with a powered tool that makes your hand(s) or arm(s) vibrate (e.g. chain saw, pneumatic drill)?	☐	☐
i)	Working with your hand(s) above shoulder height for longer than 1 hour in total?	☐	☐
j)	Lifting or carrying weights of 5 kg (11 lbs) or more in one hand (e.g. a tool bag or heavy briefcase)?	☐	☐
k)	Lifting or carrying a weight of 10 kg (22 lbs) or more?	☐	☐
l)	Tasks involving pushing or pulling a heavy weight?	☐	☐
m)	Working for longer than two hours in total with your neck bent forward?	☐	☐
n)	Working for longer than half an hour in total with your neck twisted e.g. when looking to one side?	☐	☐
o)	Driving for more than an hour?	☐	☐

19 Do you find your MAIN job demanding on your hands/wrists?
Please circle one number, where 0 represents not at all, and 10 represents very much.

0 1 2 3 4 5 6 7 8 9 10

Not at all Very much

20 Does your MAIN employer (or boss/colleagues if self-employed) know about your hand/wrist problem?
If yes, please answer Question 20.1; if no, or not applicable, please move on to Question 21.

Yes ☐ No ☐ N/A self-employed and work alone ☐

20.1 Is your MAIN employer (or boss/colleagues if self-employed) supportive of your hand/wrist problem?
Please circle one number, where 0 represents not at all, and 10 represents very much

0 1 2 3 4 5 6 7 8 9 10

Not at all Very much

For peer review only - http://bmjopen.bmj.com/site/about/guidelines.xhtml

SECTION B: WORK

21 **The following questions refer to how you did in your MAIN job during the *past 4 weeks*.**
Please tick one box for each question.

	How much of the time during the past 4 weeks …	**Always**	**Often**	**Sometimes**	**Rarely**	**Never**
a)	Were you unable to do your work because of problems with your hand(s) / wrist(s)?	☐	☐	☐	☐	☐
b)	Did you have to shorten your work day because of problems with your hand(s) / wrists(s)	☐	☐	☐	☐	☐
c)	Did you have to take breaks at work because of problems with your hand(s) / wrists(s)?	☐	☐	☐	☐	☐
d)	Did you get less done because of problems with your hand(s) / wrist(s)?	☐	☐	☐	☐	☐
e)	Did you take longer to do the tasks in your work because of problems with your hand(s) / wrists(s)?	☐	☐	☐	☐	☐

22 **During the *past 4 weeks*, how much time have you missed from your MAIN job for the following reasons?**
Please write 0 if you have not missed any time from work during this period. You can answer in days or hours, whichever applies.

a) Time missed because of the problem with your hand(s)/wrist(s) ☐☐ Days ***or*** ☐☐ Hours

b) Time missed because of any other problem ☐☐ Days ***or*** ☐☐ Hours

23 **If you fell ill and were off work, how long could you get your normal full pay (excluding bonuses)?**
Please tick the option that best represents your MAIN job.

a) Less than one week ☐
b) 1 – 4 weeks ☐
c) 1 – 6 months ☐
d) More than 6 months ☐
e) Not sure ☐

24 **How satisfied are you with your MAIN job as a whole, taking everything into consideration?** This includes your salary, career possibilities, management, colleagues etc. Please tick one box.

a) Very satisfied ☐
b) Satisfied/fairly satisfied ☐
c) Dissatisfied ☐
d) Very dissatisfied ☐

For peer review only - http://bmjopen.bmj.com/site/about/guidelines.xhtml

SECTION C: GENERAL HEALTH

25 In general, would you say your health is:

a) Excellent ☐ d) Fair ☐
b) Very good ☐ e) Poor ☐
c) Good ☐

26 What is your height? Please answer in either feet and inches or centimetres.

☐ feet ☐☐ inches ***or*** ☐☐☐ cms

27 What is your weight? Please answer in either stones and pounds or kilograms.

☐☐ stones ☐☐ lbs ***or*** ☐☐ kgs

28 Do you, or have you ever, smoked regularly? Please tick one box.

a) I have never smoked regularly ☐ c) I regularly smoke ☐
b) I have smoked in the past, but do not currently smoke regularly ☐

29 The following is a list of common health problems. Please indicate if you currently have, or don't have, the problem listed in part 1. If you have the problem, please answer the corresponding question in part 2.
Please answer all questions in part 1.

	HEALTH PROBLEM	PART 1 Do you have the problem? NO	YES (if yes move to part 2) →	PART 2 Does it limit your activities? NO	YES
a)	Heart disease	☐	☐	☐	☐
b)	High blood pressure	☐	☐	☐	☐
c)	Lung disease	☐	☐	☐	☐
d)	Diabetes	☐	☐	☐	☐
e)	Ulcer or stomach disease	☐	☐	☐	☐
f)	Kidney disease	☐	☐	☐	☐
g)	Liver disease	☐	☐	☐	☐
h)	Thyroid disease	☐	☐	☐	☐
i)	Anaemia or other blood disease	☐	☐	☐	☐
j)	Cancer	☐	☐	☐	☐

For peer review only

SECTION C: GENERAL HEALTH

HEALTH PROBLEM continued…	Do you have the problem? NO	YES (if yes move to part 2) →	Does it limit your activities? NO	YES
k) Depression	☐	☐	☐	☐
l) Osteoarthritis	☐	☐	☐	☐
m) Back pain	☐	☐	☐	☐
n) Rheumatoid arthritis	☐	☐	☐	☐

29.1 Please list any other medical problems that have not been mentioned.

	Does it limit your activities? NO	YES
o)	☐	☐
p)	☐	☐
q)	☐	☐

30 The following questions are about how you feel and how things have been with you during the *past 4 weeks*. For each question, please give the answer that comes closest to the way you have been feeling. Please tick one box for each row.

How much of the time during the past 4 weeks …	All of the time	Most of the time	A good bit of the time	Some of the time	A little bit of the time	None of the time
a) Did you feel full of 'get-up-and-go'?	☐	☐	☐	☐	☐	☐
b) Have you been a very nervous person?	☐	☐	☐	☐	☐	☐
c) Have you felt so down in the dumps that nothing could cheer you up?	☐	☐	☐	☐	☐	☐
d) Have you felt calm and peaceful?	☐	☐	☐	☐	☐	☐
e) Did you have a lot of energy?	☐	☐	☐	☐	☐	☐
f) Have you felt downhearted and blue?	☐	☐	☐	☐	☐	☐
g) Did you feel worn out?	☐	☐	☐	☐	☐	☐
h) Have you been a happy person?	☐	☐	☐	☐	☐	☐
i) Did you feel tired?	☐	☐	☐	☐	☐	☐

For peer review only - http://bmjopen.bmj.com/site/about/guidelines.xhtml

SECTION C: GENERAL HEALTH

31 **Below is a list of problems that people sometimes have. Please read each one carefully and tick the box that best describes how much that problem has distressed or bothered you during the *past 7 days*, including today?**
Please tick one box for each row.

		Not at all	A little bit	Moderately	Quite a bit	Extremely
a)	Faintness or dizziness	☐	☐	☐	☐	☐
b)	Pains in the heart or chest	☐	☐	☐	☐	☐
c)	Nausea or upset stomach	☐	☐	☐	☐	☐
d)	Trouble getting your breath	☐	☐	☐	☐	☐
e)	Hot or cold spells	☐	☐	☐	☐	☐

For peer review only

For peer review only - http://bmjopen.bmj.com/site/about/guidelines.xhtml

SECTION D: HAND AND WRIST FUNCTION

32 **In the *past 7 days*, have you experienced any pain, tingling (pins and needles) or numbness (loss of sensation) in your RIGHT hand or wrist?**
Please mark where on your hand/wrist you experienced these symptoms using the key below.
If you do not have any symptoms in your right hand, please move on to Question 34.

Pain | Tingling or numbness

RIGHT HAND

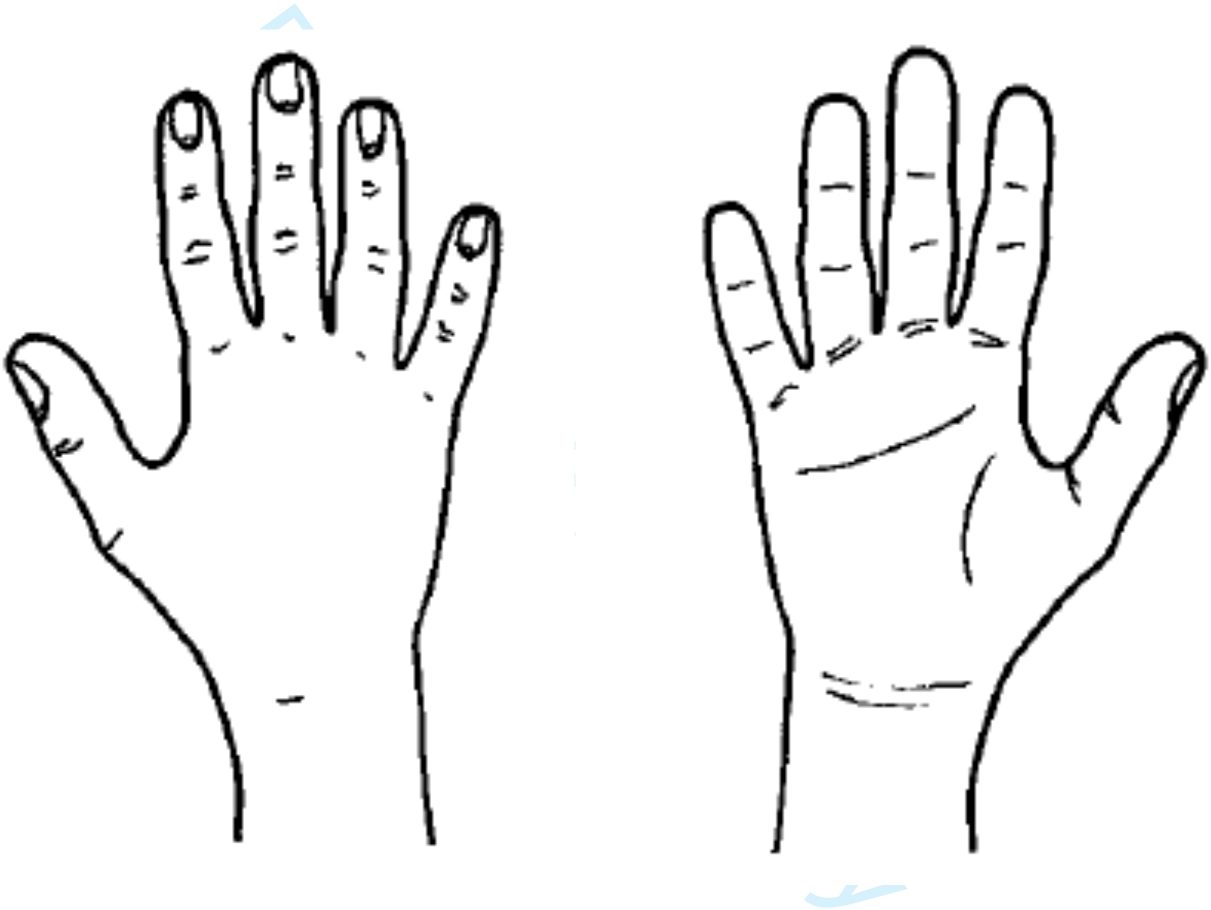

33 How long ago did the first of these symptoms begin? Please tick one box.

a) Less than 3 months ☐
b) 3 – 6 months ☐
c) 6 – 12 months ☐
d) More than a year ☐

For peer review only - http://bmjopen.bmj.com/site/about/guidelines.xhtml

SECTION D: HAND AND WRIST FUNCTION

34 **In the *past 7 days*, have you experienced any pain, tingling (pins and needles) or numbness (loss of sensation) in your LEFT hand or wrist?**
Please mark where on your hand/wrist you experienced these symptoms using the key below.
If you do not have any symptoms in your right hand, please move on to Question 36.

Pain   Tingling or numbness

LEFT HAND

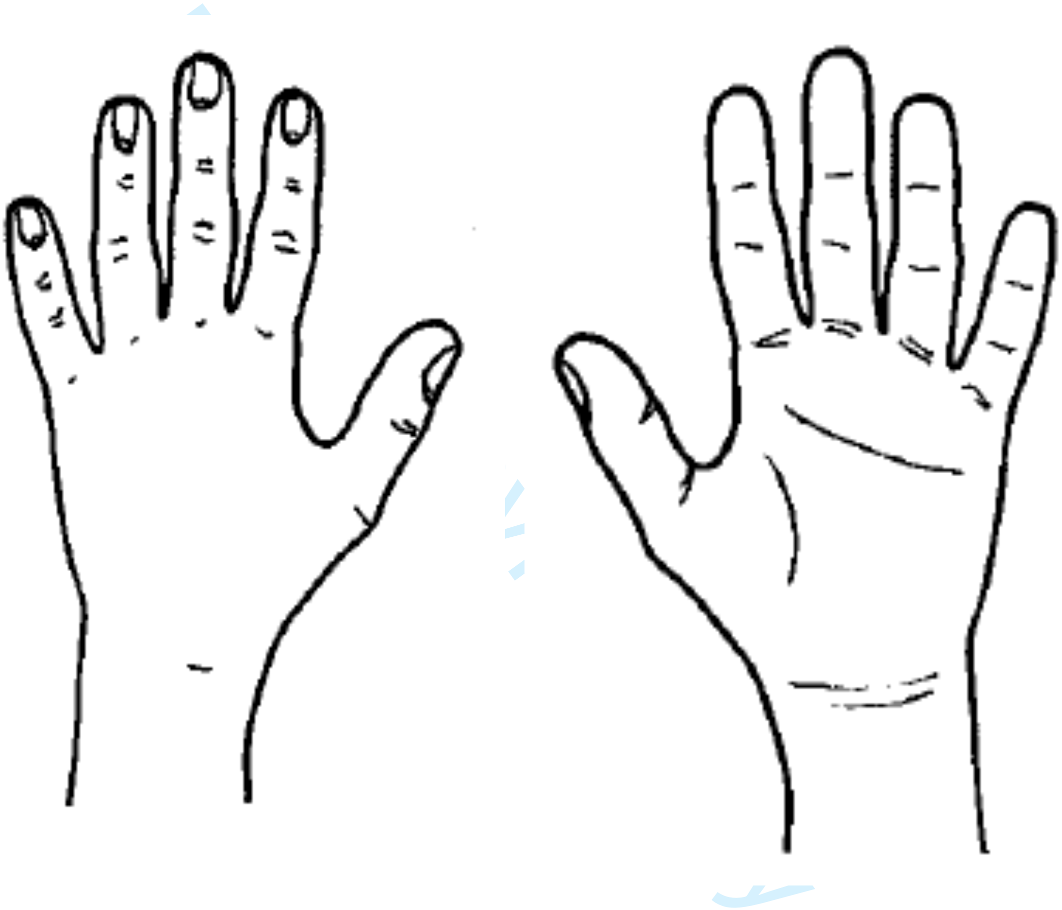

35 How long ago did the first of these symptoms begin? Please tick one box.

a) Less than 3 months ☐   c) 6 – 12 months ☐

b) 3 – 6 months ☐   d) More than a year ☐

For peer review only - http://bmjopen.bmj.com/site/about/guidelines.xhtml

SECTION D: HAND AND WRIST FUNCTION

36 **The following questions refer to your symptoms over the *last 7 days*.** Please answer for each hand, even if you only have problems with one side. Please tick one box for each row.

**36.1 How severe were the following symptoms in your RIGHT hand?**	**None**	**Mild**	**Moderate**	**Severe**	**Very severe**
a) Pain at night	☐	☐	☐	☐	☐
b) Pain during the daytime	☐	☐	☐	☐	☐
c) Numbness or tingling at night	☐	☐	☐	☐	☐
d) Numbness or tingling during the daytime	☐	☐	☐	☐	☐

**How often did the following symptoms in your RIGHT hand wake you up at night?**	**Never**	**Once**	**2 or 3 times**	**4 or 5 times**	**More than 5 times**
e) Pain	☐	☐	☐	☐	☐
f) Numbness or tingling	☐	☐	☐	☐	☐

**36.2 How severe were the following symptoms in your LEFT hand?**	**None**	**Mild**	**Moderate**	**Severe**	**Very severe**
a) Pain at night	☐	☐	☐	☐	☐
b) Pain during the daytime	☐	☐	☐	☐	☐
c) Numbness or tingling at night	☐	☐	☐	☐	☐
d) Numbness or tingling during the daytime	☐	☐	☐	☐	☐

**How often did the following symptoms in your LEFT hand wake you up at night?**	**Never**	**Once**	**2 or 3 times**	**4 or 5 times**	**More than 5 times**
e) Pain	☐	☐	☐	☐	☐
f) Numbness or tingling	☐	☐	☐	☐	☐

37 **This question refers to the appearance (look) of your hand during the *past 7 days*.** Please tick one box for each hand.

	**Strongly agree**	**Agree**	**Neither agree nor disagree**	**Disagree**	**Strongly disagree**
a) I am satisfied with the appearance (look) of my **RIGHT** hand	☐	☐	☐	☐	☐
b) I am satisfied with the appearance (look) of my **LEFT** hand	☐	☐	☐	☐	☐

For peer review only - http://bmjopen.bmj.com/site/about/guidelines.xhtml

SECTION D: HAND AND WRIST FUNCTION

38 Please answer the following questions on a scale of 0-10, where 0 represents not at all, and 10 represents very much. Please circle one number for each question.

38.1 Do you think that you will be able to use your hand normally 3 months after the operation?

0 1 2 3 4 5 6 7 8 9 10

38.2 Are you afraid of having long-term problems with your hand?

0 1 2 3 4 5 6 7 8 9 10

38.3 Do you blame yourself for your hand problem?

0 1 2 3 4 5 6 7 8 9 10

38.4 Are your family and friends supportive of your hand problem?

0 1 2 3 4 5 6 7 8 9 10

39 The following statements describe people's beliefs about their health problems. Please indicate whether you agree or disagree with them in relation to the problems you have with your hand(s) or wrist(s).
Please tick the box which most closely reflects how you feel for each statement.

	Strongly agree	Agree	Neither agree or disagree	Disagree	Strongly Disagree
a) Problems like this run in my family	☐	☐	☐	☐	☐
b) I think I was born with a weakness or underlying problem in this part of my body	☐	☐	☐	☐	☐
c) My problem was caused by work	☐	☐	☐	☐	☐
d) Work probably didn't cause my problem, but it made it worse	☐	☐	☐	☐	☐
e) I have a lot of stress in my life and that has made my problem a lot worse	☐	☐	☐	☐	☐
f) I think a lack of exercise probably contributed to my problem	☐	☐	☐	☐	☐
g) As you get older, parts of the body start to wear out and problems like mine are likely to occur	☐	☐	☐	☐	☐

For peer review only - http://bmjopen.bmj.com/site/about/guidelines.xhtml

SECTION D: HAND AND WRIST FUNCTION

40 We are interested in the types of thoughts and feelings that you have when you are in pain. The following statements describe different thoughts and feelings that may be associated with pain. Please indicate the degree to which you have these thoughts and feelings when you are experiencing pain.
Please tick one box for each statement.

	Not at all	To a slight degree	To a moderate degree	To a great degree	All of the time
a) I keep thinking about how badly I want the pain to stop	☐	☐	☐	☐	☐
b) It's terrible and I think it's never going to get any better	☐	☐	☐	☐	☐
c) I become afraid that the pain may get worse	☐	☐	☐	☐	☐
d) I anxiously want the pain to go away	☐	☐	☐	☐	☐

The following questions refer to the function of your hands/wrists during the ***past 7 days***. Please answer all questions for the right and left sides, even if you do not experience any problems. Please tick one box for each question.

**41 RIGHT SIDE**	**Very well**	**Well**	**Adequately**	**Poorly**	**Very poorly**
a) Overall, how well did your ***right*** hand work?	☐	☐	☐	☐	☐
b) How well did your ***right*** fingers move?	☐	☐	☐	☐	☐
c) How well did your ***right*** wrist move?	☐	☐	☐	☐	☐
	**Very good**	**Good**	**Fair**	**Poor**	**Very poor**
d) How was the strength in your ***right*** hand?	☐	☐	☐	☐	☐
e) How was the sensation (feeling) in your ***right*** hand?	☐	☐	☐	☐	☐

**42 LEFT SIDE**	**Very well**	**Well**	**Adequately**	**Poorly**	**Very poorly**
a) Overall, how well did your ***left*** hand work?	☐	☐	☐	☐	☐
b) How well did your ***left*** fingers move?	☐	☐	☐	☐	☐
c) How well did your ***left*** wrist move?	☐	☐	☐	☐	☐
	**Very good**	**Good**	**Fair**	**Poor**	**Very poor**
d) How was the strength in your ***left*** hand?	☐	☐	☐	☐	☐
e) How was the sensation (feeling) in your ***left*** hand?	☐	☐	☐	☐	☐

For peer review only

SECTION D: HAND AND WRIST FUNCTION

The following questions refer to the ability of your hands to do certain tasks during the ***past 7 days***. If you do not do a certain task, please estimate the difficulty you would have in performing it. Please tick one box for every activity.

43 How difficult was it for you to perform the following activities using your RIGHT HAND?

		Not at all difficult	A little difficult	Somewhat difficult	Moderately difficult	Very difficult
a)	Turn a door knob	☐	☐	☐	☐	☐
b)	Pick up a coin	☐	☐	☐	☐	☐
c)	Hold a glass of water	☐	☐	☐	☐	☐
d)	Turn a key in a lock	☐	☐	☐	☐	☐
e)	Hold a frying pan	☐	☐	☐	☐	☐

44 How difficult was it for you to perform the following activities using your LEFT HAND?

		Not at all difficult	A little difficult	Somewhat difficult	Moderately difficult	Very difficult
a)	Turn a door knob	☐	☐	☐	☐	☐
b)	Pick up a coin	☐	☐	☐	☐	☐
c)	Hold a glass of water	☐	☐	☐	☐	☐
d)	Turn a key in a lock	☐	☐	☐	☐	☐
e)	Hold a frying pan	☐	☐	☐	☐	☐

45 How difficult was it for you to perform the following activities using BOTH HANDS?

		Not at all difficult	A little difficult	Somewhat difficult	Moderately difficult	Very difficult
a)	Open a jar	☐	☐	☐	☐	☐
b)	Button a shirt/blouse	☐	☐	☐	☐	☐
c)	Eat with a knife/fork	☐	☐	☐	☐	☐
d)	Carry a grocery bag	☐	☐	☐	☐	☐
e)	Wash dishes	☐	☐	☐	☐	☐
f)	Wash your hair	☐	☐	☐	☐	☐
g)	Tie shoelaces/knots	☐	☐	☐	☐	☐

For peer review only

SECTION D: HAND AND WRIST FUNCTION

The following questions refer to your satisfaction with your hands/wrists during the ***past 7 days***. Please tick one box for each question

46 How satisfied were you with your RIGHT hand/wrist during the *past 7 days*?

	RIGHT HAND	Very satisfied	Somewhat satisfied	Neither satisfied or dissatisfied	Somewhat dissatisfied	Very dissatisfied
a)	Overall function of your hand	☐	☐	☐	☐	☐
b)	Movement of the fingers	☐	☐	☐	☐	☐
c)	Movement of your wrist	☐	☐	☐	☐	☐
d)	Strength of your hand	☐	☐	☐	☐	☐
e)	Pain level of your hand	☐	☐	☐	☐	☐
f)	Sensation (feeling) of your hand	☐	☐	☐	☐	☐

47 How satisfied were you with your LEFT hand/wrist during the *past 7 days*?

	LEFT HAND	Very satisfied	Somewhat satisfied	Neither satisfied or dissatisfied	Somewhat dissatisfied	Very dissatisfied
a)	Overall function of your hand	☐	☐	☐	☐	☐
b)	Movement of the fingers	☐	☐	☐	☐	☐
c)	Movement of your wrist	☐	☐	☐	☐	☐
d)	Strength of your hand	☐	☐	☐	☐	☐
e)	Pain level of your hand	☐	☐	☐	☐	☐
f)	Sensation (feeling) of your hand	☐	☐	☐	☐	☐

Thank you for completing this questionnaire!
Please return it to the REACTS team
using the pre-paid envelope.

REACTS

If you have any questions, or would like any additional information, please contact Lisa Newington on:
ln@mrc.soton.ac.uk | 023 8077 7624 | 07866 997732

For peer review only - http://bmjopen.bmj.com/site/about/guidelines.xhtml

University Hospital Southampton NHS Foundation Trust

REACTS

Arthritis Research UK MRC Medical Research Council centre for musculoskeletal health & work

UNIVERSITY OF Southampton

REACTS ID: | | | | | |

One Month After Your Carpal Tunnel Release Surgery

REACTS

Return to Employment After Carpal Tunnel Release Surgery (REACTS)

ln@mrc.soton.ac.uk | 023 8077 7624
Arthritis Research UK – MRC Centre for Musculoskeletal Health and Work
MRC Lifecourse Epidemiology Unit, University of Southampton
Southampton General Hosptial (MP 95), SO16 6YD

IRAS reference: 209840

For peer review only - http://bmjopen.bmj.com/site/about/guidelines.xhtml

SECTION A: ABOUT YOUR OPERATION

Please fill in today's date ☐☐☐☐☐☐☐☐
d d m m y y y y

1 What was the date of your carpal tunnel release surgery? ☐☐☐☐☐☐☐☐
d d m m y y y y

2 Which side was operated on? Please tick one box.

Right ☐ Left ☐ Both ☐

3 What type of anaesthetic did you have? Please tick one box.

a) General anaesthetic (you were sent to sleep) ☐

b) Local or regional anaesthetic (your arm was made numb, but you were still awake) ☐

c) Other *(please specify)* ☐

----

d) Unsure ☐

4 How long did you need to stay in the hospital/clinic after your operation?
Please tick one box (and specify the number of nights, if applicable).

a) I went home the same day ☐

b) I needed to stay overnight (one night only) ☐

c) I needed to stay for more than one night ☐

(Please specify for how long) ☐☐ nights

For peer review only

SECTION A: ABOUT YOUR OPERATION

5 Have you used any of the following services specifically for your operated hand(s) since your surgery?
Please give the number of visits for each service, and the date(s) attended, if known.

	I used this service in the NHS		I used this service privately	
	Number of visits	Dates attended, if known	Number of visits	Dates attended, if known
a) Your surgeon, or one of the surgical team				
b) GP or practice nurse				
c) Hospital nurse				
d) Pharmacist				
e) Hand therapist				
f) Other physiotherapist or occupational therapist				
g) Chiropractor or osteopath				
h) Occupational health nurse or doctor				
i) Accident and emergency (A&E) or minor injuries unit				
j) Other (*please specify)*				

For peer review only - http://bmjopen.bmj.com/site/about/guidelines.xhtml

SECTION A: ABOUT YOUR OPERATION

6 Have you taken any antibiotics for an infection in your surgical wound?
Please do not include any antibiotics you were prescribed at the time of your operation.

Yes ☐ No ☐

If yes, what date did you start taking the antibiotics?

d d m m y y y y

7 Have you been admitted to hospital because of a problem with your operated hand(s)? If yes, please answer the rest of Question 7; if no, please move on to Question 8.

Yes ☐ No ☐

7.1 If yes, when were you first admitted?

d d m m y y y y

7.2 How many nights did you stay in hospital?
Please answer 0 if you didn't stay overnight.

nights

7.3 Did you require another operation? Yes ☐ No ☐

8 Have you been advised that you may need a carpal tunnel release for your other hand in the future?
If yes, please answer Question 8.1; if no, please move on to Question 9.

Yes ☐ No ☐

8.1 If yes, when are you expecting to have this surgery? Please tick one box.

a) In less than 2 months ☐
b) In 2-5 months ☐
c) In 6-11 months ☐
d) In more than a year ☐
e) Unsure ☐

9 If you would like to give us any other information about your operation, or the healthcare services you have used, please do so here:

For peer review only

For peer review only - http://bmjopen.bmj.com/site/about/guidelines.xhtml

SECTION B: WORK

10 Compared to before your surgery, which of the following best describes your current work situation? Please tick one box.

a) Returned to the same job, work duties and hours – **please go to Question 14** ☐

b) Returned to the same job, with altered duties or hours – **please go to Question 14** ☐

c) Started a new job – **please go to Question 11** ☐

d) Not yet returned to work, but plan to return in the future – **please go to Question 12** ☐

e) Do not plan to return to work – **please go to Question 13** ☐

11 Thinking about your new job:

11.1 What is your main occupation now (e.g. secretary, teacher, builder etc.)?

11.2 In what industry do you work (e.g. farming, shipyard, car factory, shoe shop, hospital, insurance office etc.)?

11.3 Did you change jobs because of your hand/wrist problem?
Please tick one box.

a) Yes, my hand/wrist problem was the main reason for my job change ☐

b) Yes, my hand/wrist problem was one of several reasons for my job change ☐

c) No, my job change was nothing to do with my hand/wrist problem ☐

d) Other, please specify ☐

Please go to Question 14

For peer review only - http://bmjopen.bmj.com/site/about/guidelines.xhtml

SECTION B: WORK

12 If you have not yet returned to work, when do you think you might be able to return? Please give an estimated date if you are unsure.

d	d	m	m	y	y	y	y

12.1 Have you discussed when to return to work with anyone?
If yes, please answer the rest of Question 12; if no, please move on to Question 21.

Yes ☐ No ☐

12.2 If yes, who have you discussed this with? Please tick all that apply.

a) Your surgeon or a member of the surgical team ☐
b) Hospital nurse ☐
c) GP or practice nurse ☐
d) Hand therapist ☐
e) Physiotherapist or occupational therapist ☐
f) Occupational health nurse or doctor ☐
g) Employer or manager (or colleagues if self-employed ☐
h) Friend or family member ☐
i) Other *(please specify)* ☐

12.3 Since your operation, have you been given any specific advice about when and how to return to work? This could include any activities to avoid or timescales to follow. Please list any advice here, including who gave you this advice:

Please go to Question 21

For peer review only - http://bmjopen.bmj.com/site/about/guidelines.xhtml
For peer review only

SECTION B: WORK

13 If you do not plan to return to work, what is the main reason for this decision?
Please tick one box.

a) Retirement ☐
b) Redundancy ☐
c) Position/work no longer available ☐
d) Unable to do your work because of your problem with your hand(s)/wrist(s) ☐
e) Unable to do your work because of any other problem ☐
f) Other *(please specify)* ☐

13.1 Have you been advised not to return to work by anyone?
If yes, please answer Question 13.2; if no, please move on to Question 21.

Yes ☐ No ☐

13.2 If yes, who by? Please tick all that apply.

a) Your surgeon or a member of the surgical team ☐
b) Hospital nurse ☐
c) GP or practice nurse ☐
d) Hand therapist ☐
e) Physiotherapist or occupational therapist ☐
f) Occupational health nurse or doctor ☐
g) Employer or manager (or colleagues if self-employed) ☐
h) Friend or family member ☐
i) Other *(please specify)* ☐

Please go to Question 21

For peer review only - http://bmjopen.bmj.com/site/about/guidelines.xhtml

SECTION B: WORK

14 When did you first return to work after your carpal tunnel release surgery?

d d m m y y y y

15 How much work-time did you miss between the date of your surgery and the date you first returned to work?
Please include all work-time missed, even if this had been pre-arranged with your employer, or was taken as annual leave. You can answer in hours, days or weeks, whichever applies.

hours days weeks

15.1 Was any of this time paid?
Please tick one box (and provide the amount of time, if applicable).

a) Yes, all of my time away from work was paid

b) Yes, some of my time away from work was paid
(please specify how much time was paid, you can use hours, days or weeks, whichever applies)

hours days weeks

c) No, none of my time off was paid

d) Not sure

16 Since your surgery, have you discussed when to return to work with anyone?
If yes, please answer the rest of Question 16; if no, please move on to Question 17.

Yes No

16.1 If yes, who did you discuss this with? Please tick all that apply.

a) Your surgeon or a member of the surgical team

b) Hospital nurse

c) GP or practice nurse

d) Hand therapist

e) Physiotherapist or occupational therapist

f) Occupational health nurse or doctor

g) Employer or manager (or colleagues if self-employed

h) Friend or family member

i) Other *(please specify)*

For peer review only - http://bmjopen.bmj.com/site/about/guidelines.xhtml

SECTION B: WORK

16.2 Please list any advice you have been given (since your surgery) about when and how to return to work?
This could include any activities to avoid or timescales to follow. If this advice came from more than one place, please indicate who advised what.

----

17 Since returning to work after your operation, have you needed to take any time off work because of a problem with your operated hand(s)/wrist(s)?
If yes, please answer Question 17.1; if no, please move on to Question 18.

Yes ☐ No ☐

17.1 If yes, how much time did you take off work?
Please answer in days or hours, whichever applies.

☐☐☐ hours ☐☐ days ☐☐ weeks

18 When you first returned to work after your surgery, did you work shorter hours than would be normal for your job as a direct result of your operation?
If yes, please answer the rest of Question 18; if no, please move on to Question 19.

Yes ☐ No ☐

18.1 Have you since gone back to working full hours?
If yes, please answer Question 18.2; if no, please move on to Question 19.

Yes ☐ No ☐

18.2 If yes, when did you return to full working hours?
If you do not know the exact date, approximately how many weeks did you work reduced hours?

☐☐☐☐☐☐☐☐
d d m m y y y y

a) Less than a week ☐

b) 1 – 2 weeks ☐

c) More than 2 weeks, but less than 3 weeks ☐

d) 3 weeks or longer ☐

For peer review only - http://bmjopen.bmj.com/site/about/guidelines.xhtml

SECTION B: WORK

19 When you first returned to work after your surgery, did you need to alter or avoid any of your usual work duties as a direct result of your operation?
If yes, please answer the rest of Question 19; if no, please move on to Question 20.

Yes ☐ No ☐

19.1 Have you since gone back to full duties?
If yes, please answer Question 19.2; if no, please move on to Question 20.

Yes ☐ No ☐

19.2 If yes, when did you return to full working duties?
If you do not know the exact date, approximately how many weeks did you have altered work duties?

d	d	m	m	y	y	y	y

a) Less than a week ☐

b) 1 – 2 weeks ☐

c) More than 2 weeks, but less than 3 weeks ☐

d) 3 weeks or longer ☐

20 If you would like to give us any additional information about returning to work, please do so here:

For peer review only - http://bmjopen.bmj.com/site/about/guidelines.xhtml

SECTION C: HAND AND WRIST SYMPTOMS

21 The following questions refer to your symptoms over the *last 7 days*.
Please answer for each hand. Please tick one box for each row.

**21.1 How severe were the following symptoms in your RIGHT hand?**	None	Mild	Moderate	Severe	Very severe
a) Pain at night	☐	☐	☐	☐	☐
b) Pain during the daytime	☐	☐	☐	☐	☐
c) Numbness or tingling at night	☐	☐	☐	☐	☐
d) Numbness or tingling during the daytime	☐	☐	☐	☐	☐

**How often did the following symptoms in your RIGHT hand wake you up at night?**	Never	Once	2 or 3 times	4 or 5 times	More than 5 times
e) Pain	☐	☐	☐	☐	☐
f) Numbness or tingling	☐	☐	☐	☐	☐

**21.2 How severe were the following symptoms in your LEFT hand?**	None	Mild	Moderate	Severe	Very severe
a) Pain at night	☐	☐	☐	☐	☐
b) Pain during the daytime	☐	☐	☐	☐	☐
c) Numbness or tingling at night	☐	☐	☐	☐	☐
d) Numbness or tingling during the daytime	☐	☐	☐	☐	☐

**How often did the following symptoms in your LEFT hand wake you up at night?**	Never	Once	2 or 3 times	4 or 5 times	More than 5 times
e) Pain	☐	☐	☐	☐	☐
f) Numbness or tingling	☐	☐	☐	☐	☐

22 This question refers to the appearance (look) of your hands during the *past 7 days*. Please tick one box for each hand.

	Strongly agree	Agree	Neither agree nor disagree	Disagree	Strongly disagree
a) I am satisfied with the appearance (look) of my **RIGHT** hand	☐	☐	☐	☐	☐
b) I am satisfied with the appearance (look) of my **LEFT** hand	☐	☐	☐	☐	☐

For peer review only

SECTION C: HAND AND WRIST SYMPTOMS

23 **How do you rate your symptoms in your operated hand(s) now, compared to before your surgery?** Please tick one box.

a) Completely cured ☐ c) Unchanged ☐ e) Worse ☐

b) Much better ☐ d) Slightly better ☐

24 The following questions ask specifically about your scar. Please think about your scar over the ***past 7 days.***

24.1 **Has your scar been itchy?**
If yes, please continue; if no, please move on to Question 24.2

Yes ☐ No ☐

Yes, it was itchy:	Sometimes ☐	Often ☐	Always ☐
And when it was itchy, it was:	Slightly itchy ☐	Fairly itchy ☐	Very itchy ☐

24.2 **Has your scar caused you pain?**
If yes, please continue; if no, please move on to Question 24.3

Yes ☐ No ☐

Yes, it was painful:	Sometimes ☐	Often ☐	Always ☐
And when it hurt, it was:	Slightly painful ☐	Fairly painful ☐	Very painful ☐

24.3 **Has your scar been uncomfortable?**
If yes, please continue; if no, please move on to Question 24.4

Yes ☐ No ☐

Yes, it was uncomfortable:	Sometimes ☐	Often ☐	Always ☐
And when it was uncomfortable, it was:	Slightly uncomfortable ☐	Fairly uncomfortable ☐	Very uncomfortable ☐

For peer review only - http://bmjopen.bmj.com/site/about/guidelines.xhtml

SECTION C: HAND AND WRIST SYMPTOMS

24.4 Has your scar felt numb?
If yes, please continue; if no, please move on to Question 24.5

Yes ☐ No ☐

Yes, it was numb:	Sometimes ☐	Often ☐	Always ☐
And when it felt numb, it was:	Slightly numb ☐	Fairly numb ☐	Very numb ☐

24.5 Have you had odd sensations in your scar e.g. tightening, pulling or pins and needles? If yes, please continue; if no, please move on to Question 24.6

Yes ☐ No ☐

Yes, I have had odd sensations:	Sometimes ☐	Often ☐	Always ☐

24.6 Has your scar caught on things e.g. clothing?
If yes, please continue; if no, please move on to Question 24.7

Yes ☐ No ☐

Yes, it has caught on things:	Sometimes ☐	Often ☐	Always ☐

24.7 Overall, how troublesome are the symptoms from your scar?
Please tick one box.

Not at all troublesome	A little troublesome	Fairly troublesome	Very troublesome	Unbearable
☐	☐	☐	☐	☐

For peer review only - http://bmjopen.bmj.com/site/about/guidelines.xhtml

SECTION D: HAND AND WRIST FUNCTION

The following questions refer to the function of your hands/wrists during the ***past 7 days***. Please answer all questions for the right and left sides, even if you do not experience any problems. Please tick one box for each question.

**25 RIGHT SIDE**	**Very well**	**Well**	**Adequately**	**Poorly**	**Very poorly**
a) Overall, how well did your ***right*** hand work?	☐	☐	☐	☐	☐
b) How well did your ***right*** fingers move?	☐	☐	☐	☐	☐
c) How well did your ***right*** wrist move?	☐	☐	☐	☐	☐
	**Very good**	**Good**	**Fair**	**Poor**	**Very poor**
d) How was the strength in your ***right*** hand?	☐	☐	☐	☐	☐
e) How was the sensation (feeling) in your ***right*** hand?	☐	☐	☐	☐	☐

**26 LEFT SIDE**	**Very well**	**Well**	**Adequately**	**Poorly**	**Very poorly**
a) Overall, how well did your ***left*** hand work?	☐	☐	☐	☐	☐
b) How well did your ***left*** fingers move?	☐	☐	☐	☐	☐
c) How well did your ***left*** wrist move?	☐	☐	☐	☐	☐
	**Very good**	**Good**	**Fair**	**Poor**	**Very poor**
d) How was the strength in your ***left*** hand?	☐	☐	☐	☐	☐
e) How was the sensation (feeling) in your ***left*** hand?	☐	☐	☐	☐	☐

The following questions refer to the ability of your hands to do certain tasks during the ***past 7 days***. If you do not do a certain task, please estimate the difficulty you would have in performing it. Please tick one box for every activity.

27 How difficult was it for you to perform the following activities using your RIGHT HAND?

	**Not at all difficult**	**A little difficult**	**Somewhat difficult**	**Moderately difficult**	**Very difficult**
a) Turn a door knob	☐	☐	☐	☐	☐
b) Pick up a coin	☐	☐	☐	☐	☐
c) Hold a glass of water	☐	☐	☐	☐	☐
d) Turn a key in a lock	☐	☐	☐	☐	☐
e) Hold a frying pan	☐	☐	☐	☐	☐

For peer review only

SECTION D: HAND AND WRIST FUNCTION

28 How difficult was it for you to perform the following activities using your LEFT HAND?

	Not at all difficult	A little difficult	Somewhat difficult	Moderately difficult	Very difficult
a) Turn a door knob	☐	☐	☐	☐	☐
b) Pick up a coin	☐	☐	☐	☐	☐
c) Hold a glass of water	☐	☐	☐	☐	☐
d) Turn a key in a lock	☐	☐	☐	☐	☐
e) Hold a frying pan	☐	☐	☐	☐	☐

29 How difficult was it for you to perform the following activities using BOTH HANDS?

	Not at all difficult	A little difficult	Somewhat difficult	Moderately difficult	Very difficult
a) Open a jar	☐	☐	☐	☐	☐
b) Button a shirt/blouse	☐	☐	☐	☐	☐
c) Eat with a knife/fork	☐	☐	☐	☐	☐
d) Carry a grocery bag	☐	☐	☐	☐	☐
e) Wash dishes	☐	☐	☐	☐	☐
f) Wash your hair	☐	☐	☐	☐	☐
g) Tie shoelaces/knots	☐	☐	☐	☐	☐

The following questions refer to your satisfaction with your hands/wrists during the ***past 7 days***. Please tick one box for each question.

30 How satisfied were you with your RIGHT hand/wrist during the *past 7 days*?

**RIGHT HAND**	Very satisfied	Somewhat satisfied	Neither satisfied or dissatisfied	Somewhat dissatisfied	Very dissatisfied
a) Overall function of your hand	☐	☐	☐	☐	☐
b) Movement of the fingers	☐	☐	☐	☐	☐
c) Movement of your wrist	☐	☐	☐	☐	☐
d) Strength of your hand	☐	☐	☐	☐	☐
e) Pain level of your hand	☐	☐	☐	☐	☐
f) Sensation (feeling) of your hand	☐	☐	☐	☐	☐

For peer review only - http://bmjopen.bmj.com/site/about/guidelines.xhtml

SECTION D: HAND AND WRIST FUNCTION

31 How satisfied were you with your LEFT hand/wrist during the *past 7 days*?

	LEFT HAND	Very satisfied	Somewhat satisfied	Neither satisfied or dissatisfied	Somewhat dissatisfied	Very dissatisfied
a)	Overall function of your hand	☐	☐	☐	☐	☐
b)	Movement of the fingers	☐	☐	☐	☐	☐
c)	Movement of your wrist	☐	☐	☐	☐	☐
d)	Strength of your hand	☐	☐	☐	☐	☐
e)	Pain level of your hand	☐	☐	☐	☐	☐
f)	Sensation (feeling) of your hand	☐	☐	☐	☐	☐

32 If you would like to give us any additional information about your hand and wrist function, please do so here:

Thank you for completing this questionnaire!
Please return it to the REACTS team
using the pre-paid envelope.

REACTS

If you have any questions or would like any additional information, please contact Lisa Newington on:
ln@mrc.soton.ac.uk | 023 8077 7624 | 07866 997732

For peer review only - http://bmjopen.bmj.com/site/about/guidelines.xhtml

SUPPLEMENTARY TABLES

1. Cox proportional hazards univariate analyses of the association between demographic and general health factors and the duration of work absence after carpal tunnel release (non-significant findings)

	N	Median time to return to work (days)	Inter quartile range	Hazards ratio [1]	95% Confidence intervals	P value
Body mass index (kg/m^2) (censored 1 normal, 1 overweight, 2 obese; 13 missing)						
Normal (18.5-24.9)	47	20	12-33	1	-	-
Over (25.0-29.9)	64	19	12.5-29	0.98	0.65, 1.46	0.91
Obese (≥30)	67	21	10-40	0.88	0.60, 1.28	0.50
Surgery to dominant hand [2] (censored 3 yes, 2 no; no missing data)						
Yes	128	19.5	12-33	1	-	-
No	62	22	12-35	0.83	0.61, 1.14	0.25
General health status (censored 5 excellent; 1 missing)						
Excellent/very good/ good	165	19	12-32	1	-	-
Fair/poor	24	28.5	12.5-42	0.74	0.47, 1.16	0.19
Number of comorbidities [3] (censored 3 none, 1 one; 1 missing)						
None	49	18	7-32	1	-	-
One	67	24	14-42	0.87	0.59, 1.27	0.47
Two or more	74	19.5	10-29	1.11	0.76, 1.62	0.60
Number of disability comorbidities [3] (censored 3 none, 2 one; no missing data)						
None	130	19	9-33	1	-	-
One	39	26	13-31	0.75	0.52, 1.08	0.12
Two or more	21	20	14-31	0.81	0.50, 1.32	0.40
Number of somatising symptoms [4] (censored 1 none, 1 one, 3 two or more; 1 missing)						
None	96	19	10.5-35	1	-	-
One	51	22	13-34	0.98	0.70, 1.38	0.91
Two or more	42	19.5	14-31	0.84	0.58, 1.22	0.36
SF36 Mental health score (tertiles) [5] (censored 2 poor, 1 intermediate, 2 good; 2 missing)						
Poor (22.2-57.8)	60	24	16-39	0.71	0.50, 1.03	0.07
Intermediate (60.0-75.6)	68	18.5	11-36.5	0.88	0.62, 1.25	0.48
Good (77.8-97.8)	60	16.5	7-30	1	-	-

Total sample size n=195. Median duration of work absence relates to the 190 non-censored events.

1. Adjusted for age and sex.

2. Considered and the non-dominant hand for those who reported ambidexterity (n=4) and dominant hand for those undergoing simultaneous bilateral surgery (n=2).

3. Adapted from the self-administered co-morbidity questionnaire [1].

4. Adapted from the four-dimensional symptom questionnaire [2].

5. Taken from the SF-36 with data-driven tertiles [3].

For peer review only

2. Cox proportional hazards univariate analyses of the association between health beliefs and the duration of work absence after carpal tunnel release (non-significant findings)

	N	Median time to return to work (days)	Inter quartile range	Hazards ratio [1]	95% Confidence intervals	P value
Believe will be unable to use hand normally in 3 months [2] (censored 5 no; 2 missing)						
No	168	20	12-32.5	1	-	-
Yes	20	20.5	14.5-40	1.04	0.65, 1.67	0.87
Blames self for hand problem [2] (censored 5 no; 2 missing)						
No	162	20	12-35	1	-	-
Yes	26	22	14-31	1.08	0.70, 1.66	0.73
Lack of support from family/friends [2] (censored 4 no, 1 yes; 2 missing)						
No	156	20	12-32.5	1	-	-
Yes	32	19.5	13-40	0.88	0.60, 1.29	0.51
Problem runs in the family/born with a weakness [3] (censored 3 no, 2 agree/strongly agree; 2 missing)						
No	134	20	12-35	1	-	-
Agree/strongly agree	54	23	12-31	0.88	0.63, 1.21	0.42
Stress made the problem worse [3] (censored 5 no; 2 missing)						
No	169	20	12-33	1	-	-
Agree/strongly agree	19	28	14-40	0.79	0.48, 1.28	0.33
Lack of exercise contributed to the problem [3] (censored 5 no; 2 missing)						
No	172	20	13-33	1	-	-
Agree/strongly agree	16	15	10.5-34.5	1.23	0.73, 2.06	0.43
Getting older contributes to the problem [3] (censored 3 no, 2 yes; 3 missing)						
No	79	26	14-42	1	-	-
Agree/strongly agree	108	16	10-29	1.28	0.94, 1.74	0.11
Work probably didn't cause the problem, but made it worse [3] (censored 2 no, 3 yes; 4 missing)						
No	100	20.5	12-35	1	-	-
Agree/strongly agree	86	19.5	13-31	1.01	0.75, 1.35	0.95
Pain catastrophisation to at least a moderate degree [4] (censored 3 no, 2 yes; missing 2)						
No	133	19	10-33	1	-	-
Yes	55	22	15-31	0.81	0.59, 1.12	0.21

Total sample size n=195. Median duration of work absence relates to the 190 non-censored events.

1. Adjusted for age and sex.

2. Reported on a 0-10 scale and dichotomised to no (0-6) and yes (7-10) [4].

3. Reported on a 5-point scale and dichotomised to agree/strongly agree and neither agree nor disagree/disagree/disagree strongly [5].

4. Pain catastrophizing scale dichotomised to those who reported catastrophising pain thoughts and feelings to at least a moderate degree in response to all questions and those who did not [6].

For peer review only

3. Cox proportional hazards univariate analyses of the association between clinical and surgical factors and the duration of work absence after carpal tunnel release (non-significant findings)

	N	Median time to return to work (days)	Inter quartile range	Hazards ratio [1]	95% Confidence intervals	P value
Katz hand diagram score for side of surgery [2] (censored 3 classics/probable, 2 possible/unlikely; 4 missing)						
Classic/probable	126	22	14-38	1	-	-
Possible/unlikely	60	16.5	9.5-30.5	1.10	0.81, 1.51	0.54
Duration of symptoms (censored 1 ≤ 1 year, 4 > 1 year; 2 missing)						
≤ 1 year	47	19	10-31	1	-	-
> 1 year	141	20	13-34	0.86	0.62, 1.21	0.38
Nerve conduction studies performed (censored 3 no, 2 yes; 40 missing)						
No	71	21	14-35	1	-	-
Yes	79	20	11-33	1.14	0.83, 1.58	0.41
Type of suture material (censored 4 non-absorbable; 49 missing)						
Non-absorbable	119	21	12-38	1	-	-
Absorbable	23	20	13-29	1.15	0.73, 1.83	0.55
MHQ function score for side of surgery (tertiles) [3] (censored 3 poor, 1 intermediate, 1 good; 2 missing)						
Poor (0-40)	79	20	13-41	0.80	0.57, 1.12	0.19
Intermediate (44-55)	48	19.5	13.5-33	0.81	0.55, 1.19	0.29
Good (60-100)	61	20	7-29	1	-	-
MHQ bilateral activities of daily living score (tertiles) [3] (censored 1 poor, 4 intermediate; 1 missing)						
Poor (0-61)	72	19	12.5-33.5	0.96	0.66, 1.38	0.81
Intermediate (64-82)	55	21	13-38	0.70	0.49, 1.01	0.06
Good (83-100)	62	20	10-31	1	-	-
MHQ activities of daily living score, side of surgery (tertiles) [3] (censored 2 poor, 3 intermediate; 1 missing)						
Poor (0-55)	63	20	12-42	0.81	0.56, 1.18	0.27
Intermediate (58-80)	63	20	14-35	0.86	0.61, 1.23	0.42
Good (85-100)	63	20	9-32	1	-	-
MHQ satisfaction score for side of surgery (tertiles) [3] (censored 3 poor, 2 intermediate; no missing data)						
Poor (0-25)	88	21.5	13-42	0.78	0.55, 1.11	0.16
Intermediate (29-50)	49	18	14-29	0.87	0.59, 1.28	0.48
Good (54-100)	53	20	9-31	1	-	-
Satisfaction with appearance for side of surgery [4] (censored 2 satisfied, 3 dissatisfied; 1 missing)						
Satisfied	136	20	10.5-31.5	1	-	-
Dissatisfied	53	20	13-40	0.84	0.61, 1.16	0.29

Total sample size n=195. Median duration of work absence relates to the 190 non-censored events.

1. Adjusted for age and sex.

2. Adapted from Calfee et al. [7].

3. Michigan Hand Questionnaire [8]. Data-driven tertiles.

4. Reported on a 5-point scale in response to the statement: I am satisfied with the appearance (look) of my hand. Dichotomised as satisfied (strongly agree/agree) and dissatisfied (neither agree nor disagree, disagree, strongly disagree).

For peer review only

4. **Cox proportional hazards univariate analyses of the association between occupational factors and the duration of work absence after carpal tunnel release (non-significant findings)**

	N	Median time to return to work (days)	Inter quartile range	Hazards ratio [1]	95% Confidence intervals	P value
Have more than one paid job (censored 4 no, 1 yes; 1 missing)						
No	177	20	12-32	1	-	-
Yes	12	26.5	19-42	0.61	0.34, 1.11	0.11
Total paid work hours per week [2] (censored 4 ≤37.5, 1 >37.5; no missing data)						
≤37.5	97	22	15-38	1	-	-
>37.5	93	16	6-29	1.25	0.88, 1.79	0.21
Number of work days per week (censored 1 <5, 4 5; no missing data)						
<5	55	23	15-35	1	0.72, 1.39	0.98
5	116	19	10.5-33	1	-	-
>5	19	13	6-31	1.28	0.77, 2.14	0.34
Sick leave taken for this problem in the last month (censored 5 no; 16 missing)						
No	154	20	12-33	1	-	-
Yes	20	15	9.5-30	0.99	0.61, 1.60	0.95
Sick leave taken for another problem in the last month (censored 4 no, 1 yes; 20 missing)						
No	152	19	10.5-31	1	-	-
Yes	18	30	21-42	0.67	0.41, 1.09	0.10
Received advice about return to work after surgery (censored 5 yes; 3 missing)						
Yes	134	19	12-31	1	-	-
No	53	22	14-41	0.92	0.67, 1.27	0.61
Required to work to tight deadlines [3] (censored 3 no, 2 yes; missing 3)						
No	75	23	14-40	1	-	-
Yes	112	18	9.5-31	1.19	0.89, 1.61	0.24
Use power tools that make the hand/arm vibrate [3] (censored 5 no; missing 7)						
No	145	19	12-32	1	-	-
Yes	38	22	14-42	0.68	0.44, 1.05	0.08
Work with hands above shoulder height for >1 hour [3] (censored 4 no, 1 yes; missing 9)						
No	147	20	12-33	1	-	-
Yes	34	19.5	13-33	0.89	0.60, 1.32	0.56
Work with neck bent forward for >2 hours [3] (censored 3 no, 2 yes; missing 3)						
No	114	21	13-39	1	-	-
Yes	73	18	8-31	1.19	0.88, 1.60	0.26
Work with neck twisted for >30 minutes [3] (censored 4 no, 1 yes; missing 7)						
No	136	20	12-34	1	-	-
Yes	47	22	13-32	0.93	0.66, 1.32	0.70
Drive for >1 hour per day [3] (censored 5 no; missing 5)						
No	115	21	13-38	1	-	-
Yes	70	16	7-31	1.22	0.88, 1.68	0.24

Total sample size n=195. Median duration of work absence relates to the 190 non-censored events.

1. Adjusted for age and sex.
2. Dichotomised by the median for the sample population
3. As part of the normal working day [9].

For peer review only

References

1. Sangha O, Stucki G, Liang MH et al. The self-administered comorbidity questionnaire: a new method to assess comorbidity for clinical and health services research *Arthritis & Rheumatism* 2003;49(2):156-63.
2. Terluin B, van Marwijk HW, Adèr HJ et al. The Four-Dimensional Symptom Questionnaire (4DSQ): a validation study of a multidimensional self-report questionnaire to assess distress, depression, anxiety and somatization. *BMC Psychiatry* 2006;6(1):34.
3. Ware J, Sherbourne C. The MOS 36-item short-form health survey (SF-36): I. conceptual framework and item selection. *Medical Care* 1992;30:473-83.
4. Hansen TB, Dalsgaard J, Meldgaard A et al. A prospective study of prognostic factors for duration of sick leave after endoscopic carpal tunnel release. *BMC Musculoskelet Disord* 2009;10:144.
5. Palmer K, Calnan M, Wainwright D et al. Upper limb pain in primary care: health beliefs, somatic distress, consulting and patient satisfaction. *Family Practice* 2006;23(6):609-17.
6. Bot AG, Becker SJ, Bruijnzeel H et al. Creation of the abbreviated measures of the Pain Catastrophizing Scale and the Short Health Anxiety Inventory: the PCS-4 and SHAI-5. *J Musculoskelet Pain* 2014;22(2):145-51.
7. Calfee RP, Dale AM, Ryan D et al. Performance of simplified scoring systems for hand diagrams in carpal tunnel syndrome screening. *J Hand Surg Am* 2012;37(1):10-17.
8. University of Michigan Medical School. The Michigan Hand Questionnaire: http://mhq.lab.medicine.umich.edu/. Accessed 04/04/2019.
9. Karasek R, Brisson C, Kawakami N et al. The Job Content Questionnaire (JCQ): an instrument for internationally comparative assessments of psychosocial job characteristics. *J Occup Health Psychol* 1998;3(4):322-55.

For peer review only

STROBE checklist (cohort study)

Item	Recommendation	Location
**Title and abstract**		
Title	Indicate study design	Title
**Introduction**		
Background	Explain scientific background and rationale	Introduction
Objectives	State objectives and any pre-specified hypotheses	Introduction
**Methods**		
Study design	Present key elements of the study design early	Introduction & methods
Setting	Describe the setting, locations and relevant dates	Methods
Participants	Give eligibility criteria, sources and methods of selection of participants, and methods of follow-up	Methods & table 1
Variables	Clearly define all outcomes, exposures, predictors, potential confounders and effect modifiers	Methods & supplementary files 1-2
Data sources	Give sources of data and methods of assessment	Methods & tables 2-5
Bias	Describe and effects to address potential sources of bias	Methods & discussion
Study size	Explain how the study size was determined	Methods
Quantitative variables	Described how quantitative variables were handled in the analyses	Tables 2-5
Statistical methods	Described all statistical methods<br>Explain how missing data were addressed<br>Explain how lost to follow-up was addressed	Statistical methods & tables 2-5
**Results**		
Participants	Reported the number of individuals at each stage of the study<br>Give reasons for non-participation	Figure 1<br>Figure 1
Descriptive data	Give characteristics of study participants<br>Indicate number of participants for each variables of interest<br>Summarise follow-up time	Tables 2-5<br>Tables 2-5<br>Results
Outcome data	Report number of outcome events over time	Results
Main results	Give adjusted and unadjusted estimates and measures of precision. Make clear which confounders were adjusted for	Table 4 & supplementary file 3
Other analyses	Reported any subgroup analyses	Table 4 & supplementary file 3

For peer review only
